# Peptide Backbone Editing via Post-Translational O to C Acyl Shift

**DOI:** 10.1021/jacs.4c14103

**Published:** 2025-02-11

**Authors:** Carly K. Schissel, Helena Roberts-Mataric, Isaac J. Garcia, Hana Kang, Riaz Mowzoon-Mogharrabi, Matthew B. Francis, Alanna Schepartz

**Affiliations:** † Department of Chemistry, University of California, Berkeley, California 94720, United States; ‡ Molecular and Cell Biology, University of California, Berkeley, California 94720, United States; § California Institute for Quantitative Biosciences, University of California, Berkeley, California 94720, United States; ∥ Chan Zuckerberg Biohub, San Francisco, California 94158, United States; ⊥ ARC Institute, Palo Alto, California 94304, United States

## Abstract

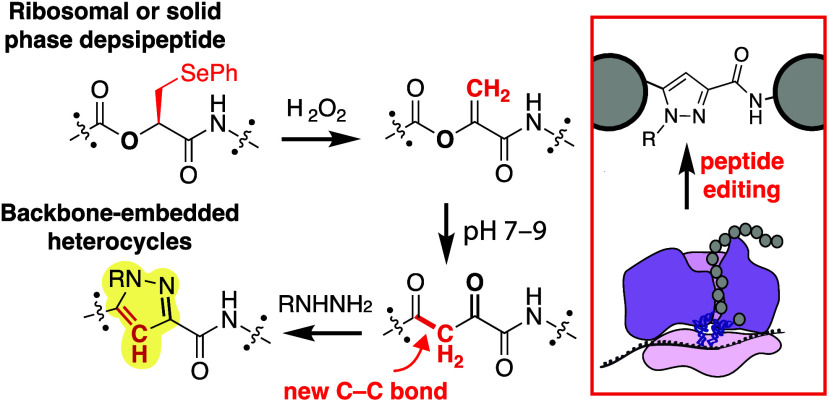

Despite tremendous efforts to engineer translational machinery, replacing the encoded peptide backbone with new-to-nature structures remains a significant challenge. C, H, O, and N are the elements of life, yet ribosomes are capable of forming only C–N bonds as amides, C–O bonds as esters, and C–S bonds as thioesters. There is no current strategy to site-selectively form C–C bonds as ketones embedded in the backbones of ribosomal products. As an alternative to direct ribosomal C–C bond formation, here we report that peptides containing a dehydrolactic acid motif rapidly isomerize to generate backbone-embedded α,γ-diketoamides via a spontaneous formal O to C acyl shift rearrangement. The dehydrolactic acid motif can be introduced into peptides ribosomally or via solid-phase synthesis using α-hydroxyphenylselenocysteine followed by oxidation. Subsequent incubation at physiological pH produces an α,γ-diketoamide that can be diversified using a variety of nucleophiles, including hydrazines and hydroxylamines, to form pyrazoles and oximes, respectively. All of these groups remain embedded directly within the polypeptide backbone. This general strategy for peptide backbone editing, predicated on an intricate cascade of acyl rearrangements, provides the first nonenzymatic example of a C–C bond forming reaction to take place within a peptide backbone. The products so-produced are easily diversified into protein-like materials with backbone-embedded heterocycles. Application of this peptide editing strategy should accelerate the discovery of genetically encoded molecules whose properties more closely resemble those of bioactive natural products.

## Introduction

**Figure 1:**
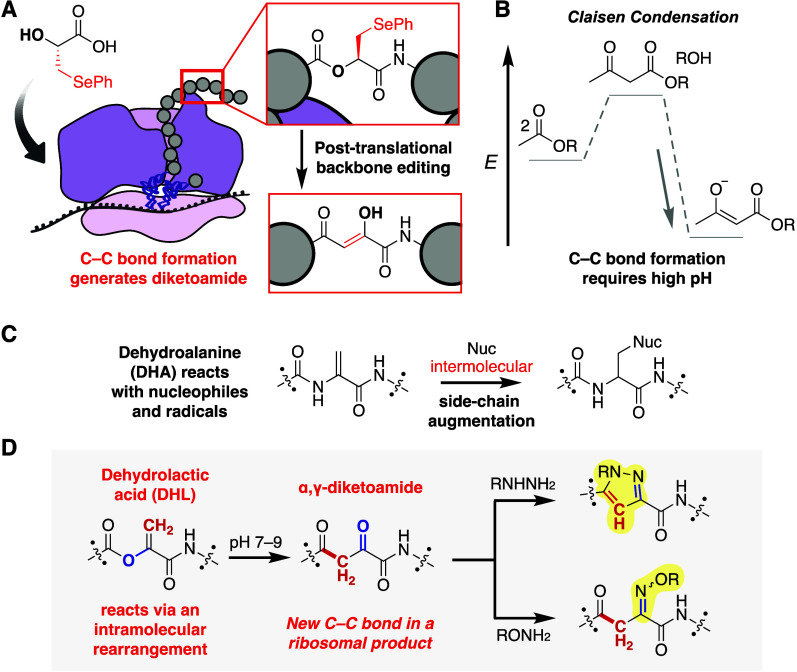
Dehydrolactic acid (DHL) rearrangement provides a strategy for post-translational C–C bond formation to install diketones in ribosomal products. (A) The strategy reported herein envisions the ribosomal translation of a polypeptide carrying a single α-hydroxy acid monomer whose side chain can be converted to an enol ester and rearrange into a backbone diketoamide motif. This rearrangement eliminates a depsipeptide bond and replaces it with a new C–C bond. (B) Simplified free energy diagram for the Claisen condensation, in which the C–C bond forming step is uphill, and which is driven downhill by deprotonation at high pH. (C) The unique reactivity of dehydroalanine (DHA) has been utilized previously by others for the post-translational installation of diverse side chains. (D) DHL resembles DHA but contains an enol ester once introduced into a polypeptide. Unlike DHA, DHL reacts preferentially in an intramolecular fashion to form an α,γ-diketoamide product embedded within the polypeptide backbone. The α,γ-diketoamide can be diversified using hydrazines to form pyrazoles and hydrazones and hydroxylamines to form oximes.

Ribosomes have evolved over billions of years to catalyze a single reaction: the formation of an amide bond between two α-amino acids. Recent work has shown that in addition to canonical and noncanonical α-amino acids,^1,2^ under cellular conditions ribosomes also promote reactions of α-hydroxy acids^3,4^ as well as certain β^2^-hydroxy^5^ and β^3^-amino acids.^6^ The substrate scope is somewhat expanded in vitro, where chemically preacylated tRNAs support ribosomal reactions of α-thio acids^7^ and a variety of non-α-amino acids, including N-terminal aramids and 1,3-dicarbonyls,^8,9^ α-aminoxy and α-hydrazino acids,^10^ and cyclic β-amino acids.^11^ Yet these products are all amides or esters; there is no current strategy to site-selectively form C–C bonds as ketones embedded in the backbone of a ribosomal product. Backbone ketone motifs are desirable for their unique reactivity toward nucleophiles, enabling late-stage orthogonal diversification. Moreover, editing the peptide backbone N–H bond suppresses its liability; amides are labile to proteolysis, and the N–H bond limits membrane permeability. We envision post-translational peptide backbone editing as a synthetic strategy to install C–C bonds in place of the native peptide bond, thus generating chimeric molecules that blend peptide and polyketide motifs (Figure 1A).

There are multiple challenges to generating C–C bonds in biological molecules and under physiological conditions. The first is the challenge of forming a long-lived carbon-centered nucleophile at neutral pH, especially in the absence of sterically encumbering electron-withdrawing groups. The second is thermodynamics: common C–C bond forming reactions, such as the Claisen condensation, face an uphill energy battle. The driving force in this reaction is a final deprotonation of a moderately acidic carbon at a high pH (Figure 1B). Polyketide synthase enzymes overcome these challenges via proximity-driven decarboxylation reactions; other enzymes install backbone ketones by excision via recognition of an 11-amino acid tag^12,13^ or biosynthetically at the peptide C-terminus.^14^ Finally, ketones can be appended synthetically to the side chains or termini of peptides made on the solid phase.^15−18^ However, none of these given examples install a ketone internally within the backbone, without a requisite recognition sequence and in a genetically programmable manner.

Herein, we report the discovery of a reaction cascade that edits the peptide backbone by replacing a backbone N–H bond with a new C–C bond. The product is a versatile α,γ-diketone, the fundamental element of polyketide natural products, which can be derivatized to form myriad acyclic and cyclic backbone products. The precursor for this reaction cascade can be incorporated into peptides directly by ribosomes or by chemical synthesis. Oxidation followed by incubation in physiological buffer promotes a rapid intramolecular isomerization that generates the α,γ-diketoamide. Subsequently, and in a manner reflective of classic dicarbonyl chemistry,^19,20^ the newly formed α,γ-diketoamide can be diversified to embed substituted pyrazoles and oximes within the polypeptide backbone, generating ribosomal products whose structures begin to capture the diversity of synthetic materials.

## Results and Discussion

### DHL Rearrangement Forms a New C–C Bond in a Model Tripeptide

As part of ongoing work to expand the chemistry of polypeptide backbones,^5,6,21,22^ we explored the reactivity of dehydrolactic acid (DHL). DHL is the ester analog of dehydroalanine (DHA),^23,24^ a species that may be generated within a polypeptide either biosynthetically^25,26^ or synthetically via oxidation of a selenocysteine analog^23^ or β-elimination of serine or cysteine heteroatoms.^27−29^ While DHA reacts readily in an intermolecular fashion at the electrophilic β-carbon^23,30^ (Figure 1C), we were surprised to discover that DHL reacts preferentially in an intramolecular fashion to form an α,γ-diketoamide. The result is a formal transposition of the β-carbon and α-oxygen as well as the formation of a new C–C bond (Figure 1D).

**Figure 2:**
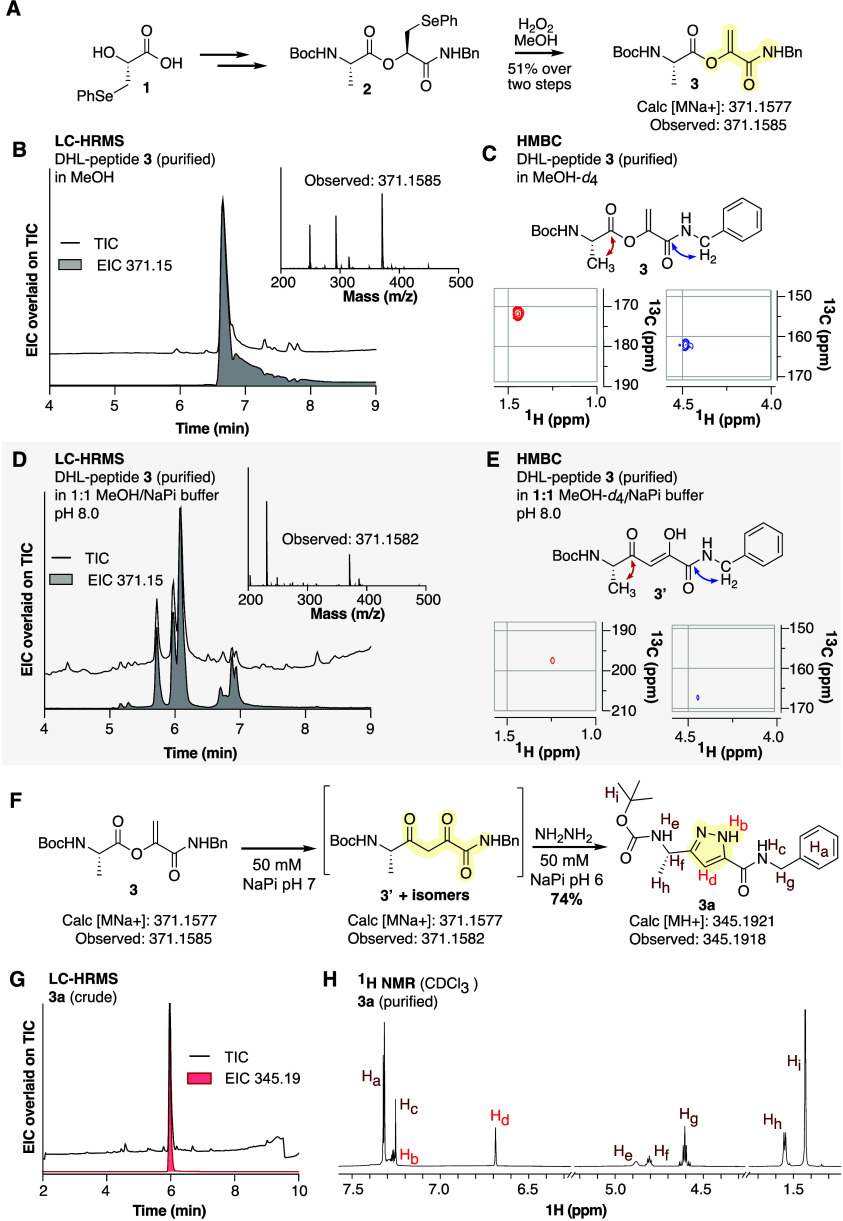
DHL-peptide **3** isomerizes into an α,γ-diketoamide at pH 7. (A) Scheme illustrating the structure of the DHL precursor HO-SecPh (**1**), its incorporation into tripeptide **2**, and oxidation to produce DHL-peptide **3**. (B) LC-HRMS chromatogram and spectrum of DHL-peptide **3**, which is stable in methanol. (C) Two sections of the HMBC NMR spectrum of DHL-peptide **3** in methanol-*d*_4_, showing correlation of the methyl protons with the ester carbonyl (left) and the benzyl protons with the amide carbonyl (right). (D) LC-HRMS chromatogram and spectrum of DHL-peptide **3** following 4 h of incubation in 50 mM NaP_i_ at pH 8. (E) Two sections of the HMBC NMR spectrum of DHL-peptide **3** in 50% methanol-*d*_4_ and 50 mM NaPi pH 8, showing correlation of the methyl protons with a newly formed ketone carbonyl (left) and the benzyl protons with the amide carbonyl (right). (F) Incubation of DHL-peptide **3** in 50 mM NaP_i_ at pH 7 followed by 20 mM hydrazine in 50 mM NaP_i_ at pH 6 generates a product containing a backbone pyrazole, **3a**. (G) LC-HRMS of crude **3a** showing the EIC overlaid on the TIC following reaction in (D). (H) ^1^H NMR confirms the structure of purified pyrazole-peptide **3a**.

The unprecedented DHL rearrangement to form a new C–C bond was first observed in the context of a model tripeptide. DHL was installed using an α-l-hydroxy-phenylselenocysteine (HO-SecPh) (**1**) precursor, which was synthesized via a known epoxide ring-opening reaction^31^ followed by hydrolysis, and introduced into tripeptide **2**. Treatment of tripeptide **2** with H_2_O_2_ in MeOH for 1 h afforded DHL-peptide **3**, which was purified to homogeneity using RP-HPLC and characterized by NMR and LC-HRMS (Figure 2A,B, Figure S1). The heteronuclear multiple bond correlation (HMBC) spectrum of **3** shows correlation between the Ala side-chain methyl protons and the ester carbonyl (171 ppm), as well as between the benzyl protons and the amide carbonyl (162 ppm) (Figure 2C, Figure S2). Although purified DHL-peptide **3** was stable in MeOH, the addition of 50 mM NaPi pH 8 for 15 min at RT led to the appearance of several chromatographically distinct species that possessed the same mass as DHL-peptide **3** when analyzed by LC-HRMS (Figure 2D). These isobaric species also arose from treatment of DHL-peptide **3** with tetramethyl guanidinium in MeOH and persisted for at least 8 h (Figure 2D, Figure S1). When isolated, each peak re-equilibrated into the same set of multiple peaks, suggesting that base treatment of DHL-peptide **3** and analysis by RP LC-MS resulted in a set of at least three interconverting isomers. At the same time, an analogue of DHL-peptide **3** containing a C-terminal methyl ester in place of the amide (**S4**) failed to isomerize under identical basic conditions as determined by LC-MS and NMR (Figure S2).

Although the isomers of DHL-peptide **3** formed in base could not be isolated, they could be partially characterized by NMR. The HMBC spectrum of purified DHL-peptide **3** in 50% 50 mM NaPi pH 8 in methanol-*d*_4_ revealed the presence of a single species containing the same benzyl proton–amide carbonyl cross-peak as purified **3** in methanol-*d*_4_ (167 ppm) (Figure 2E, Figure S3). However, the cross peak corresponding to the Ala methyl protons shifted downfield on the ^13^C channel to an apparent ketone region (197 ppm). A singlet methine peak also emerged at 5.9 ppm, suggesting that the species was enol **3′** (Figures S4, S5).^32^ While this single species is present under the basic buffer conditions as determined by NMR, multiple chromatographic peaks are observed by LC-HRMS because the diketone is subject to dynamic exchange under the acidic conditions of reverse phase chromatography.

We confirmed the structure of α,γ-diketoamide isomer **3′** by characterizing its conversion into pyrazole **3a**. DHL-peptide **3** was treated first with 50 mM NaP_i_ at pH 7 for 1 h at RT and then with excess hydrazine at pH 6, as appropriate for a classic Knorr pyrazole synthesis (Figure 2F).^20^ Within minutes after the addition of hydrazine, the chromatographically distinct but unisolable peaks evident in Figure 2D coalesced into a single chromatographic peak with a mass that corresponded to pyrazole **3a** (Figure 2G). Full characterization of the product by NMR confirmed the structure of pyrazole **3a** and by inference the structure of its direct precursor, α,γ-diketoamide **3′** (Figure 2H).

### Computational Studies Support a Dual Acyl-Shift Cascade Pathway for C–C Bond Formation

Acyl shifts are known to alter the peptide backbone. Intramolecular acyl shifts occur spontaneously and rapidly in the context of native chemical ligation (NCL)^33^ or during intein splicing.^34^ Recent work has shown that β-amine nucleophiles generated in proximity to a reactive acyl group promote O to N acyl shifts that establish β^2^-peptide linkages within short peptides in vitro.^35^ In these cases, the acyl shifts utilize a side chain nucleophile and generate a thermodynamically stabilized amide product. Although pseudointramolecular decarboxylative Claisen reactions generate new C–C bonds via S to C acyl shifts during polyketide biosynthesis, in this case the carbon nucleophile is generated enzymatically and transiently while held in proximity to the electrophile.^36^ Intramolecular Claisen-type rearrangements and O to C acyl shifts to form C–C bonds have precedent in chemical synthesis,^37,38^ but to our knowledge, there is no reported example of an acyl shift that generates a new C–C bond in a peptide backbone.

**Figure 3:**
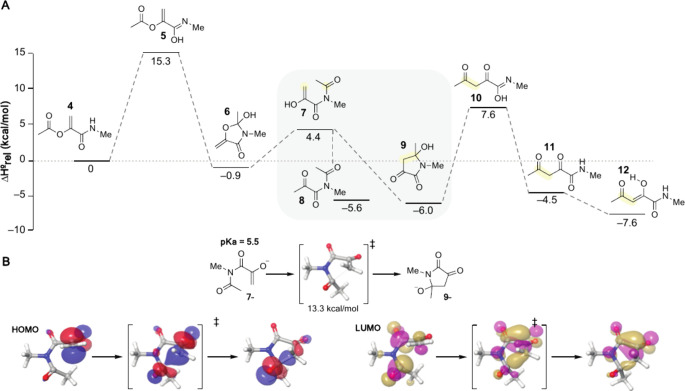
DFT studies suggest that peptides containing a dehydrolactic acid motif can isomerize via an overall O to C acyl shift. (A) Shown is an energy landscape illustrating the relative calculated enthalpies (Δ*H*°_(rel)_) of intermediates along the proposed pathway between DHL **4** and enol **12**. The pathway begins with the intramolecular cyclization of DHL tautomer **5** to form succinimide **6**. Cleavage of the tetrahedral intermediate C–O bond generates the crucial enol-containing intermediate **7**, which can tautomerize into unproductive intermediate **8** or cyclize with the acetamide carbonyl to generate the new C–C bond in pyrrolidine **9**. A final ring opening installs the second keto group in **11**, and subsequent tautomerization ultimately leads to low-energy enol **12**. For all species, geometry optimization and frequency calculations were performed at the B3LYP-D4/6-31G** level using an implicit CPCM solvation model. Final electronic energy calculations were performed using ωB97M-V/def2-TZVPPD with a CPCM solvation model. (B) Transition state analysis of the key C–C bond forming transformation from anionic enol **7**^–^ to pyrrolidine **9**^–^ revealed a barrier of 13.3 kcal/mol relative to **9**^–^ (top). Calculations of the HOMO (bottom left) and LUMO (bottom right) orbitals demonstrate good overlap throughout this transformation.

While O to C acyl shifts have precedent, the observed transformation of DHL proposed above does not. The DHL rearrangement pathway we envision is outlined in Figure 3. We suspect that the amide nitrogen initiates isomerization by reacting with the backbone ester, as seen during the formation of succinimides during isoaspartate and aspartimide formation.^39,40^ This reaction step could also proceed through imidate tautomer **5**, although a direct addition of nitrogen to the ester carbonyl cannot be ruled out. Following addition, cyclic intermediate **6** could ring-open to form transient enol intermediate **7**, which would be in equilibrium with its stable tautomer, **8**. Attack of the carbon of enol **7** on the proximal carbonyl of the acyl-imide would yield compound **9**, thus forging the key C–C bond of the rearrangement. This step is reminiscent of intramolecular Claisen rearrangements of allylic alcohols that are used to produce γ,δ-unsaturated esters stereoselectively^38^ or the O to C acyl shift employed in the synthesis of benzofurans.^37^ A second ring-opening step forms the diketone species **10**, which tautomerizes to low-energy diketoamide **11** and enol **12**. DFT calculations support that the intermediates on this pathway are energetically accessible and predict an overall enthalpy change of −7.6 kcal/mol for the full transformation (Figure 3A, Figure S6).

We were especially intrigued by two elements of the proposed transformation. The first is the enol proton p*K*_a_. In simple enols, the p*K*_a_ of this proton is 10 or above, limiting the availability of the corresponding enolate at physiological pH. However, the calculated p*K*_a_ of enol **7** is only 5.5, presumably because of the inductive effect of the adjacent carbonyl. This lower value implies that the deprotonated, and hence more nucleophilic, form of enol **7** would predominate at neutral pH.

The second intriguing element of the proposed transformation is the C–C bond forming step. The proposed mechanism includes a 5-(enolendo)-exo-trig cyclization, a pathway classically disallowed by Baldwin’s stereoelectronic rules for enolates due to poor orbital overlap.^41^ Although rare, previous exceptions to Baldwin’s rules for 5-(enolendo)-exo-trig cyclizations have been reported.^42^ To better understand the energetics of this cyclization, we performed additional DFT calculations to evaluate the transition state of the critical C–C bond forming step, the cyclization of enolate **7**^**–**^ into pyrrolidine **9**^**–**^. A transition state candidate was found along a well-defined intrinsic reaction coordinate, which was 13.3 kcal above the energy of the anion of **9**^**–**^ (Figure 3B, Figure S7). The feasibility of the C–C bond forming step was further supported by examination of the calculated HOMO and LUMO during these transformations. Visualization of the mixing of these frontier orbitals in the transition state that connects enolate **7**^**–**^ to anionic pyrrolidine **9**^**–**^ reveals good overlap (Figure S8, Supplemental Video).

### DHL Rearrangements Are Efficient in Tripeptides

**Figure 4:**
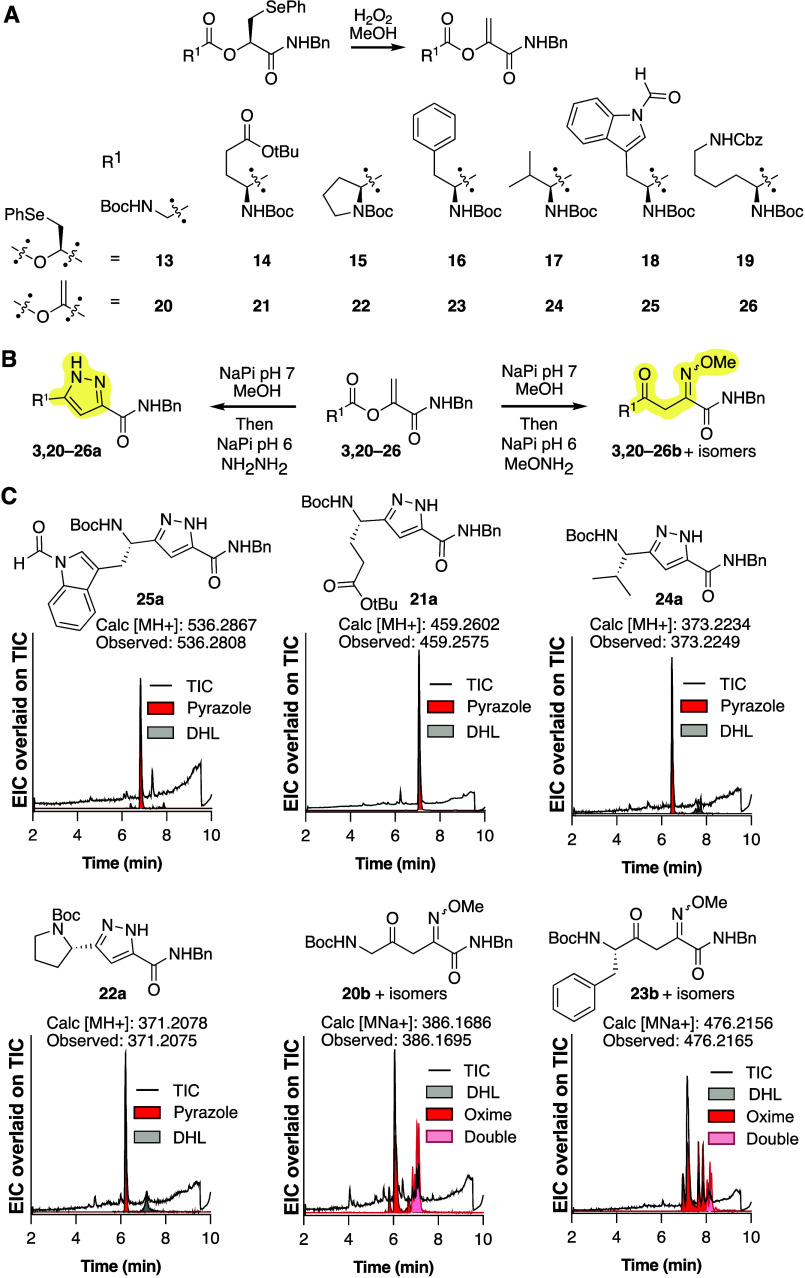
DHL rearrangements proceed in multiple contexts, and the α,γ-diketoamide products react with α-nucleophiles. (A) Seven additional tripeptides, **13**–**19**, containing the DHL precursor HO-SecPh were prepared and oxidized to generate DHL-peptides **20**–**26**. (B) DHL-containing tripeptides isomerized in buffer at pH 7 for 1 h were subsequently reacted with either hydrazine to form pyrazoles or *O*-methyl hydroxylamine to form oximes. (C) LC-HRMS chromatograms showing the EIC (red) corresponding to pyrazole-peptides **21a**, **22a**, **24a**, and **25a** and oxime-peptides **20b** and **23b**. Pink EIC traces indicate oxime formation on both ketones, while gray traces indicate the residual DHL starting material, overlaid on the TIC.

Next we explored whether the DHL rearrangement to generate a reactive α,γ-diketoamide would proceed in diverse α-amino acid contexts. We synthesized seven analogs of tripeptide **2** in which the N-terminal Boc-Ala was replaced by either Boc-Gly (**13**), Boc-Glu(OtBu) (**14**), Boc-Pro (**15**), Boc-Phe (**16**), Boc-Val (**17**), Boc-Trp(for) (**18**), or Boc-Lys(Cbz) (**19**) (Figure 4A). Treatment of each tripeptide with H_2_O_2_ in MeOH afforded the corresponding DHL-peptides **20**–**26** within an hour as determined by LC-HRMS. The new DHL-peptides (**20**–**26**) were purified by RP-HPLC, and their structures were confirmed by NMR. Each DHL-peptide was incubated at pH 7 for 1 h and then combined at pH 6 with hydrazine (**a**) or *O*-methyl hydroxylamine (**b**) (Figure 4B, Figures S9, S10). Reactions were incubated for 6 h at RT and analyzed by LC-HRMS.

All DHL-peptides were transformed into their respective pyrazoles and oximes under these conditions. Pyrazole formation was quantitative for DHL-peptides containing an N-terminal Ala, Gly, Glu(OtBu), or Trp(for) residue, while reactions of DHL-peptides containing an N-terminal Pro, Phe, Val, or Lys(Cbz) contained a small amount of residual DHL-peptide as determined by LC-HRMS. These observations suggest that rearrangement is most efficient with reduced steric hindrance near the ester carbonyl and proceeds in the presence of protected amines and acids. Example chromatograms are shown for pyrazole-peptides **21a**, **22a**, **24a**, and **25a** and oxime-peptides **20b** and **23b** (Figure 4C). We observed the double addition of *O*-methyl hydroxylamine to the model tripeptides, further confirming the presence of two ketone motifs. Oxime formation can occur on either ketone, and each oxime can exist as the *E* or *Z* isomer, as indicated by the multiple chromatographic peaks observed for the product.

### DHL Rearrangements Edit the Backbones of Multiple Peptides Prepared on the Solid Phase

**Figure 5:**
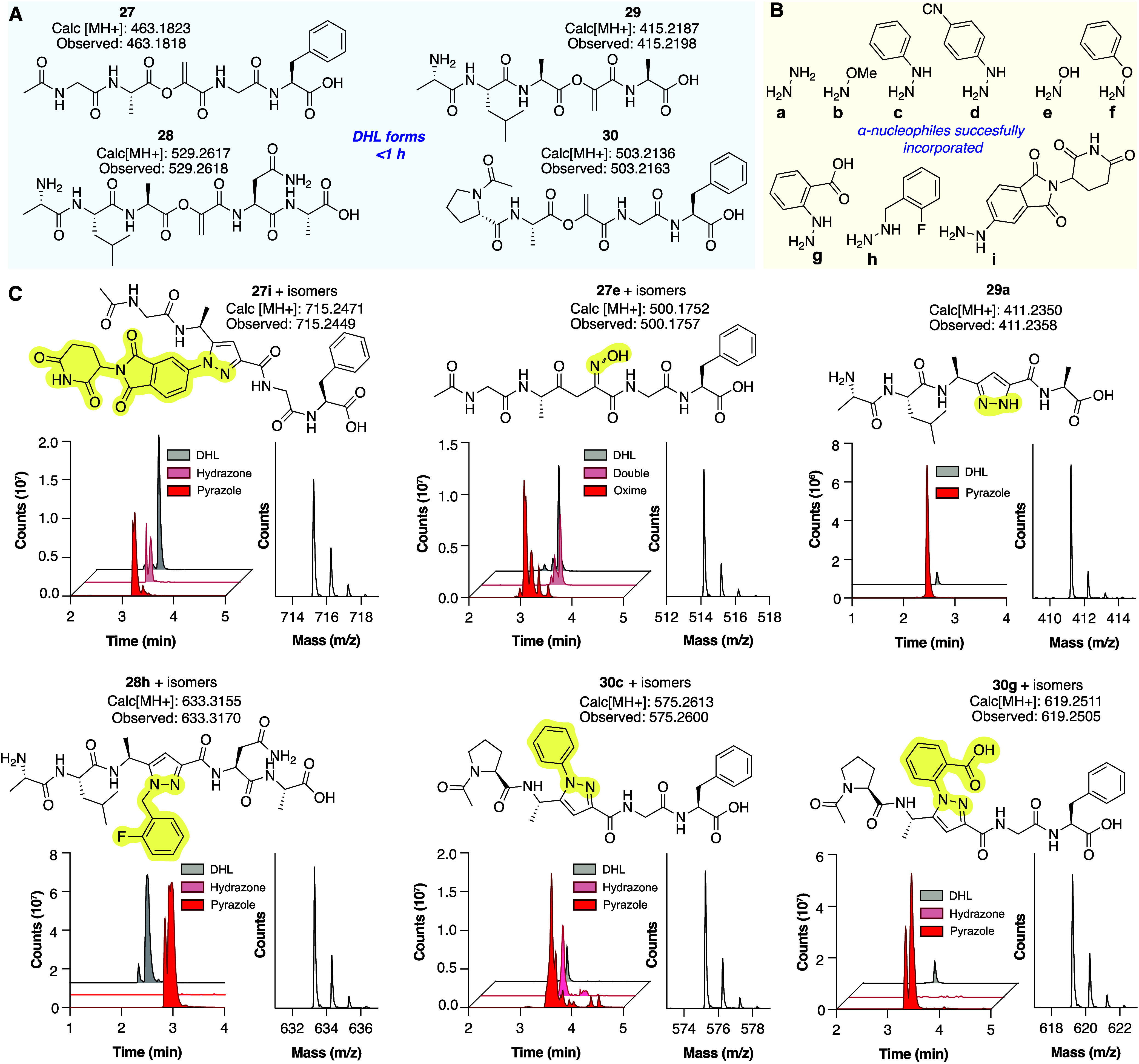
DHL-containing peptides prepared via solid-phase peptide synthesis can be diversified into oximes and pyrazoles following an O to C acyl shift. (A) Structures of DHL-peptides **27**–**30**. (B) Structures of α-nucleophiles a–i used to make pyrazole- and oxime-peptides. (C) Structures of pyrazole- and oxime-peptides and corresponding EICs and mass spectra from LC-HRMS following reactions with the α-nucleophiles, including thalidomide-peptide **27i**, oxime **27e**, pyrazole-peptide **29a**, fluoro-benzyl pyrazole-peptide **28h**, phenyl-pyrazole **30c**, and benzoic acid-pyrazole **30g**. EICs include the remaining starting material DHL (gray), product pyrazole or a single oxime (red), and product hydrazone, indicating incomplete pyrazole formation or double oxime formation (pink).

The α,γ-diketoamide functional group provides a new strategy for peptide diversification because of the ease with which it can be orthogonally labeled and diversified into drug-like motifs. Incorporation of ketones into polypeptides (>5 amino acids) is challenging on the solid phase and is often limited to reactions of side chains or the N- or C-termini.^15−17^ To evaluate whether DHL rearrangements could edit the backbones of peptides prepared using solid phase methods, we synthesized a series of hexa- or heptapeptides containing an internal HO-SecPh monomer. The HO-SecPh monomer was installed using a depsi-dipeptide block^43^ containing HO-SecPh (**1**) preceded by Gly (**S7**) or Ala (**S8**) and containing a variety of natural side chains. Following resin cleavage, deprotection, and purification, these peptides were treated with H_2_O_2_ to generate DHL-peptides **27**–**30** (Figure 5A, SI Section 4). Although oxidative elimination to generate peptides **27**–**30** proceeded slowly in water (>1 h) and was accompanied in certain cases by ester hydrolysis, the reaction was accelerated in aprotic solvents such as acetonitrile (Figures S11–S13).^44^ DHL-peptides **27**–**30** formed within 1 h in MeCN and H_2_O_2_ and were dried via lyophilization. All peptides were characterized by LC-HRMS, and DHL-peptides **27** and **30** and their precursors were also characterized by NMR (SI Section 3).

DHL-peptides **27**–**30** were then isomerized in 50 mM NaP_i_ at pH 7–9 for varying times and derivatized with a variety of substituted hydrazines and hydroxylamines (**a**–**i**) (Figure 5B). We first investigated the isomerization of DHL-peptides **27**–**30** in buffered solution at pH 7, 8, or 9. Samples were incubated between 15 min and 4 h before dilution in pH 6 NaP_i_ containing excess hydrazine. While higher pH enhanced the rate of isomerization, the majority of the sequences isomerized fully within 1 h at pH 8 and were converted to the corresponding pyrazole-peptide within 15 min at pH 6 (Figures S14, S15). The exception was Pro-containing DHL-peptide **30**, which required a 4 h incubation in NaP_i_ at pH 9 and then excess hydrazine in NaP_i_ at pH 6 for 1 h to convert fully into pyrazole peptide **30a** (Figure S16). Pyrazole-peptides **27a** and **30a** were also characterized by NMR (SI Section 3).

DHL peptides **27**–**30** could also be diversified with α-nucleophiles other than hydrazine. The highest yielding reactions occurred with electron-rich hydrazines, such as 2-hydrazine-benzoic acid (**g**) and 2-fluoro-benzyl hydrazine (**h**), while reactions with the electron-poor 4-cyano-phenyl hydrazine (**d**) proceeded in a lower yield. *O*-Benzyl hydroxylamine (**f**) and *O*-methyl hydroxylamine (**b**) also efficiently formed their respective oximes. Finally, the thalidomide hydrazine (**i**), an analog of a known binder to the E3 ligase CBRN, also led to substituted pyrazole formation in the case of peptides **27i** and **30i**. Example chromatograms illustrating reactions to generate thalidomide-peptide **27i**, oxime **27e**, pyrazole-peptide **29a**, fluoro-benzyl pyrazole-peptide **28h**, phenyl-pyrazole **30c**, and benzoic acid-pyrazole **30g** are shown in Figure 5C, with the remainder shown in the Supporting Information (Figures S17–S20). Substituted pyrazole peptides **27h**, **29g**, and **30g** were purified by RP-HPLC and characterized by LC-HRMS (Figure S21). These labeling reactions demonstrate the ease with which DHL-peptides prepared via SPPS can be diversified into a wide array of heterocyclic or bioconjugated materials.

### DHL Rearrangements Edit the Polypeptide Backbones of Ribosomally Synthesized Peptides

**Figure 6:**
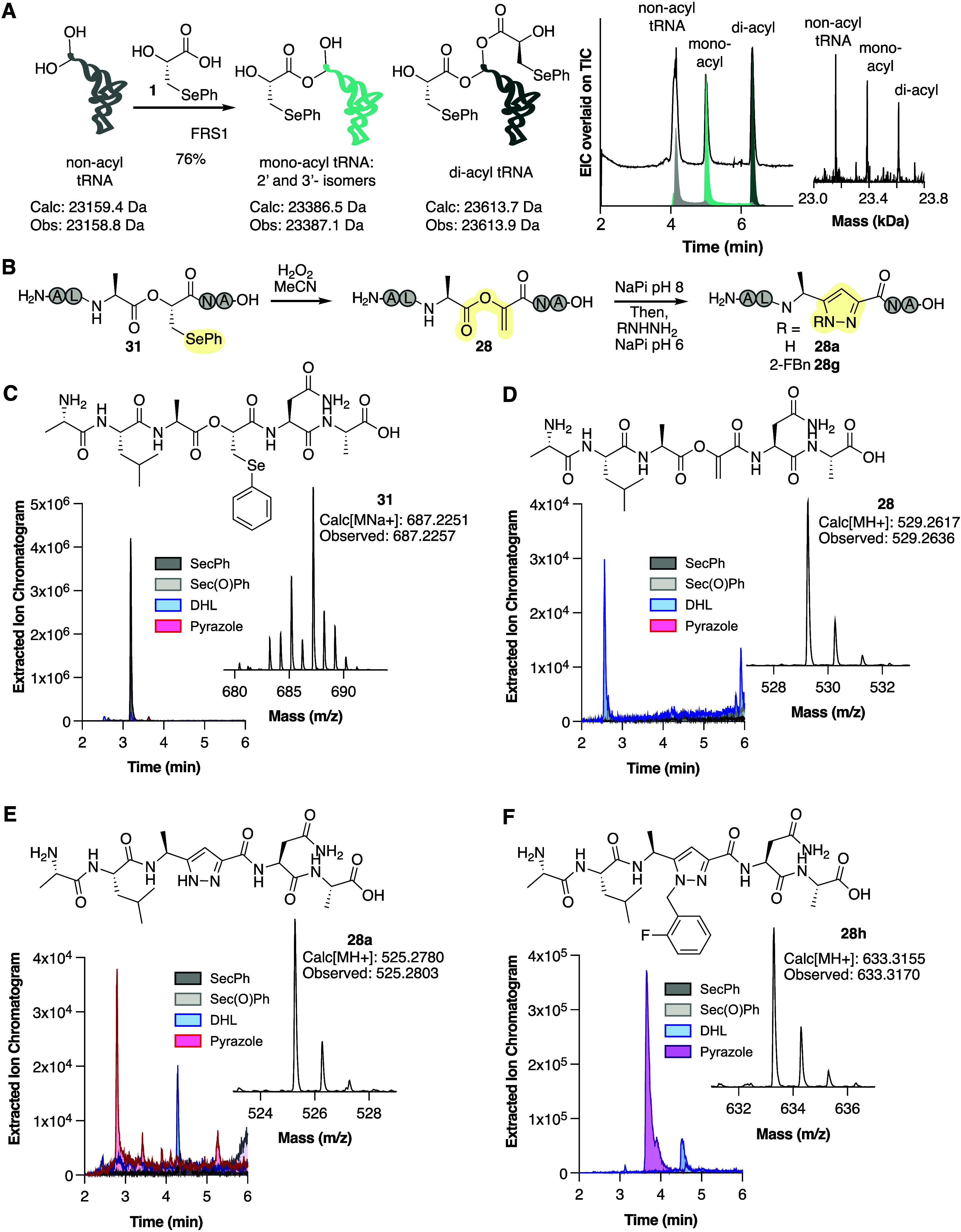
Genetic encoding of HO-SecPh-peptide and transformation into pyrazole-peptide. (A) Scheme illustrating the acylation of tRNA^Pyl^_Val_ with HO-SecPh **1** using the *M. alvus* PylRS variant FRS1. Shown is the LC-MS total ion chromatogram (TIC) of the product mixture overlaid with the extracted ion chromatograms (EIC) corresponding to unreacted tRNA^Pyl^_Val_ (nonacyl tRNA), monoacylated tRNA^Pyl^_Val_ (monoacyl), and diacylated tRNA^Pyl^_Val_ (diacyl) following incubation with HO-SecPh **1** and FRS1. This mixture was added to an in vitro translation reaction along with cDNA coding for peptide MALAVNA (**S11**), WT translation machinery, and the amino acids Ala, Leu, and Asn to generate HO-SecPh-containing peptide **31**. (B) Scheme illustrating the oxidation of HO-SecPh-containing peptide **31** into DHL-peptide **28** and its diversification into pyrazole **28a** and **28h**. (C–F) Structures, extracted ion chromatograms, and mass spectra of (C) IVT-generated HO-SecPh-containing peptide **31**; (D) DHL-peptide **28**; (E) pyrazole **28a**; and (F) 2-fluorobenzoic acid pyrazole-peptide **28h**.

Encouraged by the reactivity described above, we sought to establish the conditions needed to support DHL rearrangements and modifications of genetically encoded polypeptides prepared by in vitro translation. We evaluated several synthetase/tRNA pairs to generate an appropriate acyl-tRNA, including PhSeRS-K4, which was engineered to incorporate SecPh.^45^ We found the highest activity using FRS1, an analog of *M. alvus* PylRS that acylates tRNA^Pyl^ with several α-hydroxy phenylalanine derivatives.^21,46^ FRS1 acylated tRNA^Pyl^_Val_ with HO-SecPh (**1**) within 4 h at 37 °C to produce a mixture of mono- and diacylated tRNA^Pyl^_Val_ products in 76% yield, confirmed by triplicate experiments (Figure 6A). The acylated tRNA^Pyl^_Val_ products were added to a commercial in vitro translation system composed of purified components^47^ (PureExpress, NEB) lacking Met and Val, along with cDNA encoding MALAVNA (**S11**), with Val as the recoded position (Figure 6B, Figure S22). After 2 h at 37 °C, the peptide products were isolated and desalted; LC-HRMS revealed the efficient biosynthesis of peptide **31** (Figure 6C, Figure S22). We found desalting to be necessary before DHL formation, likely due to excess reducing agent in the PureExpress translation mixture.

The ribosomal product **31** was then oxidized, isomerized, and subjected to Knorr pyrazole synthesis. The desalted translation mixture containing **31** was first treated with 100 mM H_2_O_2_ in MeCN for 9 h, and the emergence of DHL peptide **28** was detected by LC-HRMS (Figure 6D). The peptide was then lyophilized to remove the oxidant and reconstituted in 50 mM NaP_i_ at pH 8 to isomerize the mixture for 2 h. Subsequent addition of 20 mM hydrazine in 50 mM NaP_i_ at pH 6 afforded pyrazole-peptide **28a** (Figure 6E, Figure S23). DHL-peptide **28** was also diversified with 2-fluorobenzyl hydrazine, resulting in substituted pyrazole-peptide **28g** (Figure 6F, Figure S23).

## Conclusion

While much of the 10^7^-fold improvement in peptide bond formation arises from induced proximity,^48^ ribosomal catalysis has thus far been limited to O, N, and S nucleophiles to form carbon–heteroatom bonds. C–C bonds have yet to be formed within the ribosomal peptidyl transferase center (PTC) because of the challenges associated with controlling the reactivity of a carbanion in water. The DHL rearrangement reported herein overcomes this limitation by revealing the carbon nucleophile only as a reactive intermediate and in the proximity of an equally reactive carbonyl. The result is a new C–C bond in the form of a versatile α,γ-diketone that is replete among polyketide natural product precursors. While α,γ-diketones can be generated biosynthetically using mixed multienzyme nonribosomal cascades, here we show that similar products can be genetically encoded using the ribosome and post-translational chemistry. Like a polyketide, the α,γ-diketoamide produced by DHL rearrangement can be subsequently diversified to generate genetically encoded materials with diverse and valuable heterocycles embedded within the peptide backbone.

We envision the incorporation of DHL will significantly impact the ability to install nonpeptidic elements into genetically encoded peptides or proteins, in vitro or in cells. This advancement provides otherwise nonexistent opportunities to expand protein and polypeptide structure and function. For example, cyclic peptides are emerging as powerful therapeutic candidates, but often suffer from proteolysis and poor cell permeability due to solvent-exposed amide protons,^49^ and backbone *N*-methylation is a hallmark of bioactive cyclic peptides and natural products.^50^ Unnatural peptide backbones, such as additional methylenes or heterocycles, can enhance membrane permeability while adding chemical diversity.^51,52^ While recent years have seen notable advances in the ribosomal synthesis of multiple types of amide bonds, no general strategy exists to embed genetically encoded C–C bonds as ketones internally within the polypeptide backbone. We anticipate that the ease of incorporating DHL into synthetic and ribosomal peptides will inspire further transformations that allow for post-translational polypeptide backbone editing, even in a cellular context.

## Supporting Information Available

Computational data summary (XLSX)
Video illustrating the C–C bond formation with and without HOMO and LUMO orbitals displayed, as calculated using Density Functional Theory (DFT) (MOV)
Video illustrating the C–C bond formation with and without HOMO and LUMO orbitals displayed, as calculated using Density Functional Theory (DFT) (MOV)
Supporting information and methods; supplementary figures; supplementary tables; NMR spectra; LC-HRMS chromatograms (PDF)

## Supplementary Material

ja4c14103_si_001.xlsx

ja4c14103_si_002.mov

ja4c14103_si_003.mov
