# Supplementary material for: Peptide Backbone Editing via Post-Translational O to C Acyl Shift
Source: J Am Chem Soc. 2025 Feb 11;147(8):6503–13. doi: 10.1021/jacs.4c14103 (PMC11869294; doi:10.1021/jacs.4c14103)
Supplement: Supplementary file 4 — ja4c14103_si_004.pdf [file ja4c14103_si_004.pdf]

Supplementary Information for

Peptide Backbone Editing via Post-Translational O to C Acyl Shift

Carly K. Schissel[1], Helena Roberts-Mataric[1], Isaac J. Garcia[1], Hana Kang[1], Riaz Mowzoon-Mogharrabi[1], Matthew B. Francis[1*], Alanna Schepartz[1,2,3,4,5,6*]

[1]Department of Chemistry, University of California, Berkeley, CA 94720, USA
[2]Molecular and Cell Biology, University of California, Berkeley, CA 94720, USA
[3]California Institute for Quantitative Biosciences, University of California, Berkeley, CA 94720, USA
[4]Chan Zuckerberg Biohub, San Francisco, CA 94158, USA
[5]Innovation Investigator, ARC Institute, Palo Alto, CA 94304, USA
[6]Lead contact: Alanna Schepartz
*Correspondence: Matthew Francis (mfrancis@berkeley.edu); Alanna Schepartz (schepartz@berkeley.edu)

Table of Contents

Synthetic Methods **3**
General Synthetic Details 3
Synthesis of α-hydroxy-phenylselenocysteine and derivatives 4
Synthesis of DHL-tripeptides 5
Ester block synthesis 12
Methods to Support Figure 2 17
Computational methods **17**
Methods to Support Figure 3 17
General Peptide Synthesis & Labeling Methods **18**
Methods to Support Figure 4 18
Solid-phase peptide synthesis methods to support Figure 5 19
Methods for labeling SPPS-synthesized peptides (to support Figure 5) 19
General Biochemistry Methods **19**
Methods to Support Figure 6A 19
Methods to Support Figure 6B–F 20
References **21**
Supplementary Materials Section 1: Supplementary Figures **22**
Supplementary Materials Section 2: Supplementary Tables **47**
Supplementary Materials Section 3: Processed NMR spectra **48**
Supplementary Materials Section 4: LC chromatograms & mass spectra **110**
Supplementary Materials Section 5: DFT Coordinates **119**

Synthetic Methods

General Synthetic Details

All reactions were carried out under ambient atmosphere unless otherwise noted. Room temperature (rt or RT) is defined as 21–23 °C. All reagents were obtained from commercial sources and used without further purification, unless otherwise noted. Deionized water was used for reactions, extraction solutions, and reverse phase chromatography. Acetonitrile and ethyl acetate used for chromatography were High-Performance Liquid Chromatography (HPLC) grade, while hexanes and methanol used for chromatography was certified ACS grade. Analytical thin-layer chromatography (TLC) was performed using 60 Å Silica Gel F254 pre-coated plates (0.25 mm thickness). TLC plates were visualized by irradiation with a UV lamp or with Hanessian's stain (cerium ammonium molybdate). Flash chromatography was performed using a Teledyne ISCO Combiflash NextGen 300+ equipped with a 4, 12, or 24 g RediSep columns for normal phase column chromatography. Reverse phase HPLC purification was performed on an Waters LC Prep 150 system equipped with a Waters 2998 UV photodiode array detector, a Waters 2707 autosampler, and an Waters Fraction Collector III using a preparative reverse phase C18 column (CSH C18 19 x 150 mm OBD Column 5 μm). The mobile phase for HPLC was water with 0.1% (v/v) trifluoroacetic acid (solvent A) and acetonitrile with 0.1% (v/v) trifluoroacetic acid (solvent B), at a flow rate of 20 mL/min. Analytes were collected based on their absorbance at 280 nm or 214 nm. Routine ^{1}H nuclear magnetic resonance (NMR) spectra were recorded on a Bruker 500, or 600 MHz spectrometers at ambient temperature unless otherwise stated. Chloroform-*d* was purchased from Cambridge Isotope Laboratories and used without further purification. Methanol-d_4 was purchased from Sigma Aldrich. Spectra were processed using MestReNova 14.2.0 using the automatic phasing, polynomial baseline correction capabilities, and zero-filling. Splitting was determined using the automatic multiplet analysis function with intervention as necessary. Spectral data are reported as follows: chemical shift (multiplicity [singlet (s), broad singlet (br s), doublet (d), triplet (t), quartet (q), pentet (p), multiplet (m), doublet of doublets (dd), doublet of doublet of doublets (ddd), doublet of triplet of doublets (dtd), doublet of doublet of doublet of doublets (dddd), doublet of triplets (dt), triplet of doublets (td), etc.], coupling constant (Hz), integration). The abbreviation "app" denotes an apparent multiplicity (e.g., "app t" denotes an apparent triplet). Chemical shifts are reported in parts per million (ppm, δ), and coupling constants are reported in Hz. ^{1}H resonances are referenced to solvent residual peaks for $CDCl_3$ (7.26 ppm) or to methanol-d_4 (3.31 ppm)[1]. Routine ^{13}C NMR spectra were recorded on a Bruker 500 (126) or 600 (151) MHz spectrometers with protons fully decoupled. ^{13}C Resonances are reported in ppm relative to solvent residual peaks for $CDCl_3$ (77.16 ppm) or DMSO-d_6 (39.52 ppm) or methanol-d_4 (49.00 ppm).

Synthesis of α-hydroxy-phenylselenocysteine and derivatives

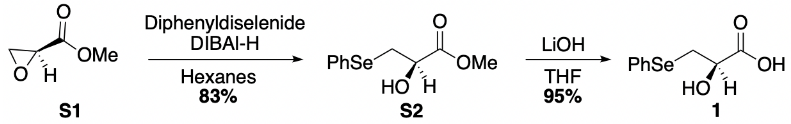

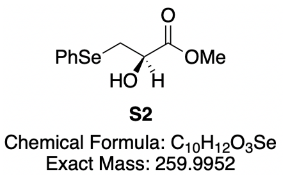

Methyl (R)-2-hydroxy-3-(phenylselanyl)propanoate (S2)

(Following a procedure adapted from Bartlett et al., 1983).[1] Hexanes (180 mL) and methyl (2S)-glycidate (6.1 g, 0.06 mol) were added to a dried flask under nitrogen and cooled to -78 °C. In a separate flask, diphenyldiselenide (9.4 g, 0.03 mol) was added to a dried flask under nitrogen, and a solution of DIBALH (60 mL, 0.06 mol) was added with stirring. Once this mixture had fully dissolved and the evolution of gas ceased, it was transferred dropwise with stirring to the flask containing **16**. The mixture was stirred for 2 h at -78 °C before being allowed to warm slowly to room temperature; stirring was continued for an additional 16 h. Concentrated phosphate buffer (500 mL, pH 3) was then added until the mixture reached pH 3. Saturated Rochelle's salt (500 mL) was added, and the aqueous phase was extracted three times with EtOAc (200 mL). Combined organic layers were dried over Na_2SO_4, concentrated under vacuum, and purified by flash chromatography (0-100% EtOAc) to afford **S2** as a yellow oil (9.4 g, 60%).

^{1}H NMR (600 MHz, $CDCl_3$): δ 7.57 – 7.54 (m, 2H), 7.28 – 7.24 (m, 3H), 4.47 (dd, *J* = 5.4, 4.4 Hz, 1H), 3.58 (s, 3H), 3.34 (dd, *J* = 13.1, 4.4 Hz, 1H), 3.25 (dd, *J* = 13.1, 5.4);

^{13}C NMR (150 MHz, $CDCl_3$): δ 173.4, 133.6, 129.3, 127.6, 69.6, 52.6, 32.8

LC-HRMS (ESI) *m/z* calcd. for $C_{10}H_{12}NaO_3Se^+$ $[MNa]^+$: 282.9844, found: 282.9839

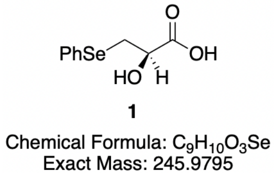

(R)-2-hydroxy-3-(phenylselanyl)propanoic acid (OH-SecPh) (1)

To a vial containing **S2** (3.7 g, 14 mmol) was added a 2 M solution of LiOH in THF (36 mL, 71 mmol) with stirring. After 30 minutes, 2 M HCl (20 mL) was added, and the aqueous mixture was extracted three times with EtOAc. The combined organic layer was dried over Na_2SO_4, filtered, and concentrated under vacuum to yield **1** as a pale yellow solid (3.5 g, 99%).

^{1}H NMR (600 MHz, $CDCl_3$): δ 7.57 – 7.54 (m, 2H), 7.27 – 7.23 (m, 3H), 4.45 (dd, *J* = 6.5, 4.2 Hz, 1H), 3.39 (dd, *J* = 13.2, 4.2 Hz, 1H), 3.22 (dd, *J* = 13.2, 6.5 Hz, 1H);

^{13}C NMR (150 MHz, $CDCl_3$): δ 176.6, 133.7, 129.4, 128.7, 127.9, 69.4, 32.5;

LC-HRMS (ESI) *m/z* calcd. for $C_9H_{10}NaO_3Se^+$ $[MNa]^+$: 268.9687, found: 268.9670

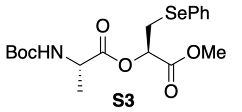

Chemical Formula: $C_{18}H_{25}NO_6Se$
Exact Mass: 431.08

Methyl (*R*)-2-(((*tert*-butoxycarbonyl)-*L*-alanyl)oxy)-3-(phenylselanyl)propanoate (S3)
NMI (154 µL, 1.9 mmol) was added to a solution of **S2** (100 mg, 0.4 mmol) and Boc-Ala-OH (183 mg, 1.0 mmol) in DCM (2 mL) at room temperature. The reaction was stirred until completion as monitored by TLC. The crude reaction mixture was then concentrated under reduced pressure and the resulting residue was purified by RP-HPLC (5-95% acetonitrile/water with 0.1% TFA over 30 min) and lyophilized to yield S3 as a white solid (142 mg, 85%).
^{1}H NMR (600 MHz, $CDCl_3$): δ 7.58 – 7.54 (m, 3.3 Hz, 2H), 7.30 – 7.27 (m, 3H), 5.34 (dt, *J* = 7.2, 3.3 Hz, 1H), 4.92 (s, 1H), 4.31 – 4.23 (m, 1H), 3.67 (s, 3H), 3.35 (dd, *J* = 13.4, 4.0 Hz, 1H), 3.26 (dd, *J* = 13.3, 7.4, 1H), 1.59 (s, 3H), 1.45 (s, 9H), 1.38 (d, *J* = 7.2 Hz, 3H);
^{13}C NMR (150 MHz, $CDCl_3$): δ 172.72, 168.80, 133.86, 129.37, 129.07, 127.91, 72.57, 52.66, 49.12, 28.47, 27.85, 18.58;
LC-HRMS (ESI) *m/z* calcd. for $C_{18}H_{25}NNaO_6Se^+$ $[MNa]^+$: 454.0739, found: 454.0750

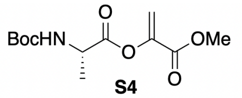

Chemical Formula: $C_{12}H_{19}NO_6$
Exact Mass: 273.12

Methyl 2-(((*tert*-butoxycarbonyl)-*L*-alanyl)oxy)acrylate (S4)
S3 was combined with MeOH (3 mL) and hydrogen peroxide (117 µL of 50% solution, 1.8 mmol) with stirring. Upon completion as determined by LC-MS, the mixture was purified by RP-HPLC (5-95% acetonitrile/water with 0.1% TFA over 30 min) to yield the DHL-containing peptide **S4** as a white solid (21 mg, 66%).
^{1}H NMR (600 MHz, MeOD): δ 6.06 (d, *J* = 2.0 Hz, 1H), 5.57 (d, *J* = 1.9 Hz, 1H), 4.29 (q, *J* = 7.4 Hz, 1H), 3.78 (s, 3H), 1.46 – 1.44 (m, 12H);
^{13}C NMR (150 MHz, MeOD): δ 173.04, 163.10, 157.84, 145.99, 114.93, 80.67, 53.03, 50.47, 28.68, 17.34;
LC-HRMS (ESI) *m/z* calcd. for $C_{12}H_{19}NaO_6^+$ $[MNa]^+$: 296.1105, found: 296.1087

Synthesis of DHL-tripeptides

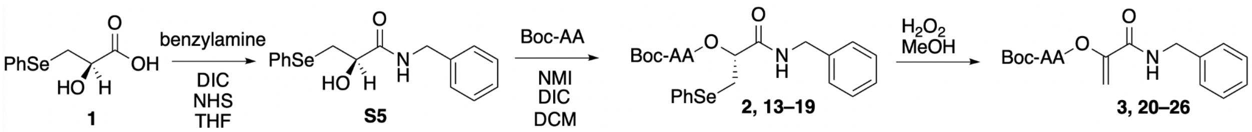

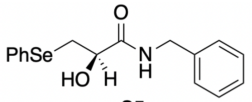

S5

Chemical Formula: $C_{16}H_{17}NO_2Se$
Exact Mass: 335.0425

(*R*)-*N*-benzyl-2-hydroxy-3-(phenylselanyl)propanamide (S5)

To a vial containing **1** (500 mg, 2.0 mmol) was added NHS (235 mg, 2.0 mmol) and anhydrous THF (5 mL) with stirring. Then, DIC was added (319 µL, 2.0 mmol) and the reaction was stirred until completion as monitored via LC-MS. The solution was then purified directly by flash chromatography (0-100% EtOAc/Hexanes) to yield **S3** as a white solid (547 mg, 80%).

^{1}H NMR (500 MHz, $CDCl_3$): δ 7.56 – 7.52 (m, 2H), 7.35 – 7.30 (m, 2H), 7.29 – 7.23 (m, 6H), 7.06 – 6.98 (s, 1H), 4.40 (dd, *J* = 5.9, 1.6 Hz, 2H), 4.19 (dd, *J* = 8.4, 3.9 Hz, 1H), 3.52 (dd, *J* = 13.2, 3.9 Hz, 1H), 3.33 – 3.20 (m, 1H), 3.13 (dd, *J* = 13.2, 8.4 Hz, 1H),

^{13}C NMR (125 MHz, $CDCl_3$): δ 171.6, 137.8, 133.3, 129.6, 128.9, 128.1, 127.9, 127.8, 70.2, 43.4, 34.0

LC-HRMS (ESI) *m/z* calcd. for $C_{16}H_{17}NNaO_2Se^+$ $[MH]^+$: 336.0497, found: 336.0536

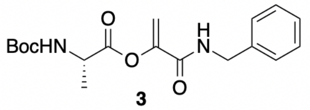

3

Chemical Formula: $C_{18}H_{24}N_2O_5$
Exact Mass: 348.1685

3-(benzylamino)-3-oxoprop-1-en-2-yl (*tert*-butoxycarbonyl)-*L*-alaninate (3)

NMI (179 µL, 2.2 mmol) was added to a solution of **S5** (150 mg, 0.5 mmol) and Boc-Ala-OH (212 mg, 1.1 mmol) in DCM (2 mL) at room temperature. The reaction was stirred until completion as monitored by TLC. The crude reaction mixture was then concentrated under reduced pressure and the resulting residue was purified by flash column chromatography on silica gel (0-100% EtOAc/Hexanes) to afford a mixture of intermediate **2** and Boc-Ala. The resulting mixture was then dissolved in MeOH (3 mL). Hydrogen peroxide (395 µL of 50% solution, 6.7 mmol) was then added with stirring. Upon completion as determined by LC-MS, the mixture was purified by RP-HPLC (5-95% acetonitrile/water with 0.1% TFA over 30 min) to yield the DHL-containing peptide **3** as a white solid (76 mg, 51% over 2 steps).

^{1}H NMR (500 MHz, $CDCl_3$): δ 7.73 (s, 1H), 7.30 (d, *J* = 4.4 Hz, 4H), 7.23 (q, *J* = 4.4, 1H), 6.21 (s, 1H), 5.31 (s, 1H), 5.01 (d, *J* = 5.7 Hz, 1H), 4.56 (dd, *J* = 14.9, 6.3 Hz, 1H), 4.45 (dd, *J* = 14.9, 5.7 Hz, 1H), 4.23 (qd, *J* = 7.2, 5.7 Hz, 1H), 1.46 (d, *J* = 7.2 Hz, 3H), 1.31 (s, 9H)

^{13}C NMR (125 MHz, $CDCl_3$): δ 171.4, 161.1, 156.3, 146.8, 138.0, 128.7, 127.9, 127.4, 111.7, 81.2, 50.0, 43.7, 28.3, 17.0

LC-HRMS (ESI) *m/z* calcd. for $C_{18}H_{24}N_2NaO_5^+$ $[MNa]^+$: 371.1577, found: 371.1585

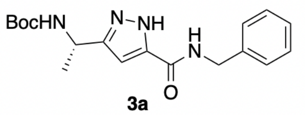

3a

Chemical Formula: $C_{18}H_{24}N_4O_3$
Exact Mass: 344.1848

tert-butyl (*S*)-(1-(5-(benzylcarbamoyl)-1*H*-pyrazol-3-yl)ethyl)carbamate (3a)

To a solution of DHL-peptide **3** (10 mg, 0.03 mmol) was added 50 mM NaPi pH 7 (850 µL) and MeOH (575 µL), which was incubated with stirring at room temperature for 1 h. Then, hydrazine (21 µL, 0.4 mmol) in 50 mM NaPi pH 5 (850 µL) was added to the mixture and stirred for 1 h. The solution was purified by RP-HPLC (5-95% acetonitrile/water with 0.1% TFA over 30 min) to yield the pyrazole-containing peptide **3a** as a white solid (7.3 mg, 74%).

^{1}H NMR (500 MHz, $CDCl_3$): δ 7.34 – 7.31 (m, 4H), 7.29 – 7.26 (m, 1H), 6.69 (s, 1H), 4.89 (s, 1H), 4.81 (p, *J* = 6.9 Hz, 1H), 4.65 – 4.58 (m, 2H), 1.56 (d, *J* = 7.0 Hz, 3H), 1.44 (s, 9H);

^{13}C NMR (125 MHz, $CDCl_3$): δ 162.4, 156.8, 148.7, 145.9, 138.1, 128.9, 127.9, 127.7, 102.9, 80.9, 43.5, 42.1, 28.5, 18.5;

LC-HRMS (ESI) m/z calcd. For $C_{18}H_{25}N_4O_3^+$ $[MH]^+$: 345.1921, found: 345.1918

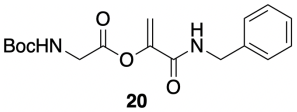

20

Chemical Formula: $C_{17}H_{22}N_2O_5$
Exact Mass: 334.1529

3-(benzylamino)-3-oxoprop-1-en-2-yl (*tert*-butoxycarbonyl)glycinate (20)

NMI (83 µL, 1 mmol) was added to a solution of **S5** (79 mg, 0.2 mmol) and Boc-Gly-OH (91 mg, 0.5 mmol) in DCM (2 mL) at room temperature. The reaction was stirred until completion as monitored by TLC. The crude reaction mixture was then concentrated under reduced pressure and the resulting residue was purified by flash column chromatography on silica gel (0-100% EtOAc/Hexanes) to afford a mixture of intermediate **13** and Boc-Gly. The resulting mixture was then dissolved in MeOH (3 mL). Hydrogen peroxide (152 µL of 50% solution, 3 mmol) was then added with stirring. Upon completion as determined by LC-MS, the mixture was purified by RP-HPLC (5-95% acetonitrile/water with 0.1% TFA over 30 min) to yield DHL-peptide **20** as a white solid (24 mg, 42% over 2 steps).

^{1}H NMR (500 MHz, $CDCl_3$): δ 7.47 (s, 1H), 7.34 – 7.23 (m, 5H), 6.19 (s, 1H), 5.37 (s, 1H), 5.18 (d, *J* = 5.9 Hz, 1H), 4.51 (d, *J* = 5.9 Hz, 2H), 3.96 (d, *J* = 5.8 Hz, 2H), 1.35 (s, 9H)

^{13}C NMR (125 MHz, $CDCl_3$): δ 168.0, 161.2, 156.9, 146.5, 137.8, 128.7, 128.0, 127.6, 111.7, 81.3, 43.8, 43.2, 28.3

LC-HRMS (ESI) *m/z* calcd. for $C_{17}H_{22}N_2NaO_5^+$ $[MNa]^+$: 357.1421, found: 357.1421

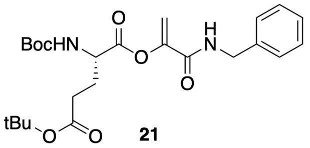

Chemical Formula: $C_{24}H_{34}N_2O_7$
Exact Mass: 462.2366

1-(3-(benzylamino)-3-oxoprop-1-en-2-yl) 5-(*tert*-butyl) (*tert*-butoxycarbonyl)-*L*-glutamate (21)

NMI (60 µL, 0.75 mmol) was added to a solution of **S5** (50 mg, 0.15 mmol) and Boc-Glu(OtBu)-OH (113 mg, 0.4 mmol) in DCM (2 mL) at room temperature. The reaction was stirred until completion as monitored by TLC. The crude reaction mixture was then concentrated under reduced pressure and the resulting residue was purified by flash column chromatography on silica gel (0-100% EtOAc/Hexanes) to afford a mixture of intermediate **14** and Boc-Glu(OtBu). The resulting mixture was then dissolved in MeOH (3 mL). Hydrogen peroxide (105 µL of 50% solution, 1.8 mmol) was then added with stirring. Upon completion as determined by LC-MS, the mixture was purified by RP-HPLC (5-95% acetonitrile/water with 0.1% TFA over 30 min) to yield DHL-peptide **21** as a white solid (19 mg, 34% over 2 steps).

^{1}H NMR (500 MHz, CDCl$_3$): δ 7.88 (s, 1H), 7.29 (d, *J* = 3.8 Hz, 4H), 7.25 – 7.20 (m, 1H), 6.23 (s, 1H), 5.44 (d, *J* = 5.9 Hz, 1H), 5.36 (s, 1H), 4.56 (dd, *J* = 14.9, 6.3 Hz, 1H), 4.46 (dd, *J* = 14.9, 5.8 Hz, 1H), 4.20 (dt, *J* = 8.2, 5.8 Hz, 1H), 2.46 – 2.34 (m, 2H), 2.18 – 2.04 (m, 2H), 1.44 (s, 9H), 1.31 (s, 9H)

^{13}C NMR (125 MHz, CDCl$_3$): δ 172.4, 170.5, 161.3, 156.6, 146.6, 137.9, 128.7, 127.9, 127.4, 112.2, 81.7, 81.1, 54.2, 43.8, 31.7, 28.3, 28.2, 25.9

LC-HRMS (ESI) *m/z* calcd. for $C_{24}H_{34}N_2NaO_7^+$ $[MNa]^+$: 485.2258, found: 485.2261

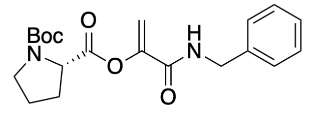

22

Chemical Formula: $C_{20}H_{26}N_2O_5$
Exact Mass: 374.1842

2-(3-(benzylamino)-3-oxoprop-1-en-2-yl) 1-(*tert*-butyl) (*S*)-pyrrolidine-1,2-dicarboxylate (22)

NMI (60 µL, 0.75 mmol) was added to a solution of **S5** (50 mg, 0.15 mmol) and Boc-Pro-OH (80 mg, 0.4 mmol) in DCM (2 mL) at room temperature. The reaction was stirred until completion as monitored by TLC. The crude reaction mixture was then concentrated under reduced pressure and the resulting residue was purified by flash column chromatography on silica gel (0-100% EtOAc/Hexanes) to afford a mixture of intermediate **15** and Boc-Pro. The resulting mixture was then dissolved in MeOH (3 mL). Hydrogen peroxide (145 µL of 50% solution, 2.5 mmol) was then added with stirring. Upon completion as determined by LC-MS, the mixture was purified by RP-HPLC (5-95% acetonitrile/water with 0.1% TFA over 30 min) to yield the DHL-peptide **22** as a white solid (30 mg, 49 % over 2 steps).

^{1}H NMR (500 MHz, CDCl$_3$): δ 8.24 (s, 1H), 7.30 – 7.27 (m, 4H), 7.24 – 7.19 (m, 1H), 6.24 (s, 1H), 5.27 (s, 1H), 4.57 (dd, *J* = 15.0, 6.4 Hz, 1H), 4.44 (dd, *J* = 15.0, 5.8 Hz, 1H), 4.35 (dd, *J* = 8.2, 5.4 Hz, 1H), 3.54 – 3.42 (m, 2H), 2.33 – 2.24 (m, 1H), 2.14 – 2.01 (m, 2H), 1.98 – 1.88 (m, 1H), 1.31 (s, 9H)
^{13}C NMR (125 MHz, CDCl$_3$): δ 171.3, 161.3, 155.5, 146.9, 138.1, 128.6, 127.8, 127.3, 112.3, 81.1, 59.1, 47.0, 43.7, 30.2, 28.4, 24.9
LC-HRMS (ESI) *m/z* calcd. for $C_{20}H_{26}N_2NaO_5^+$ $[MNa]^+$: 397.1734, found: 397.1741

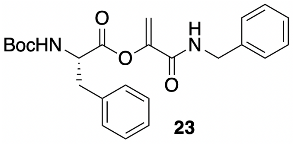

23

Chemical Formula: $C_{24}H_{28}N_2O_5$
Exact Mass: 424.1998

3-(benzylamino)-3-oxoprop-1-en-2-yl (*tert*-butoxycarbonyl)-*L*-phenylalaninate (23)

NMI (60 µL, 0.75 mmol) was added to a solution of **S5** (50 mg, 0.15 mmol) and Boc-Phe-OH (99 mg, 0.4 mmol) in DCM (2 mL) at room temperature. The reaction was stirred until completion as monitored by TLC. The crude reaction mixture was then concentrated under reduced pressure and the resulting residue was purified by flash column chromatography on silica gel (0-100% EtOAc/Hexanes) to afford a mixture of intermediate **16** and Boc-Phe. The resulting mixture was then dissolved in MeOH (3 mL). Hydrogen peroxide (136 µL of 50% solution, 2.3 mmol) was then added with stirring. Upon completion as determined by LC-MS, the mixture was purified by RP-HPLC (5-95% acetonitrile/water with 0.1% TFA over 30 min) to yield DHL-peptide **23** as a white solid (29 mg, 45% over 2 steps).
^{1}H NMR (500 MHz, CDCl$_3$): δ 7.59 (s, 1H), 7.36 – 7.19 (m, 10H), 6.14 (s, 1H), 5.00 (s, 1H), 4.98 (dd, *J* = 5.5 Hz, 1H), 4.53 (dd, *J* = 4.5 Hz, 1H), 4.44 – 4.39 (m, 1H), 3.11 (d, *J* = 7.4 Hz, 2H), 1.29 (s, 9H)
^{13}C NMR (125 MHz, CDCl$_3$): δ 170.6, 160.9, 156.3, 146.7, 138.1, 135.2, 129.3, 129.1, 128.6, 127.8, 127.3, 111.9, 81.4, 55.7, 43.6, 37.3, 28.3
LC-HRMS (ESI) m/z calcd. for $C_{24}H_{28}N_2NaO_5^+$ $[MNa]^+$: 447.1890, found: 447.1902

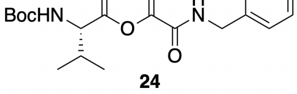

24

Chemical Formula: $C_{20}H_{28}N_2O_5$
Exact Mass: 376.1998

3-(benzylamino)-3-oxoprop-1-en-2-yl (*tert*-butoxycarbonyl)-*L*-valinate (24)

NMI (60 µL, 0.75 mmol) was added to a solution of **S5** (50 mg, 0.15 mmol) and Boc-Val-OH (81 mg, 0.4 mmol) in DCM (2 mL) at room temperature. The reaction was stirred until completion as monitored by TLC. The crude reaction mixture was then concentrated under reduced pressure and the resulting residue was purified by flash column chromatography on silica gel (0-100% EtOAc/Hexanes) to afford a mixture of intermediate **17** and Boc-Val. The resulting mixture was then dissolved in MeOH (3 mL). Hydrogen peroxide (59 µL of 50% solution, 1.2 mmol) was then

added with stirring. Upon completion as determined by LC-MS, the mixture was purified by RP-HPLC (5-95% acetonitrile/water with 0.1% TFA over 30 min) to yield DHL-peptide **24** as a white solid (32 mg, 59 % over 2 steps).

^{1}H NMR (500 MHz, CDCl$_3$): δ 7.66 (s, 1H), 7.31 – 7.28 (m, 4H), 7.26 – 7.21 (m, 1H), 6.21 (s, 1H), 5.26 (s, 1H), 4.97 (d, *J* = 6.3 Hz, 1H), 4.59 (dd, *J* = 14.9, 6.5 Hz, 1H), 4.42 (dd, *J* = 15.0, 5.6 Hz, 1H), 4.01 (t, *J* = 6.3 Hz, 1H), 2.19 – 2.09 (m, 1H), 1.32 (s, 9H), 1.05 (dd, *J* = 11.6, 6.8 Hz, 6H)

^{13}C NMR (125 MHz, CDCl$_3$): δ 170.8, 161.0, 156.7, 146.9, 138.1, 128.6, 127.8, 127.4, 111.7, 81.1, 60.0, 43.7, 30.3, 28.3, 19.2, 18.7

LC-HRMS (ESI) *m/z* calcd. for $C_{20}H_{28}N_2NaO_5^+$ [MNa]$^+$: 399.1890, found: 399.1899

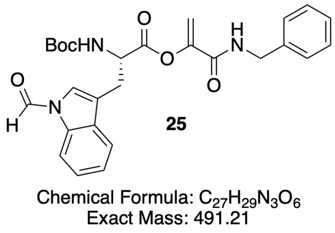

3-(benzylamino)-3-oxoprop-1-en-2-yl *Nα*-(*tert*-butoxycarbonyl)-1-formyl-*L*-tryptophanate (25)

NMI (60 µL, 0.75 mmol) was added to a solution of **S5** (50 mg, 0.15 mmol) and Boc-Trp(For)-OH (124 mg, 0.4 mmol) in DCM (2 mL) at room temperature. The reaction was stirred until completion as monitored by TLC. The crude reaction mixture was then concentrated under reduced pressure and the resulting residue was purified by flash column chromatography on silica gel (0-100% EtOAc/Hexanes) to afford a mixture of intermediate **18** and Boc-Trp(For)-OH. The resulting mixture was then dissolved in MeOH (3 mL). Hydrogen peroxide (59 µL of 50% solution, 1.2 mmol) was then added with stirring. Upon completion as determined by LC-MS, the mixture was purified by RP-HPLC (5-95% acetonitrile/water with 0.1% TFA over 30 min) to yield DHL-peptide **25** as a white solid (27 mg, 37 % over 2 steps).

^{1}H NMR (500 MHz, CDCl$_3$): δ 9.35 (s, 0.4H), 9.02 (s, 0.6H), 8.52 – 8.25 (m, 1H), 7.76 – 7.29 (m, 5H), 7.29 – 7.16 (m, 5H), 6.06 (s, 1H), 5.03 (d, *J* = 6.0 Hz, 1H), 4.96 (s, 1H), 4.51 – 4.45 (m, 2H), 4.42–4.33 (m, 1H), 3.26 (qd, *J* = 14.8, 6.5 Hz, 2H), 1.27 (s, 9H).

^{13}C NMR (125 MHz, CDCl$_3$): δ 170.35, 160.90, 159.17, 156.24, 155.84, 146.83, 138.00, 128.68, 127.83, 127.44, 126.11, 125.07, 123.81, 119.09, 116.55, 111.52, 81.52, 54.02, 43.66, 28.33, 28.26, 26.96.

LC-HRMS (ESI) *m/z* calcd. for $C_{27}H_{29}N_3NaO_6^+$ [MNa]$^+$: 514.1949, found: 514.1949

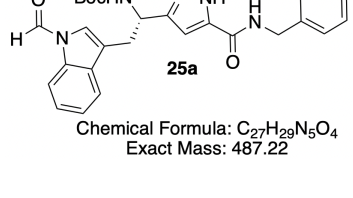

***tert*-butyl-(*S*)-(1-(5-(benzylcarbamoyl)-1*H*-pyrazol-3-yl)-2-(1-formyl-1*H*-indol-3-yl)ethyl)carbamate (25a)**

To a solution of DHL-peptide **25** (17 mg, 0.03 mmol) was added 50 mM NaPi pH 7 (285 µL) and MeOH (285 µL), which was incubated with stirring at room temperature for 1 h. Then, hydrazine (10 µL, 0.2 mmol) in 50 mM NaPi pH 5 (427 µL) was added to the mixture and stirred for 1 h. The solution was purified by RP-HPLC (5-95% acetonitrile/water with 0.1% TFA over 30 min) to yield the pyrazole-containing peptide **25a** as a white solid (3.5 mg, 50%).

^{1}H NMR (500 MHz, CDCl$_3$): δ 9.35 (s, 0.4H), 9.00 (s, 0.6H), 8.40 – 8.31 (m, 1H), 7.73 – 7.47 (m, 2H), 7.43 – 7.29 (m, 7H), 7.11 (s, 2H), 6.72 (s, 1H), 5.25 – 4.97 (m, 2H), 4.59 (d, *J* = 5.8 Hz, 2H), 3.40 – 3.20 (m, 2H), 1.41 (d, Hz, 9H).

^{13}C NMR (125 MHz, CDCl$_3$): δ 161.66, 159.27, 156.38, 155.78, 147.55, 145.30, 137.97, 135.52, 134.49, 128.89, 127.97, 127.76, 125.77, 125.31, 124.93, 123.60, 120.18, 119.92, 118.91, 116.46, 109.90, 103.32, 81.14, 52.65, 46.79, 43.48, 29.68, 28.39.

LC-HRMS (ESI) *m/z* calcd. for $C_{27}H_{30}N_5O_4^+$ [MH]$^+$: 488.2292, found: 488.2132

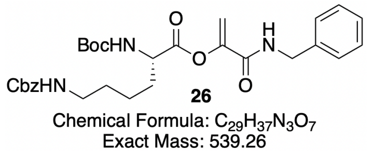

3-(benzylamino)-3-oxoprop-1-en-2-yl-*N*6-((benzyloxy)carbonyl)-*N*2-(*tert*-butoxycarbonyl)-*L*-lysinate (26)

NMI (60 µL, 0.75 mmol) was added to a solution of **S5** (50 mg, 0.15 mmol) and Boc-Lys(Cbz)-OH (142 mg, 0.4 mmol) in DCM (2 mL) at room temperature. The reaction was stirred until completion as monitored by TLC. The crude reaction mixture was then concentrated under reduced pressure and the resulting residue was purified by flash column chromatography on silica gel (0-100% EtOAc/Hexanes) to afford a mixture of intermediate **19** and Boc-Lys(Cbz)-OH. The resulting mixture was then dissolved in MeOH (3 mL). Hydrogen peroxide (59 µL of 50% solution, 1.2 mmol) was then added with stirring. Upon completion as determined by LC-MS, the mixture was purified by RP-HPLC (5-95% acetonitrile/water with 0.1% TFA over 30 min) to yield DHL-peptide **26** as a white solid (49 mg, 61 % over 2 steps).

^{1}H NMR (500 MHz, CDCl$_3$): δ 7.75 (s, 1H), 7.36–7.33 (m, 4H), 7.31 (s, 1H), 7.27–7.30 (m, 4H), 7.21–7.25 (m, 1H), 6.17 (s, 1H), 5.40 (d, *J* = 5.9 Hz, 1H), 5.29 (s, 1H), 5.12 – 5.07 (m, 2H), 4.93–4.86 (m, 1H), 4.55 (dd, *J* = 15.0, 6.2 Hz, 1H), 4.44 (dd, *J* = 14.9, 5.7 Hz, 1H), 4.15 (dt, *J* = 11.5, 5.6 Hz, 1H), 3.28–3.14 (m, 2H), 1.92 – 1.76 (m, 2H), 1.58–1.40 (m, 6H), 1.31 (s, 9H).

^{13}C NMR (125 MHz, CDCl$_3$): δ 171.08, 161.21, 157.12, 156.71, 146.85, 138.01, 136.50, 128.71, 128.65, 128.37, 128.30, 127.88, 127.40, 111.77, 81.00, 66.99, 54.25, 43.71, 39.88, 30.29, 29.65, 28.30, 27.72, 22.24.

LC-HRMS (ESI) *m/z* calcd. for $C_{29}H_{37}N_3NaO_7^+$ [MNa]$^+$: 562.2524, found: 562.2525

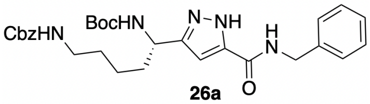

26a

Chemical Formula: $C_{29}H_{37}N_5O_5$
Exact Mass: 535.28

benzyl *tert*-butyl (1-(5-(benzylcarbamoyl)-1*H*-pyrazol-3-yl)pentane-1,5-diyl)(*S*)-dicarbamate (26a)

To a solution of DHL-peptide **26** (17 mg, 0.03 mmol) was added 50 mM NaPi pH 7 (630 µL) and MeOH (630 µL), which was incubated with stirring at room temperature for 1 h. Then, hydrazine (23 µL, 0.5 mmol) in 50 mM NaPi pH 5 (945 µL) was added to the mixture and stirred for 1 h. The solution was purified by RP-HPLC (5-95% acetonitrile/water with 0.1% TFA over 30 min) to yield the pyrazole-containing peptide **26a** as a white solid (3.5 mg, 21%).

^{1}H NMR (500 MHz, CDCl$_3$): δ 7.37 – 7.31 (m, 10H), 7.29 – 7.26 (d, *J* = 4.2 Hz, 1H), 6.66 (s, 1H), 5.16 – 5.11 (m, 1H), 5.10 (d, *J* = 3.8 Hz, 2H), 4.85 (br-s, 1H), 4.69 – 4.63 (m, 1H), 4.61 (d, *J* = 5.7 Hz, 2H), 3.29 – 3.12 (m, 2H), 1.98 – 1.81 (m, 2H), 1.60 – 1.48 (m, 2H), 1.47 – 1.36 (m, 11H).

^{13}C NMR (125 MHz, CDCl$_3$): δ 168.42, 162.24, 157.04, 156.77, 147.96, 145.81, 138.07, 136.54, 128.86, 128.70, 128.35, 128.29, 127.97, 127.68, 103.06, 80.64, 67.02, 46.72, 43.45, 40.28, 32.34, 29.70, 28.46, 22.84.

LC-HRMS (ESI) *m/z* calcd. for $C_{29}H_{38}N_5O_5^+$ [MH]$^+$: 536.2867, found: 536.2808

Ester block synthesis

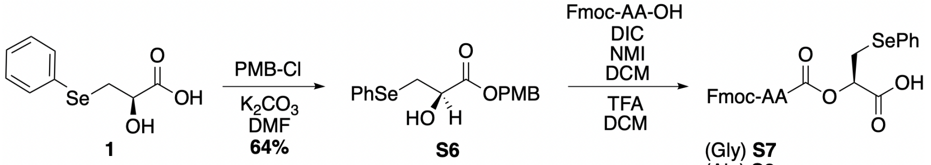

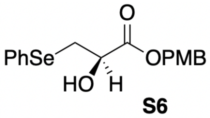

S6

Chemical Formula: $C_{17}H_{18}O_4Se$
Exact Mass: 366.0370

4-methoxybenzyl (*R*)-2-hydroxy-3-(phenylselanyl)propanoate (S6)

To a solution of carboxylic acid **1** (2g, 8.2 mmol) in DMF (10 mL) was added K_2CO_3 (1.7 g, 12.2 mmol) and PMB-Cl (1.7 mL, 12.2 mmol) with stirring. The reaction was heated to 50 °C and determined to be complete after 4 hours as determined by TLC. The mixture was quenched with sat. NH_4Cl (30 mL) and extracted with EtOAc (3 x 30 mL). Combined organic layers were dried over Na_2SO_4, concentrated under vacuum, and purified by flash column chromatography on silica gel (0-100% EtOAc/Hexanes) to afford the PMB ester **S6** as a white solid (1.9 g, 64%).

^{1}H NMR (500 MHz, CDCl$_3$): δ 7.57 – 7.52 (m, 2H), 7.27 – 7.24 (m, 3H), 7.22 – 7.18 (m, 2H), 6.89 – 6.84 (m, 2H), 5.06 (d, *J* = 11.8 Hz, 1H), 4.83 (d, *J* = 11.8 Hz, 1H), 4.49 (q, *J* = 5.0 Hz, 1H), 3.81 (s, 3H), 3.35 (dd, *J* = 13.0, 4.4 Hz, 1H), 3.23 (dd, *J* = 13.0, 5.3 Hz, 1H), 3.16 (d, *J* = 6.1 Hz, 1H);
^{13}C NMR (125 MHz, CDCl$_3$): δ 173.0, 160.0, 133.6, 130.5, 129.5, 129.3, 127.55, 127.1, 114.1, 69.8, 67.6, 55.4, 32.8;
LC-HRMS (ESI) m/z calcd. For $C_{17}H_{18}NaO_4Se^+$ [MNa]$^+$: 389.0263, found: 389.0296

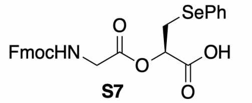

S7

Chemical Formula: $C_{26}H_{23}NO_6Se$
Exact Mass: 525.0691

(*R*)-2-((((9*H*-fluoren-9-yl)methoxy)carbonyl)glycyl)oxy)-3-(phenylselanyl)propanoic acid (S7)

To a solution of **S6** (630 mg, 1.4 mmol) in DCM (2 mL) was added Fmoc-Gly-OH (2.1 g, 6.9 mmol), NMI (775 µL, 9.7 mmol), and DIC (1.1 mL, 6.9 mmol) with stirring and monitored by TLC. Upon completion, the solution was purified directly by flash column chromatography on silica gel (0-100% EtOAc/Hexanes) to afford a mixture of PMB-protected ester block and Fmoc-Gly. To the isolated residue in DCM (10 mL) was added trifluoroacetic acid (1.3 mL, 16.7 mmol) with stirring. The reaction was monitored by TLC and concentration under vacuum upon completion. The resulting residue was purified directly by flash column chromatography on silica gel (0-10% MeOH/DCM 1% AcOH) to afford a white solid (710 mg, 97%).
^{1}H NMR (500 MHz, CDCl$_3$): δ 7.75 (d, *J* = 7.5 Hz, 2H), 7.56 (d, *J* = 7.5 Hz, 2H), 7.52 – 7.49 (m, 2H), 7.39 (t, *J* = 7.5 Hz, 2H), 7.29 (t, *J* = 7.4 Hz, 2H), 7.25 – 7.21 (m, 3H), 5.37 (dd, *J* = 8.2, 3.8 Hz, 1H), 5.17 (t, *J* = 5.9 Hz, 1H), 4.44 – 4.36 (m, 2H), 4.19 (t, *J* = 6.7 Hz, 1H), 3.84 (qd, *J* = 18.2, 5.9 Hz, 2H), 3.35 (dd, *J* = 13.6, 3.9 Hz, 1H), 3.22 (dd, *J* = 13.6 Hz, 8.2 Hz, 1H);
^{13}C NMR (125 MHz, CDCl$_3$): δ 171.5, 169.5, 156.9, 143.8, 141.5, 133.8, 129.4, 129.1, 127.9, 127.3, 125.2, 120.2, 73.0, 67.4, 47.2, 42.5, 27.5;
LC-HRMS (ESI) m/z calcd. For $C_{26}H_{23}NNaO_6Se^+$ [MNa]$^+$: 548.0583, found: 548.0536

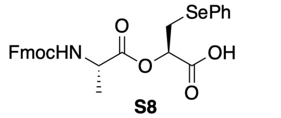

S8

Chemical Formula: $C_{27}H_{25}NO_6Se$
Exact Mass: 539.0847

***(R)*-2-((((9*H*-fluoren-9-yl)methoxy)carbonyl)-*L*-alanyl)oxy)-3-(phenylselanyl)propanoic acid (S8)**

To a solution of **S6** (1.1 g, 2.2 mmol) in DCM (2 mL) was added Fmoc-Ala-OH (3.5 g, 11.2 mmol), NMI (1.3 mL, 15.7 mmol), and DIC (1.8 mL, 11.2 mmol) with stirring and monitored by TLC. Upon completion, the solution was purified directly by flash column chromatography on silica gel (0-100% EtOAc/Hexanes) to afford a mixture of PMB-protected ester block and Fmoc-Ala. To this residue was added DCM (10 mL) and trifluoroacetic acid (1.6 mL, 20.9 mmol) with stirring.

The reaction was monitored by TLC and concentration under vacuum upon completion. The resulting residue was purified directly by flash column chromatography on silica gel (0-10% MeOH/DCM 1% AcOH) to afford a white solid (1.0 g, 84%).

^{1}H NMR (500 MHz, CDCl$_3$): δ 7.77 (d, *J* = 7.7 Hz, 2H), 7.60 (t, *J* = 7.0 Hz, 2H), 7.54–7.49 (m, 2H), 7.40 (t, *J* = 7.6 Hz, 2H), 7.32 (t, *J* = 7.4 Hz, 2H), 7.24 (d, *J* = 7.0 Hz, 3H), 5.48 – 5.42 (m, 1H), 5.06 (d, *J* = 7.8 Hz, 1H), 4.55 – 4.41 (m, 2H), 4.33 – 4.28 (m, 1H), 4.30 (t, *J* = 6.9 Hz, 1H), 3.38 (dd, 13.5, 4.0 Hz, 1H), 3.26 (dd, *J* = 13.5, 7.7 Hz, 1H), 1.36 (d, *J* = 7.2 Hz, 3H);

^{13}C NMR (125 MHz, CDCl$_3$): δ 172.0, 156.1, 143.9, 143.8, 141.5, 133.8, 129.4, 129.0, 128.0, 127.3, 125.2, 125.1, 120.2, 72.5, 67.2, 49.6, 47.2, 27.5, 18.3, 2.0;

LC-HRMS (ESI) m/z calcd. for $C_{27}H_{25}NNaO_6Se^+$ [MNa]$^+$: 562.0739, found: 562.0749

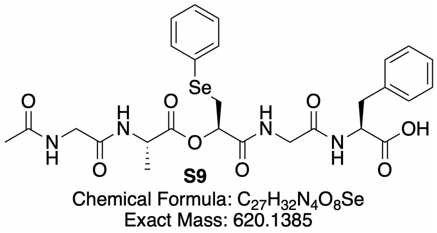

Seleno-peptide S9

Peptide **S9** was synthesized according to the general peptide synthesis methods section below. Cleavage of 0.04 mmol peptidyl resin, followed by RP-HPLC purification according to described methods, yielded pure peptide **S9** as a white solid (3.1 mg, 14.3% from resin).

^{1}H NMR (500 MHz, DMSO) δ 8.36 (d, *J* = 7.2 Hz, 1H), 8.32 (t, *J* = 5.9 Hz, 1H), 8.12 – 8.04 (m, 2H), 7.55 – 7.48 (m, 2H), 7.35 – 7.12 (m, 9H), 6.54 (s, 1H), 5.24 (dd, *J* = 8.2, 4.3 Hz, 1H), 4.43 (td, *J* = 8.3, 5.0 Hz, 1H), 4.26 (p, *J* = 7.1 Hz, 1H), 3.87 (dd, *J* = 17.7, 5.9 Hz, 1H), 3.77 – 3.53 (m, 3H), 3.22 (dd, *J* = 13.0, 8.2 Hz, 1H), 3.04 (dd, *J* = 13.8, 5.1 Hz, 1H), 2.88 (dd, *J* = 13.8, 8.9 Hz, 1H), 2.08 (s, 2H), 1.86 (s, 3H), 1.22 (d, *J* = 7.0 Hz, 3H).

^{13}C NMR (126 MHz, DMSO) δ 173.22, 172.52, 170.76, 169.85, 169.21, 167.95, 137.62, 132.41, 130.35, 129.71, 129.58, 128.68, 127.43, 126.94, 73.52, 53.95, 48.95, 42.07, 40.87, 40.57, 40.41, 40.24, 40.07, 37.27, 31.17, 28.43, 22.69, 18.37.

LC-HRMS (ESI) m/z calcd. for $C_{27}H_{33}N_4O_8Se^+$ [MH]$^+$: 621.1458, found: 621.1451

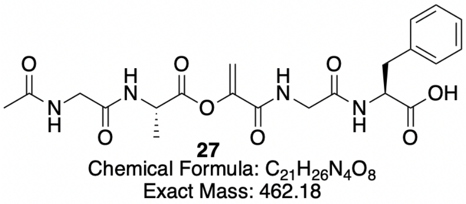

DHL Peptide 27

Seleno-peptide **S9** (5 mM) was stirred in 20 mM H_2O_2 in MeCN until complete DHL formation was observed as determined by LC-MS, the mixture was purified by RP-HPLC (5-95% acetonitrile/water with 0.1% TFA over 30 min) to yield DHL-peptide **27** as a white solid (1.1 mg, 47%).

^{1}H NMR (500 MHz, DMSO) δ 12.75 (s, 1H), 8.49 (t, *J* = 5.8 Hz, 1H), 8.24 (d, *J* = 7.2 Hz, 1H), 8.13 (t, *J* = 5.8 Hz, 1H), 8.08 (d, *J* = 8.0 Hz, 1H), 7.30 – 7.18 (m, 5H), 5.94 (d, *J* = 2.1 Hz, 1H), 5.37 (d, *J* = 2.1 Hz, 1H), 4.42 (td, *J* = 8.4, 5.1 Hz, 1H), 4.31 (p, *J* = 7.0 Hz, 1H), 4.04 (dd, *J* = 5.7, 2.8 Hz, 1H), 3.68 (qd, *J* = 16.7, 5.8 Hz, 2H), 3.04 (dd, *J* = 13.8, 5.1 Hz, 1H), 2.87 (dd, *J* = 13.8, 8.9 Hz, 1H), 2.09 (s, 2H), 1.89 (s, 3H), 1.26 (d, *J* = 7.2 Hz, 3H).
^{13}C NMR (126 MHz, DMSO) δ 173.20, 172.51, 170.90, 169.13, 168.74, 160.95, 146.99, 137.89, 130.06, 128.68, 127.13, 110.81, 53.97, 50.26, 42.50, 41.41, 37.28, 31.55, 23.50, 18.08.
LC-HRMS (ESI) m/z calcd. for $C_{21}H_{26}N_4O_8^+$ [MH]$^+$: 463.1823, found: 463.1818

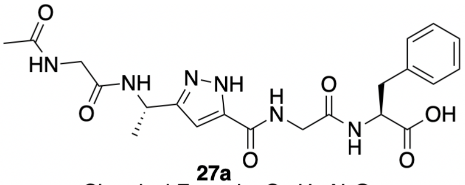

27a
Chemical Formula: $C_{21}H_{26}N_6O_6$
Exact Mass: 458.19

Pyrazole Peptide 27a
Seleno-peptide **S9** (13 mg, 20 mM) was stirred in 100 mM H_2O_2 in MeCN until complete DHL formation was observed as determined by LC-MS. Solvent was removed via lyophilization, and the peptide was resuspended in 50 mM NaPi pH 9 (20 mM) and stirred for 3 h. Then, hydrazine (5 µL, 0.15 mmol) in 50 mM NaPi pH 5 (1884 µL) was added to the mixture and stirred for 1 h. The solution was purified by RP-HPLC (5-95% acetonitrile/water with 0.1% TFA over 30 min) to yield the pyrazole-containing peptide **27a** as a white solid (1 mg, 7% over two steps).
^{1}H NMR (700 MHz, DMSO) δ 8.30 – 8.21 (m, 1H), 8.19 – 8.04 (m, 1H), 7.30 – 7.14 (m, 5H), 6.54 (s, 1H), 4.45 – 4.22 (m, 2H), 3.79 – 3.69 (m, 1H), 3.67 – 3.58 (m, 1H), 3.06–3.00 (m, 1H), 2.90–2.84 (m, 1H), 1.89 (s, 1.5H), 1.76 (s, 1.5H), 1.29 (d, *J* = 7.0 Hz, 1H), 1.22–1.19 (m, 2H).
LC-HRMS (ESI) m/z calcd. for $C_{21}H_{27}N_6O_6^+$ [MH]$^+$: 459.1987 found: 459.1993

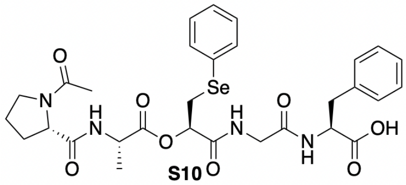

S10
Chemical Formula: $C_{30}H_{36}N_4O_8Se$
Exact Mass: 660.17

Peptide **S10** was synthesized according to the general peptide synthesis methods section below. Cleavage of 0.04 mmol peptidyl resin, followed by RP-HPLC purification according to described methods, yielded pure peptide **S10** as a white solid (8.9 mg, 39% from resin).
^{1}H NMR (500 MHz, DMSO) δ 8.36 (d, *J* = 7.1 Hz, 1H), 8.19 – 8.01 (m, 2H), 7.58 – 7.47 (m, 2H), 7.35 – 7.17 (m, 8H), 5.22–5.15 (m, 1H), 4.43 (td, *J* = 8.4, 5.1 Hz, 1H), 4.32 – 4.18 (m, 1H), 3.77 – 3.69 (m, 1H), 3.68 – 3.60 (m, 1H), 3.26 – 3.18 (m, 1H), 3.08 – 3.01 (m, 1H), 2.91 – 2.84 (m, 1H), 2.09 (s, 1H), 1.99 (s, 1H), 1.96 (s, 1H), 1.93 (s, 1H), 1.23 – 1.17 (m, 3H).

^{13}C NMR (126 MHz, DMSO) δ 173.21, 172.46, 170.04, 169.00, 168.16, 167.99, 137.91, 132.39, 130.38, 129.73, 129.68, 129.66, 129.58, 128.67, 127.41, 127.38, 126.93, 73.31, 73.16, 58.49, 53.94, 48.93, 47.73, 42.08, 37.27, 31.17, 29.24, 28.47, 24.76, 22.52, 20.91, 18.38.
LC-HRMS (ESI) m/z calcd. for $C_{30}H_{37}N_4O_8Se^+$ $[MH]^+$: 661.1771, found: 661.1760

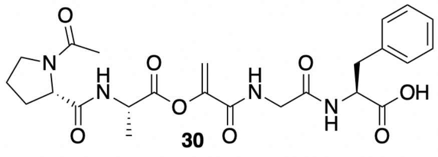

Peptide **S10** (5 mM) was stirred in 20 mM H_2O_2 in MeCN until complete DHL formation was observed as determined by LC-MS, the mixture was purified by RP-HPLC (5-95% acetonitrile/water with 0.1% TFA over 30 min) to yield DHL-peptide **30** as a white solid (3.9 mg, 76%).

^{1}H NMR (600 MHz, DMSO) δ 8.14 (t, *J* = 5.9 Hz, 1H), 8.10 (d, *J* = 6.8 Hz, 1H), 8.03 (dd, *J* = 10.5, 8.0 Hz, 1H), 7.28 – 7.24 (m, 2H), 7.22 – 7.17 (m, 3H), 5.95 (d, *J* = 2.1 Hz, 1H), 5.39 (d, *J* = 2.1 Hz, 1H), 4.46 – 4.39 (m, 2H), 4.34 – 4.25 (m, 1H), 3.74 – 3.49 (m, 4H), 3.04 (dd, *J* = 13.8, 5.1 Hz, 1H), 2.87 (dd, *J* = 13.9, 8.9, 1H), 2.32 – 2.17 (m, 2H), 2.12 – 2.06 (m, 5H), 2.01 (s, 3H), 1.99 – 1.89 (m, 2H), 1.25 (dd, *J* = 10.6, 7.1 Hz, 3H).
^{13}C NMR (126 MHz, DMSO) δ 168.16, 150.41, 149.96, 129.60, 128.49, 126.81, 123.64, 58.68, 58.56, 53.79, 53.06, 51.12, 49.43, 47.79, 37.57, 35.20, 31.47, 29.56, 29.43, 25.07, 22.79, 22.05, 18.43.
LC-HRMS (ESI) m/z calcd. for $C_{24}H_{31}N_4O_8^+$ $[MH]^+$: 503.2136, found: 503.2163

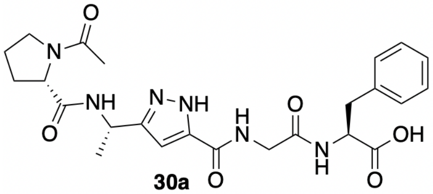

Pyrazole Peptide 30a
To a solution of DHL-peptide **30** (7 mg, 0.01 mmol) was added 50 mM NaPi pH 9 (562 µL) and MeCN (140 µL), which was incubated with stirring at room temperature for 12 h. Then, hydrazine (5 µL, 0.1 mmol) in 50 mM NaPi pH 5 (937 µL) was added to the mixture and stirred for 9 h. The solution was purified by RP-HPLC (5-95% acetonitrile/water with 0.1% TFA over 30 min) to yield the pyrazole-containing peptide **30a** as a white solid (2.3 mg, 36%).
^{1}H NMR (700 MHz, DMSO) δ 12.79 (s, 1H), 8.23 (s, 1H), 8.11 (d, *J* = 8.0 Hz, 0.6H), 8.09 (d, *J* = 8.0 Hz, 0.4H), 7.30 – 7.13 (m, 5H), 6.49 (s, 1H), 5.11 – 5.03 (m, 1H), 4.51 – 4.34 (m, 2H),

3.75–3.70 (m, 1H), 3.66–3.60 (m, 1H), 3.56–3.50 (m, 2H), 3.06–3.01 (m, 1H), 2.91–2.86 (m, 1H), 2.28–2.21 (m, 1H), 2.13–2.06 (m, 1H), 1.98 (s, 1.5H), 1.95 – 1.81 (m, 2H), 1.79 (s, 1.5H), 1.29 (d, J = 3.0 Hz, 1.5H), 1.28 (d, J = 3.0 Hz, 1.5H).
^{13}C NMR (126 MHz, DMSO) δ 172.73, 172.41, 172.38, 168.57, 168.46, 158.26, 158.00, 157.74, 137.44, 129.12, 128.19, 126.43, 102.79, 53.50, 48.25, 48.12, 46.94, 45.59, 41.73, 36.80, 34.02, 31.98, 23.72, 22.51, 22.23, 21.91, 18.36, 18.13.
LC-HRMS (ESI) m/z calcd. for $C_{24}H_{31}N_6O_6^+$ $[MH]^+$: 499.2300 found: 499.2305

Methods to Support Figure 2

Purified DHL-peptide **3** in MeOH (10 mM, 15 µL) were transferred to PCR tubes. The peptide was either diluted with 50 mM NaPi pH 8, or supplemented with 1 equivalent of tetramethyl guanidinium and analyzed after 4 and 16 hours, and 15 minutes, respectively. Each reaction was then analyzed on a Zorbax Eclipse Plus C18 column (1.8 µm, 1.2 x 50 mm, room temperature, Agilent) using a linear gradient from 5 to 95% acetonitrile over 7.5 min with 0.1% formic acid as the aqueous mobile phase after an initial hold at 95% 0.1% formic acid for 0.5 min (0.7 mL/min) using an 1290 Infinity II UHPLC (G7120AR, Agilent).

DHL-peptide **3** (3 mg) was analyzed by NMR in methanol-d_4 before and after addition of 50% 50 mM NaPi pH 8. 1H, Heteronuclear Multiple Bond Correlation (HMBC), and multiplicity-edited Heteronuclear Single Quantum Coherence (multiHSQC) spectroscopy were used in order to characterize the structure of isomer **3'**. Analysis was performed on a Bruker 700 MHz instrument.

DHL-peptide **3** (10 mg) was incubated in 50% MeOH 50 mM NaPi pH 7 for 1 hour before addition of 20 mM hydrazine in 50 mM NaPi pH 6 for 1 hour. Resulting pyrazole-peptide 3a was purified using RP-HPLC (5-95% acetonitrile/water with 0.1% TFA over 30 min) and characterized by NMR (see Synthetic Methods).

Computational methods

Methods to Support Figure 3

For each compound that was studied, molecular mechanics methods (Macromodel, OPLS4 force field) were used to minimize a starting population of 1,000 conformers that was initially generated using the Conformational Search tool in the Schrödinger Maestro 2024-1 environment. The geometries of all unique conformers that were found to be within 5 kcal/mol of the global minimum were then optimized again using DFT at the B3LYP-D3 / 6-31G** level. An implicit CPCM water solvation model was used in these calculations. All species resulting from this round of computation were subjected to a second geometry optimization and vibrational spectrum calculation in QChem v. 6.02 (B3LYP-D3 / 6-31G**) to determine the zero-point energies, enthalpy corrections, and the internal entropy values at 298.15 K. A CPCM model was used with Bondi van der Waals radii and a SAS solvent probe radius of 1.4 Å in these calculations. All non-transition state compounds were confirmed to have only positive vibrational frequencies. For all

species, refined electronic energy calculations were performed on the optimized geometries using an improved functional and an expanded basis set (ωB97M-V / def2-TZVPPD) in QChem v. 6.02, again using the CPCM implicit solvation model described above. For each intermediate, tabulated energy and entropy values for the minimum energy conformations appear in Figure S5. Atomic coordinates of the optimized structures are tabulated in an accompanying file.

pK_a Calculations

The pK_a values of key intermediates were estimated using the Jaguar pK_a Prediction Tool (Micro pK_a mode) in the Schrödinger Maestro 2024-1 environment. The "Thorough" method was used, with up to 5 conformers for each species.

Transition State Identification for the Cyclization of 7

Transition state candidates were identified using the Jaguar Transition State Search Tool in the Schrödinger Maestro 2024-1 environment. The Linear Synchronous Transit (LST) strategy was used with starting and product structures that were generated using the Builder tool. The lowest Hessian eigenvector search algorithm was applied using B3LYP-D4 / 6-31G** as the DFT method and an implicit CPCM water solvation model. The lowest energy transition state structure was subjected to a single-point frequency calculation using B3LYP-D4 / 6-31G** / CPCM and verified to have one and only one negative vibrational frequency. This structure was further verified using the Jaguar IRC Tool in the Schrödinger Maestro 2024-1 environment. Starting with the transition state candidate, 10 forward and 10 reverse points were calculated at 0.1 step intervals along the intrinsic reaction coordinate using B3LYP-D4 / 6-31G** / CPCM. Each of the structures in the resulting reaction coordinate series was then subjected to a single point calculation to obtain the molecular orbitals and vibrational frequencies using B3LYP-D4 / 6-31G** / CPCM. Finally, the electronic energies of the reaction coordinate series were determined using ωB97M-V / def2-TZVPPD with a CPCM implicit water solvation model using Orca 6.01. The relative enthalpy values along the reaction coordinate were calculated from the final electron energy values and the thermochemical data obtained at the B3LYP-D4 / 6-31G** / CPCM level. The distances between the atoms that are joined in the reaction step were calculated in Schrödinger Maestro 2024-1. Snapshots of the compound structures, HOMOs, and LUMOs were visualized in Schrödinger Maestro 2024-1 and combined into the Supplemental Movies using Quicktime.

General Peptide Synthesis & Labeling Methods

Methods to Support Figure 4

Purified DHL-containing peptides in MeOH (10 mM, 15 µL) were transferred to PCR tubes. Then, NaPi pH 7 (50 mM, 7.5 µL) was added to each tube and incubated for 1 h at room temperature. Then, solutions of hydrazine or O-methyl hydroxylamine (75 mM, 7.5 µL) dissolved in 50 mM NaPi pH 6 were added to each respective tube and incubated for 6 h. Each reaction was then

analyzed on a Zorbax Eclipse Plus C18 column (1.8 µm, 1.2 x 50 mm, room temperature, Agilent) using a linear gradient from 5 to 95% acetonitrile over 7.5 min with 0.1% formic acid as the aqueous mobile phase after an initial hold at 95% 0.1% formic acid for 0.5 min (0.7 mL/min) using an 1290 Infinity II UHPLC (G7120AR, Agilent).

Solid-phase peptide synthesis methods to support Figure 5

Peptides were synthesized on 2-chlorotrityl chloride resin (0.6 mmol/g) on a 0.1 mmol scale. The resin was first swelled with DCM (5 mL). The first Fmoc-amino acid (5 eq.) was coupled with DIPEA (7.5 eq.) in DCM (3 mL) for 30 min. Fmoc-amino acids (5 eq.) were coupled to the resin along with HATU (4.9 eq.), and DIPEA (7.5 eq.) in DMF (2 mL) for 30 min, followed by Fmoc deprotection with 20% piperidine in DMF (1 mL). Each ester block (1.1 eq.) was coupled in DCM along with DIC (1.1 eq.) and NHS (1.1 eq.) for 2 – 16 h. Peptidyl resins were cleaved in a solution of 97.5% TFA, 1.25% water, and 1.25% thioanisole to yield peptides with a C-terminal carboxylic acid. If no side chain protecting groups were present, the resin was cleaved with 5% TFA in DCM (3 mL). Peptides were purified by RP-HPLC (5-95% acetonitrile/water with 0.1% TFA over 30 min).

Methods for labeling SPPS-synthesized peptides (to support Figure 5)

Purified OH-SecPh-containing peptides in MeCN (5 mM) were transferred to PCR tubes. Then, H_2O_2 in MeCN (50 mM) was added to each tube. Oxidation and elimination were monitored by LC-HRMS. When elimination was completed, the tubes were lyophilized to remove excess H_2O_2. Then, peptides were resuspended in MeCN and diluted into 50 mM NaPi at the pH indicated (7–9) to 5 mM concentration. For time and pH-dependent experiments, isomerization was monitored by addition of hydrazine (20 mM) in 50 mM NaPi pH 6, incubated for 15 min and analyzed by LC-HRMS. Peptide **26** required 4 hours of isomerization at pH 9, and then 1 hour incubation with hydrazine before analysis by LC-HRMS. Once the time and pH needed for isomerization was determined, peptides were then treated accordingly before incubation with other substituted hydrazine labels (40 mM) in 50 mM NaPi pH 6 for 2 hours. Each reaction was then analyzed on a Zorbax Eclipse Plus C18 column (1.8 µm, 1.2 x 50 mm, room temperature, Agilent) using a linear gradient from 5 to 95% acetonitrile over 7.5 min with 0.1% formic acid as the aqueous mobile phase after an initial hold at 95% 0.1% formic acid for 0.5 min (0.7 mL/min) using an 1290 Infinity II UHPLC (G7120AR, Agilent).

General Biochemistry Methods

Methods to Support Figure 6A

DNA templates used for transcribing cDNA for in vitro translation as well as E. coli $tRNA^{Val}_{AsnE2}$ were prepared using polymerase chain reactions (PCR) by annealing and extending overlapping primers.[2] The templates were then purified from protein components with a 1:1 (v/v)

phenol/chloroform solution and precipitated in 3 volumes of 75% (v/v) ethanol. T7 HiScribe RNA synthesis kit (New England Biolabs (NEB)) was used to generate each tRNA in 200 µL reactions containing 10 µg of DNA template. Transcription reactions were incubated at 37° C for 4 h, and then treated with 100 U of RNAse-free DNAse I (Sigma-Aldrich) for an additional 2 h to digest template DNA. Sodium acetate pH 5.2 was added to a final concentration of 200 mM and isolated using a Quick-RNA miniprep kit (Zymo Research).

In vitro tRNA acylation was performed with the synthetase FRS1, and activity was confirmed to yield 65-80% acyl tRNA in triplicate experiments. cDNA templates and tRNA were made in vitro as previously described.[3] Bis-Tris pH 6.5 (100mM), $MgCl_2$ (10 mM), DTT (4 mM), ATP (10 mM), substrate (10 mM), iPPase (0.004 U/µL), tRNA (25 µM), and FRS1 (25 µM) were combined and incubated for 2 – 6 hours.[4] tRNA was isolated from the mixture using a Quick-RNA miniprep kit, and used directly in in vitro translation experiments following intact RNA LC-MS analysis as previously described.[4]

Methods to Support Figure 6B–F

In vitro transcription/translation of short peptides were carried out using the PureExpress (ΔtRNA, Δaa (E6840S)) kit by New England Biolabs with minor modifications. For each reaction on a 12.5 µL scale: Solution A ((ΔtRNA, Δaa) (2.5 µL), an amino acid mixture containing only the encoded residues at 33 mM (0.25 µL), tRNA solution (1.25 µL), Solution B (3.75 µL), 500 ng dsDNA template (0.25 µL), and water (to 12.5 µL). Met was omitted from the reaction mixture. When using precharged tRNA, Valine was also omitted from the reaction mixture. The reactions were then incubated for 2 h at 37ºC. The translated peptides were first passed through a molecular cutoff filter (Pall Corporation) to remove protein components. The mixture was first analyzed on a Zorbax Eclipse Plus C18 column (1.8 µm, 1.2 x 50 mm, room temperature, Agilent) using a linear gradient from 0 to 55% acetonitrile over 6.5 min with 0.1% formic acid as the aqueous mobile phase after an initial hold at 95% 0.1% formic acid for 0.5 min (0.7 mL/min) using an 1290 Infinity II UHPLC (G7120AR, Agilent). Peptides were identified using LC-HRMS with an Agilent 6530 QTOF AJS-ESI (G6230BAR). The mixture was then desalted using an analytical RP-HPLC, using a Zorbax Eclipse Plus C18 column (1.8 µm, 1.2 x 150 mm, room temperature, Agilent) using a linear gradient from 5 to 65% acetonitrile over 30 minutes with 0.1% trifluoroacetic acid as the aqueous mobile phase (1 mL/min) using a 1260 Infinity II LC system (G7110B, Agilent). Fractions containing the peptide were identified using LC-HRMS. Extracted ion chromatograms were gathered by extracting the exact calculated mass of the molecule and peptide ± 5 ppm.

Desalted reaction mixture was removed of solvent by lyophilization, and then reconstituted in 20 mM H_2O_2 in MeCN and incubated at room temperature for 9 hours. Oxidant was removed by lyophilization, and the mixture was then dissolved in 50 mM NaPi pH 8 for 2 hour. The solution was then diluted to 20 mM hydrazine or 2-hydrazinobenzoic acid in 50 mM NaPi pH 6 for 30 minutes before analysis by LC-HRMS.

References

(1) Bartlett, P. A.; Chouinard, P. M. Stereocontrolled Synthesis of (E)- and (Z)-3-Deuteriophosphoenolpyruvate. *J. Org. Chem.* **1983**, *48* (21), 3854–3855. https://doi.org/10.1021/jo00169a064.

(2) Katoh, T.; Iwane, Y.; Suga, H. tRNA Engineering for Manipulating Genetic Code. *RNA Biol* **2017**, *15* (4–5), 453–460. https://doi.org/10.1080/15476286.2017.1343227.

(3) Ad, O.; Hoffman, K. S.; Cairns, A. G.; Featherston, A. L.; Miller, S. J.; Söll, D.; Schepartz, A. Translation of Diverse Aramid- and 1,3-Dicarbonyl-Peptides by Wild Type Ribosomes in Vitro. *ACS Cent. Sci.* **2019**, *5* (7), 1289–1294. https://doi.org/10.1021/acscentsci.9b00460.

(4) Fricke, R.; Swenson, C. V.; Roe, L. T.; Hamlish, N. X.; Shah, B.; Zhang, Z.; Ficaretta, E.; Ad, O.; Smaga, S.; Gee, C. L.; Chatterjee, A.; Schepartz, A. Expanding the Substrate Scope of Pyrrolysyl-Transfer RNA Synthetase Enzymes to Include Non-α-Amino Acids in Vitro and in Vivo. *Nat. Chem.* **2023**, *15* (7), 960–971. https://doi.org/10.1038/s41557-023-01224-y.

Supplementary Materials Section 1: Supplementary Figures

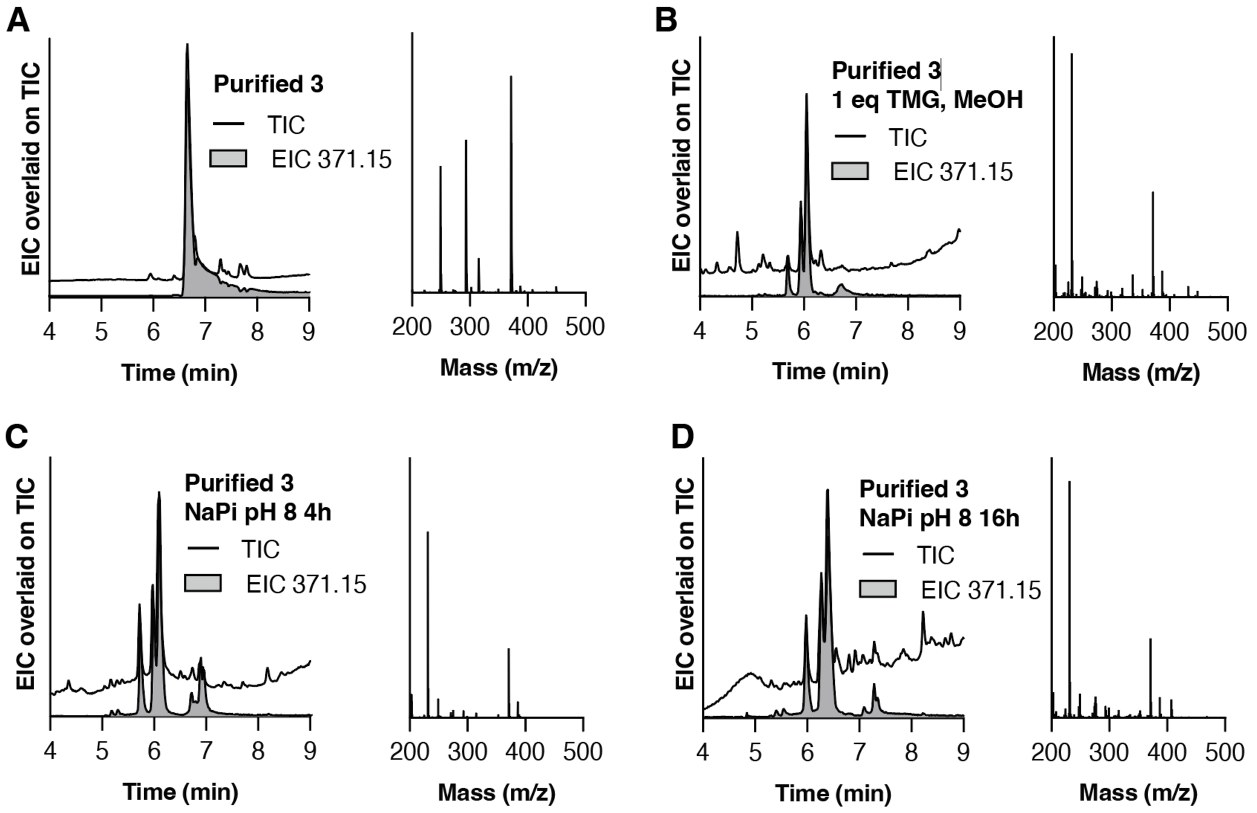

Figure S1. DHL-peptide **12** isomerizes under basic conditions. (A) LC-HRMS trace (left) and mass spectrum (right) of purified DHL-peptide 12, as seen in Figure 3. (B) LC-HRMS trace (left) and mass spectrum (right) following incubation of DHL-peptide **12** with 1 equivalent TMG in methanol for 15 minutes. (B–C) LC-HRMS trace (left) and mass spectrum extracted over the entire range of the chromatogram (right) following incubation of DHL-peptide **12** with 50 mM NaPi pH 8 for (C) 4 hours (as seen in Figure 3) and (D) 16 hours.

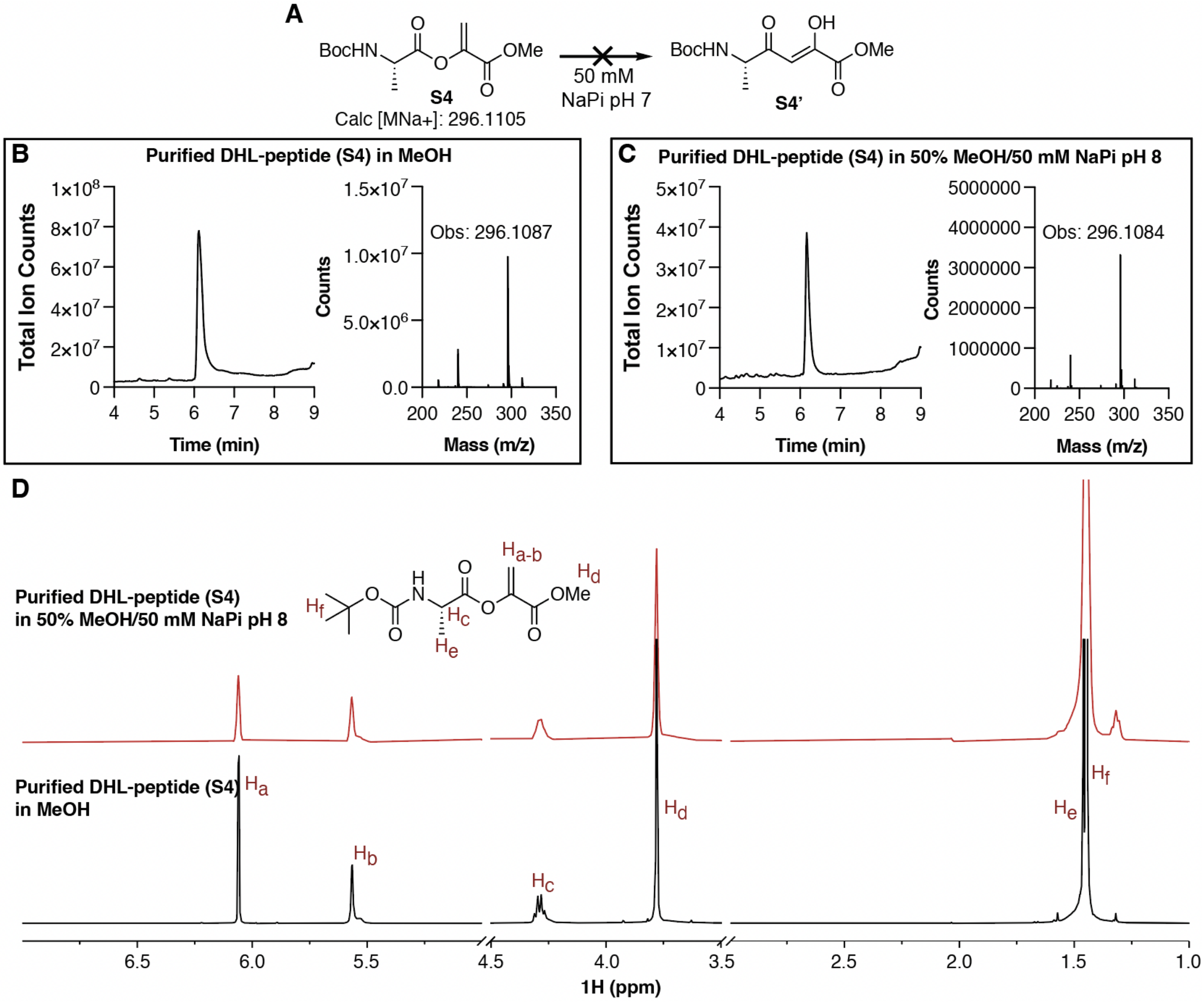

Figure S2. Methyl ester DHL analog **S4** does not undergo rearrangement. (A) Scheme illustrates that the methyl ester (**S4**) analog of DHL-peptide **3** does not undergo DHL rearrangement in base. (B) LC-MS chromatogram (left) and mass spectrum (right) corresponding to DHL-peptide (**S4**) in MeOH. (C) LC-MS chromatogram (left) and mass spectrum (right) corresponding to DHL-peptide (**S4**) in 50% MeOH and 50 mM NaPi pH 8 following 1 h incubation. (D) 1H NMR spectrum of DHL-peptide (**S4**) in methanol-d4 (bottom) and DHL-peptide (**S4**) in 50% methanol-d4 and 50 mM NaPi pH 8 (top).

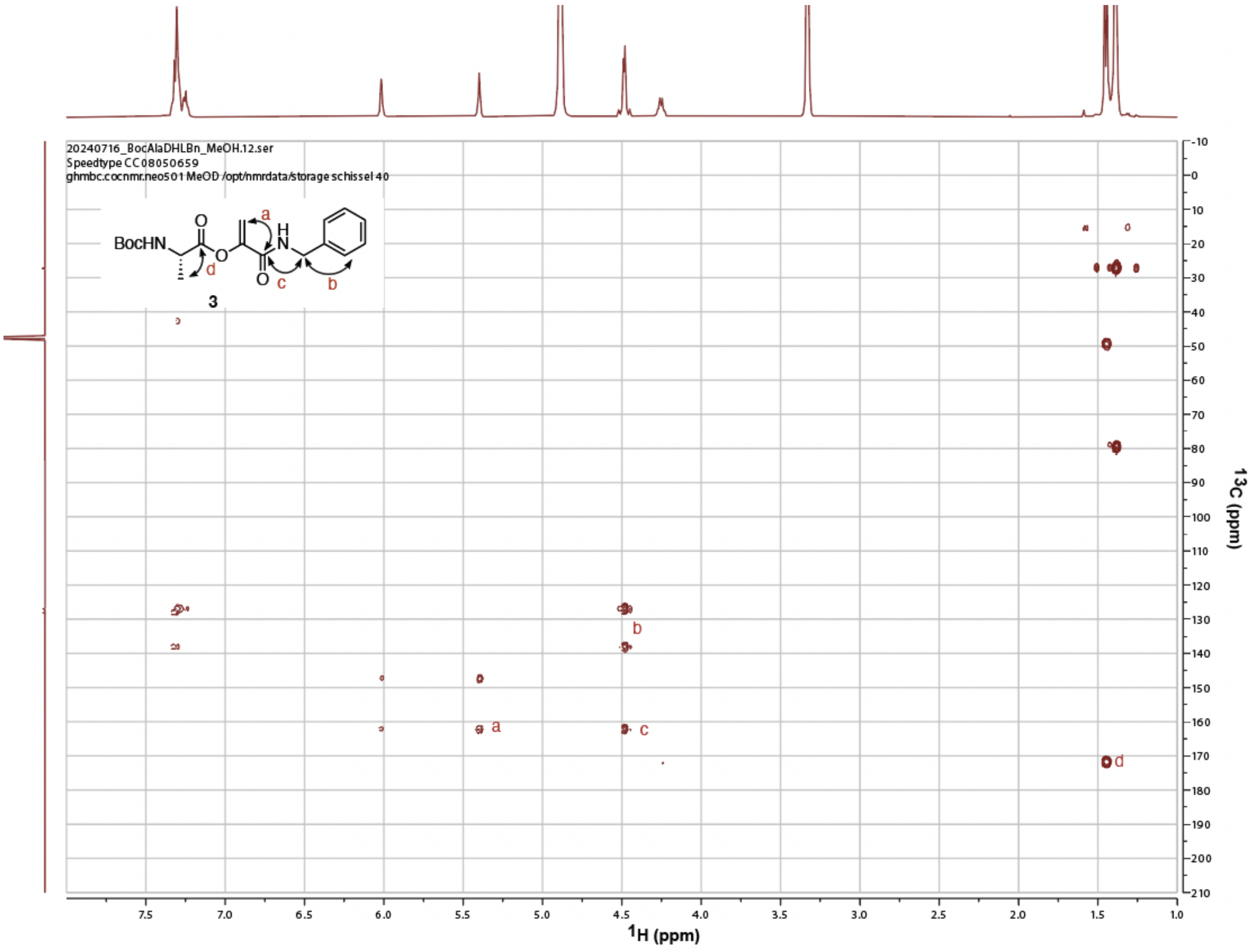
20240716_BocAlaDHLBn_MeOH.12.ser
Speedtype CC08050659
ghmbc.cocnmr.neo501 MeOD /opt/nmrdata/storage schissel 40
BocHN
3
a
b
c
d
^{1}H (ppm)
^{13}C (ppm)

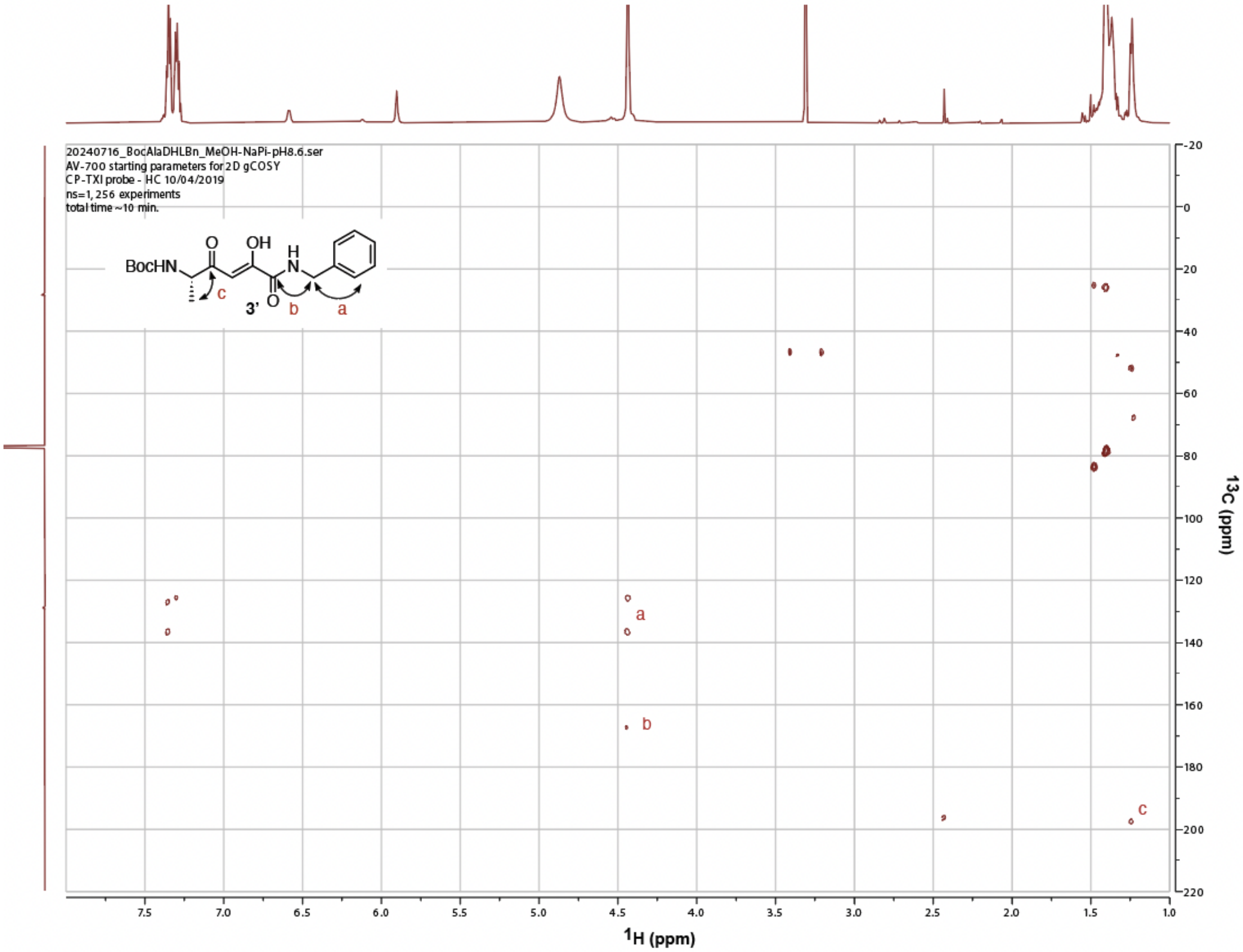
20240716_BocAlaDHLBn_MeOH-NaPi-pH8.6.ser
AV-700 starting parameters for 2D gCOSY
CP-TXI probe - HC 10/04/2019
ns=1, 256 experiments
total time ~10 min.
BocHN
OH
3'
a
b
c
^{1}H (ppm)
^{13}C (ppm)

Figure S3 Heteronuclear Multiple Bond Correlation (HMBC) in methanol-d_4 correlations for DHL-tripeptide **3** before (top) and after incubation with 50% 50 mM NaPi pH 8 (bottom)**.**

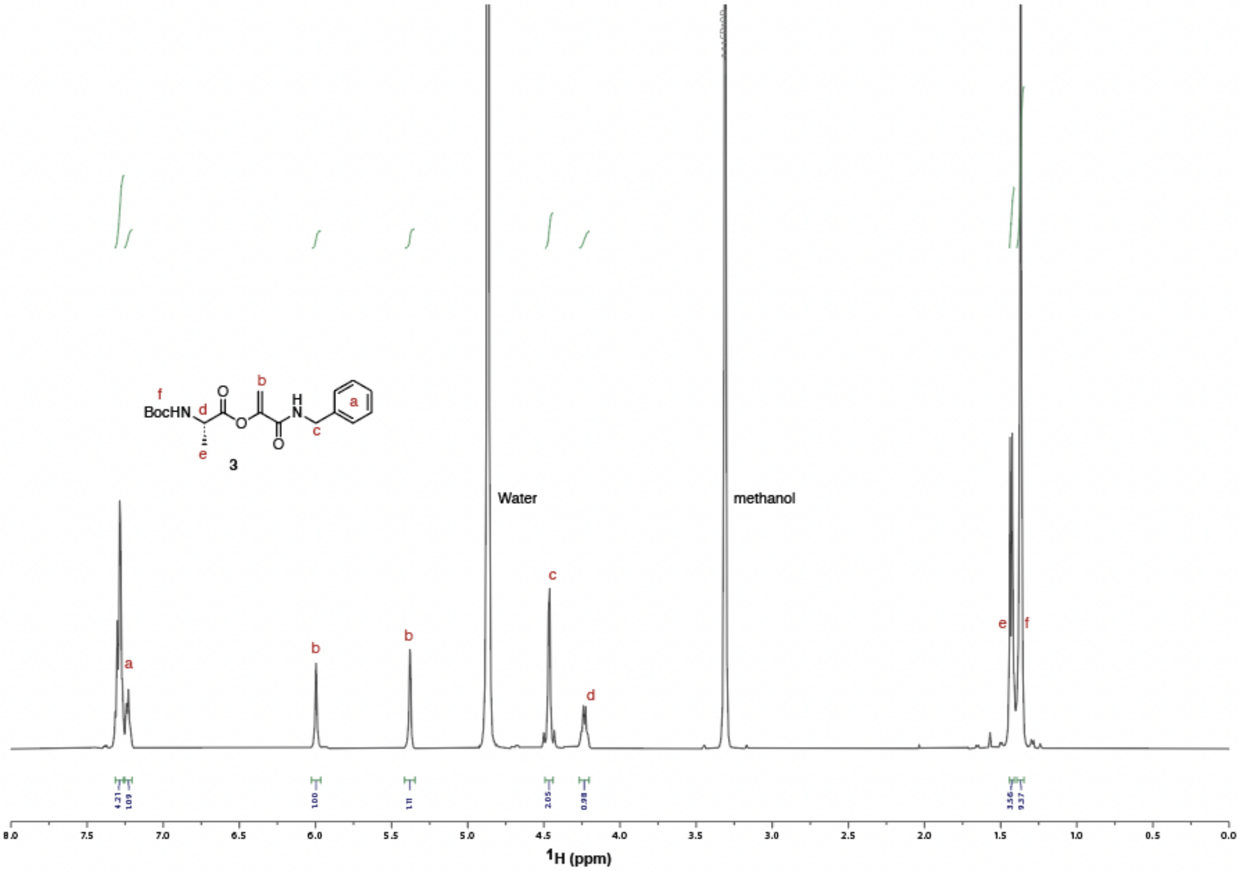
BocHN
3
Water
methanol
a
b
b
c
d
e
f
4.21
1.09
1.00
1.11
2.05
0.98
3.56
9.37
8.0
7.5
7.0
6.5
6.0
5.5
5.0
4.5
4.0
3.5
3.0
2.5
2.0
1.5
1.0
0.5
0.0
1H (ppm)

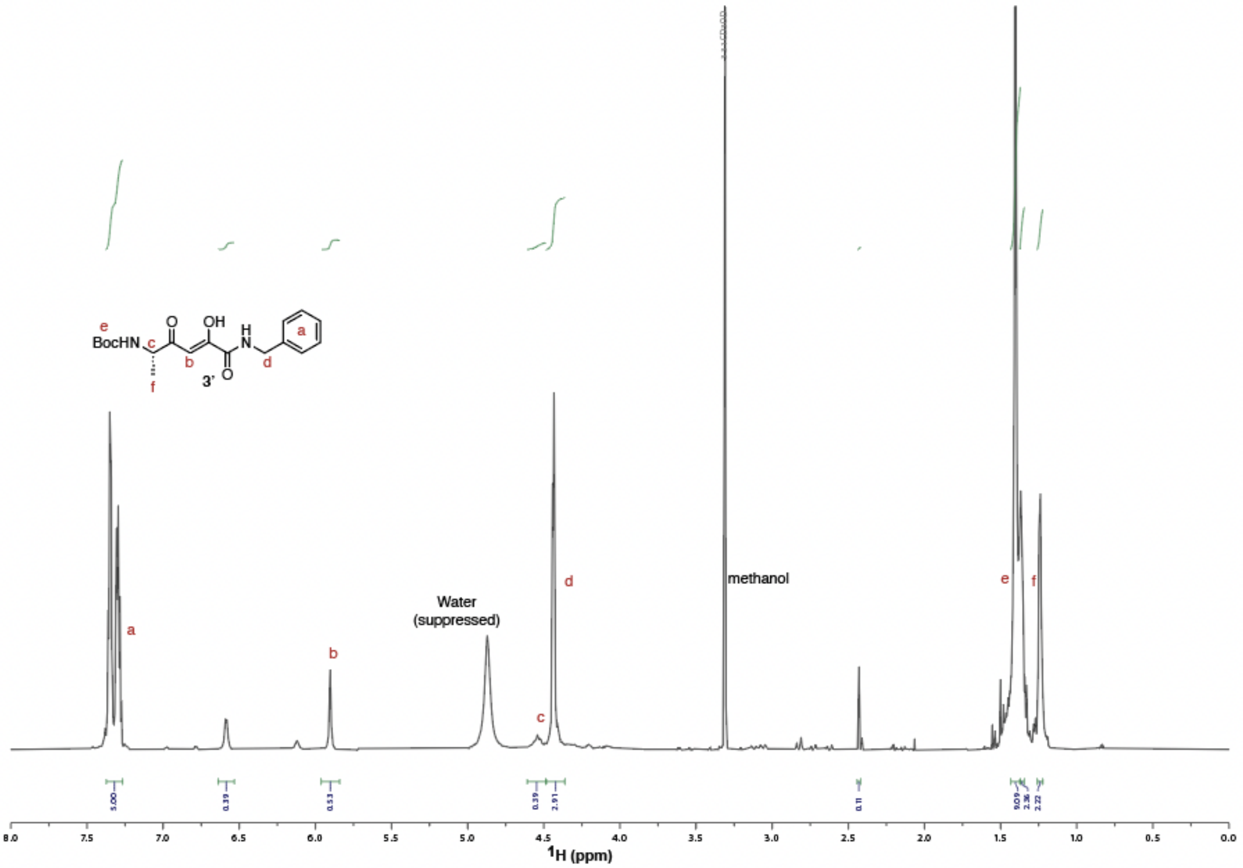
BocHN
OH
3'
Water
(suppressed)
methanol
a
b
c
d
e
f
5.00
0.39
0.53
0.39
2.91
0.11
9.09
2.36
2.22
8.0
7.5
7.0
6.5
6.0
5.5
5.0
4.5
4.0
3.5
3.0
2.5
2.0
1.5
1.0
0.5
0.0
1H (ppm)

Figure S4 1H NMR in methanol-d_4 of DHL-tripeptide **3** before (top) and after incubation with 50% 50 mM NaPi pH 8 (bottom)

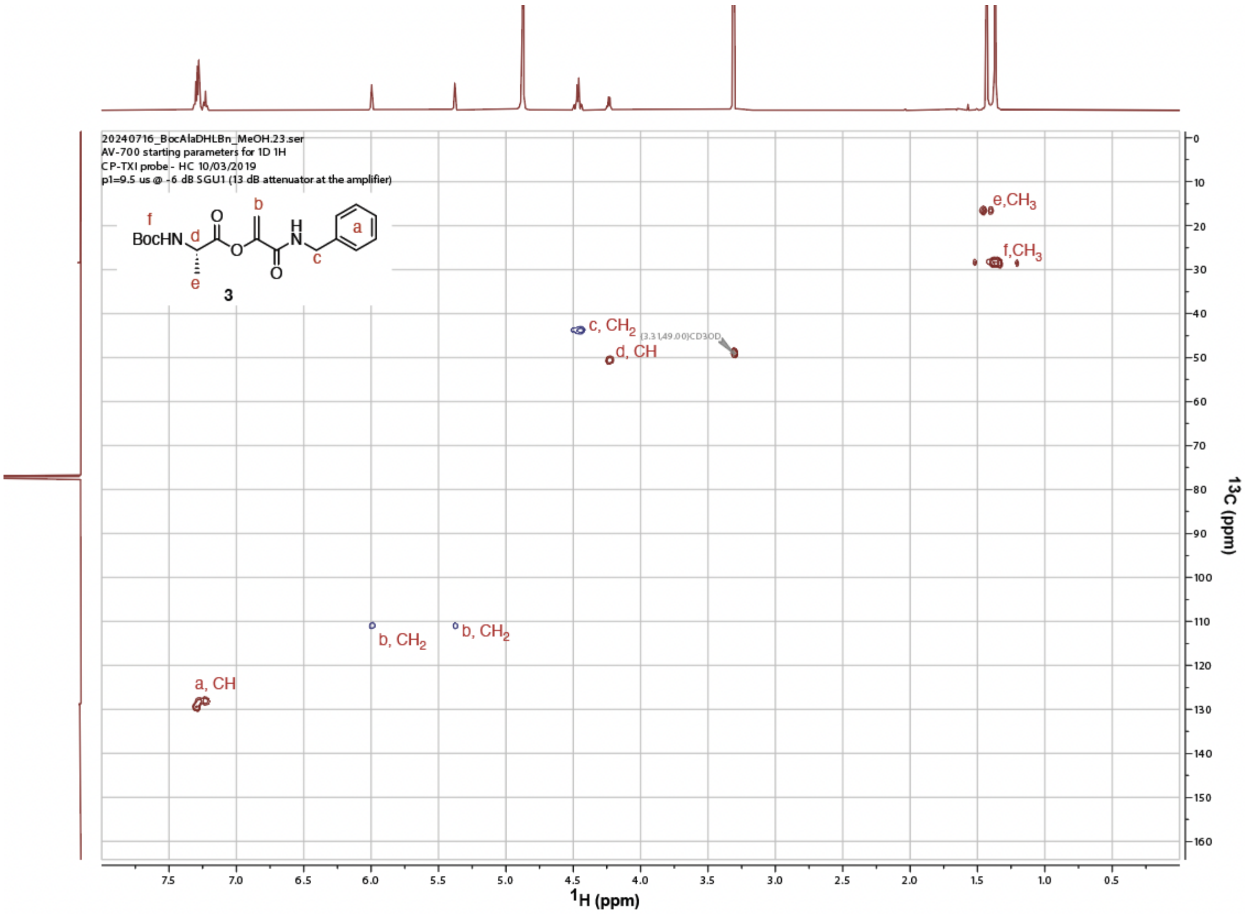

20240716_BocAlaDHLBn_MeOH.23.ser
AV-700 starting parameters for 1D 1H
CP-TXI probe - HC 10/03/2019
p1=9.5 us @ -6 dB SGU1 (13 dB attenuator at the amplifier)
BocHN
3
e,CH3
f,CH3
c, CH2
d, CH
(3.31,49.00)CD3OD
b, CH2
b, CH2
a, CH
1H (ppm)
13C (ppm)

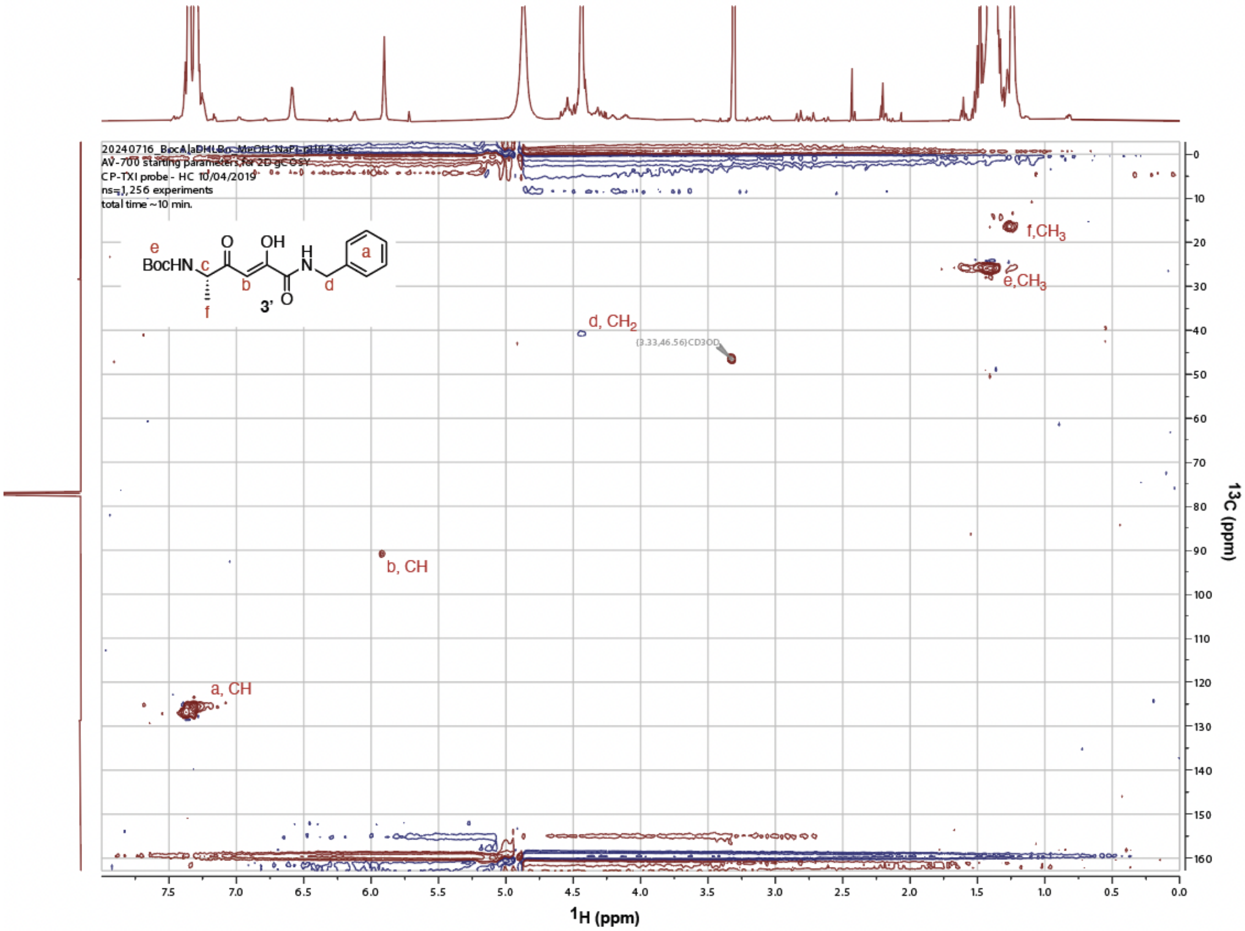

20240716_BocAlaDHLBn_MeOH-NaPi-pH4.ser
AV-700 starting parameters for 2D gCOSY
CP-TXI probe - HC 10/04/2019
ns=1,256 experiments
total time ~10 min.
BocHN
OH
3'
f,CH3
e,CH3
d, CH2
(3.33,46.56)CD3OD
b, CH
a, CH
1H (ppm)
13C (ppm)

Figure S5. Heteronuclear Single Quantum Coherence (HSQC) in methanol-d_4 correlations for DHL-tripeptide **3** before (top) and after incubation with 50% 50 mM NaPi pH 8 (bottom)

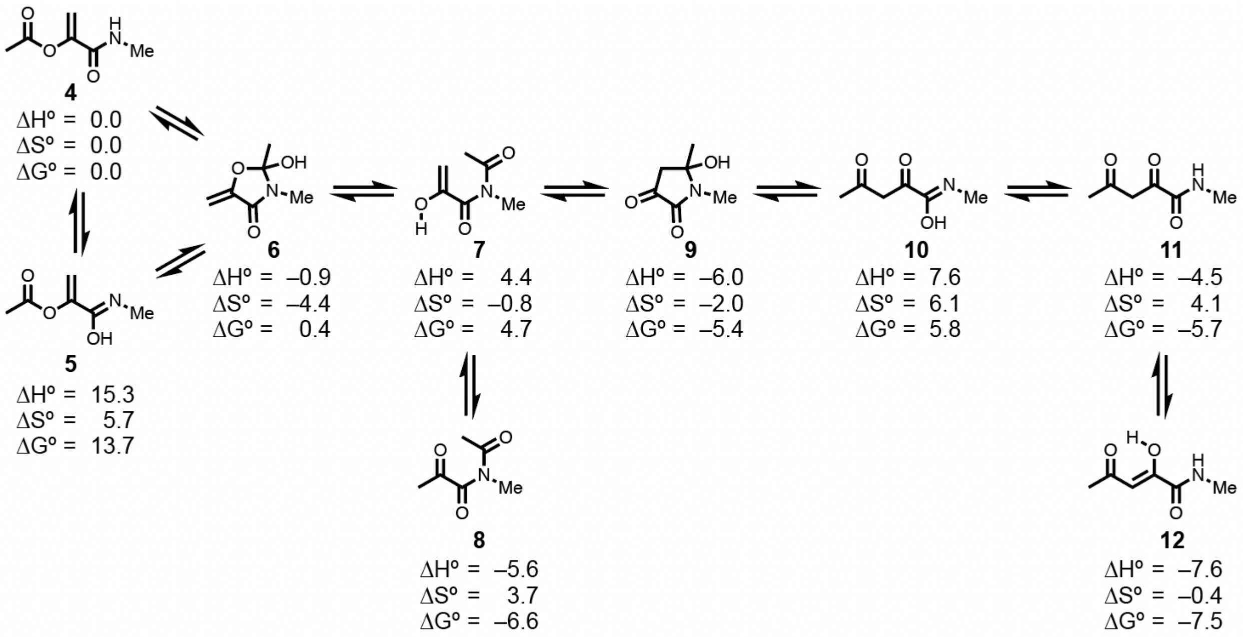

Figure S6. Proposed reaction pathway for DHL ester 4 to form ketone product 12. For each intermediate, DFT calculations were used to predict the ethalpy and entropy changes relative to starting compound 4. Geometry optimizations and frequency calculations were performed at the B3LYP-D4/6-31G** level, with the addition of an implicit CPCM aqueous solvation model. Final electronic energies were determined using ωB97M-V/def2-TZVPPD, again with an aqueous CPCM solvation model. ΔH° and ΔG° values are reported in kcal/mol. ΔS° values are reported in cal/(mol•K). Compound coordinates and raw energy values are available as a separate spreadsheet file.

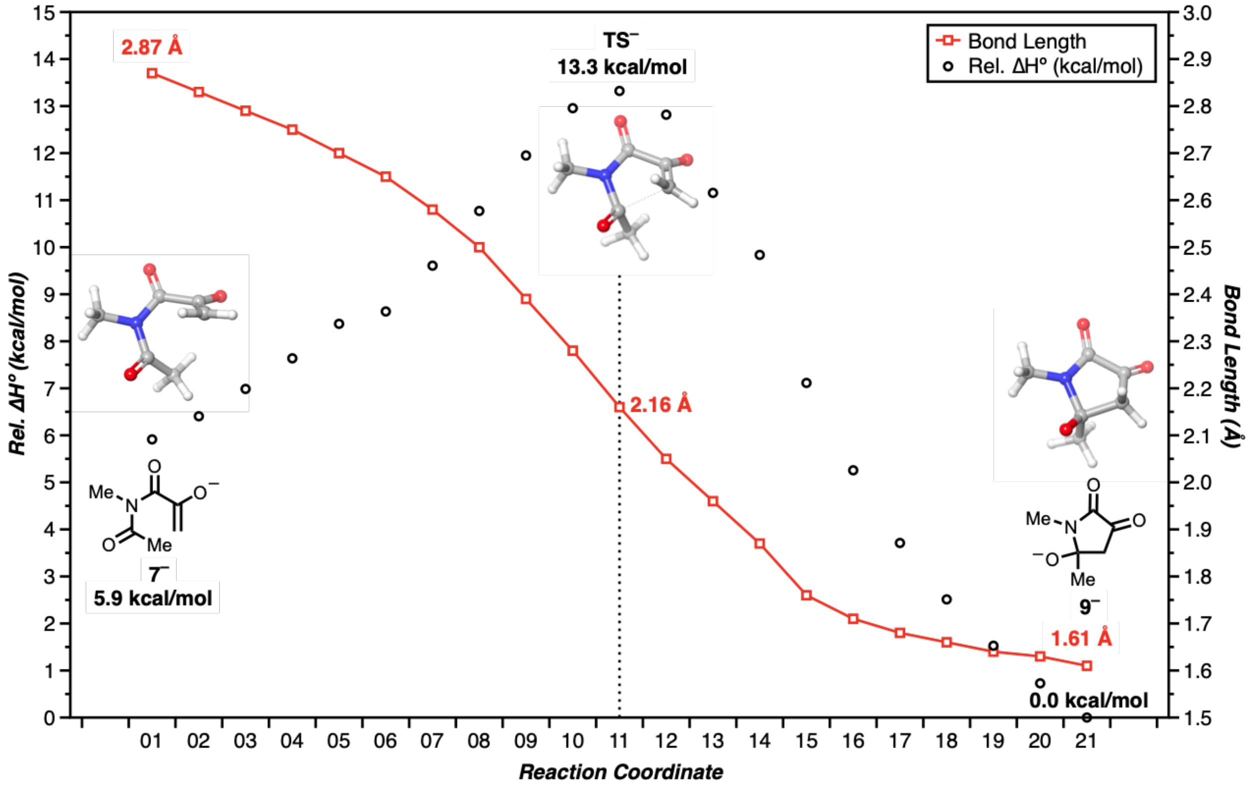

Figure S7. Intrinsic reaction coordinate diagram showing the transformation of enolate **7-** to cyclic intermediate **9-** via C–C bond formation. Relative enthalpies reported, with values in kcal/mol. Theory: B3LYP-D4 / 6-31G** / CPCM for transition state identification and IRC profile; wB97M-V / def2-TZVPPD / CPCM for final electronic energies.

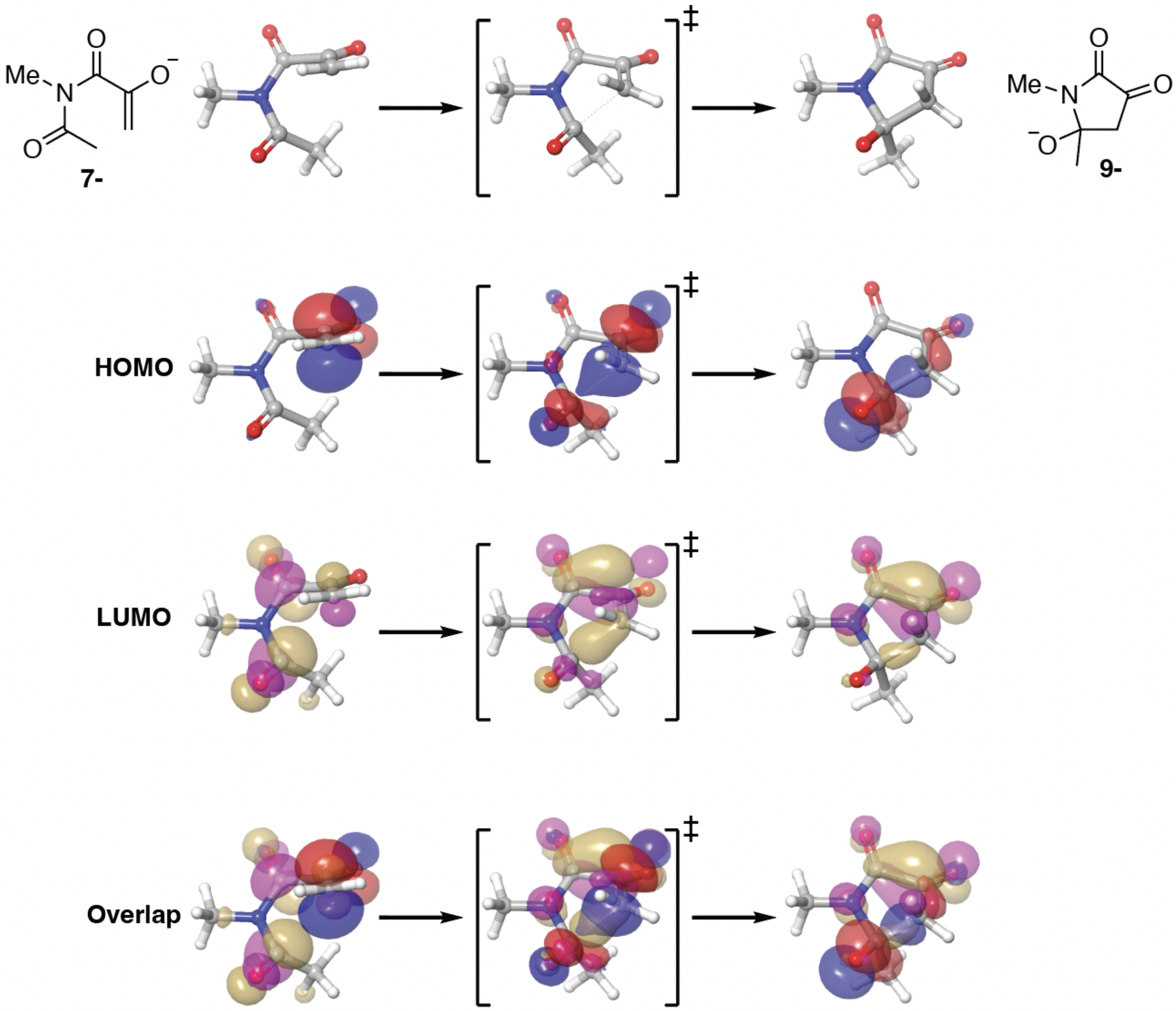

Figure S8. Transition state analysis and orbital visualization of the transformation between enolate **7-** and cyclic intermediate **9-** demonstrate a low barrier (13.3 kcal/mol) and good orbital overlap between HOMO and LUMO. Theory: B3LYP-D4 / 6-31G** / CPCM. Taken from structures on IRC profile.

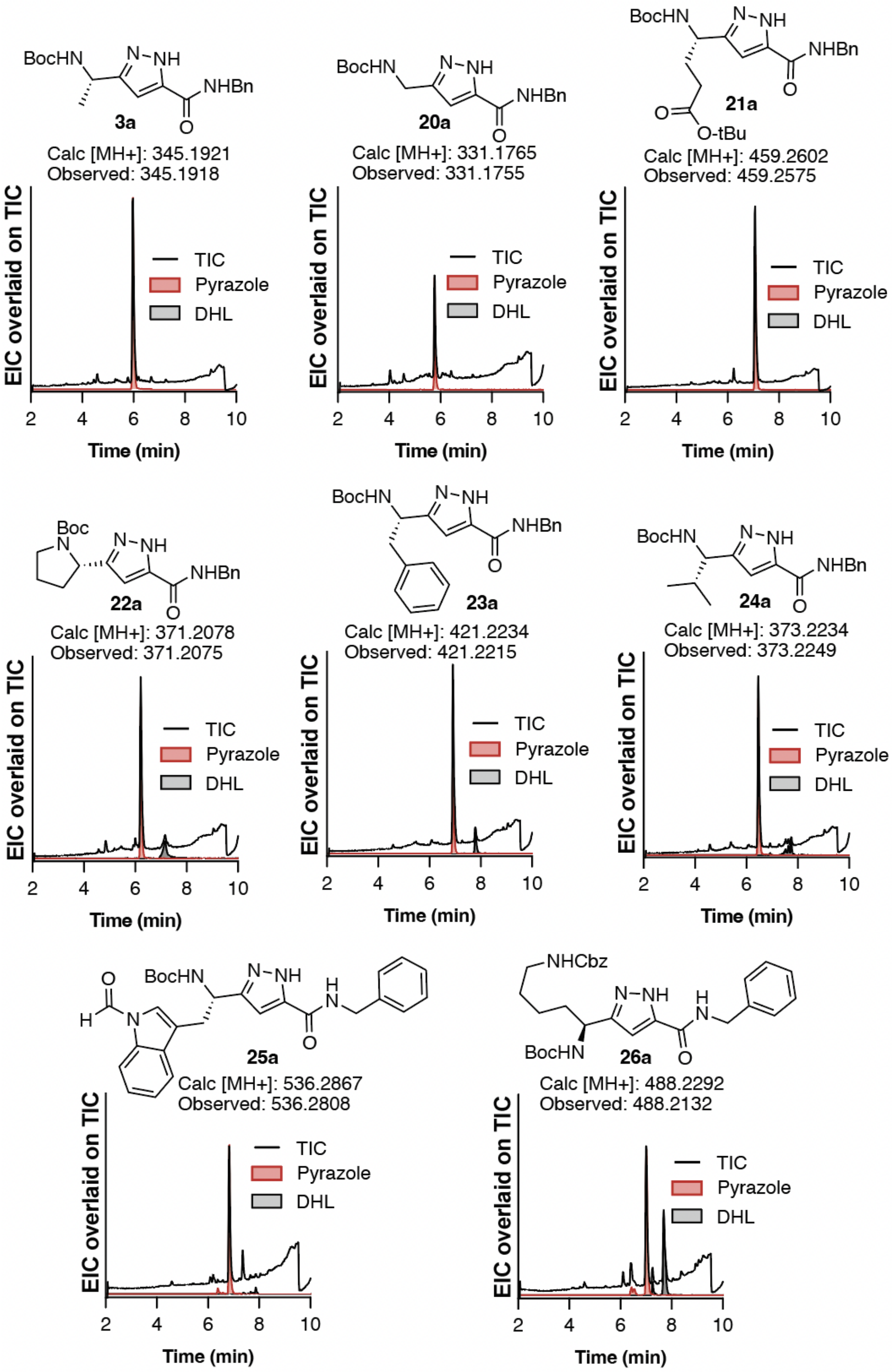

Figure S9. Extracted ion chromatograms (EIC) and total ion chromatograms (TIC) following reaction with DHL tripeptides (**3, 20-26**) and hydrazine to form pyrazole-peptides **3a, 20a-26a**. the EIC of pyrazole-peptide is shown as red, and the EIC of remaining DHL-peptide is shown as gray.

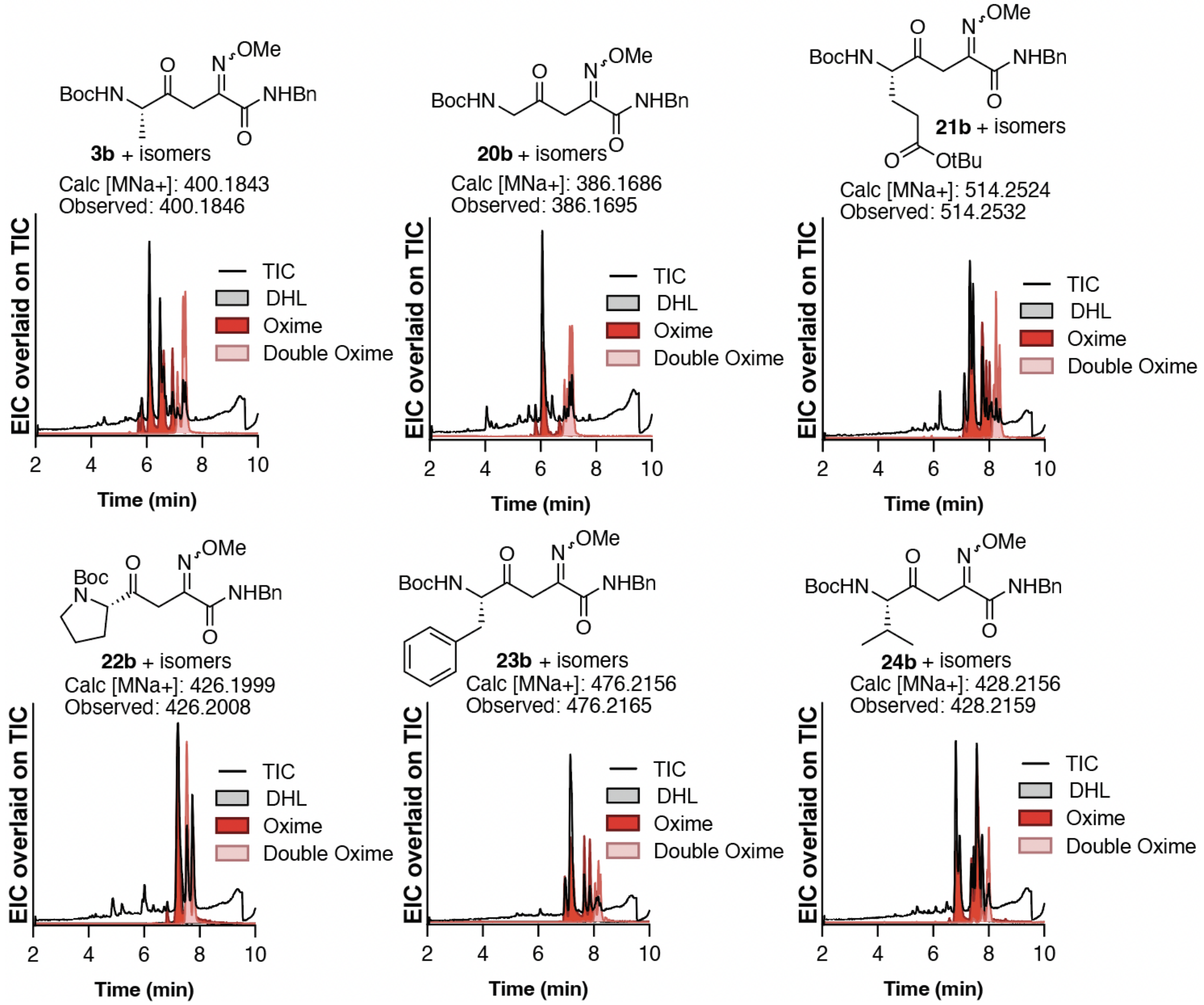

Figure S10. Extracted ion chromatograms (EIC) and total ion chromatograms (TIC) following reaction with DHL tripeptides (**3, 20-24**) and *O*-methyl hydroxylamine to form oxime-peptides **3b, 20b-24b**. The EIC of oxime-peptide is shown as red, the EIC of double oxime addition is shown as pink, and the EIC of remaining DHL-peptide is shown as gray.

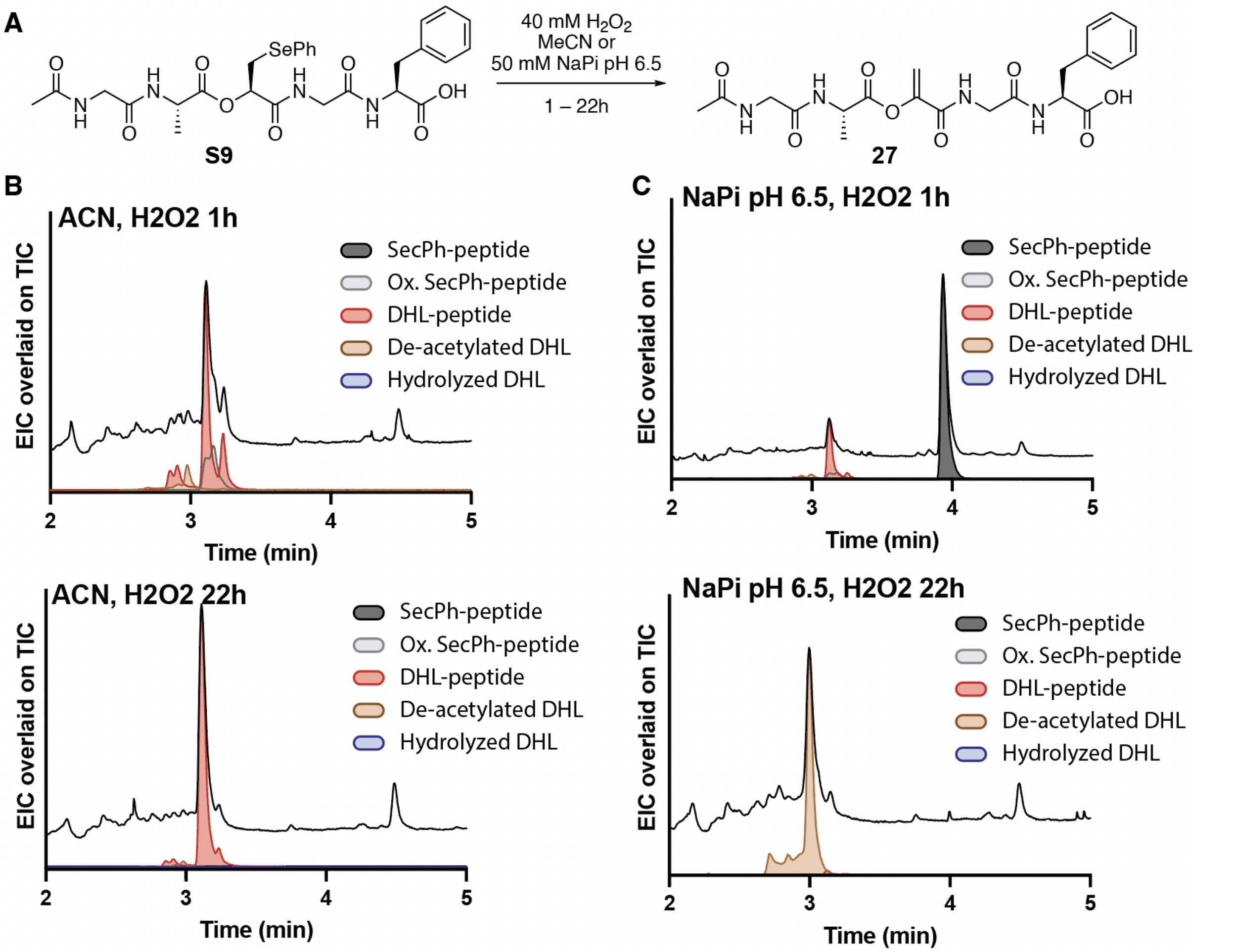

Figure S11. HO-SecPh peptides more rapidly form DHL in MeCN than in buffer. (A) OH-SecPh-peptide **S9** was treated with 20 eq. H_2O_2 in either (B) acetonitrile or (C) 50 mM NaPi pH 6.5 for 1h or 22 h. (B–C) Extracted ion chromatograms (EIC) corresponding to OH-SecPh-peptide **S9** (gray) and DHL-peptide **27** (pink) are overlaid on the total ion chromatogram (TIC)**.** In water, removal of the acetyl group from the N-terminus is also observed (yellow).

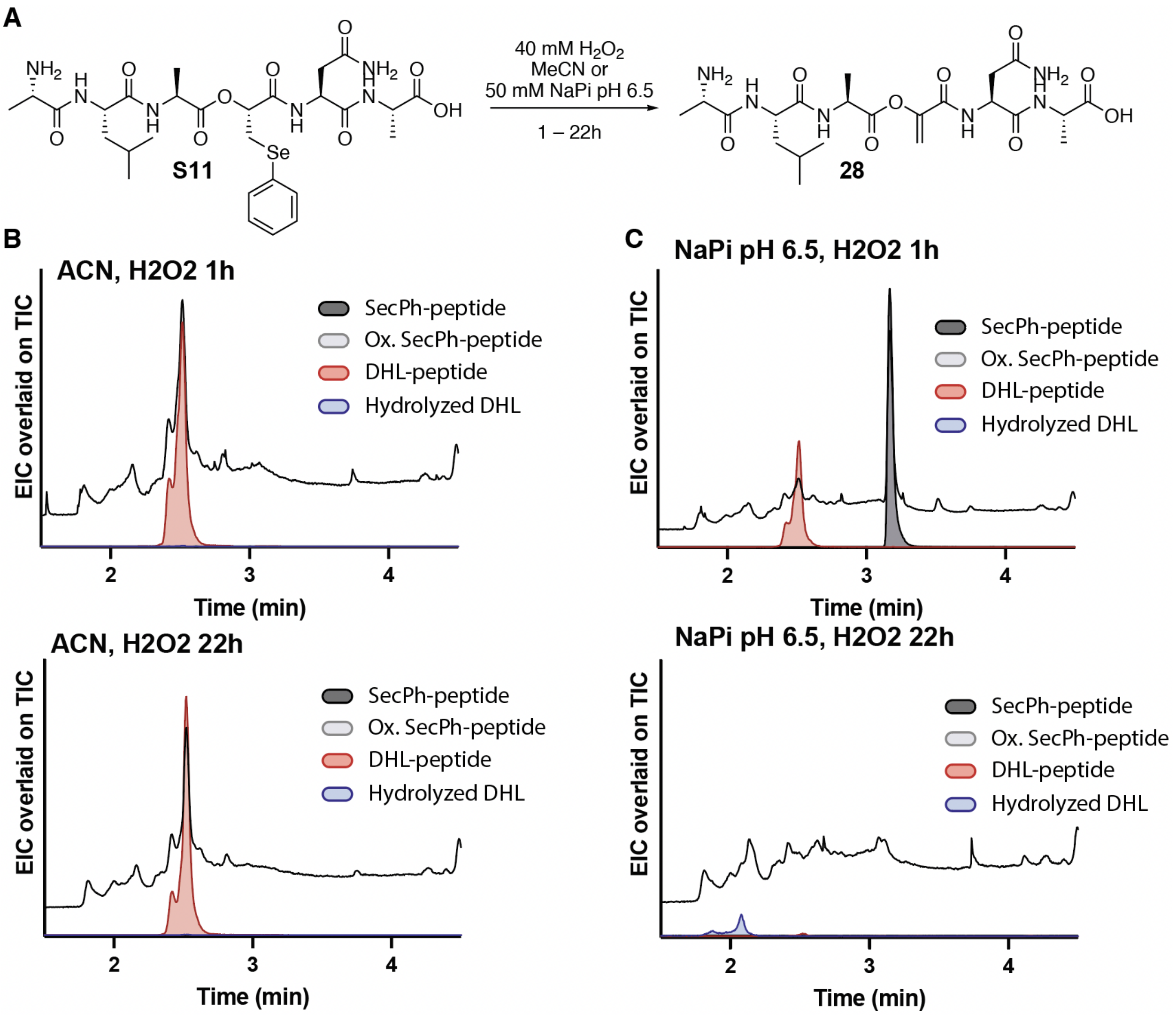

Figure S12. HO-SecPh peptides more rapidly form DHL in MeCN than in buffer. (A) OH-SecPh-peptide **S10** was treated with 20 eq. H_2O_2 in either (B) acetonitrile or (C) 50 mM NaPi pH 6.5 for 1h or 22h. (B–C) Extracted ion chromatograms (EIC) corresponding to OH-SecPh-peptide **S10** (gray) and DHL-peptide **27** (pink) are overlaid on the total ion chromatogram (TIC)**.** In water, cleavage of the ester bond in **27** is observed (blue).

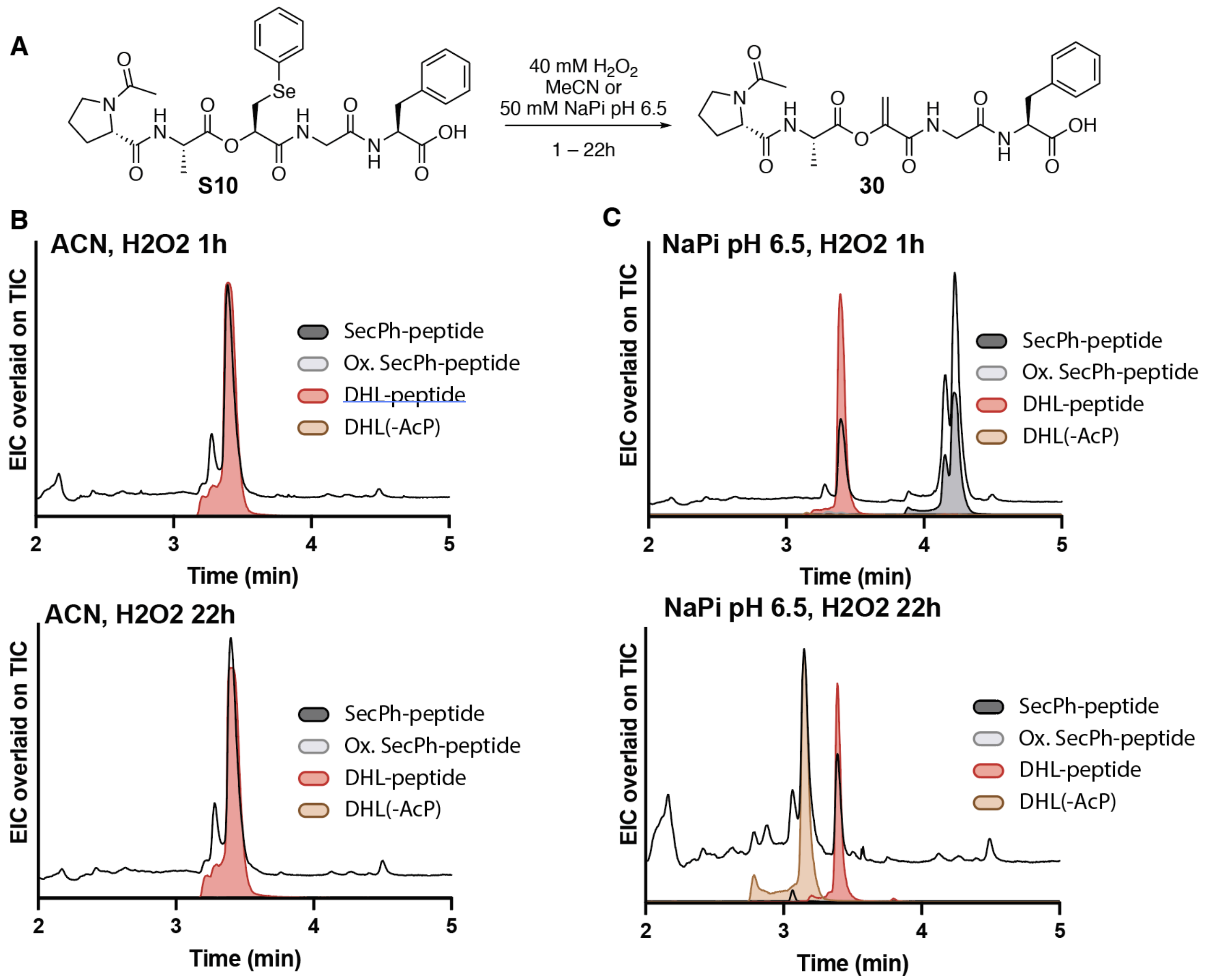

Figure S13. HO-SecPh peptides more rapidly form DHL in MeCN than in buffer. (A) OH-SecPh-peptide **S11** was treated with 20 eq. H_2O_2 in either (B) acetonitrile or (C) 50 mM NaPi pH 6.5 for 1h or 22h. (B–C) Extracted ion chromatograms (EIC) corresponding to OH-SecPh-peptide **S11** (gray) and DHL-peptide **30** (pink) are overlaid on the total ion chromatogram (TIC)**.** In water, cleavage of the N-terminal acetyl-Proline is also observed (yellow).

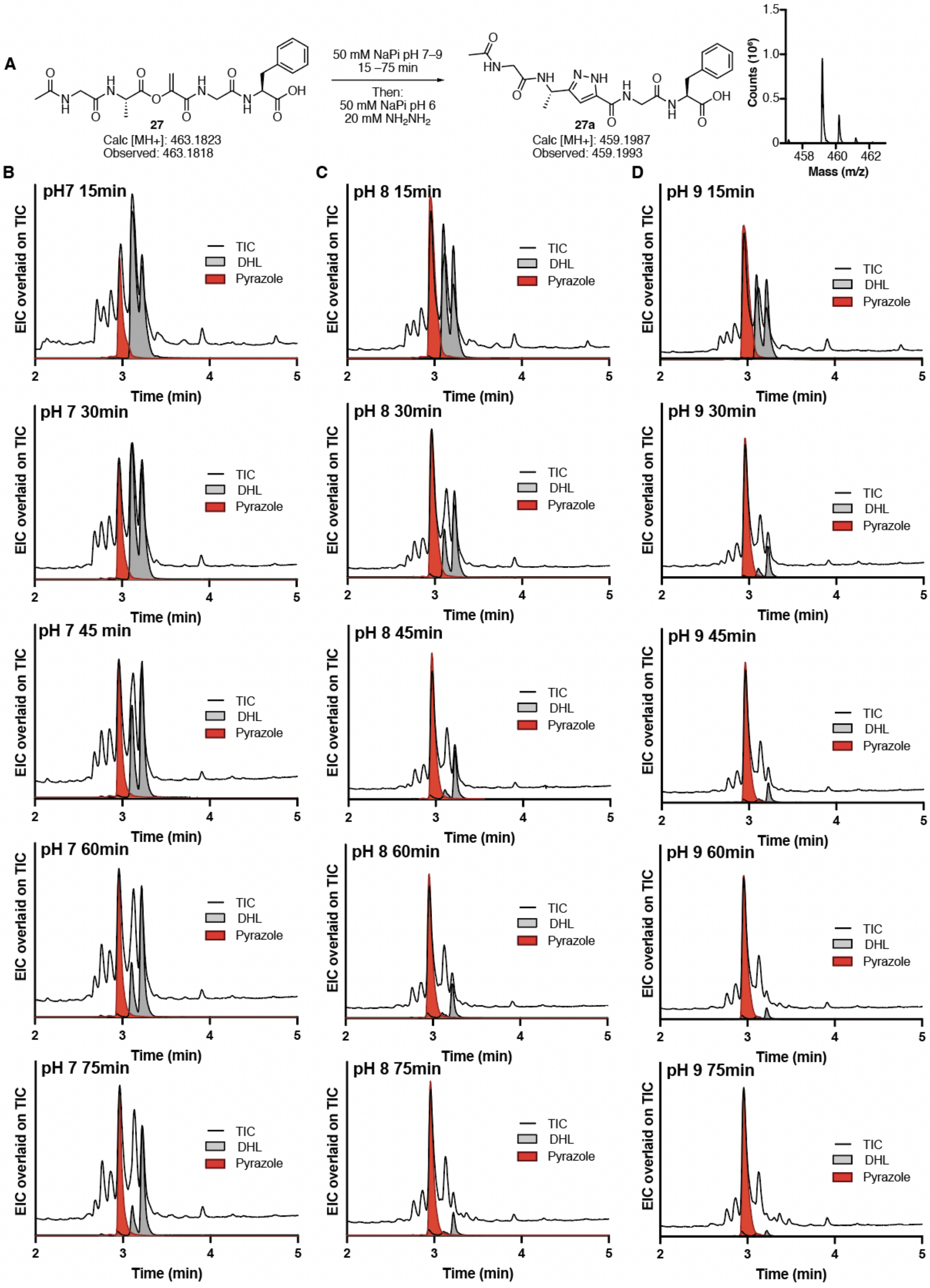
A
27
Calc [MH+]: 463.1823
Observed: 463.1818
50 mM NaPi pH 7–9
15 –75 min
Then:
50 mM NaPi pH 6
20 mM NH_2NH_2
27a
Calc [MH+]: 459.1987
Observed: 459.1993
Counts (10^6)
Mass (m/z)
B
C
D
pH7 15min
pH 7 30min
pH 7 45 min
pH 7 60min
pH 7 75min
pH 8 15min
pH 8 30min
pH 8 45min
pH 8 60min
pH 8 75min
pH 9 15min
pH 9 30min
pH 9 45min
pH 9 60min
pH 9 75min
EIC overlaid on TIC
Time (min)
TIC
DHL
Pyrazole

Figure S14. DHL-peptide isomerization to the reactive diketone is pH and time-dependent. (A) DHL-peptide **27** was incubated in NaPi buffer pH 7, 8, or 9 between 15 – 75 mins, and then diluted in buffer containing hydrazine at pH 6 and incubated for 15 min before analysis by LC-HRMS. Mass spectrum of pyrazole-peptide **27a** (right) (B) Time course from pH 7 condition. (C) Time course from pH 8 condition. (D) Time course from pH 9 condition. (B-D) Extracted ion chromatograms (EIC) corresponding to DHL-peptide (gray) and pyrazole-peptide (red) are overlaid on the total ion chromatogram (TIC).

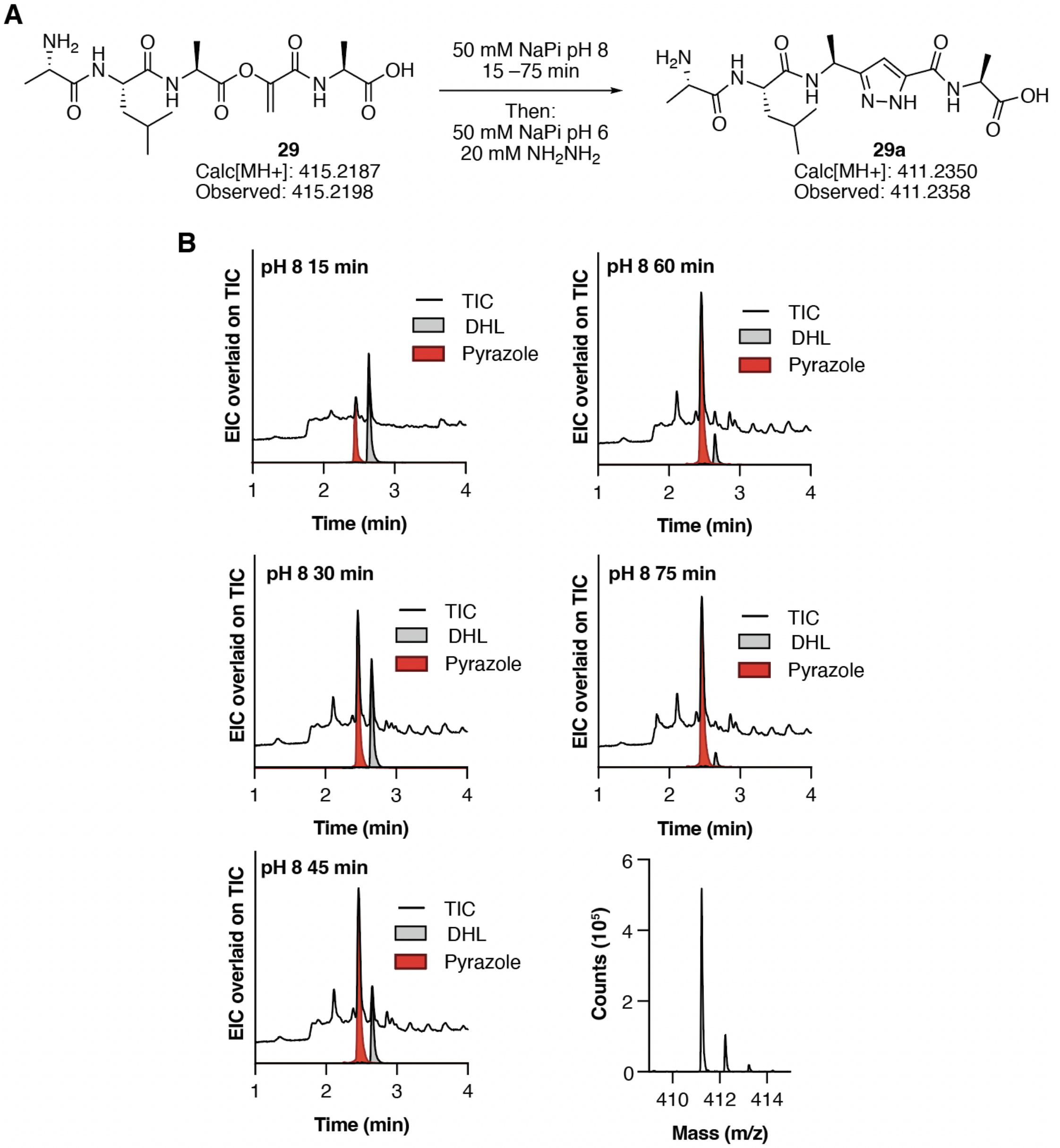

Figure S15. DHL-peptide isomerization to the reactive diketone is time-dependent. (A) DHL-peptide **29** was incubated in NaPi buffer pH 8 between 15 – 75 mins, and then diluted in buffer containing hydrazine at pH 6 and incubated for 15 min before analysis by LC-HRMS. (B) Time course from pH 8 condition. Extracted ion chromatograms (EIC) corresponding to DHL-peptide

(gray) and pyrazole-peptide (red) are overlaid on the total ion chromatogram (TIC). Mass spectrum of pyrazole-peptide **29a** (lower right).

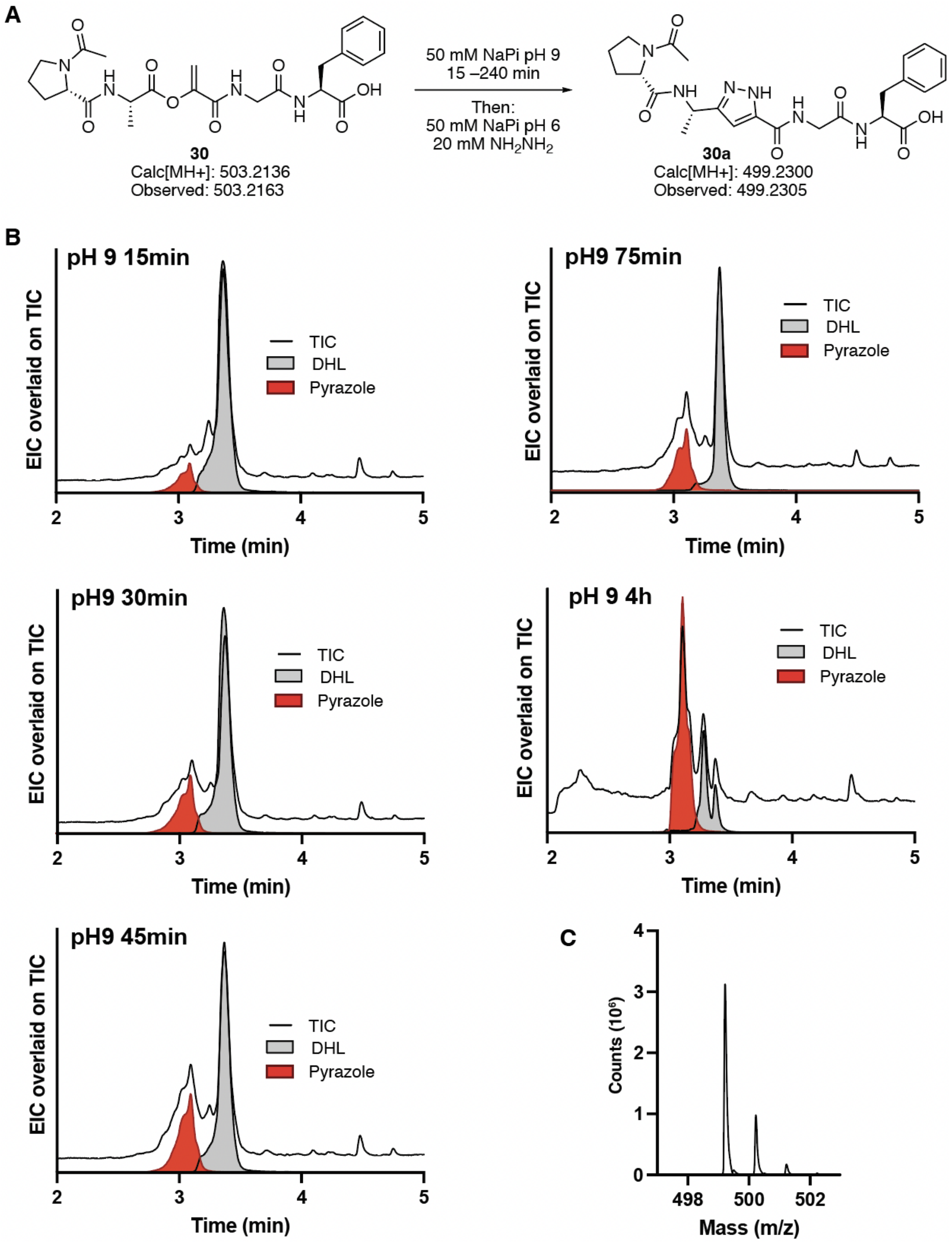

Figure S16. DHL-peptide isomerization to the reactive diketone is time-dependent. (A) DHL-peptide **30** was incubated in NaPi buffer pH 9 between 15 mins – 4h, and then diluted in buffer containing hydrazine at pH 6 and incubated for 1 hour before analysis by LC-HRMS. (B) Time course from pH 9 condition. Extracted ion chromatograms (EIC) corresponding to DHL-peptide

(gray) and pyrazole-peptide (red) are overlaid on the total ion chromatogram (TIC). (C) Mass spectrum of pyrazole-peptide **30a**.

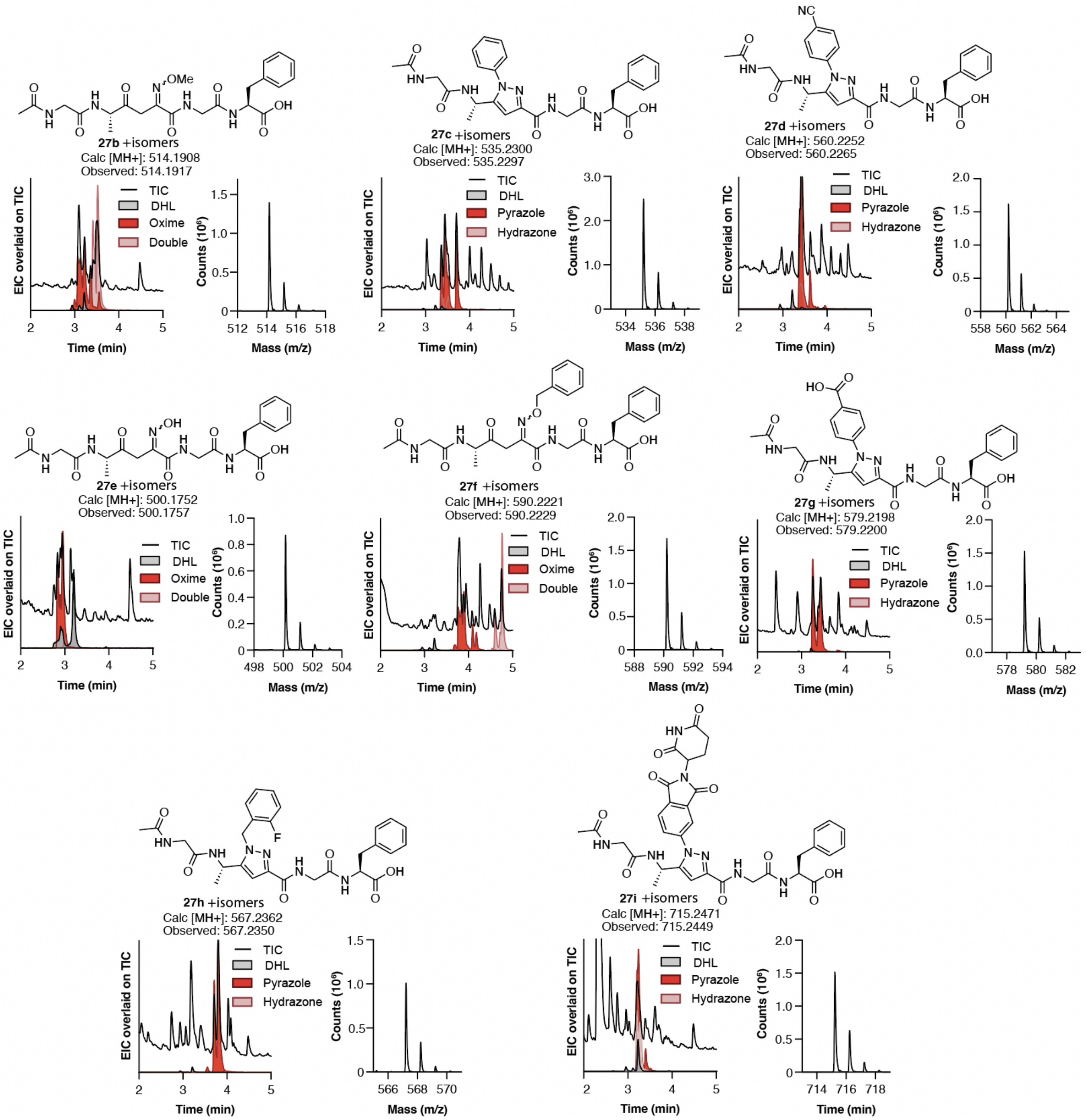

Figure S17. DHL-peptide **27** reacts with a variety of alpha-nucleophiles to form substituted pyrazoles and oximes. Extracted ion chromatograms (EIC) corresponding to DHL-peptide (gray) and pyrazole- or oxime-peptide (red) are overlaid on the total ion chromatogram (TIC).

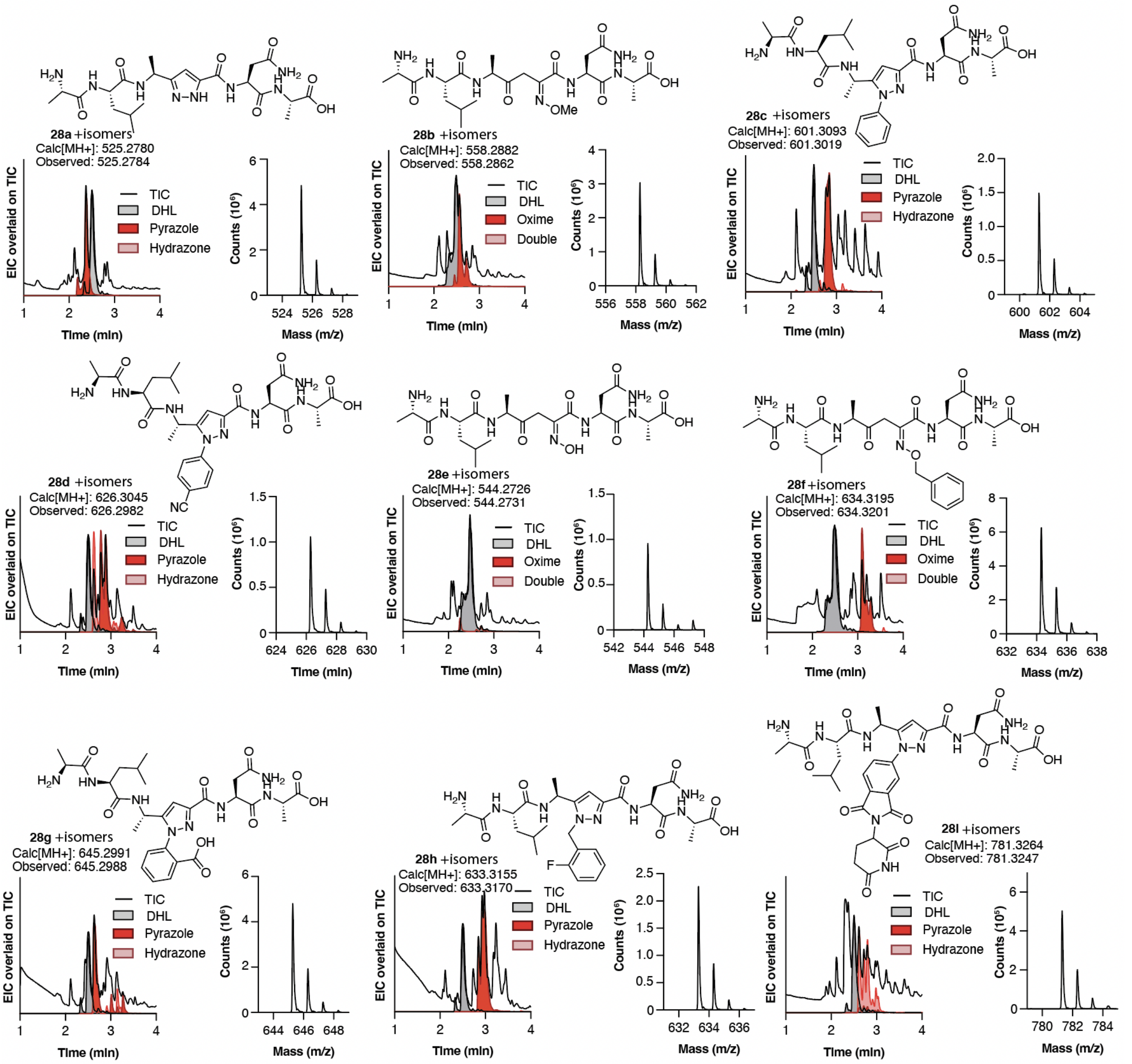

Figure S18. DHL-peptide **28** reacts with a variety of alpha-nucleophiles to form substituted pyrazoles and oximes. Extracted ion chromatograms (EIC) corresponding to DHL-peptide (gray) and pyrazole- or oxime-peptide (red) are overlaid on the total ion chromatogram (TIC).

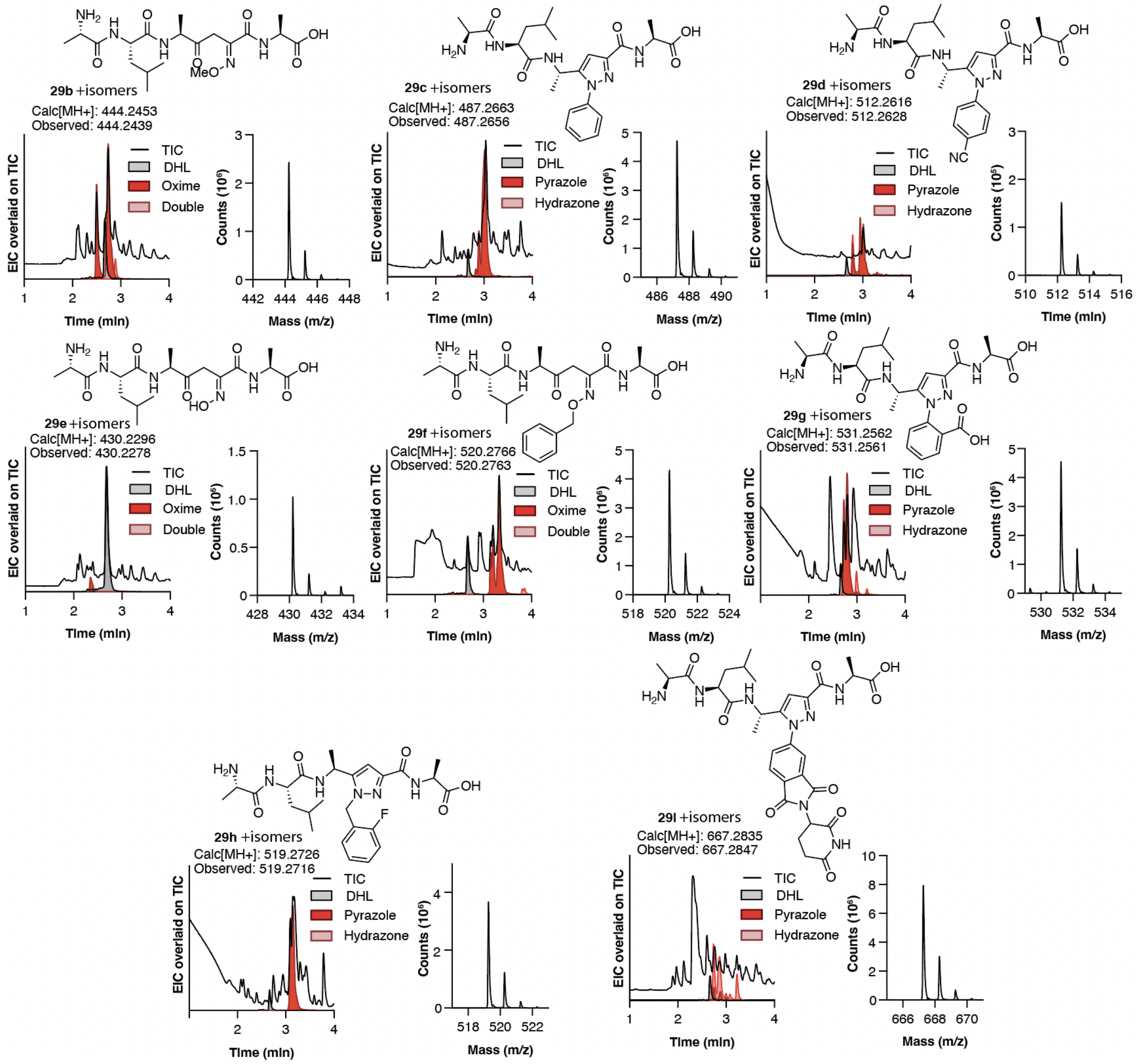

Figure S19. DHL-peptide **29** reacts with a variety of alpha-nucleophiles to form substituted pyrazoles and oximes. Extracted ion chromatograms (EIC) corresponding to DHL-peptide (gray) and pyrazole- or oxime-peptide (red) are overlaid on the total ion chromatogram (TIC).

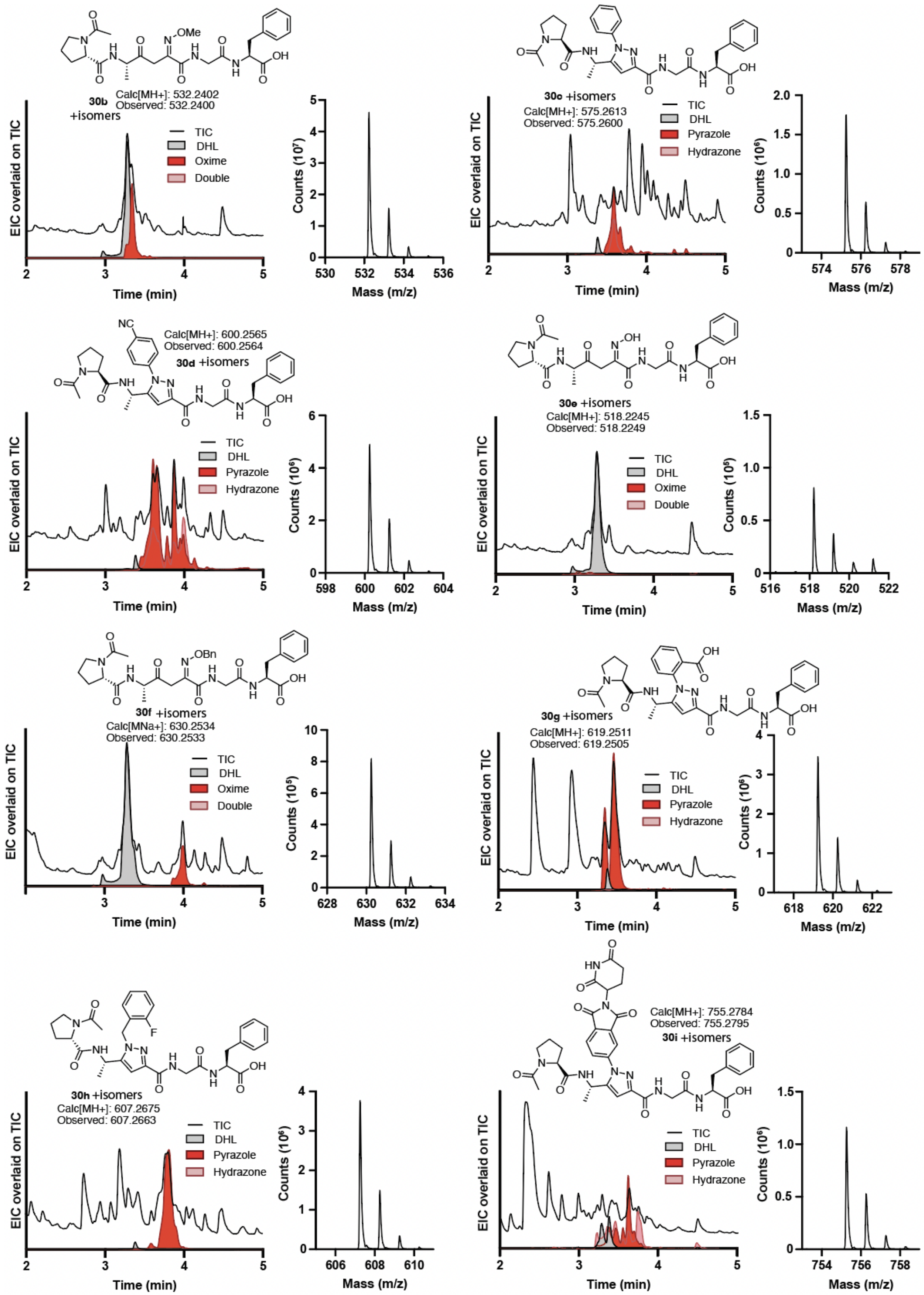
30b +isomers
Calc[MH+]: 532.2402
Observed: 532.2400
TIC
DHL
Oxime
Double
EIC overlaid on TIC
Time (min)
Counts (10^7)
Mass (m/z)
30c +isomers
Calc[MH+]: 575.2613
Observed: 575.2600
TIC
DHL
Pyrazole
Hydrazone
Counts (10^5)
30d +isomers
Calc[MH+]: 600.2565
Observed: 600.2564
Counts (10^5)
30e +isomers
Calc[MH+]: 518.2245
Observed: 518.2249
Counts (10^5)
30f +isomers
Calc[MNa+]: 630.2534
Observed: 630.2533
Counts (10^5)
30g +isomers
Calc[MH+]: 619.2511
Observed: 619.2505
Counts (10^6)
30h +isomers
Calc[MH+]: 607.2675
Observed: 607.2663
Counts (10^6)
30i +isomers
Calc[MH+]: 755.2784
Observed: 755.2795
Counts (10^6)

Figure S20. DHL-peptide **30** reacts with a variety of alpha-nucleophiles to form substituted pyrazoles and oximes. Extracted ion chromatograms (EIC) corresponding to DHL-peptide (gray) and pyrazole- or oxime-peptide (red) are overlaid on the total ion chromatogram (TIC).

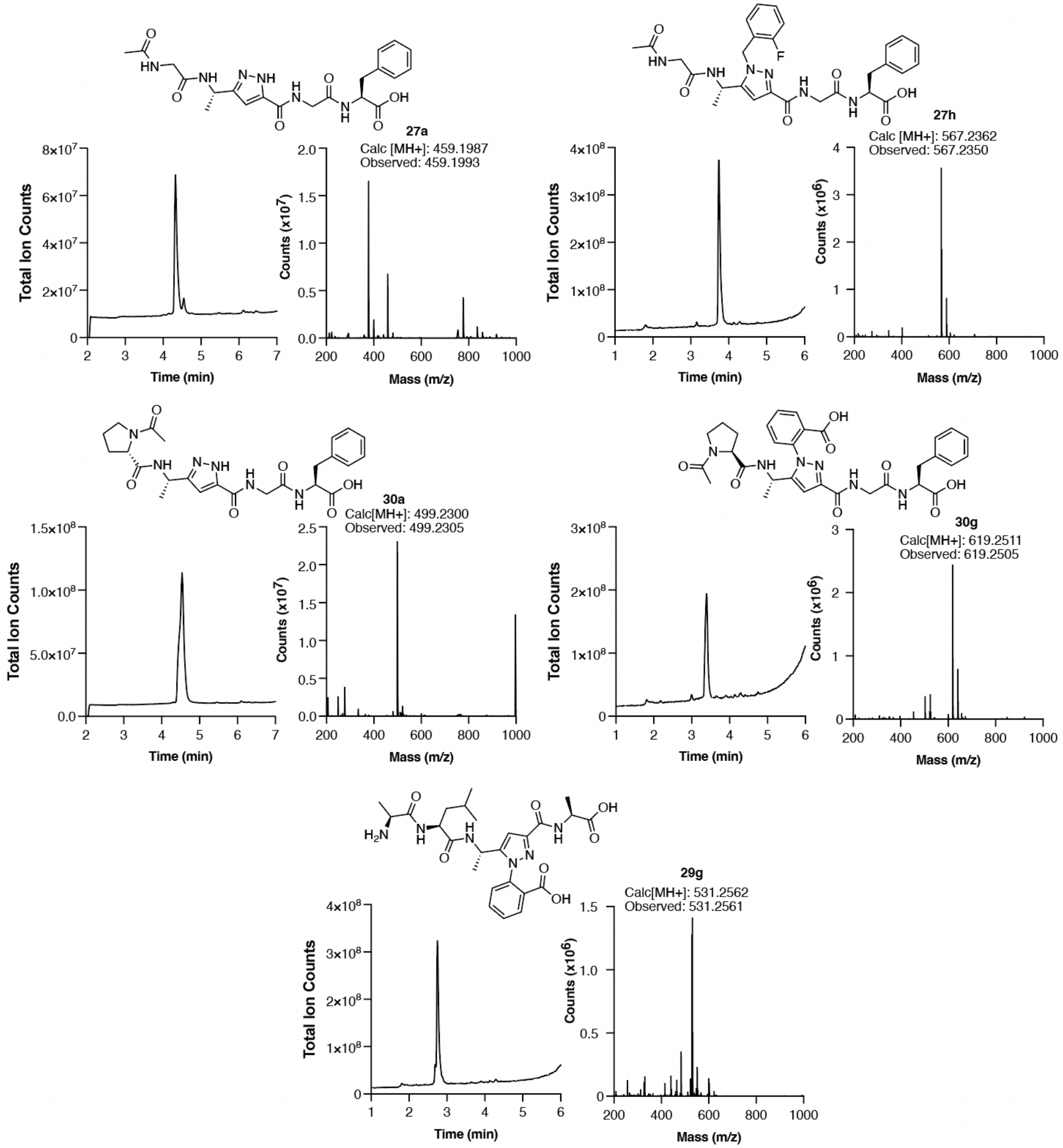

Figure S21. LC-HRMS chromatograms of pyrazole peptides isolated by RP-HPLC.

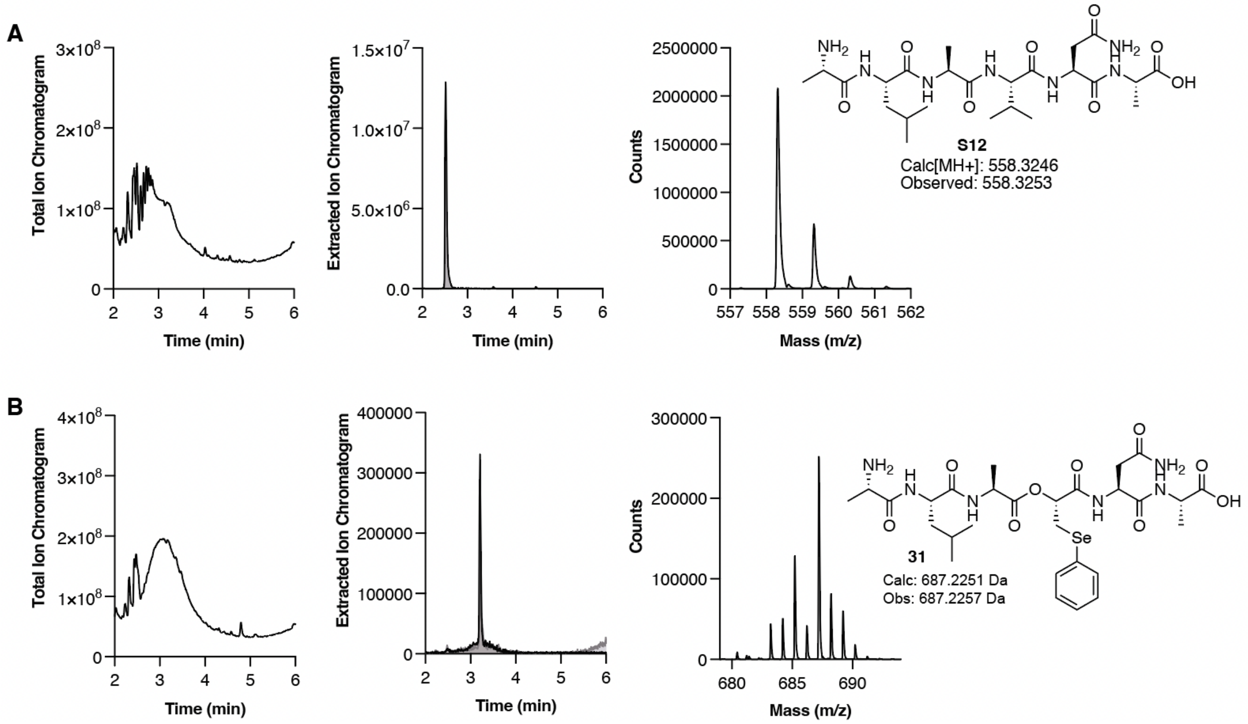

Figure S22. In vitro translation of peptide **S12** and **31**. (A) (left) Total ion chromatogram (TIC) following in vitro translation with cDNA coding for peptide MALAVNA, including Val and excluding Met. (middle) Extracted ion chromatogram (EIC) of the mass corresponding to peptide **S12**. (right) Mass spectrum of peptide **S12**. (B) (left) Total ion chromatogram (TIC) following in vitro translation with cDNA coding for peptide MALAVNA, excluding both Val and Met and adding OH-SecPH-acylated tRNA. (middle) Extracted ion chromatogram (EIC) of the mass corresponding to peptide **31**. (right) Mass spectrum of peptide **31.**

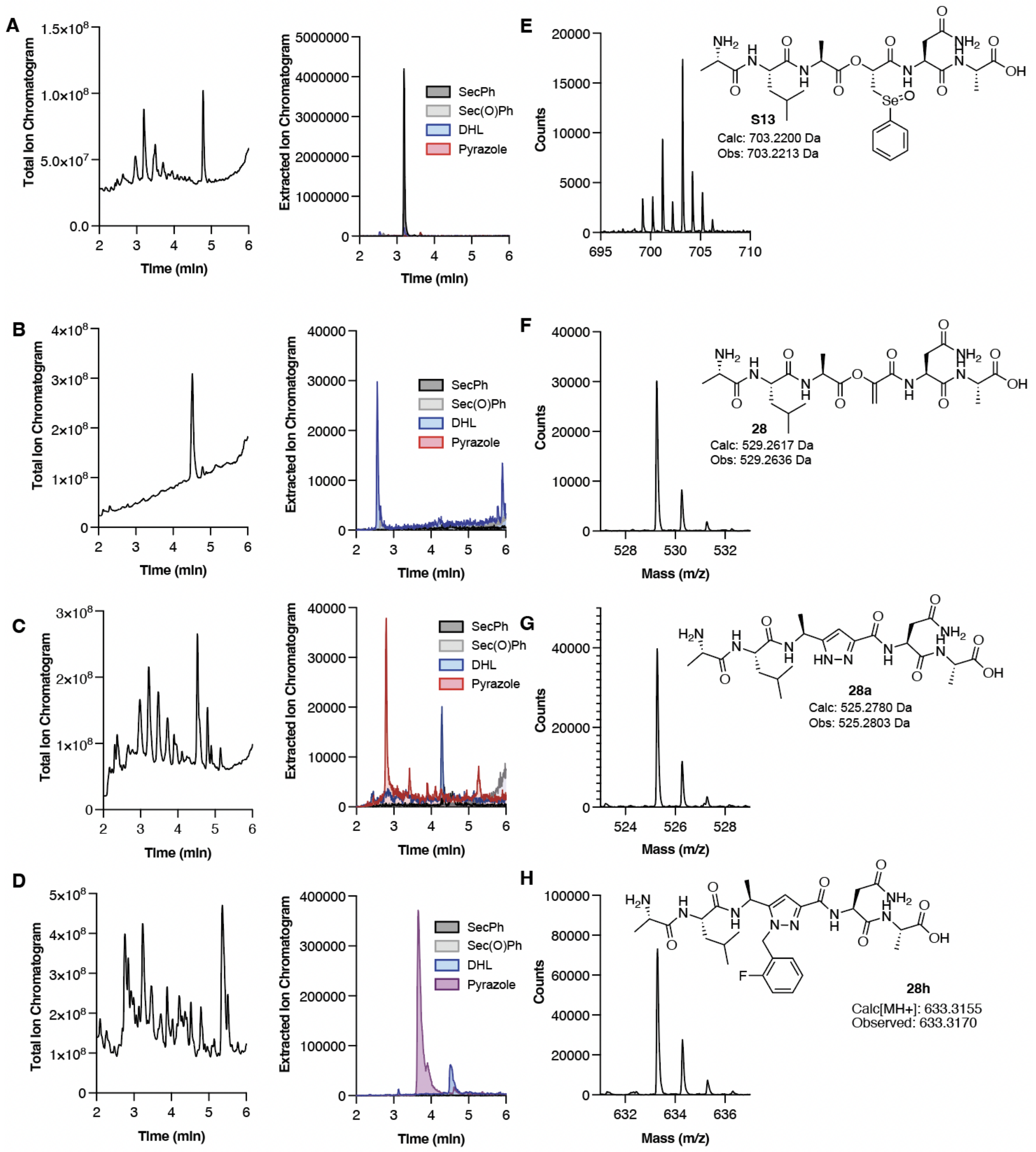

Figure S23. In vitro translation of peptide **28** and subsequent reactions. (A) Total ion chromatogram (TIC, left) and extracted ion chromatogram (EIC, right) following removal of translational machinery via MWCO filtration and desalting. (B) Total ion chromatogram (TIC, left) and extracted ion chromatogram (EIC, right) following 9 h incubation with 100 mM H_2O_2 in MeCN. (C) Total ion chromatogram (TIC, left) and extracted ion chromatogram (EIC, right) following 2 h incubation in 50 mM NaPi pH 8 followed by 15 minutes incubation in 20 mM hydrazine in 50 mM NaPi pH 6. (D) Total ion chromatogram (TIC, left) and extracted ion

chromatogram (EIC, right) following 2 hour incubation in 50 mM NaPi pH 8 followed by 15 minutes incubation in 20 mM 2-fluorobenzyl hydrazine in 50 mM NaPi pH 6. (E–H) Mass spectrum of (E) oxidized peptide **S13**, (F) DHL peptide **28**, (G) pyrazole peptide **28a**, (H) pyrazole peptide **28h**.

Supplementary Materials Section 2: Supplementary Tables

Table S1 Sequences of tRNA used in this study

Name	Sequence
tRNA Pyl	GGGGGACGGUCCGGCGACCAGCGGGUCUGACAAACCUAGCCAGCGGGGU UCGACGCCCCGGUCUCUCGCCA

Table S2 Sequences of ssDNA primers used to generate tRNA and cDNA used in this study

Name	Sequence
tRNAPyl Fwd	CTAATACGACTCACTATAGGGGGACGGTCCGGCGACCAGCGGGTCTGAC AAACCTAGCCA
tRNAPyl Rv	TGGCGAGAGACCGGGGCGTCGAACCCCGCTGGCTAGGTTTGTCAGACCC GCTGGTCGCCG
MALAVNA Fwd	GCGAATTAATACGACTCACTATAGGGTTAACTTTAACAAGGAGAAAAAC ATGGCGCTGGCGGTCAACGCG
MALAVNA Rv	AAACCCCTCCGTTTAGAGAGGGGTTATGCTAGTTACGCGTTGACCGCCA GCGCCATGTTTTTCTCCTTGT

Table S3 Sequences of dsDNA used to generate tRNA or cDNA. T7 promoter region is highlighted in blue, ribosome binding site is highlighted in red, and coding region in bold.

Name	Sequence
tRNAPyl	CTAATACGACTCACTAT**AGGGGGACGGTCCGGCGACCAGCGGGTCTGACAAACCTAGCCAGCGGGGTTCGACGCCCCGGTCTCTCGCCA**
MALAVNA	GCGAATTAATACGACTCACTATAGGGTTAACTTTAACAAGGAGAAAAAC**ATGGCGCTGGCGGTCAACGC**TAACTAGCATAACCCCTCTCTAAACGGAGGGGTTT

Supplementary Materials Section 3: Processed NMR spectra

Standard View for ^{1}H NMR is defined as 10 to -1 ppm.

Extended View for ^{1}H NMR is defined as the full collection range (14 to -2 ppm or 16 to -4 ppm).

Standard View for ^{13}C NMR is defined as 210 to -10 ppm.

Extended View for ^{13}C NMR is defined as the full collection range (235 to -15 ppm or 220 to -20 ppm).

^{1}H NMR (500 MHz, chloroform-*d*) – Standard view

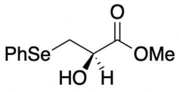

S2

Chemical Formula: $C_{10}H_{12}O_3Se$

Exact Mass: 259.9952

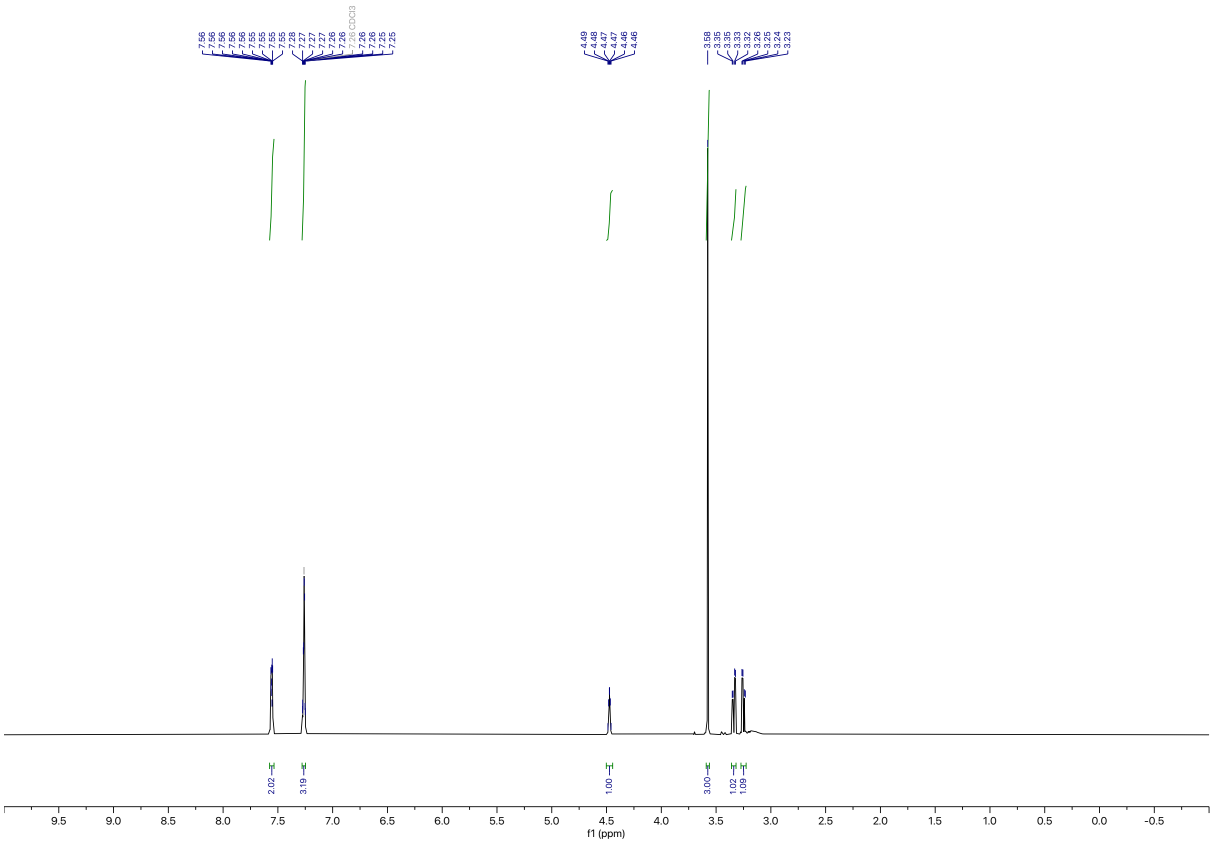

$^{13}C\{^1H\}$ NMR (125 MHz, chloroform-*d*) – Standard view

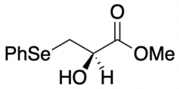

S2

Chemical Formula: $C_{10}H_{12}O_3Se$

Exact Mass: 259.9952

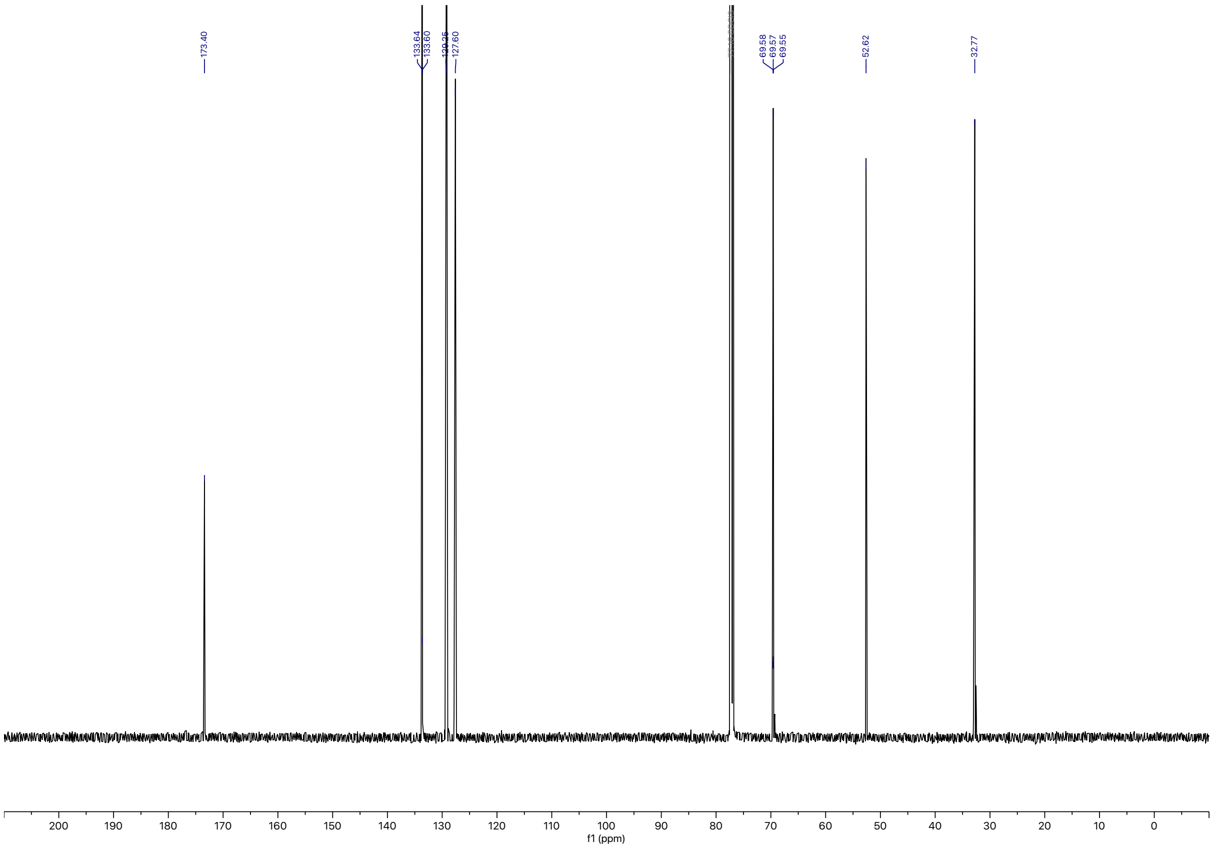

^{1}H NMR (500 MHz, chloroform-*d*) – Standard view

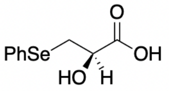

1

Chemical Formula: $C_9H_{10}O_3Se$
Exact Mass: 245.9795

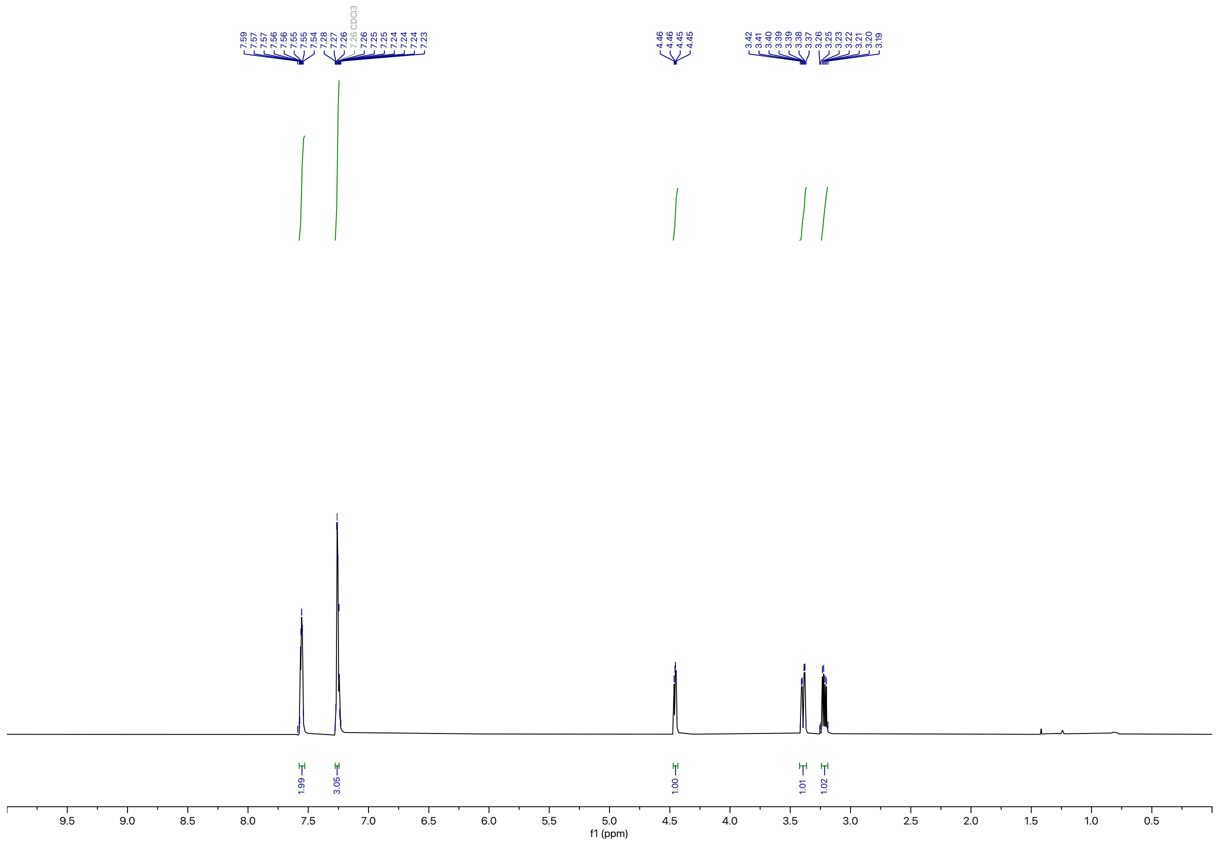

$^{13}C\{^{1}H\}$ NMR (125 MHz, chloroform-*d*) – Standard view

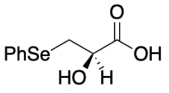

1

Chemical Formula: $C_9H_{10}O_3Se$
Exact Mass: 245.9795

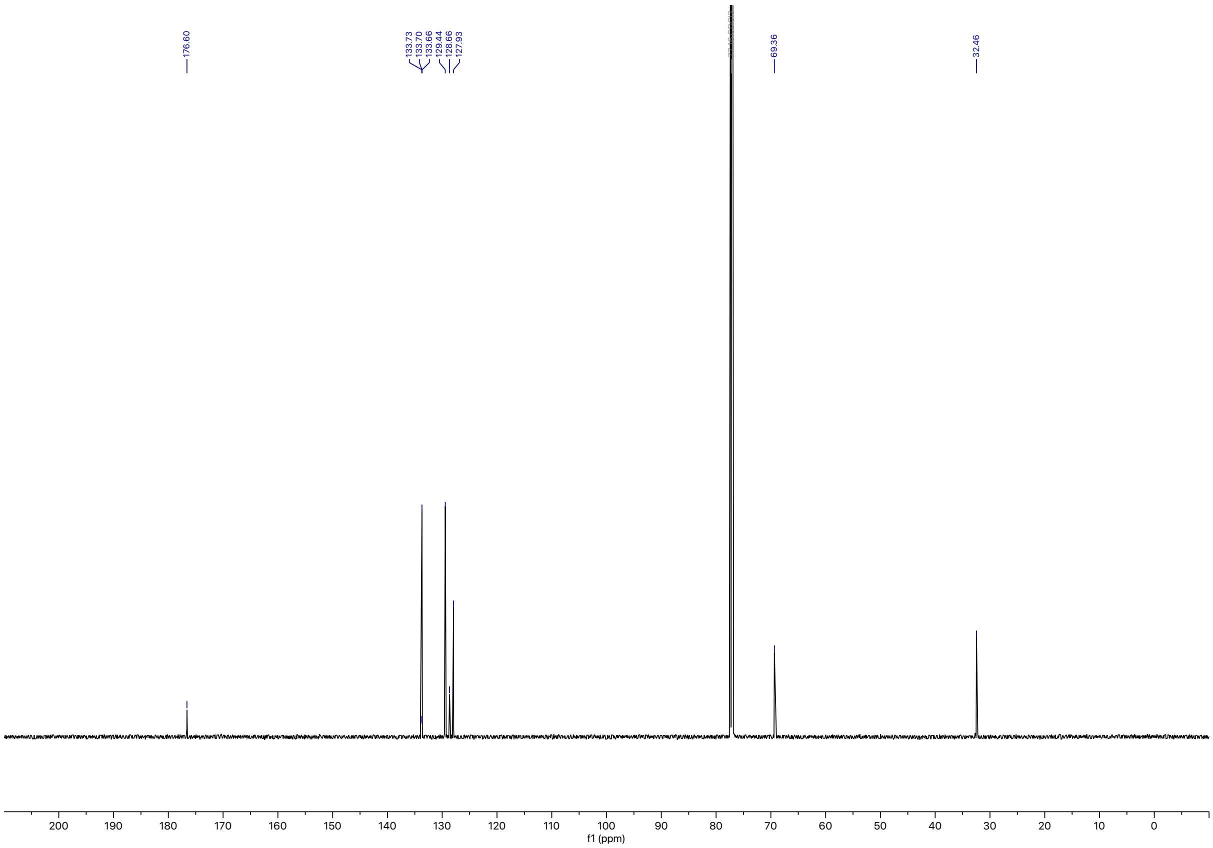

^{1}H NMR (500 MHz, chloroform-*d*) – Standard view

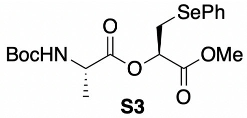

S3

Chemical Formula: $C_{18}H_{25}NO_6Se$
Exact Mass: 431.08

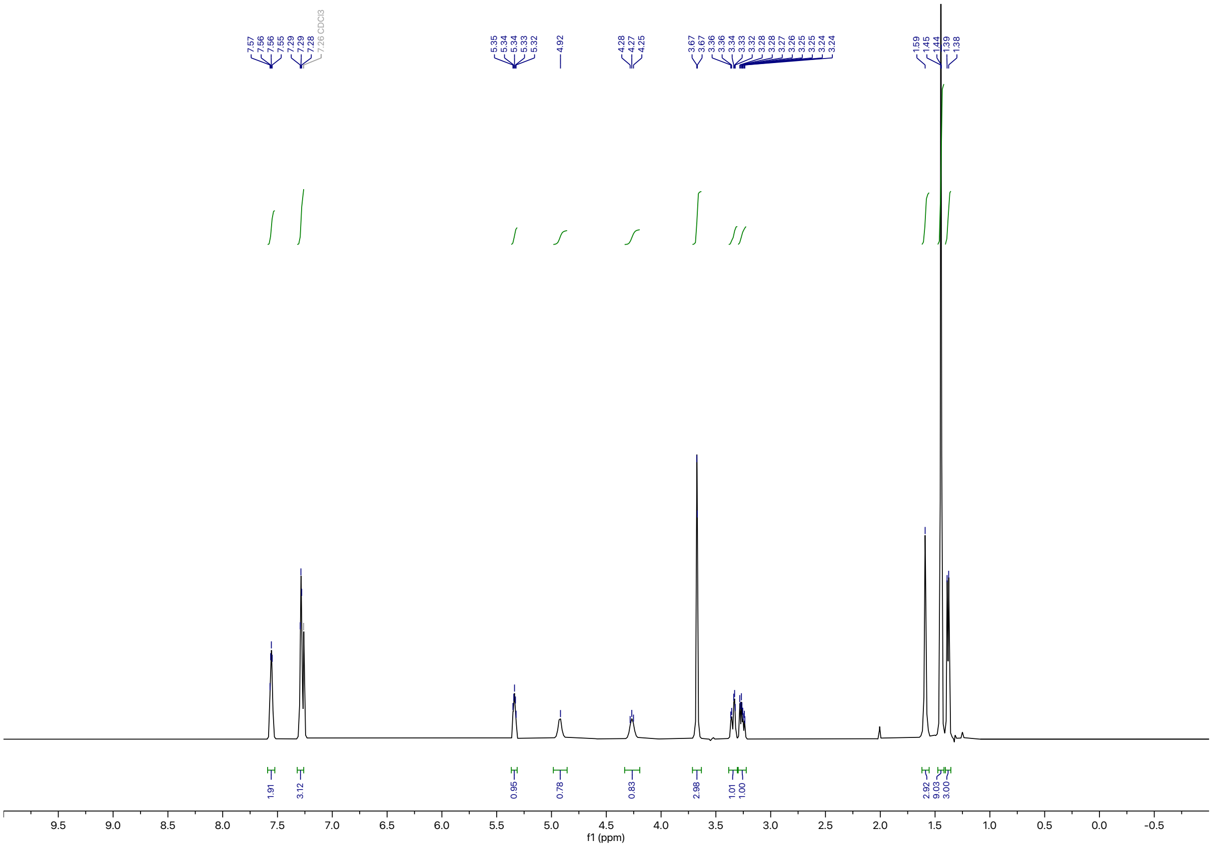

$^{13}C\{^{1}H\}$ NMR (125 MHz, chloroform-*d*) – Standard view

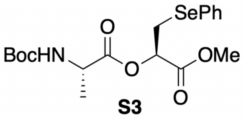

S3

Chemical Formula: $C_{18}H_{25}NO_6Se$
Exact Mass: 431.08

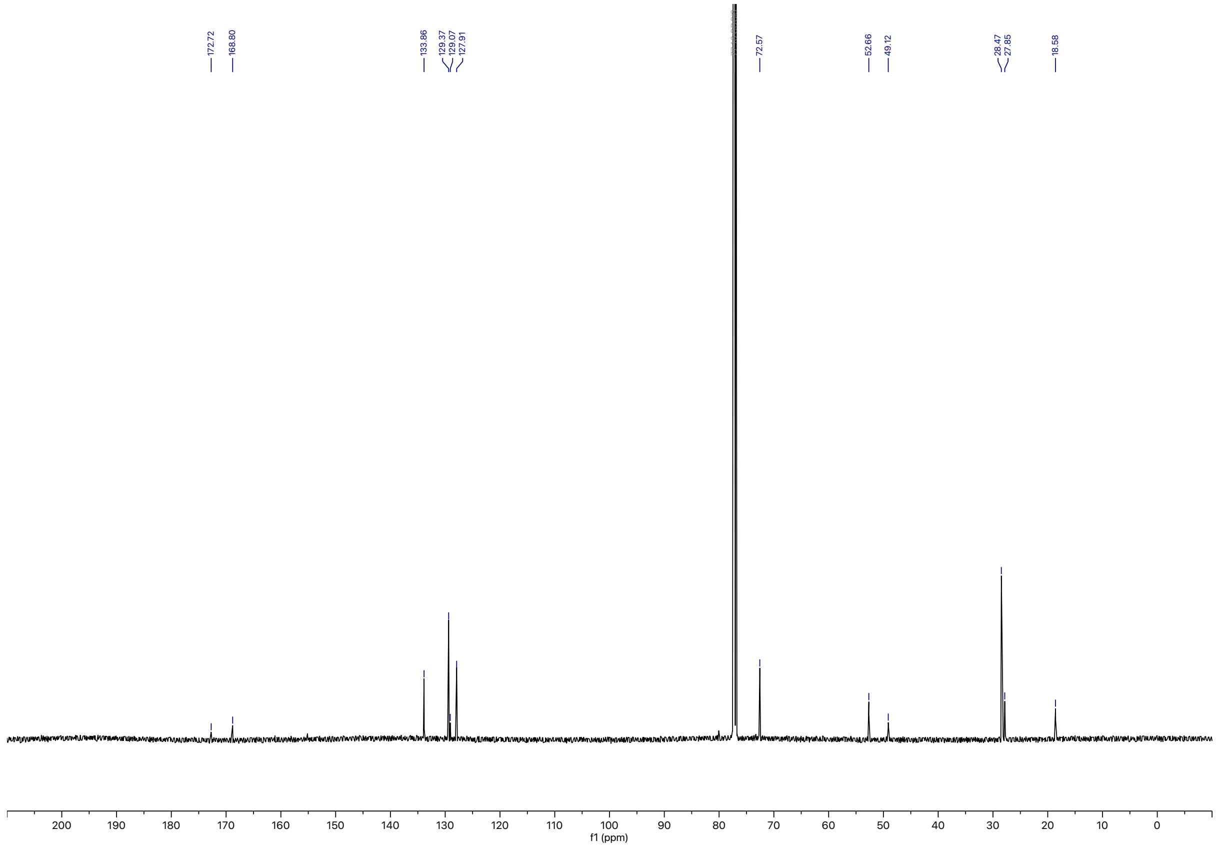

^{1}H NMR (500 MHz, chloroform-*d*) – Standard view

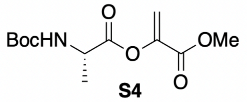

S4

Chemical Formula: $C_{12}H_{19}NO_6$
Exact Mass: 273.12

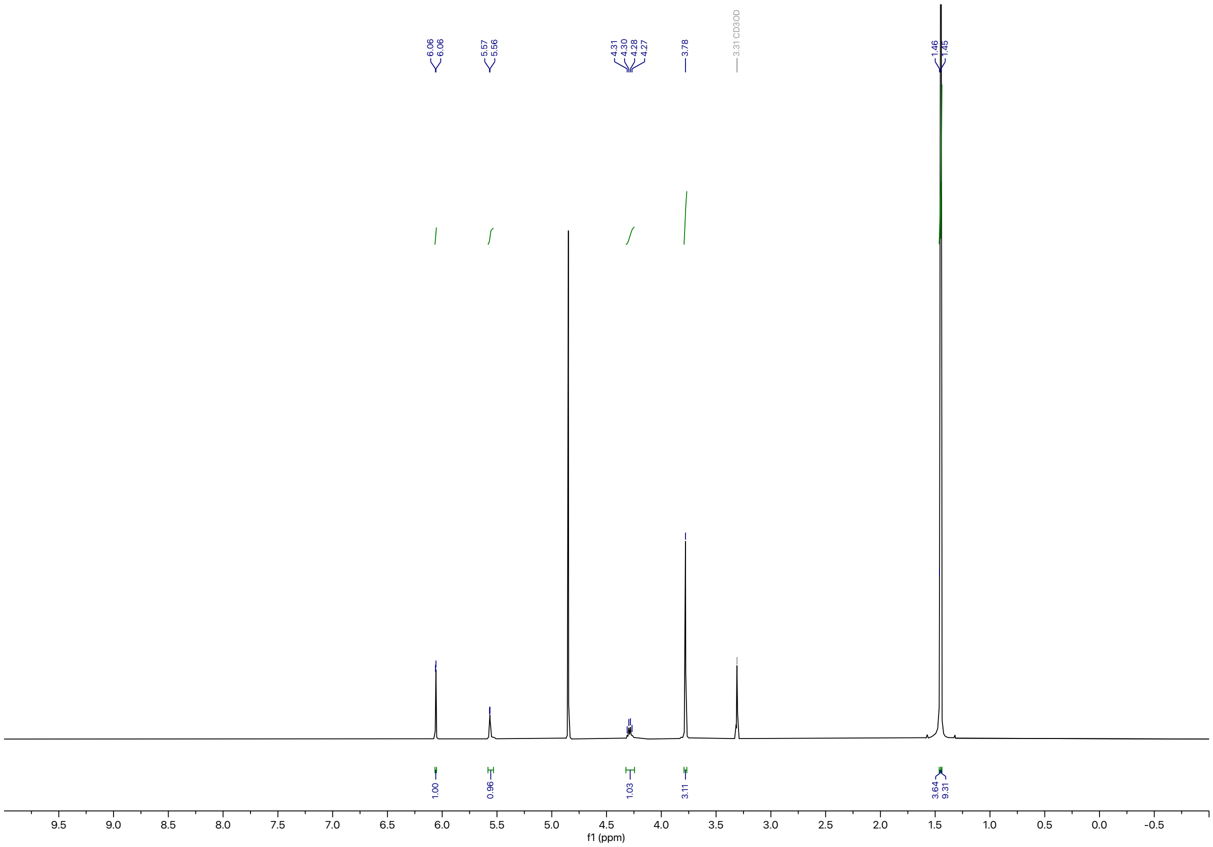

$^{13}C\{^{1}H\}$ NMR (125 MHz, chloroform-*d*) – Standard view

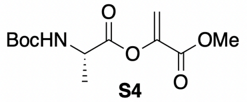

S4

Chemical Formula: $C_{12}H_{19}NO_6$

Exact Mass: 273.12

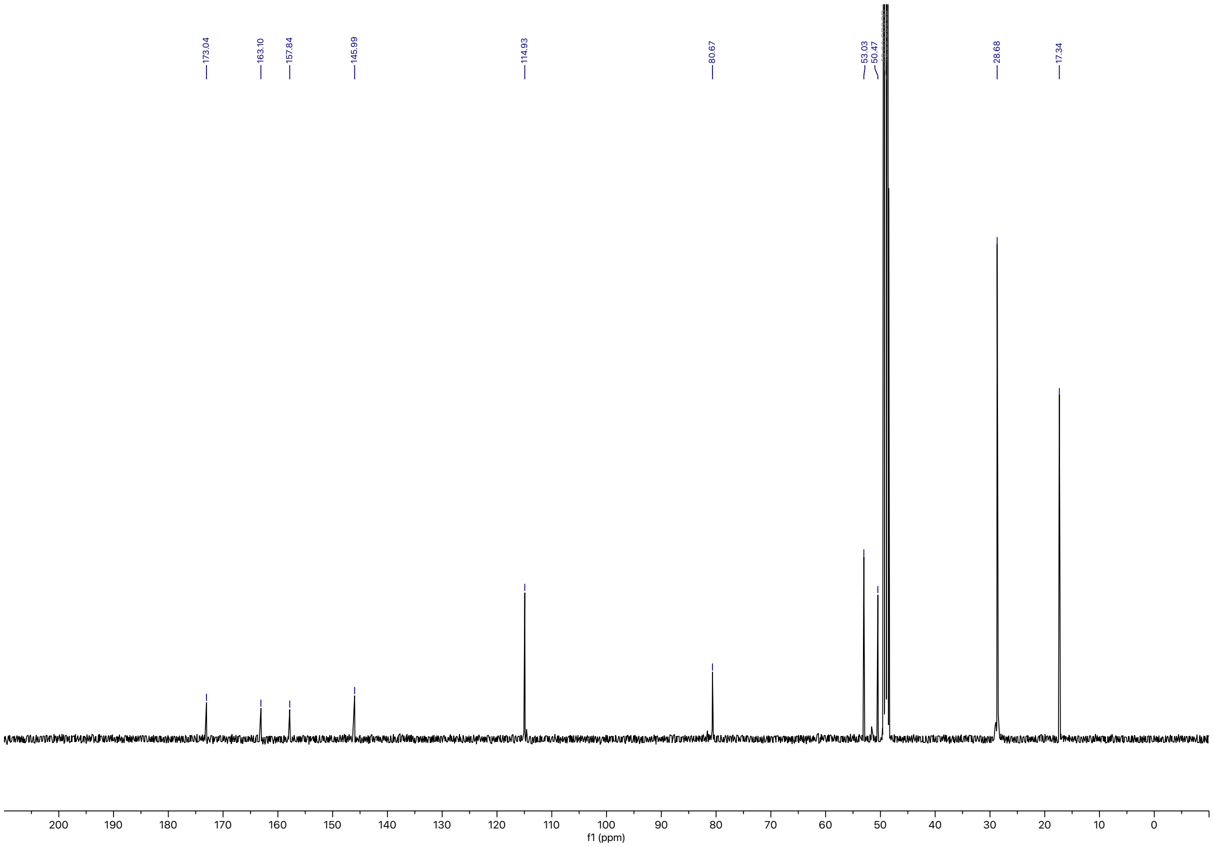

^{1}H NMR (500 MHz, chloroform-*d*) – Standard view

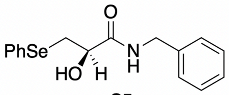

S5

Chemical Formula: $C_{16}H_{17}NO_2Se$
Exact Mass: 335.0425

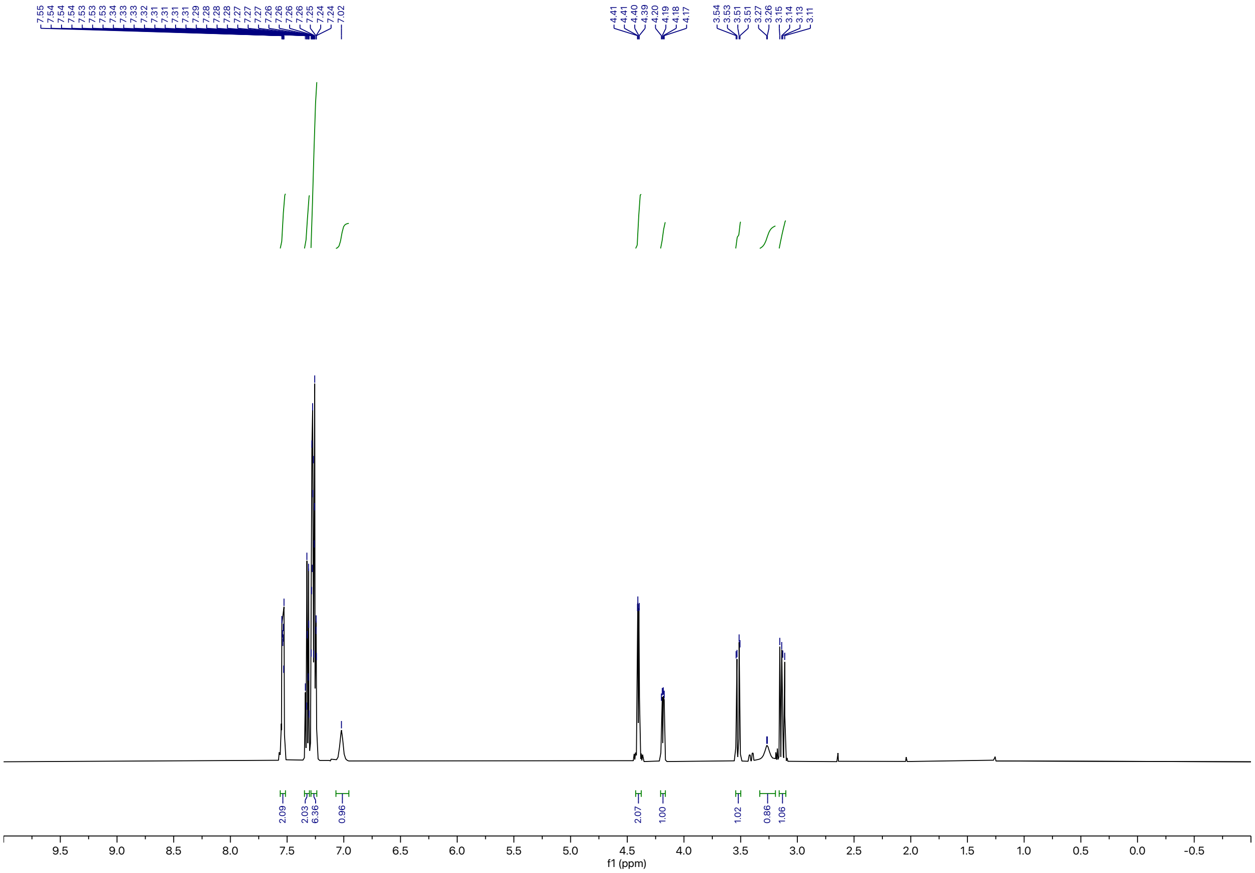

$^{13}C\{^{1}H\}$ NMR (125 MHz, chloroform-*d*) – Standard view

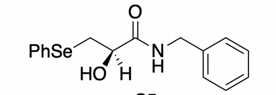

S5

Chemical Formula: $C_{16}H_{17}NO_2Se$
Exact Mass: 335.0425

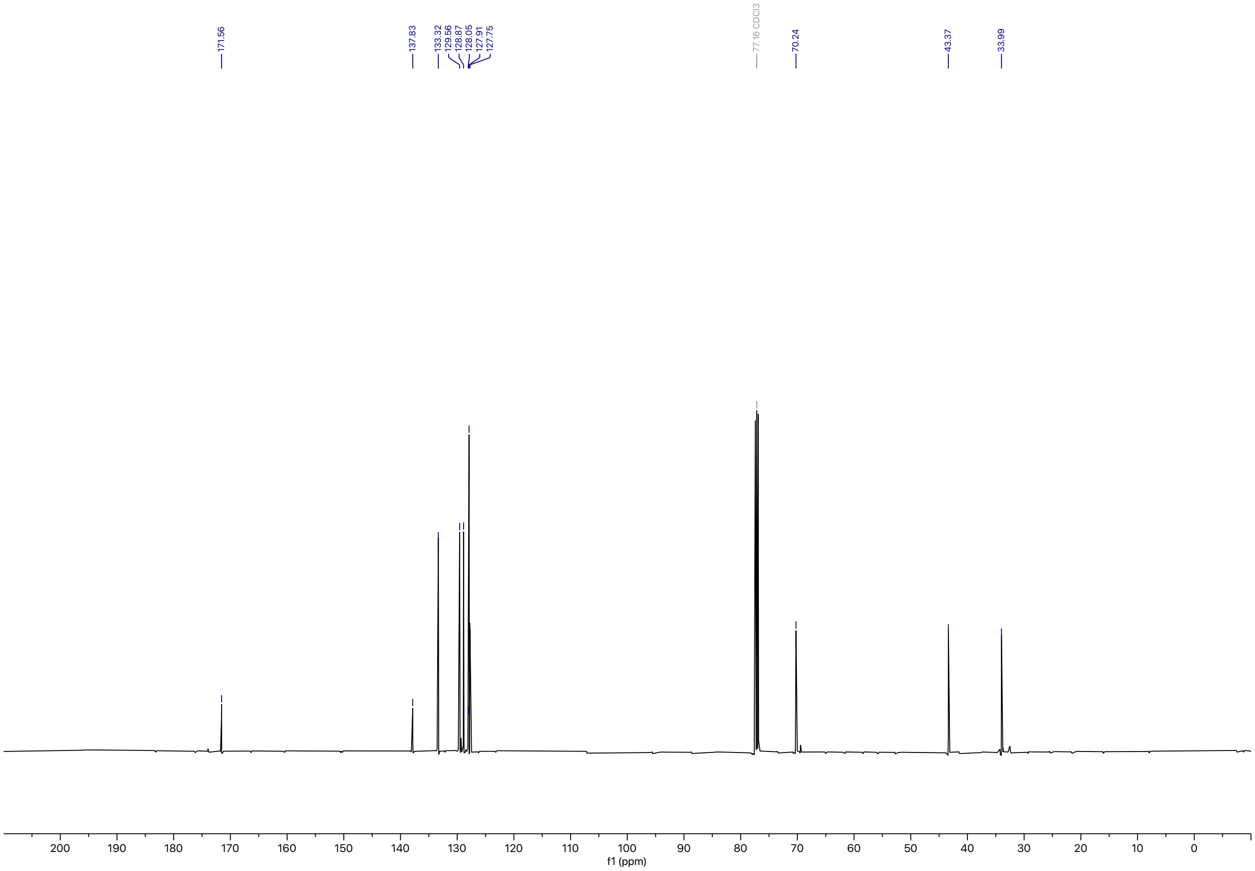

^{1}H NMR (500 MHz, chloroform-*d*) – Standard view

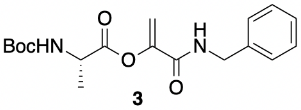

3

Chemical Formula: $C_{18}H_{24}N_2O_5$
Exact Mass: 348.1685

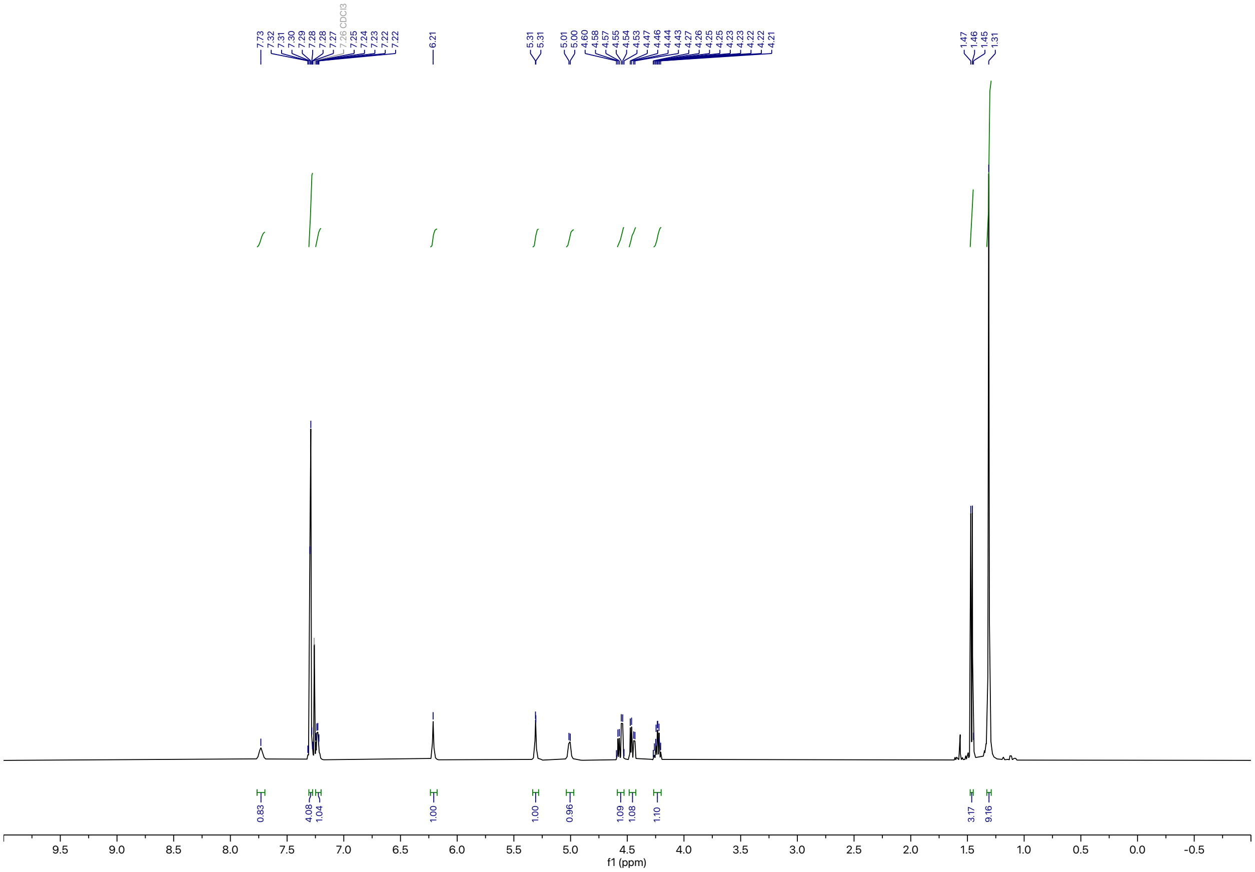

$^{13}C\{^1H\}$ NMR (125 MHz, chloroform-*d*) – Standard view

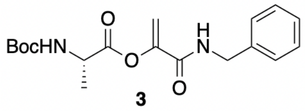

Chemical Formula: $C_{18}H_{24}N_2O_5$
Exact Mass: 348.1685

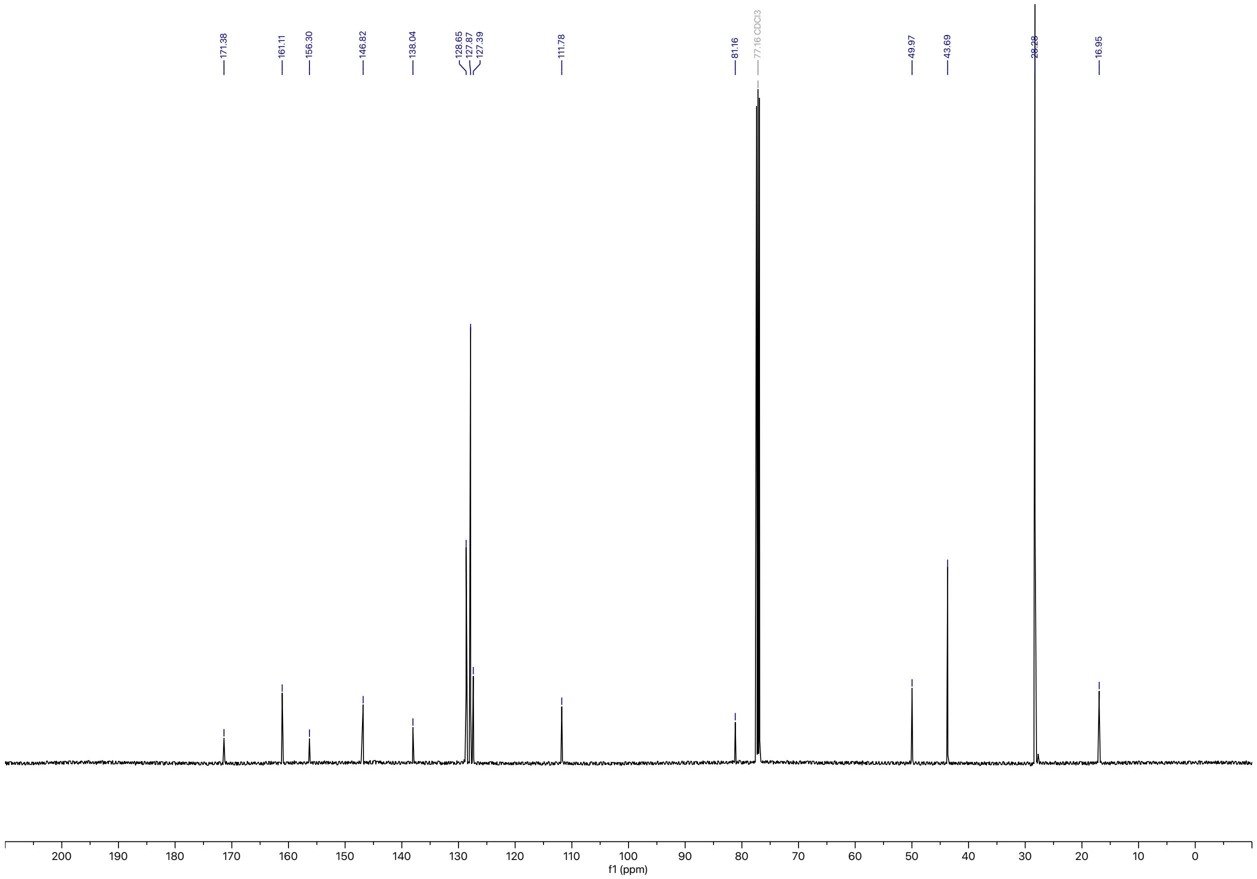

^{1}H NMR (500 MHz, chloroform-*d*) – Standard view

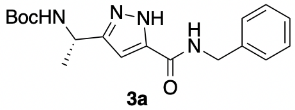

3a

Chemical Formula: $C_{18}H_{24}N_4O_3$
Exact Mass: 344.1848

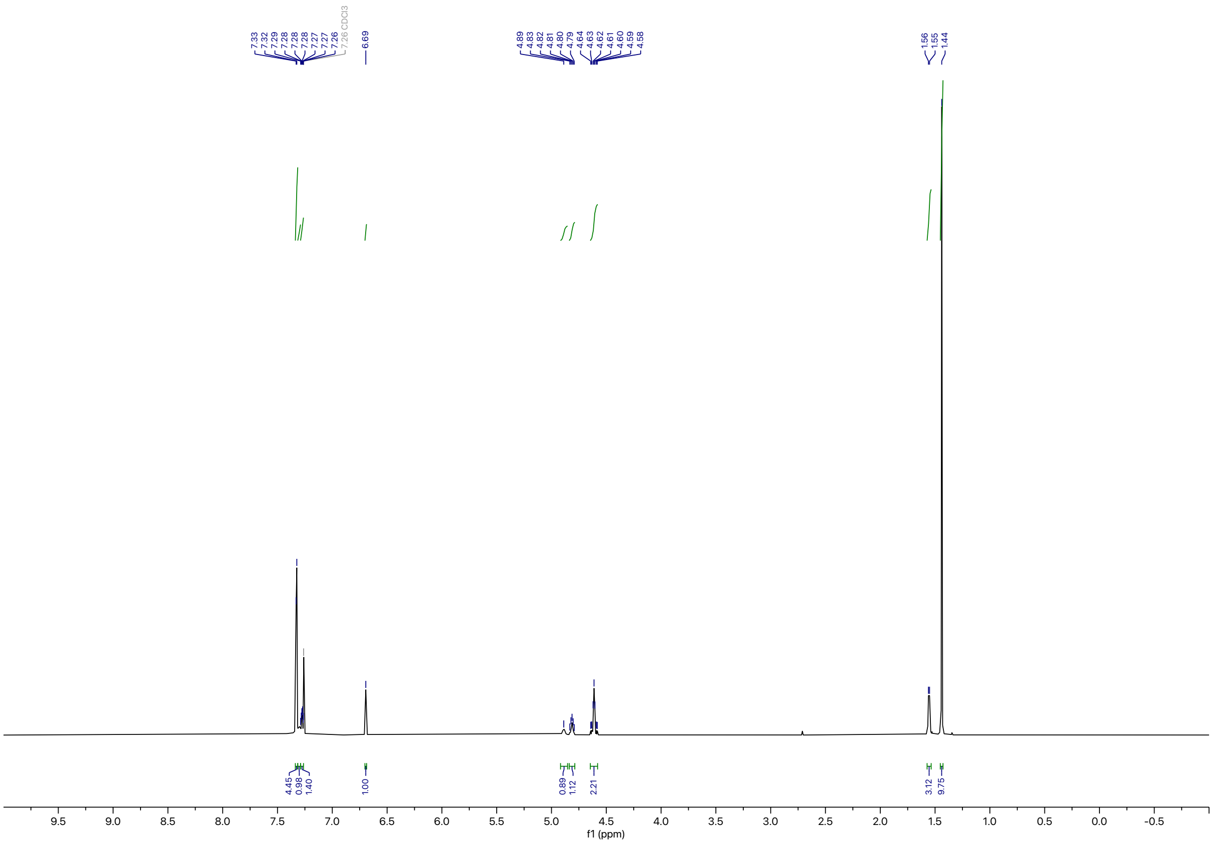

$^{13}C\{^1H\}$ NMR (125 MHz, chloroform-*d*) – Standard view

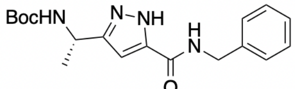

3a

Chemical Formula: $C_{18}H_{24}N_4O_3$
Exact Mass: 344.1848

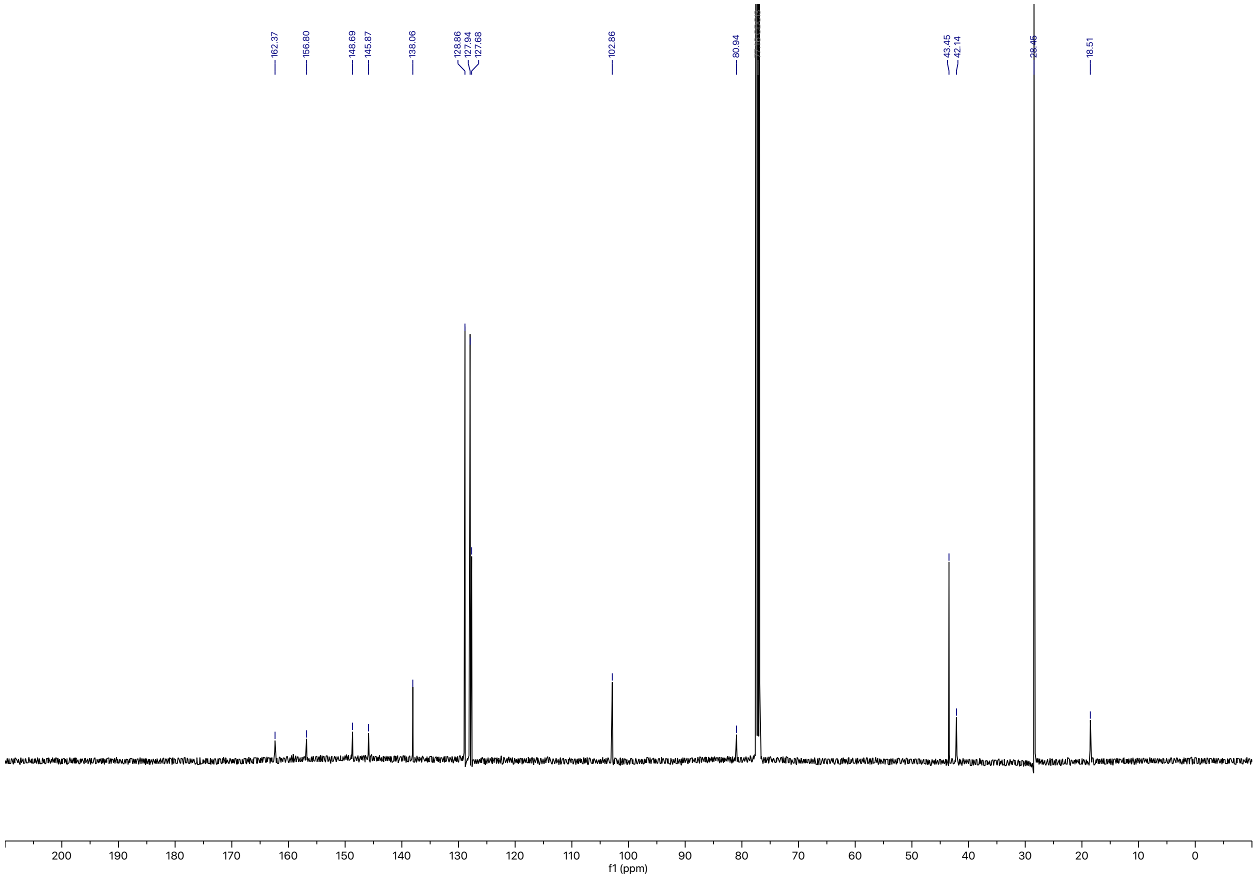

HMBC NMR (125 MHz, chloroform-*d*) – Standard view

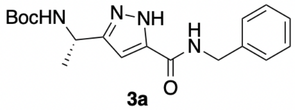

3a

Chemical Formula: $C_{18}H_{24}N_4O_3$
Exact Mass: 344.1848

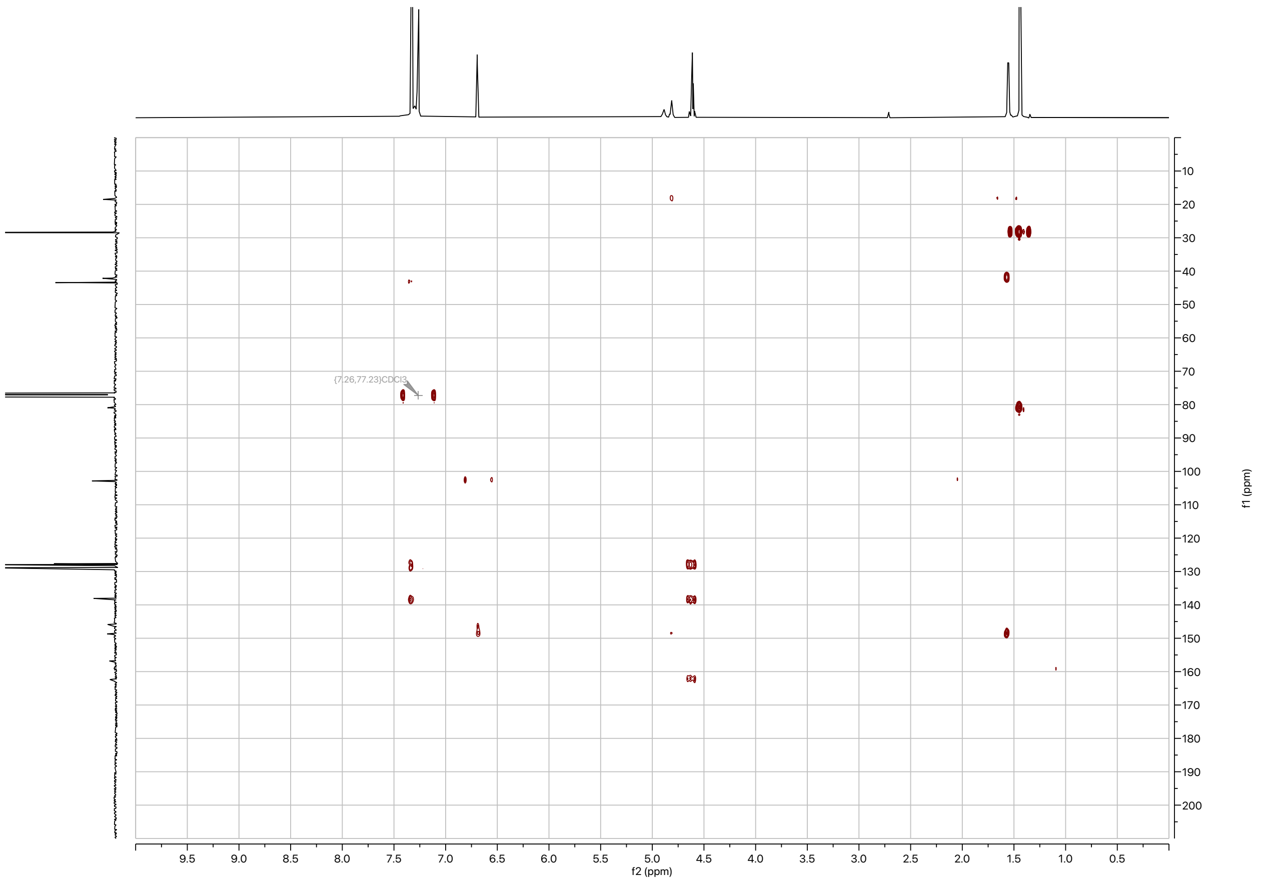

Multiplicity-edited HSQC NMR (125 MHz, chloroform-*d*) – Standard view

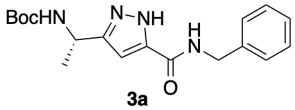

Chemical Formula: $C_{18}H_{24}N_4O_3$
Exact Mass: 344.1848

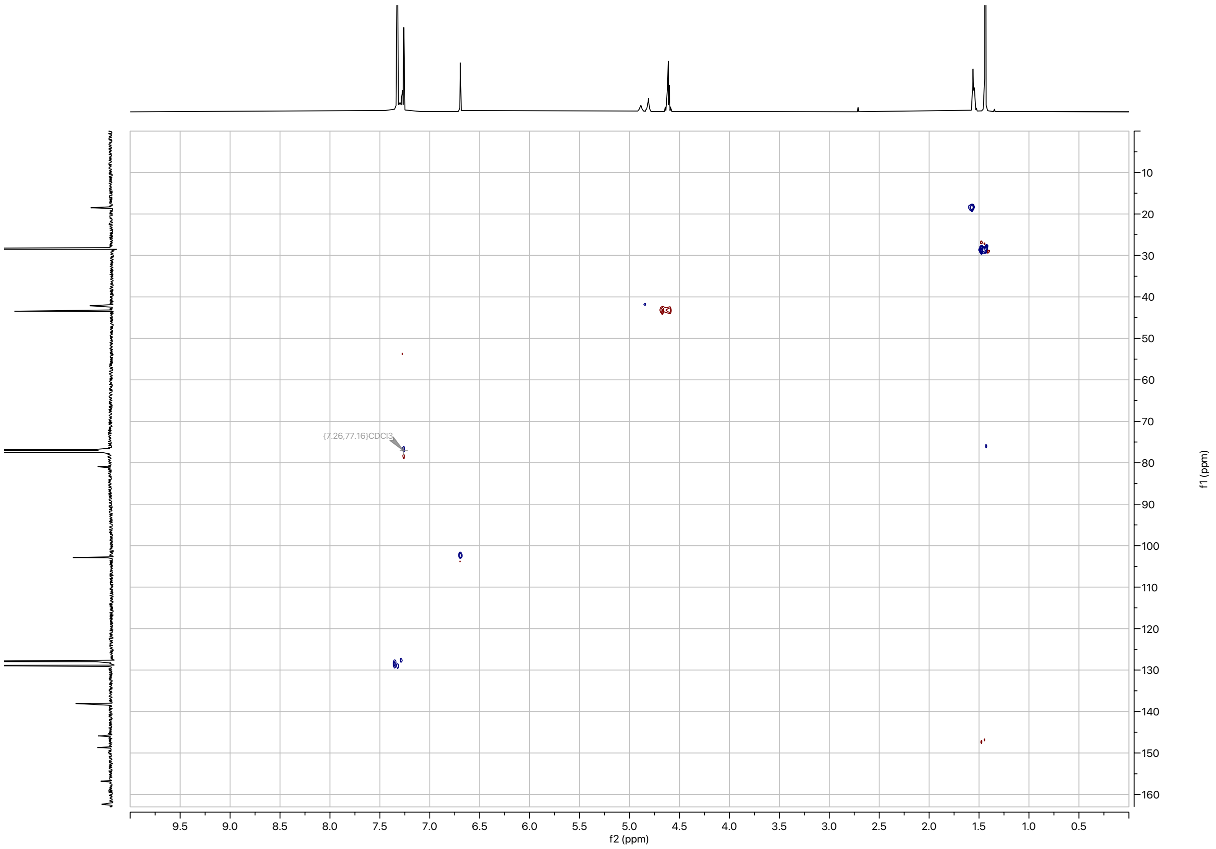

COSY NMR (125 MHz, chloroform-*d*) – Standard view

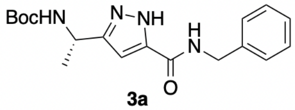

3a

Chemical Formula: $C_{18}H_{24}N_4O_3$
Exact Mass: 344.1848

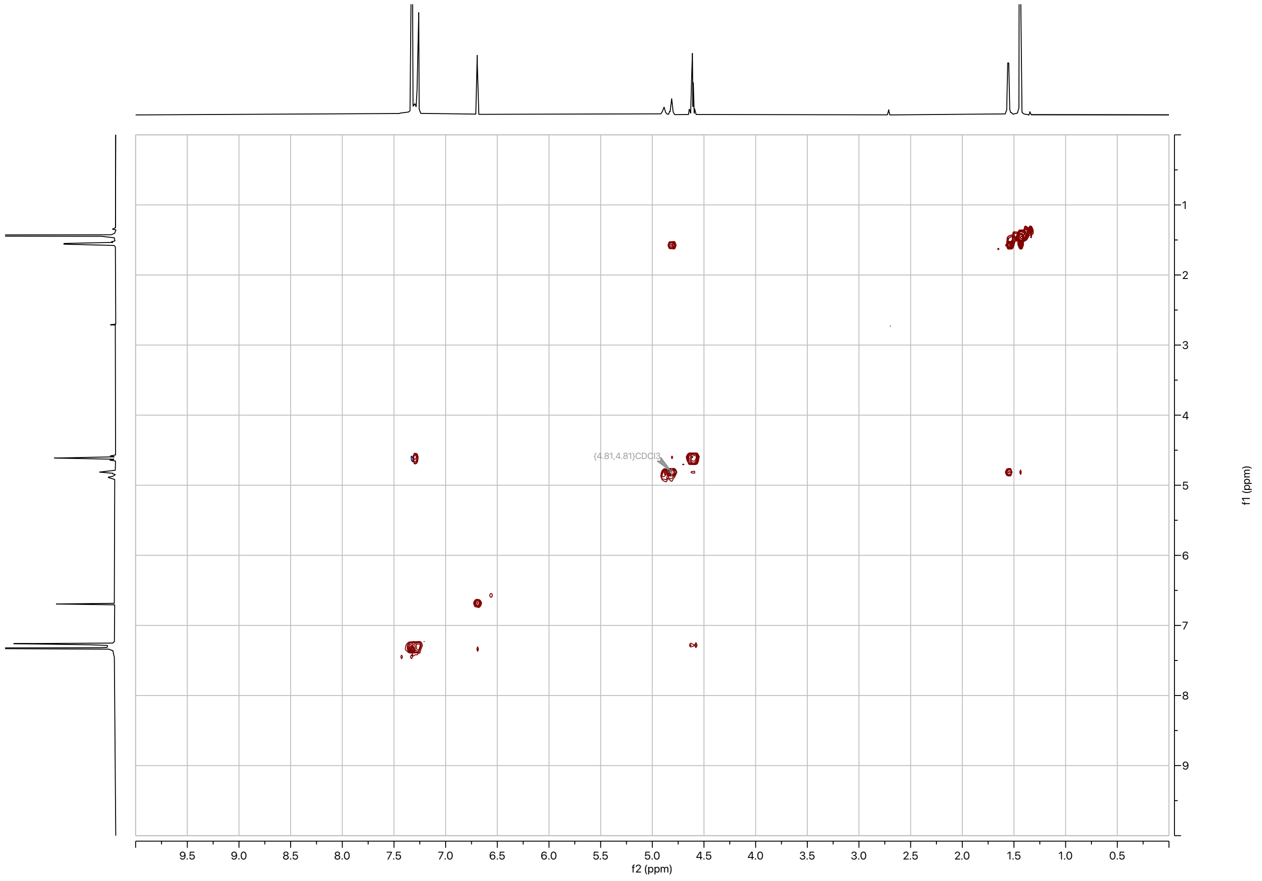

^{1}H NMR (500 MHz, chloroform-*d*) – Standard view

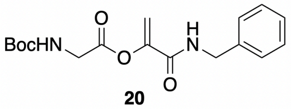

20

Chemical Formula: $C_{17}H_{22}N_2O_5$
Exact Mass: 334.1529

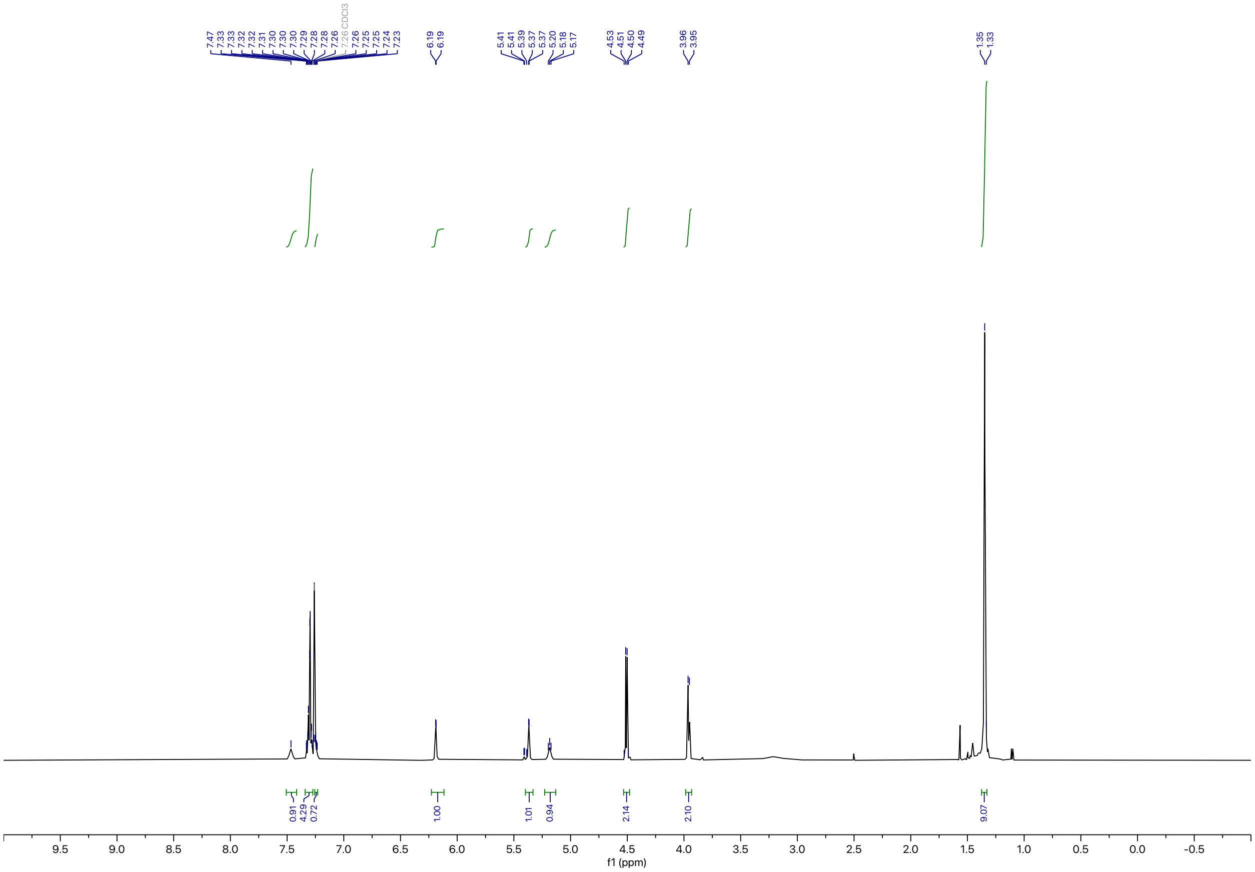

$^{13}C\{^1H\}$ NMR (125 MHz, chloroform-*d*) – Standard view

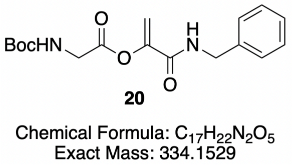

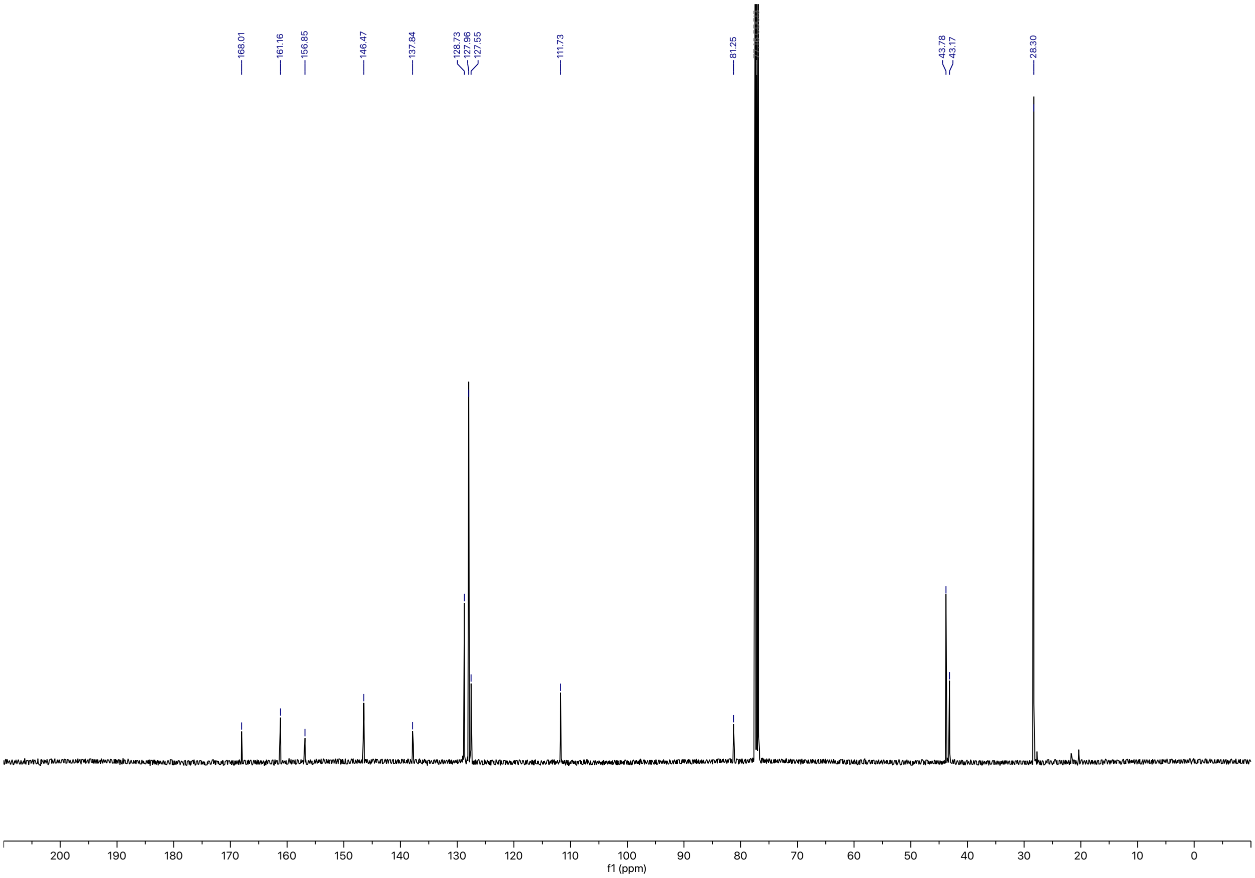

^{1}H NMR (500 MHz, chloroform-*d*) – Standard view

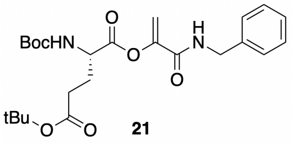

21

Chemical Formula: $C_{24}H_{34}N_2O_7$

Exact Mass: 462.2366

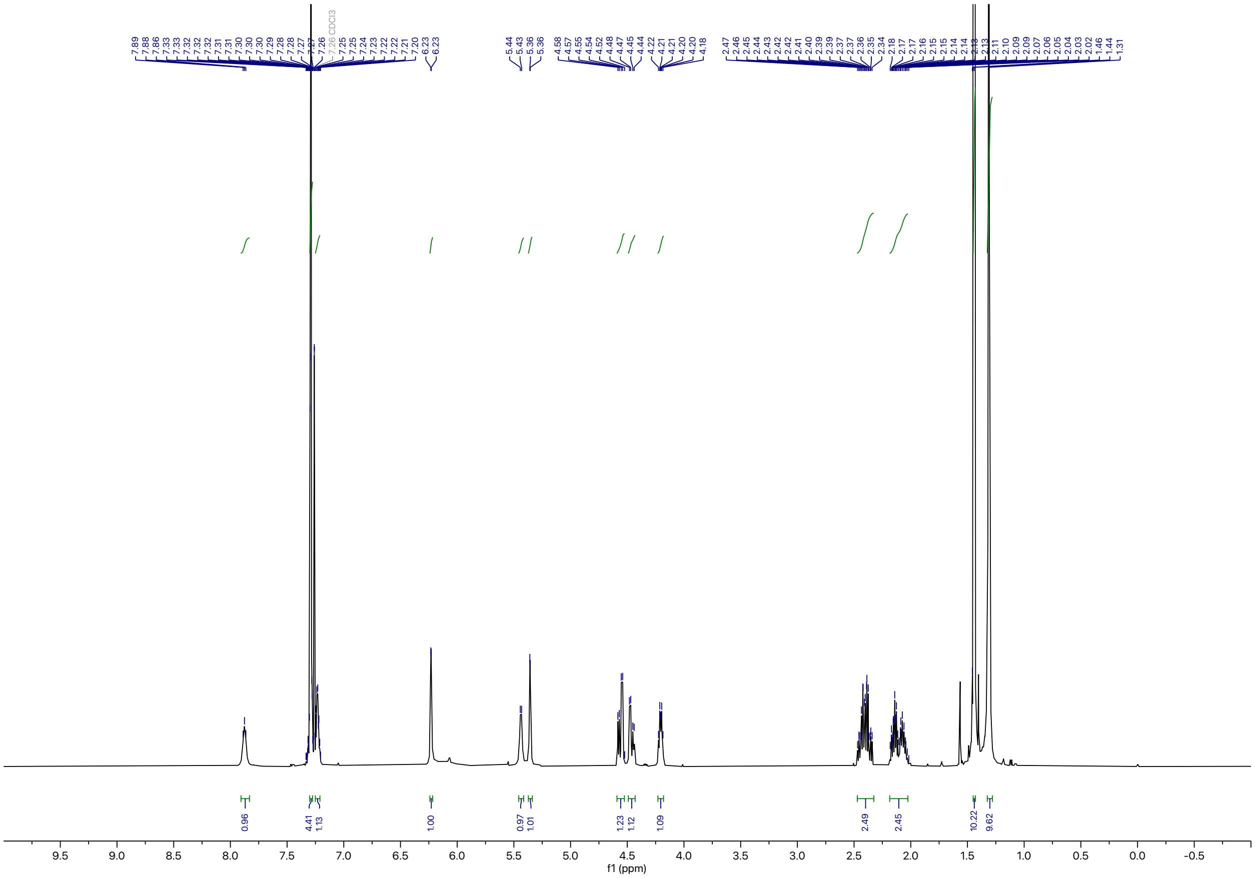

$^{13}C\{^1H\}$ NMR (125 MHz, chloroform-*d*) – Standard view

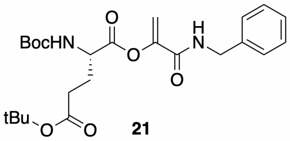

21

Chemical Formula: $C_{24}H_{34}N_2O_7$
Exact Mass: 462.2366

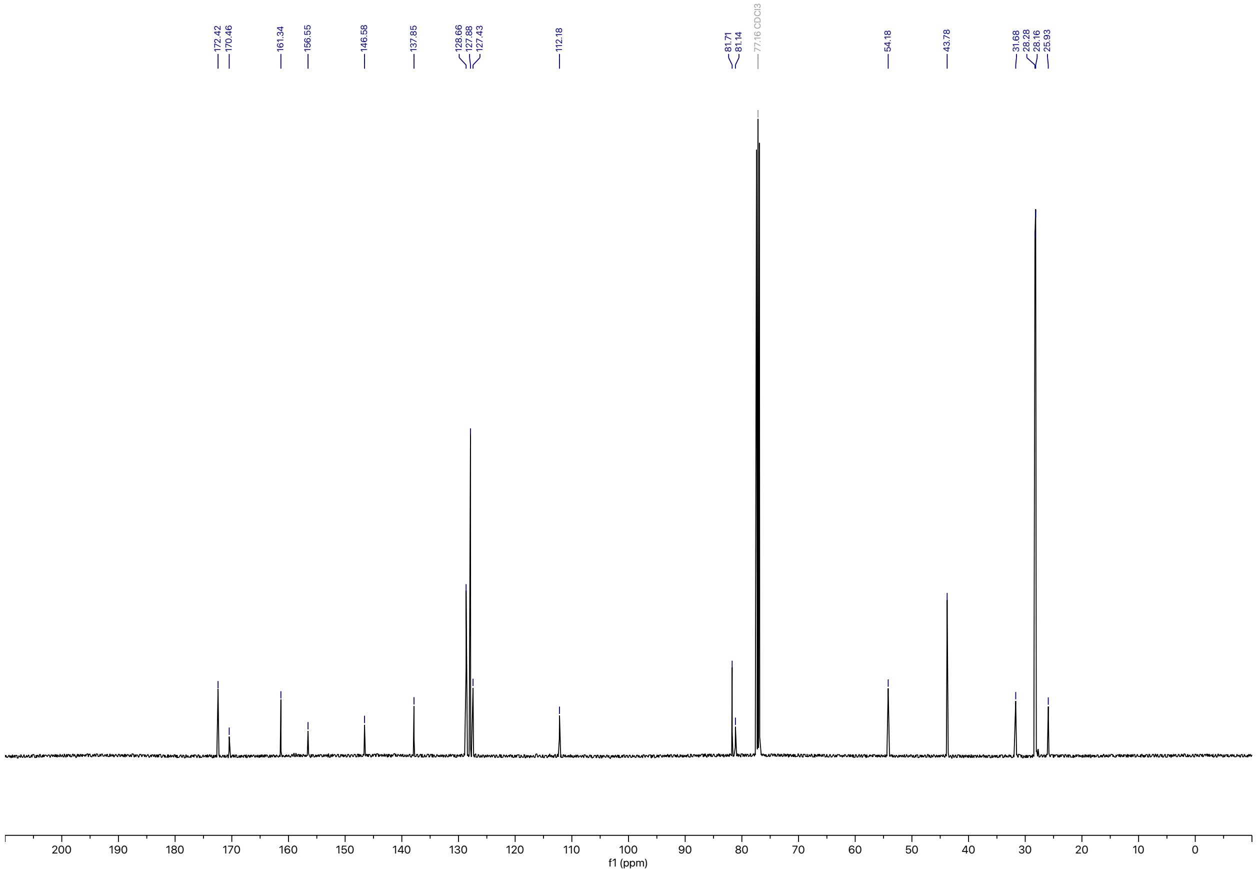

^{1}H NMR (500 MHz, chloroform-*d*) – Standard view

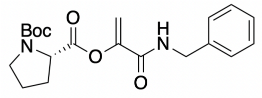

22

Chemical Formula: $C_{20}H_{26}N_2O_5$

Exact Mass: 374.1842

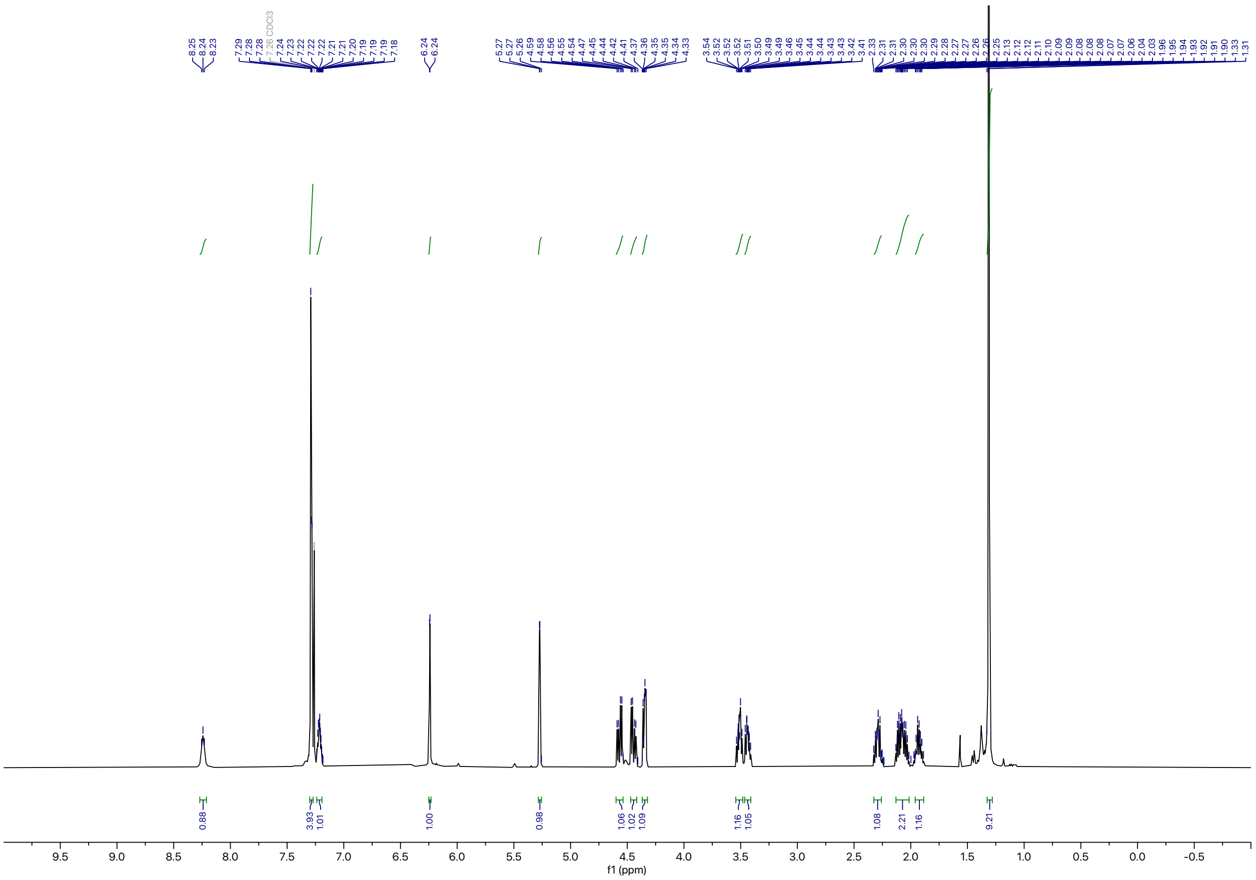

$^{13}C\{^{1}H\}$ NMR (125 MHz, chloroform-*d*) – Standard view

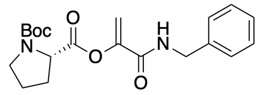

22

Chemical Formula: $C_{20}H_{26}N_2O_5$

Exact Mass: 374.1842

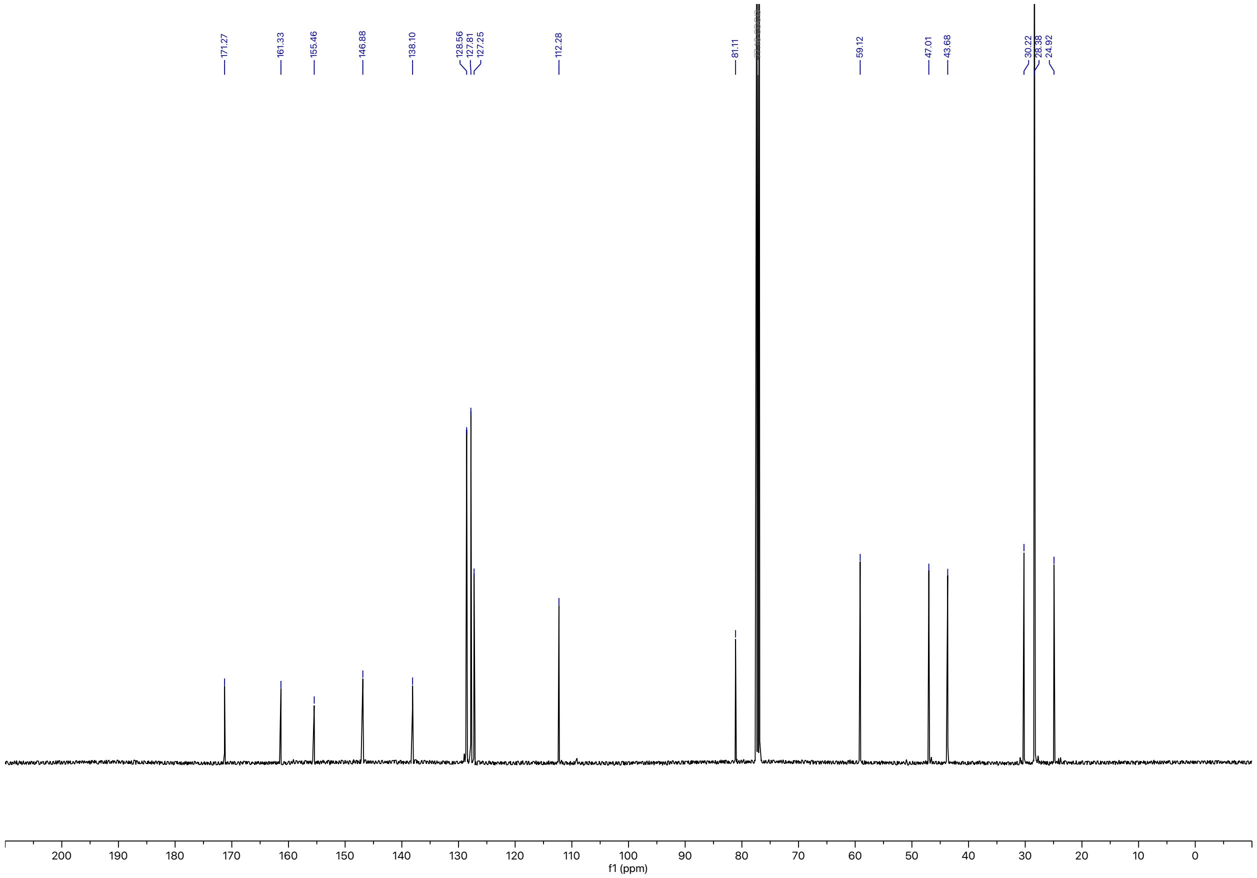

^{1}H NMR (500 MHz, chloroform-*d*) – Standard view

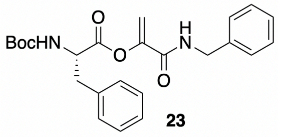

23

Chemical Formula: $C_{24}H_{28}N_2O_5$
Exact Mass: 424.1998

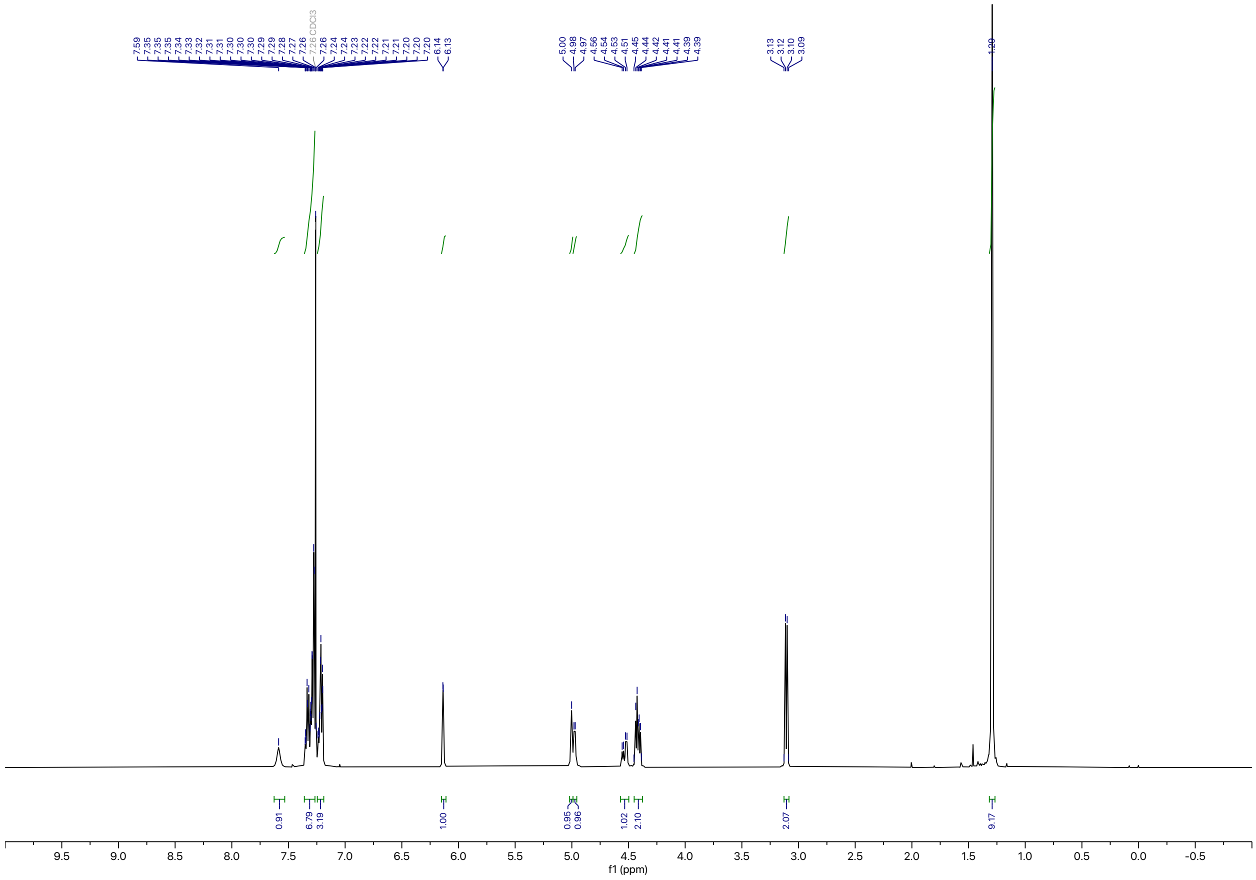

$^{13}C\{^1H\}$ NMR (125 MHz, chloroform-*d*) – Standard view

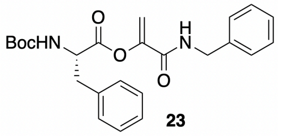

23

Chemical Formula: $C_{24}H_{28}N_2O_5$
Exact Mass: 424.1998

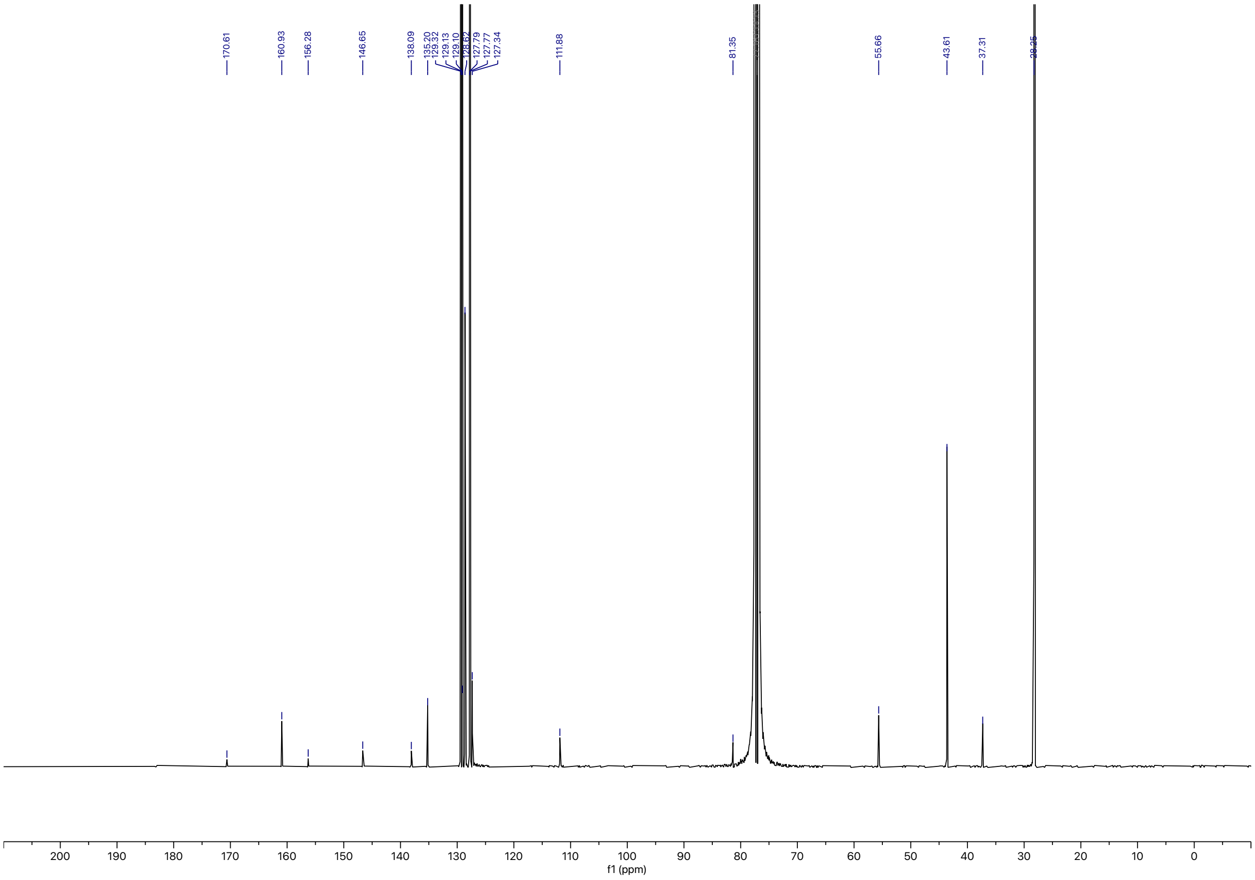

^{1}H NMR (500 MHz, chloroform-*d*) – Standard view

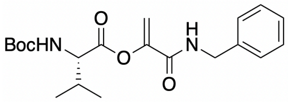

24

Chemical Formula: $C_{20}H_{28}N_2O_5$
Exact Mass: 376.1998

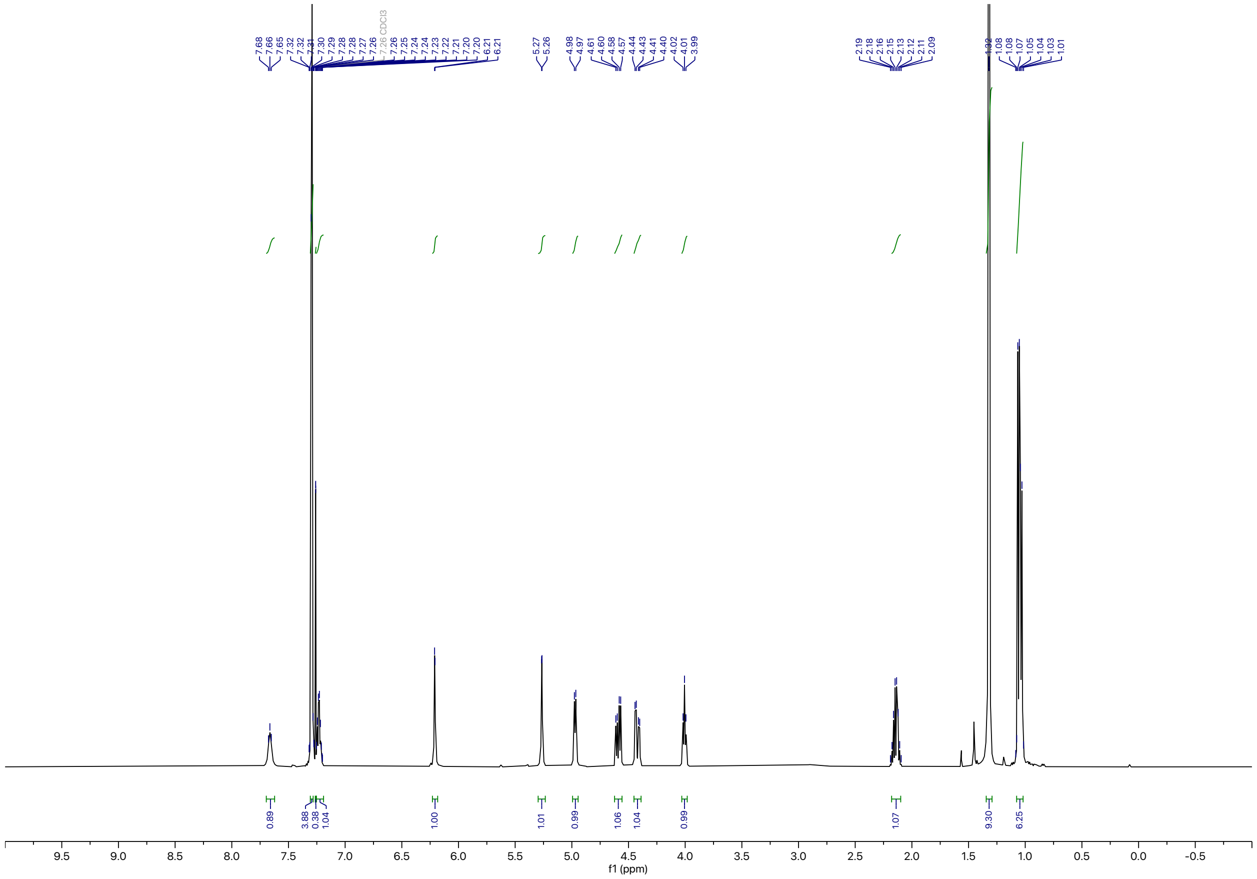

$^{13}C\{^{1}H\}$ NMR (125 MHz, chloroform-*d*) – Standard view

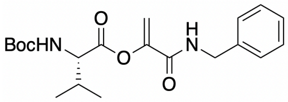

24

Chemical Formula: $C_{20}H_{28}N_2O_5$
Exact Mass: 376.1998

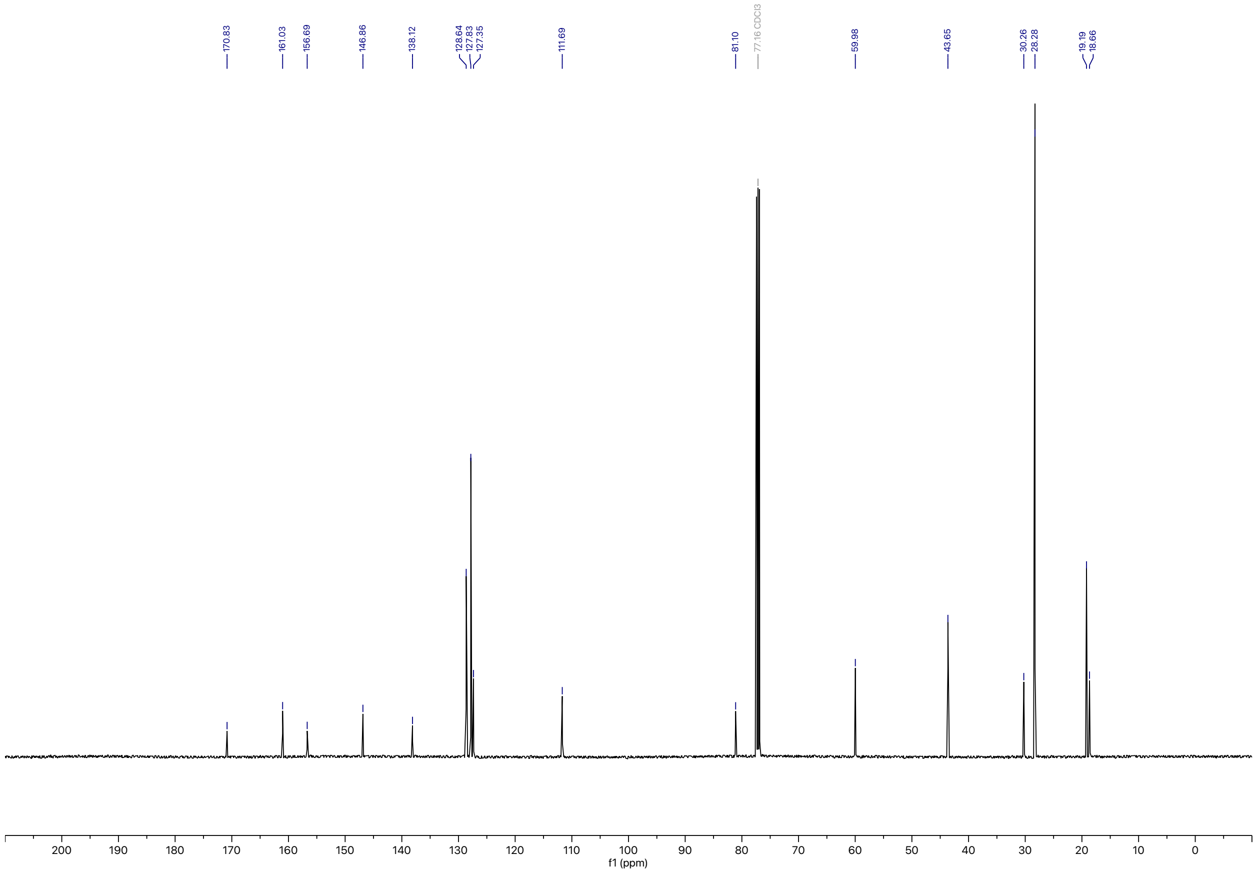

^{1}H NMR (500 MHz, chloroform-*d*) – Standard view

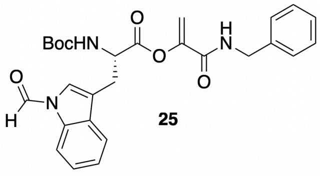

25

Chemical Formula: $C_{27}H_{29}N_3O_6$
Exact Mass: 491.21

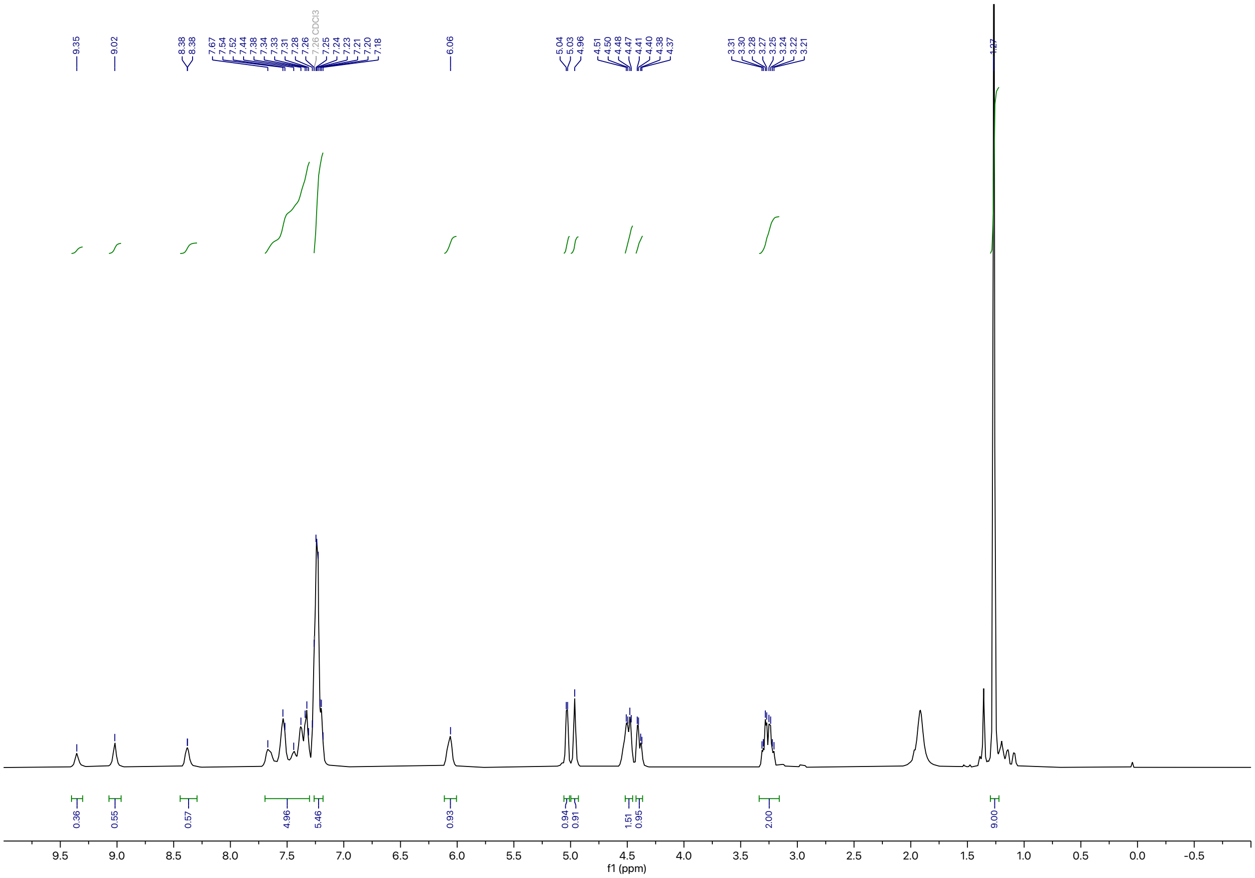

$^{13}C\{^1H\}$ NMR (125 MHz, chloroform-*d*) – Standard view

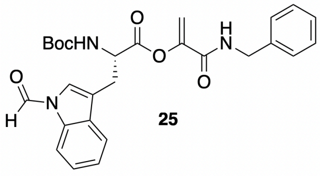

25

Chemical Formula: $C_{27}H_{29}N_3O_6$
Exact Mass: 491.21

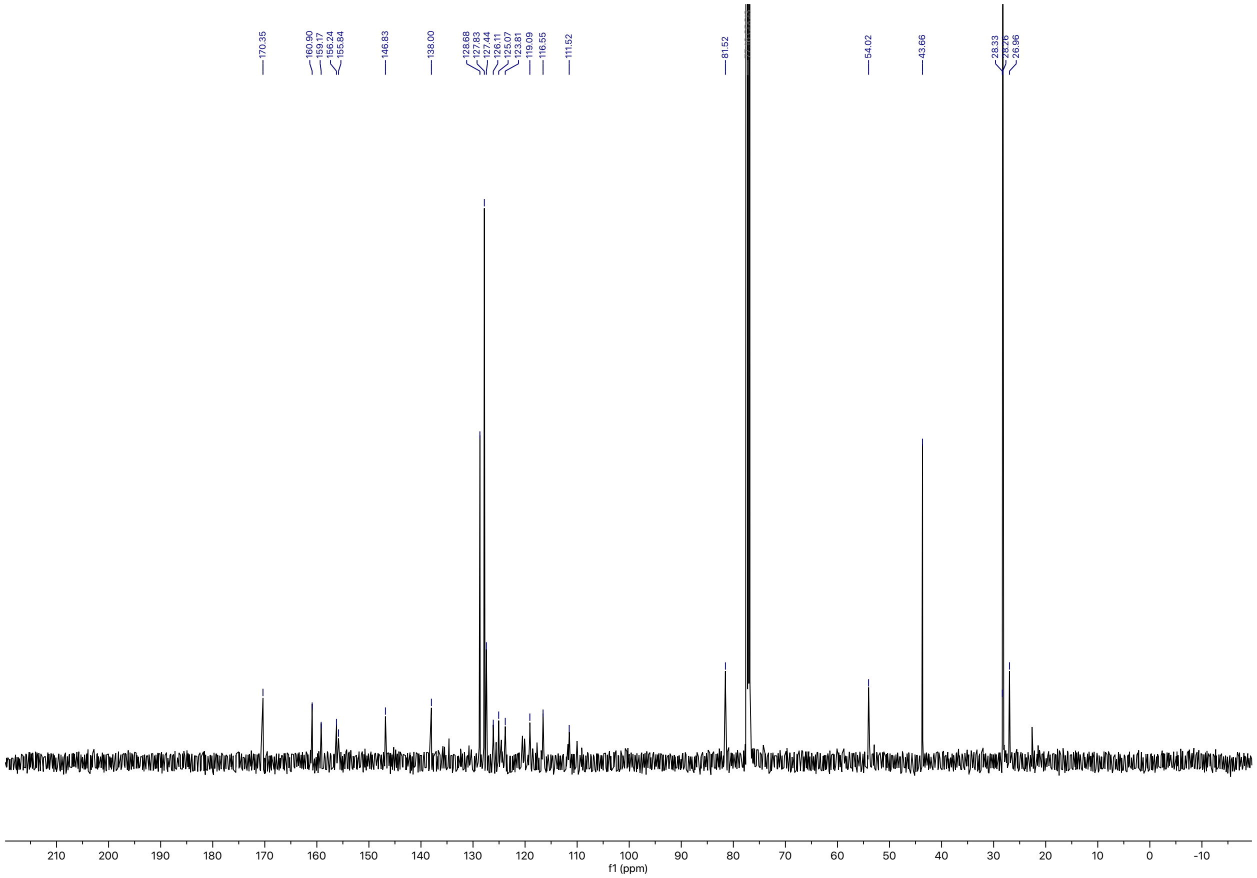

^{1}H NMR (500 MHz, chloroform-*d*) – Standard view

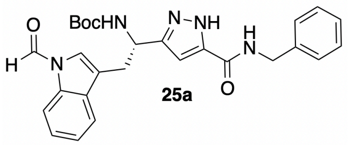

Chemical Formula: $C_{27}H_{29}N_5O_4$
Exact Mass: 487.22

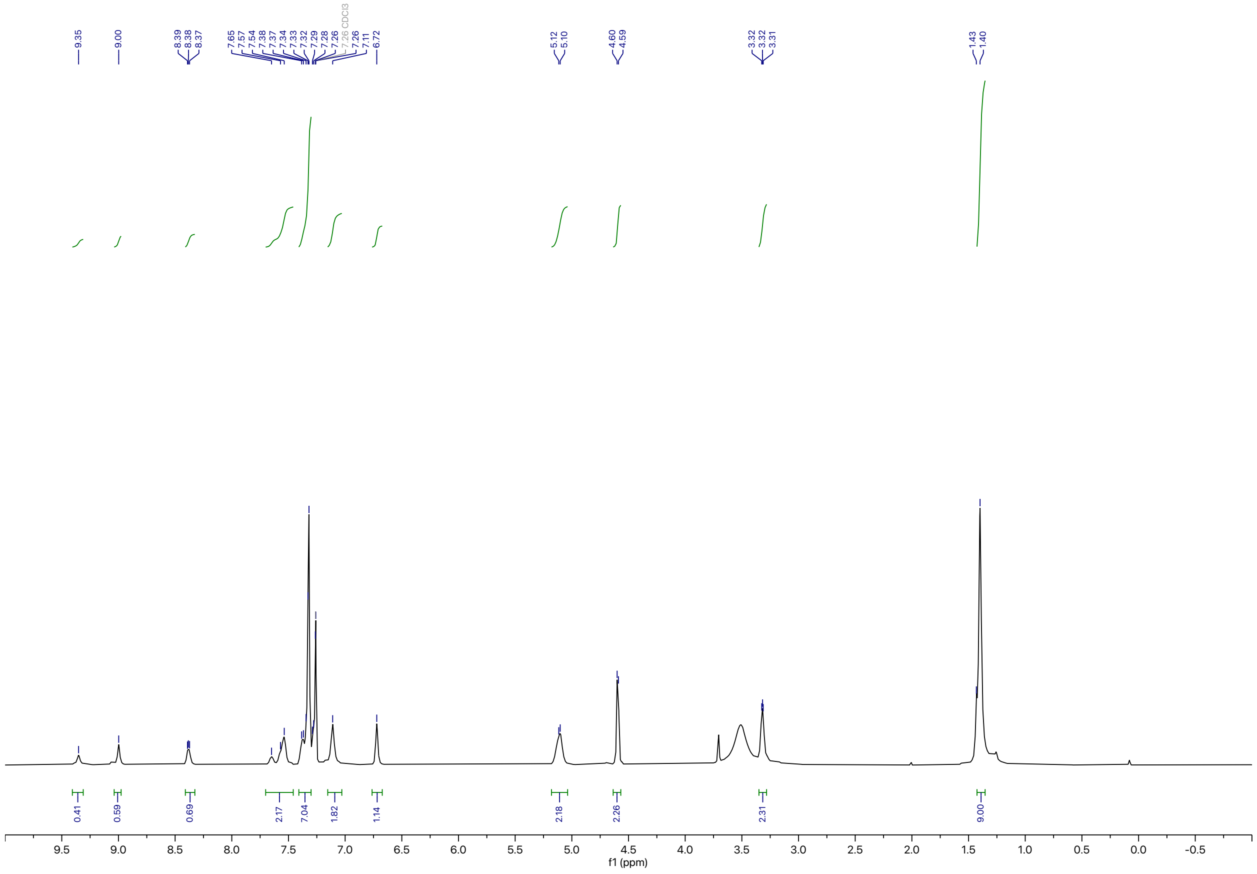

$^{13}C\{^{1}H\}$ NMR (125 MHz, chloroform-*d*) – Standard view

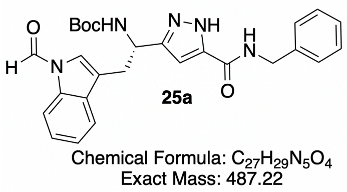

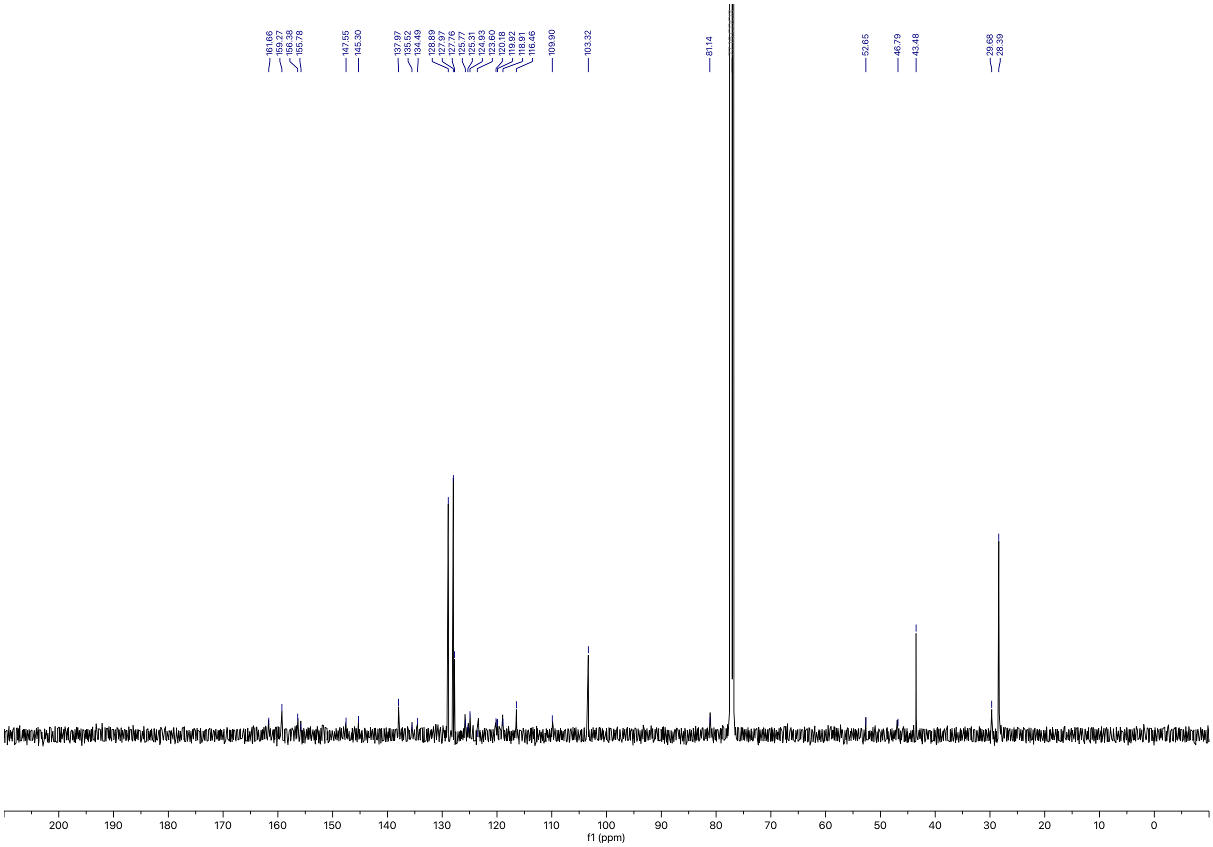

Multiplicity-edited HSQC NMR (125 MHz, chloroform-*d*) – Standard view

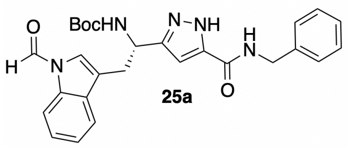

Chemical Formula: $C_{27}H_{29}N_5O_4$
Exact Mass: 487.22

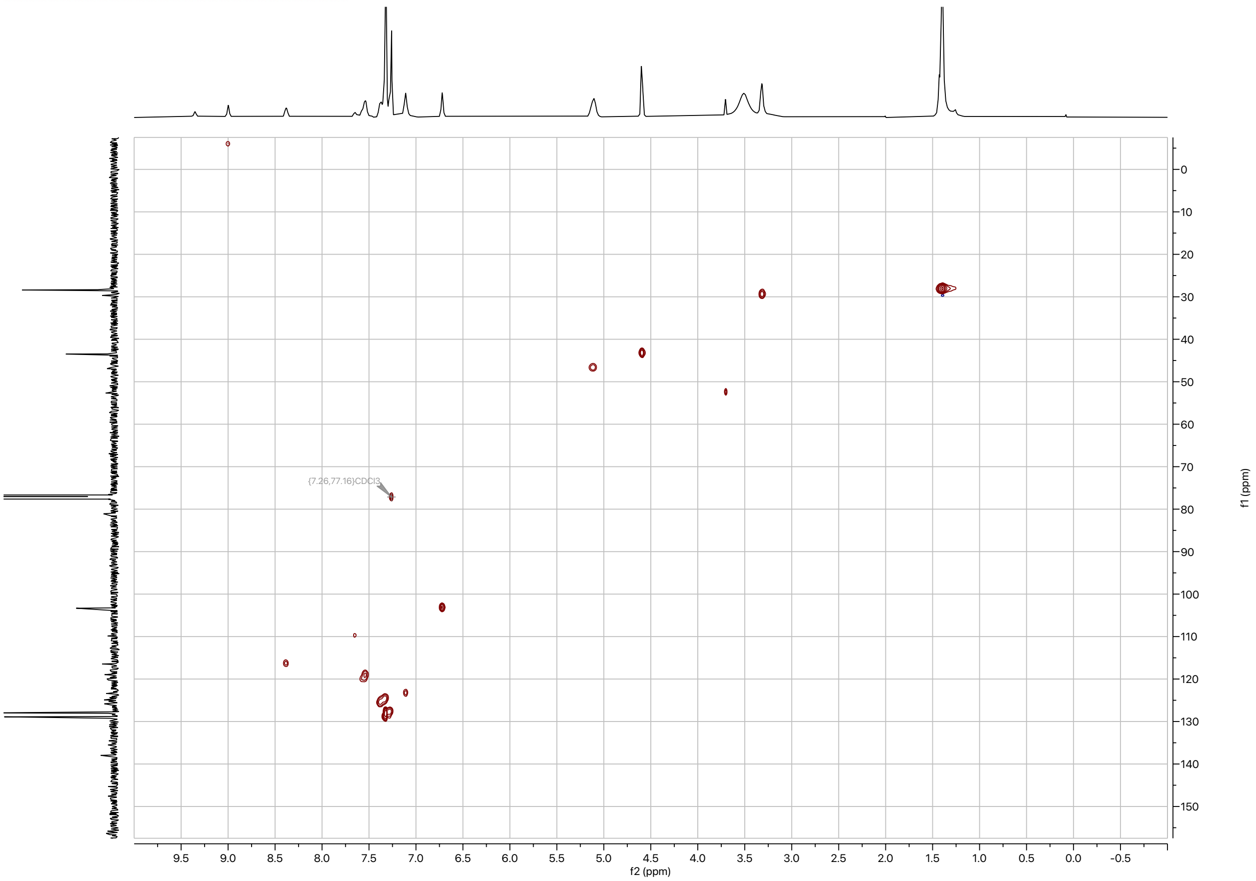

COSY NMR (125 MHz, chloroform-*d*) – Standard view

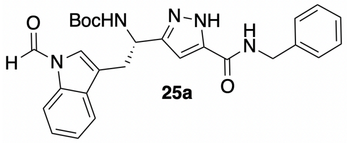

Chemical Formula: $C_{27}H_{29}N_5O_4$
Exact Mass: 487.22

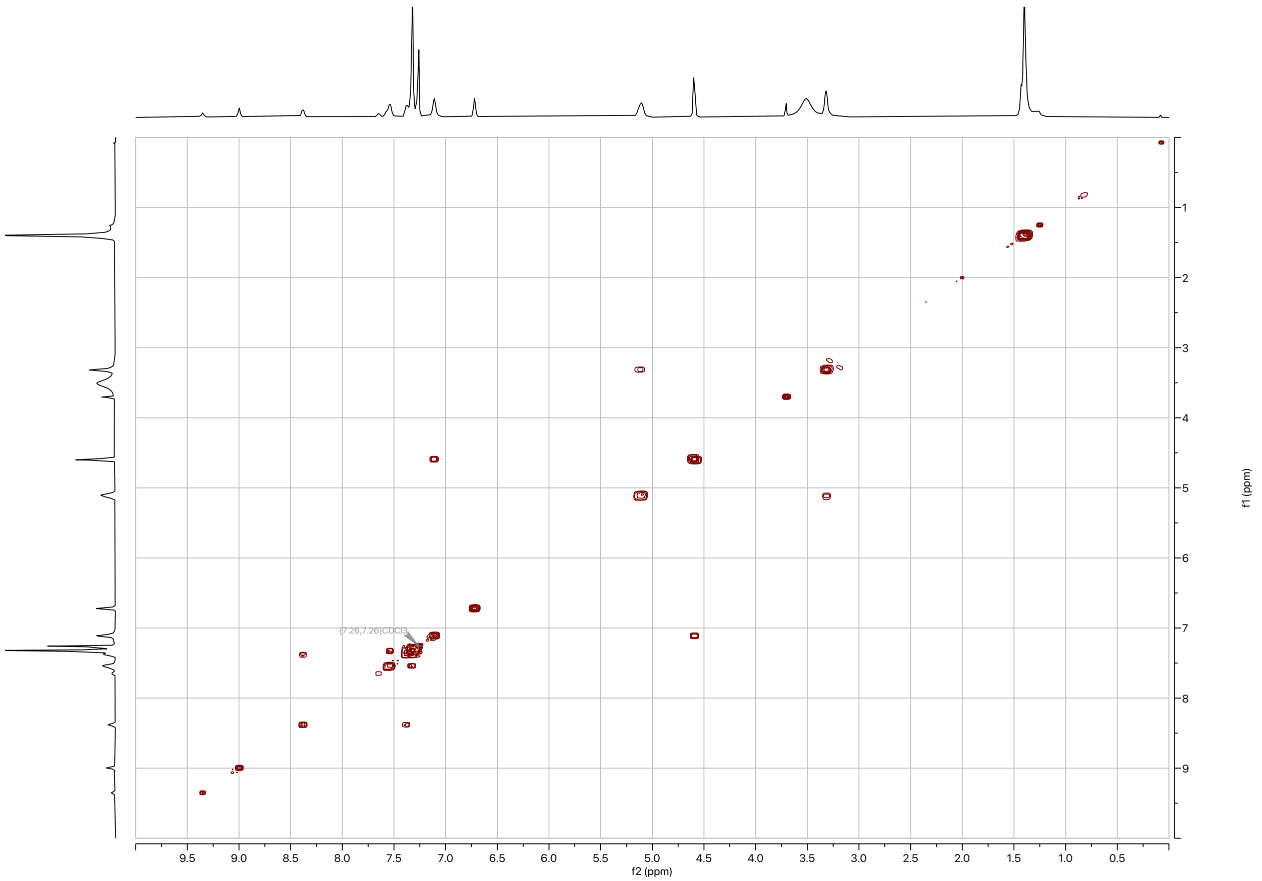

^{1}H NMR (500 MHz, chloroform-*d*) – Standard view

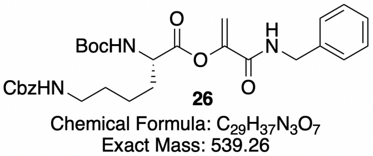

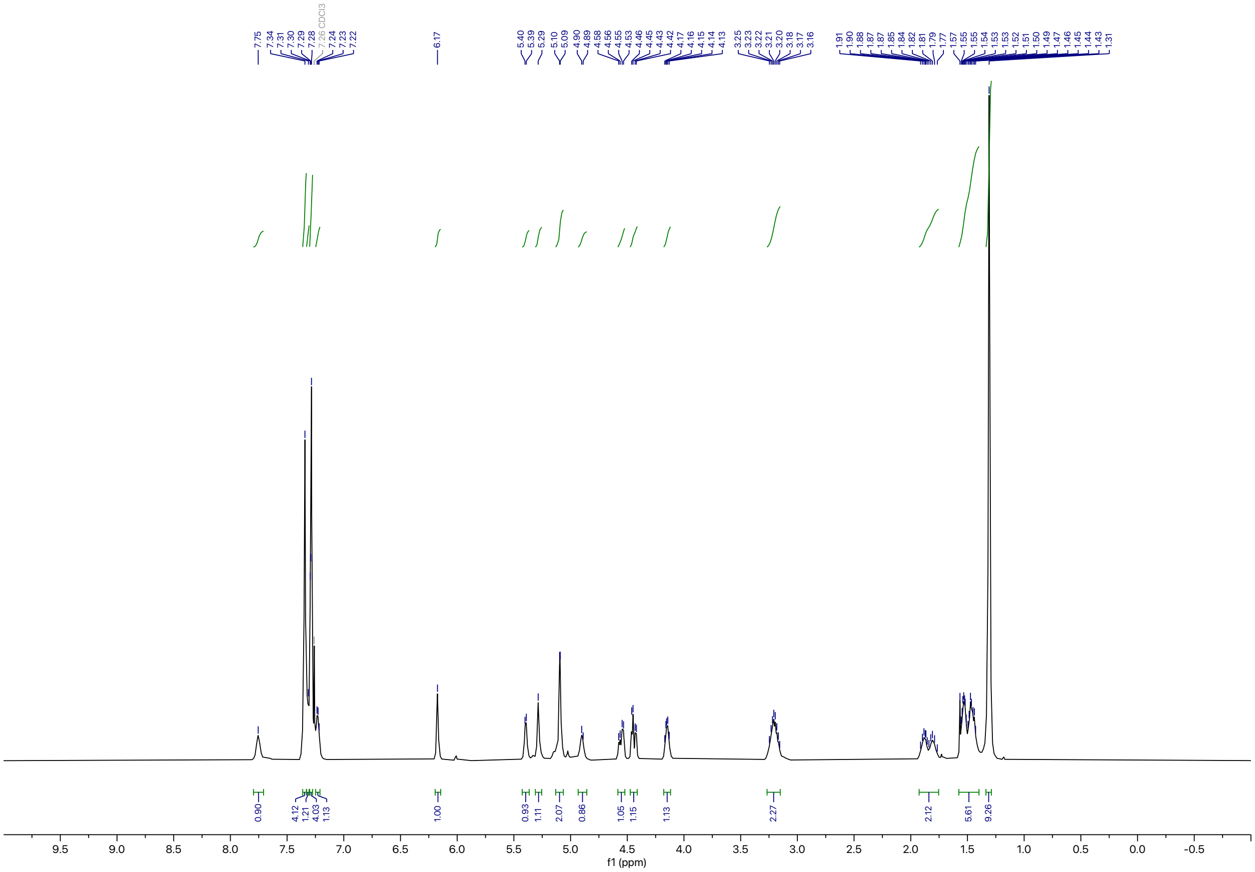

$^{13}C\{^{1}H\}$ NMR (125 MHz, chloroform-*d*) – Standard view

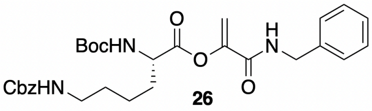

26

Chemical Formula: $C_{29}H_{37}N_3O_7$
Exact Mass: 539.26

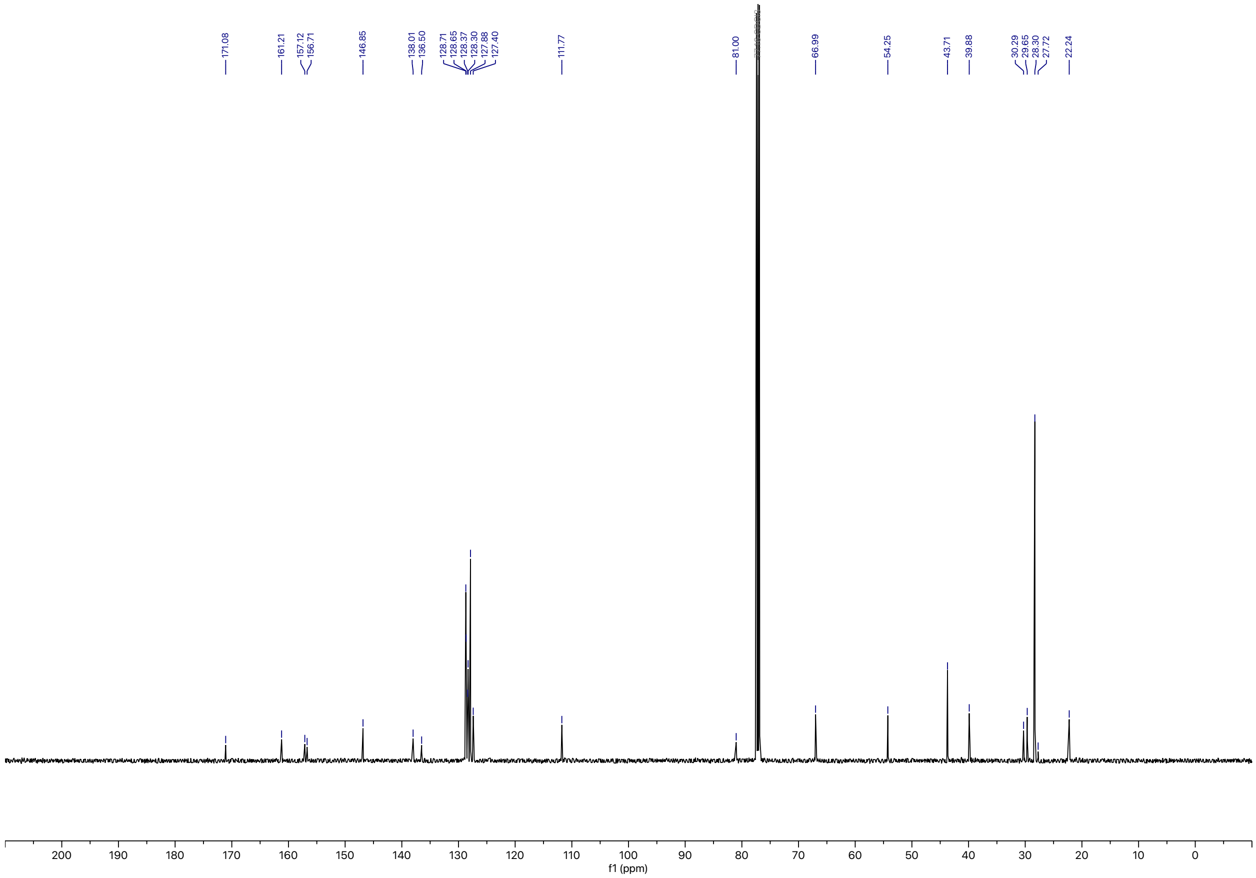

^{1}H NMR (500 MHz, chloroform-*d*) – Standard view

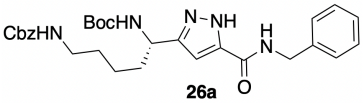

26a

Chemical Formula: $C_{29}H_{37}N_5O_5$

Exact Mass: 535.28

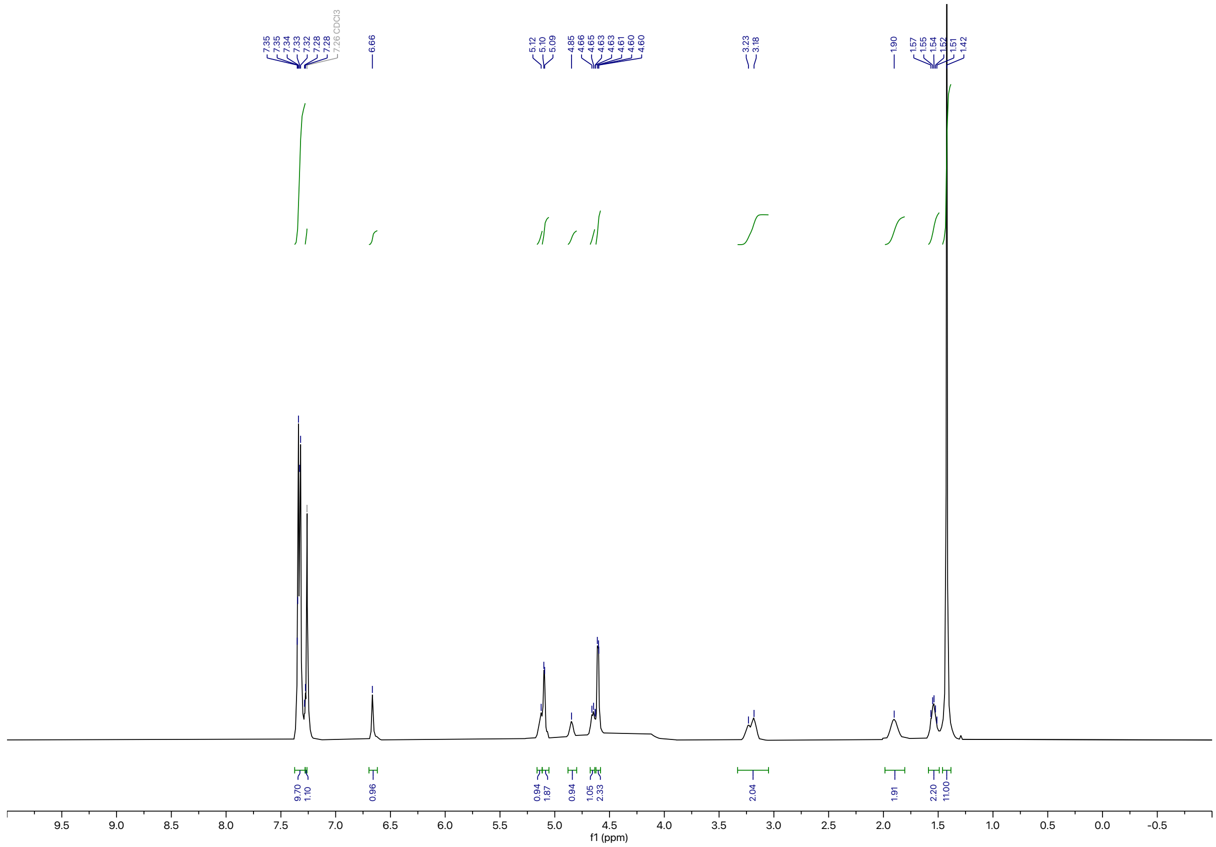

$^{13}C\{^1H\}$ NMR (125 MHz, chloroform-*d*) – Standard view

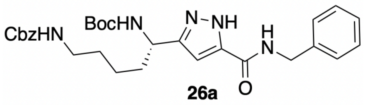

26a

Chemical Formula: $C_{29}H_{37}N_5O_5$

Exact Mass: 535.28

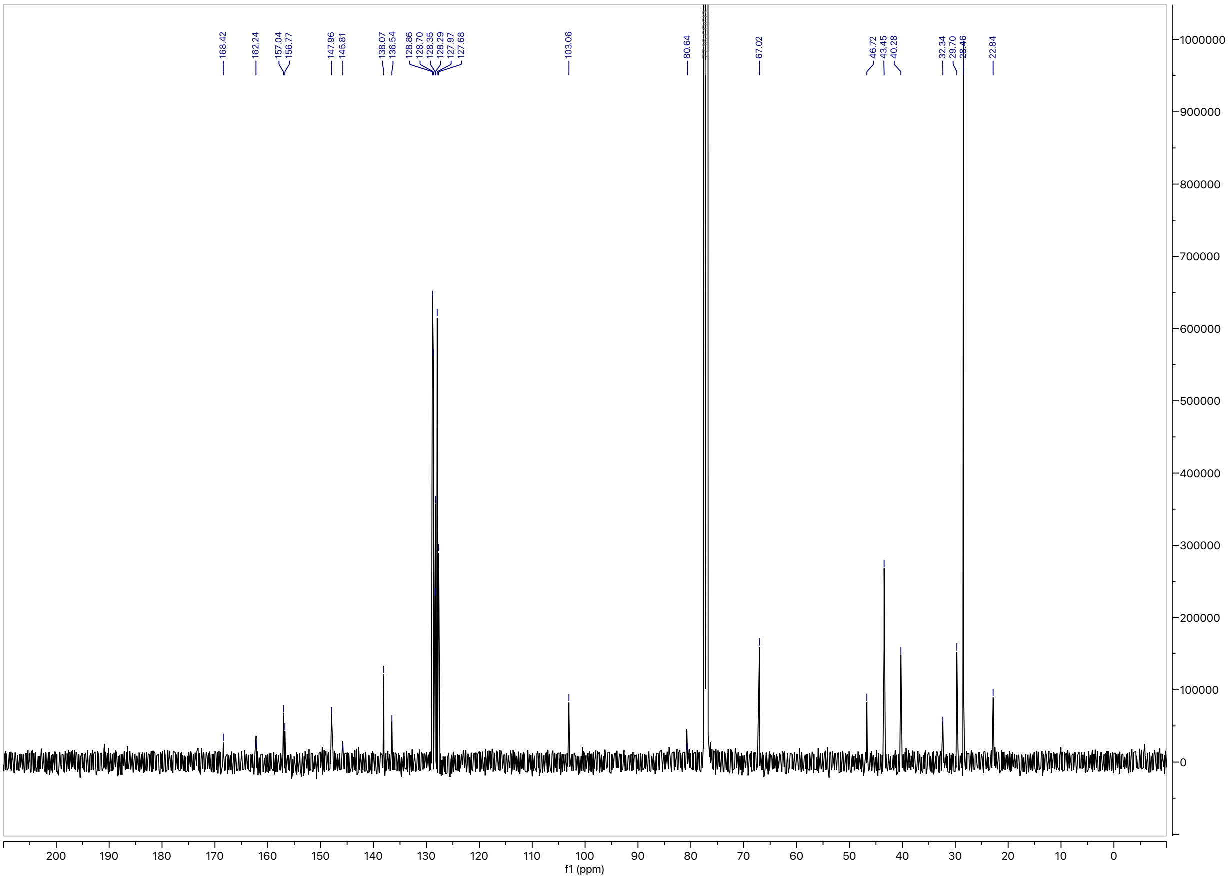

Multiplicity-edited HSQC NMR (125 MHz, chloroform-*d*) – Standard view

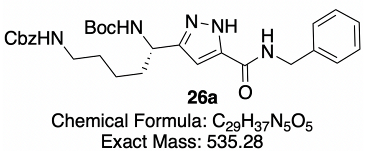

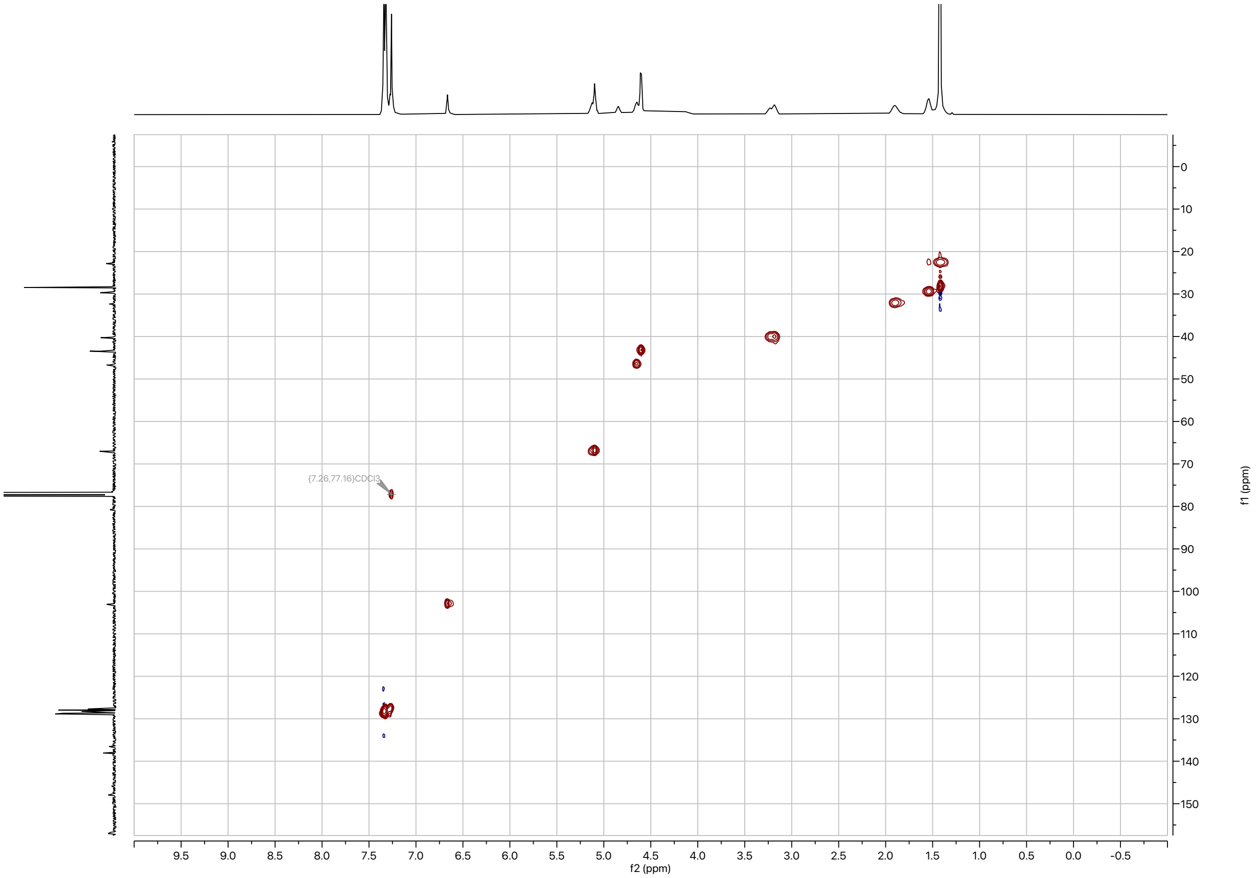

COSY NMR (125 MHz, chloroform-*d*) – Standard view

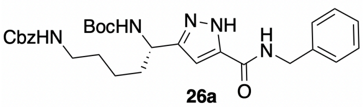

26a

Chemical Formula: $C_{29}H_{37}N_5O_5$

Exact Mass: 535.28

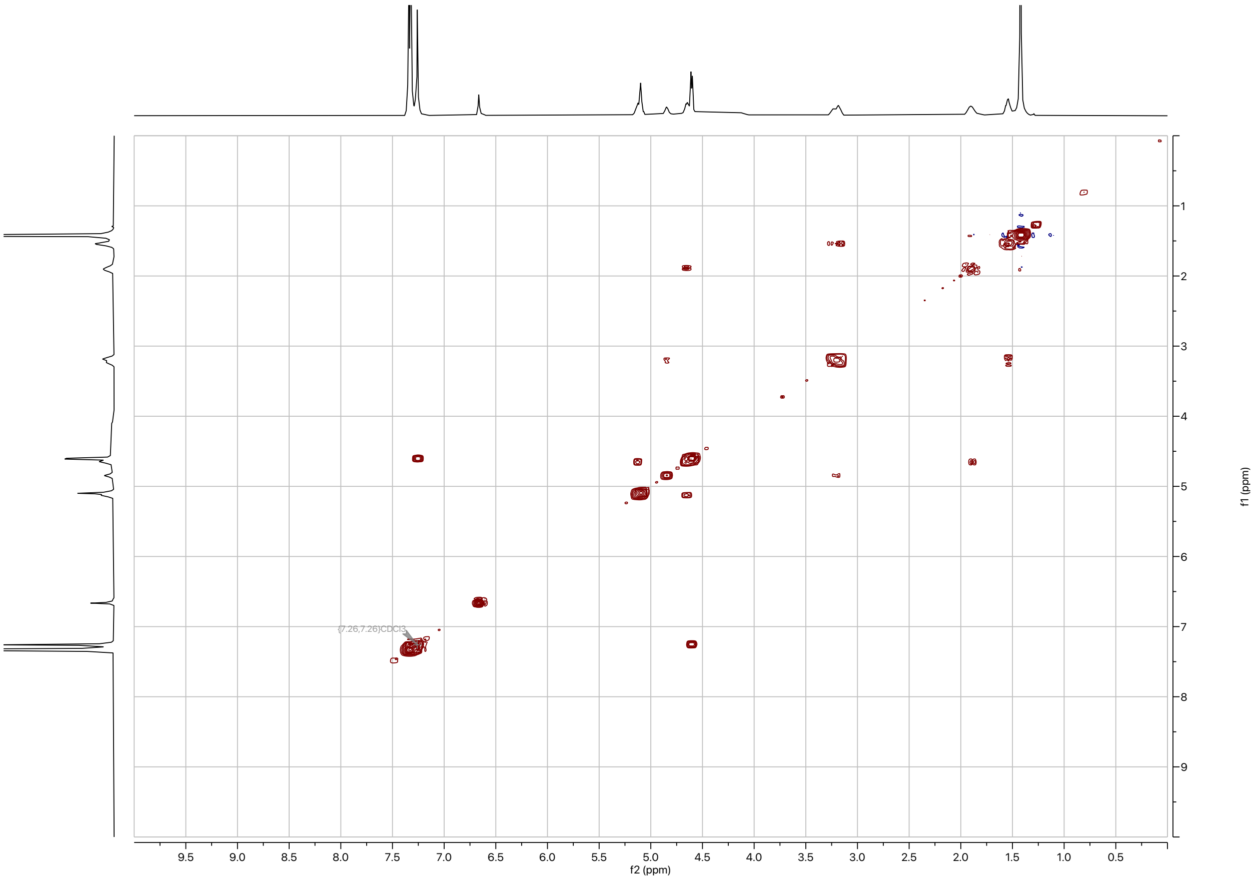

^{1}H NMR (500 MHz, chloroform-*d*) – Standard view

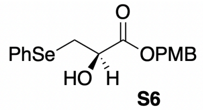

S6

Chemical Formula: $C_{17}H_{18}O_4Se$
Exact Mass: 366.0370

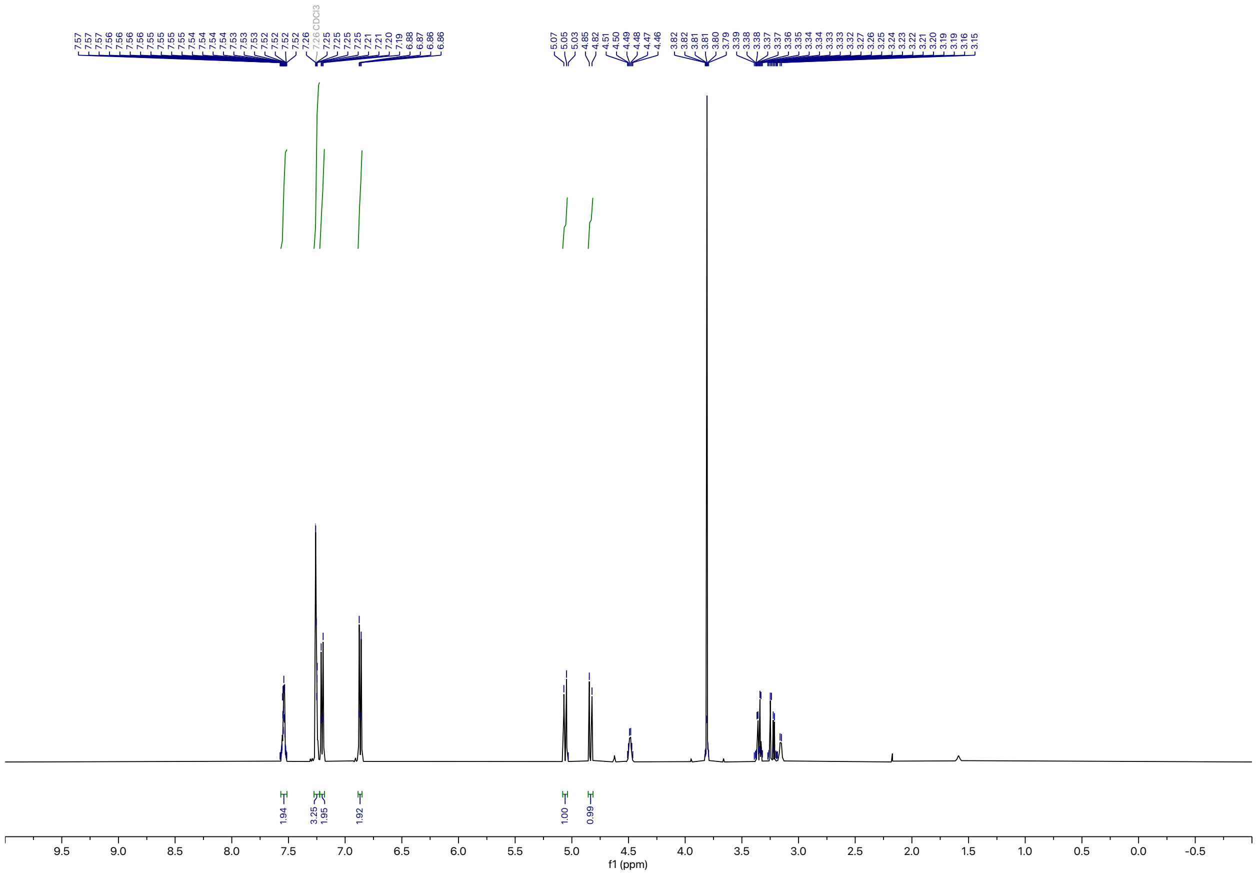

$^{13}C\{^1H\}$ NMR (125 MHz, chloroform-*d*) – Standard view

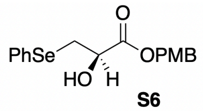

S6

Chemical Formula: $C_{17}H_{18}O_4Se$
Exact Mass: 366.0370

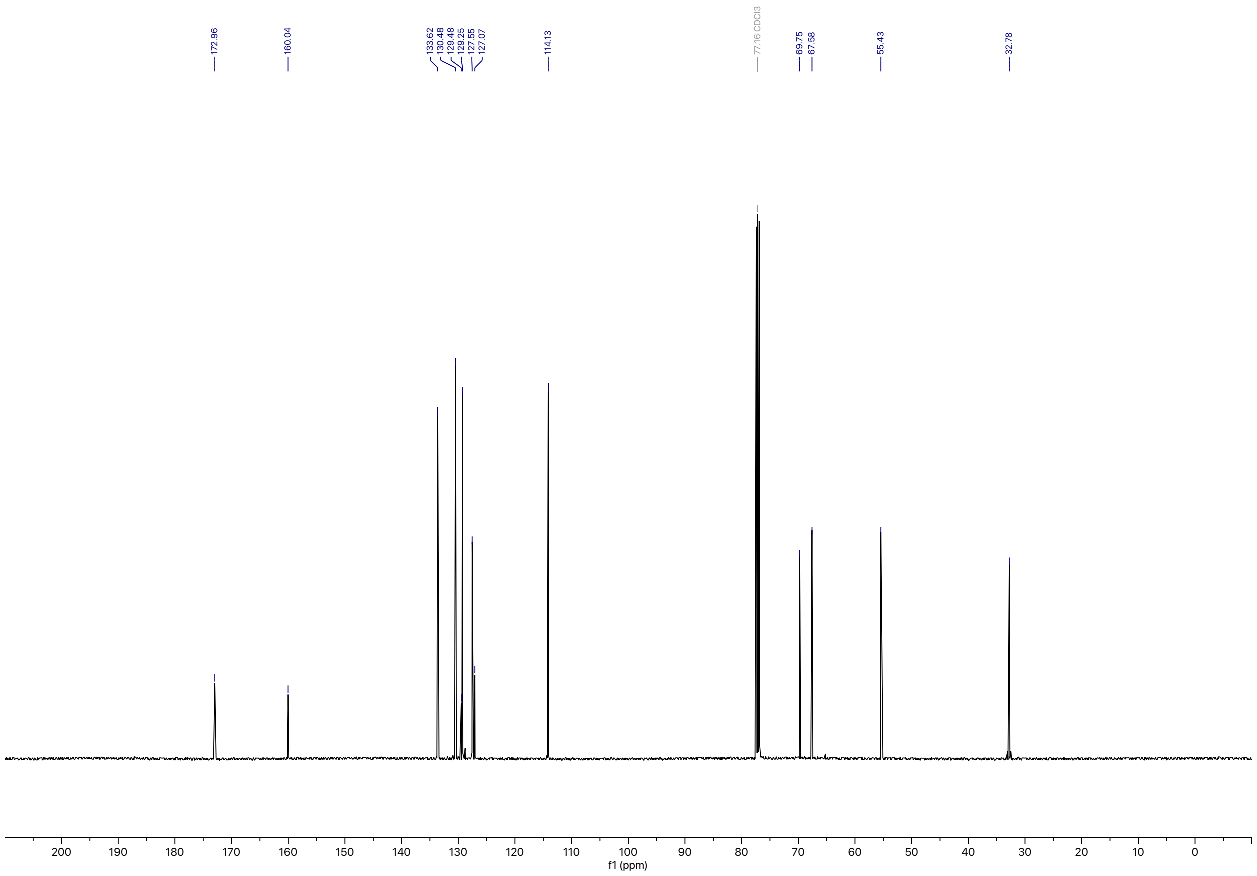

^{1}H NMR (500 MHz, chloroform-*d*) – Standard view

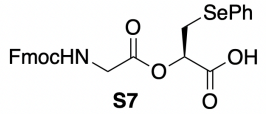

Chemical Formula: $C_{26}H_{23}NO_{6}Se$
Exact Mass: 525.0691

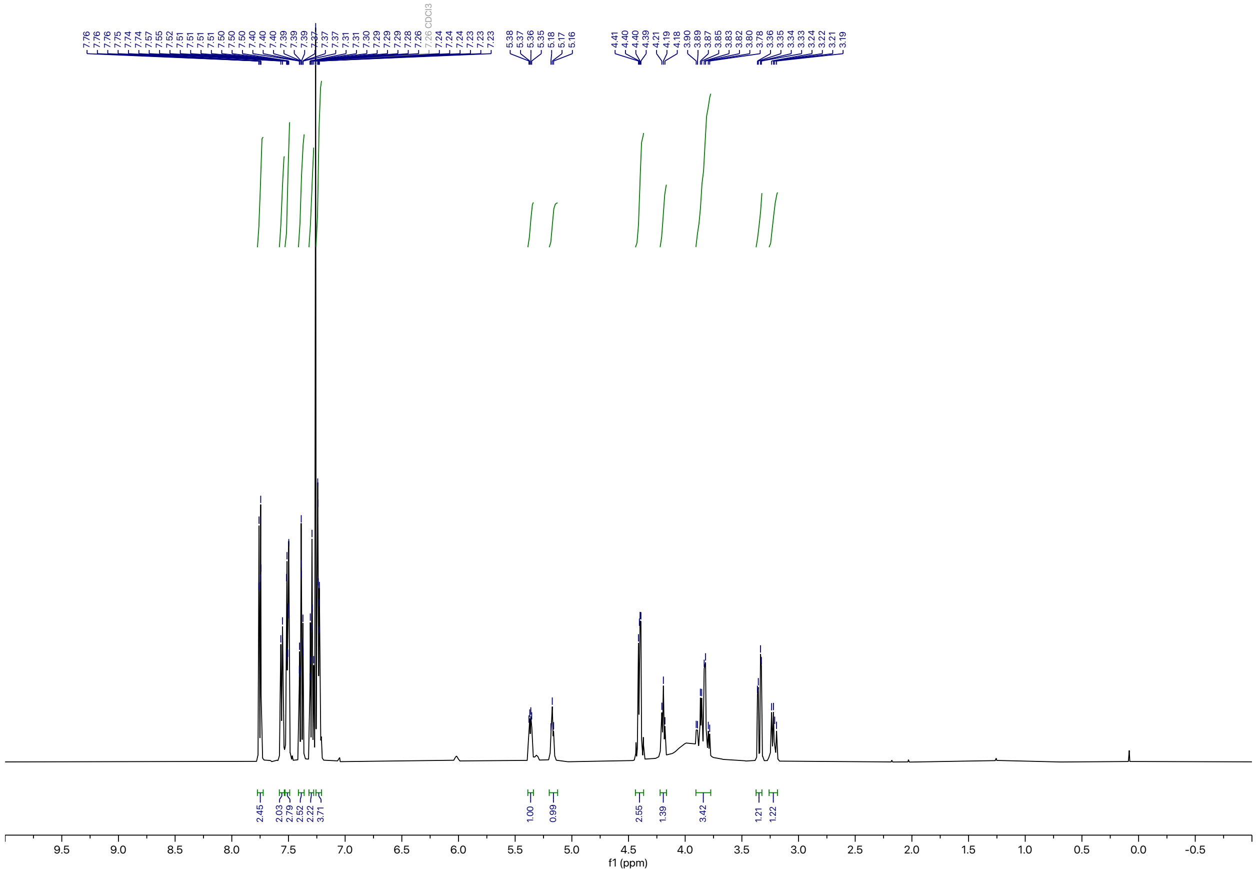

$^{13}C\{^{1}H\}$ NMR (125 MHz, chloroform-*d*) – Standard view

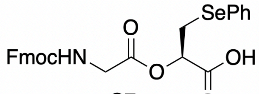

S7

Chemical Formula: $C_{26}H_{23}NO_6Se$
Exact Mass: 525.0691

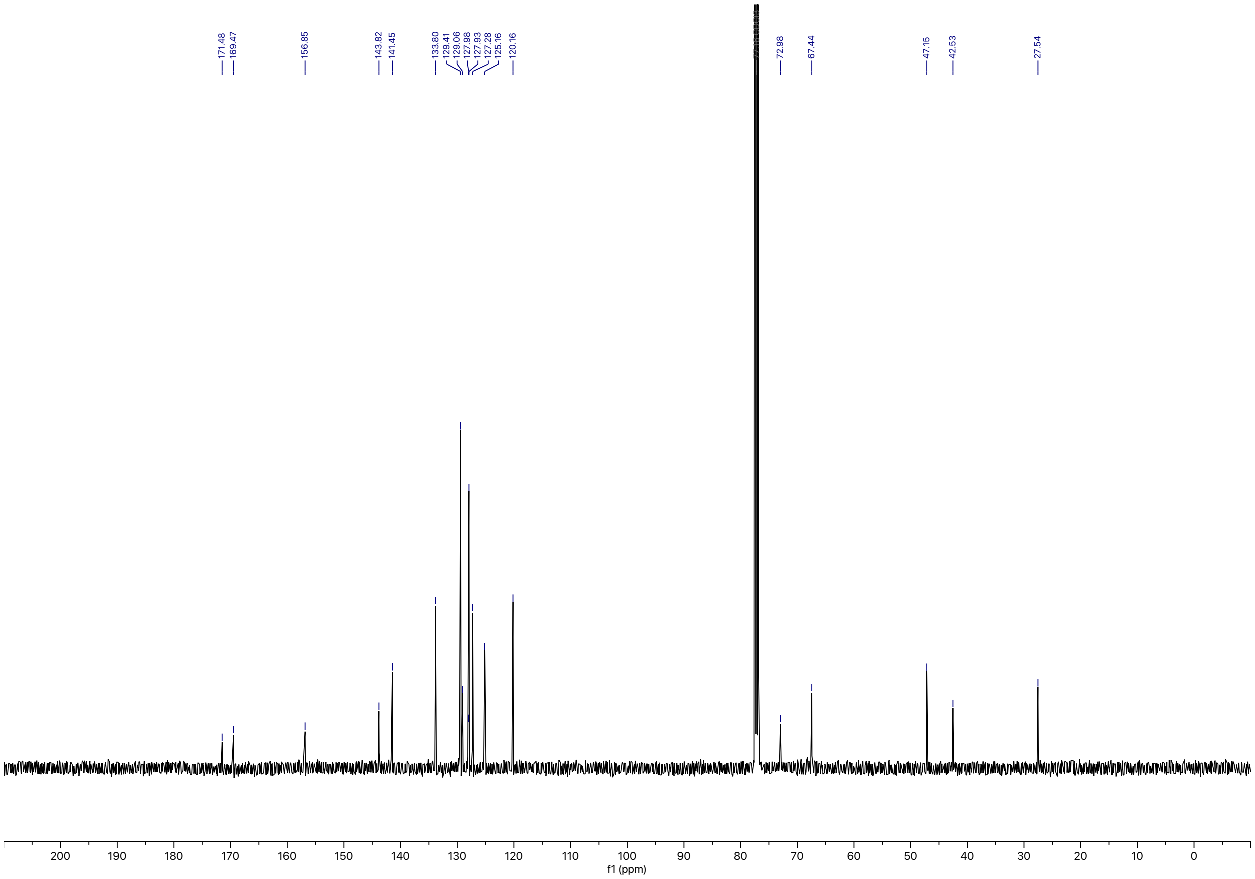

^{1}H NMR (500 MHz, chloroform-*d*) – Standard view

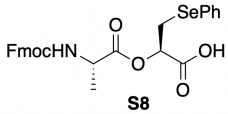

S8

Chemical Formula: $C_{27}H_{25}NO_6Se$
Exact Mass: 539.0847

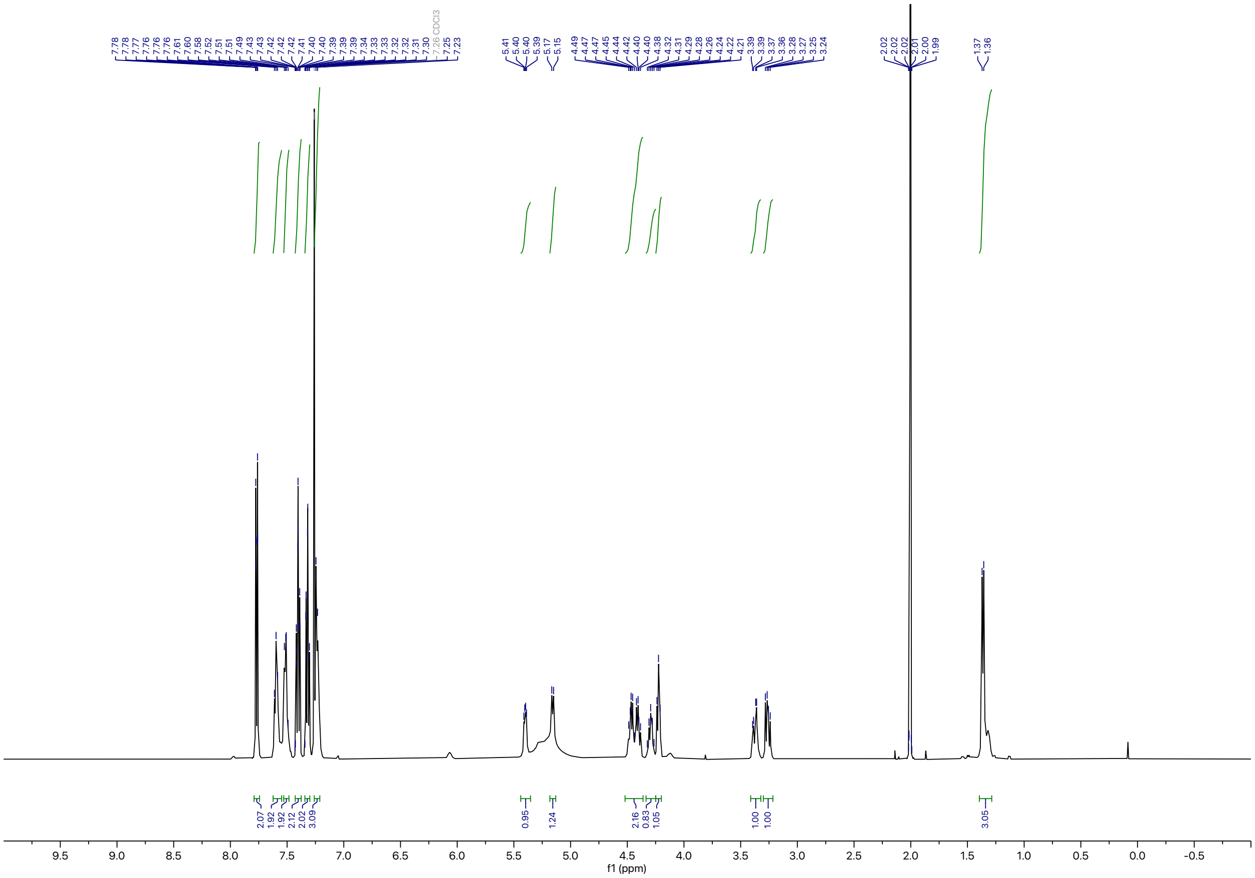

$^{13}C\{^{1}H\}$ NMR (125 MHz, chloroform-*d*) – Standard view

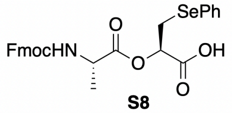

S8

Chemical Formula: $C_{27}H_{25}NO_6Se$
Exact Mass: 539.0847

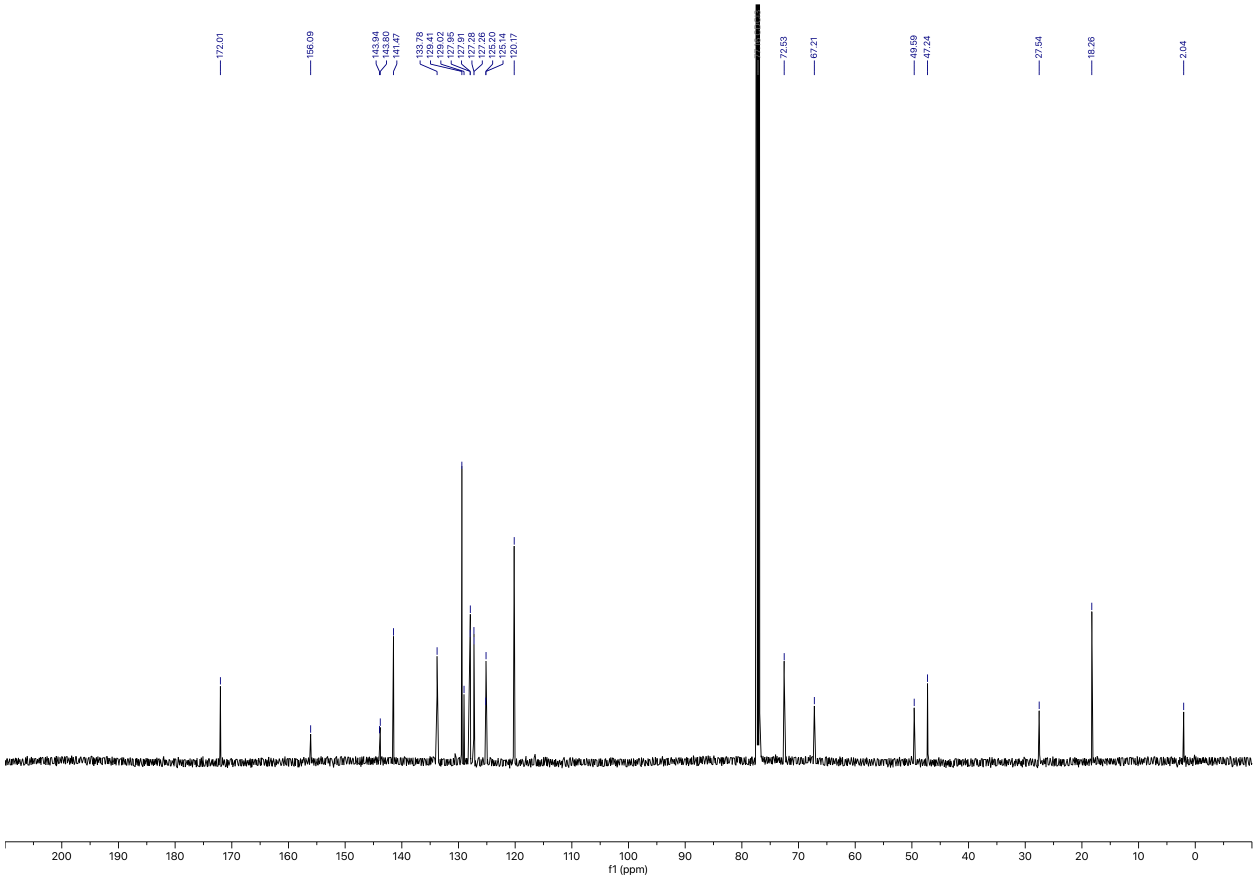

^{1}H NMR (500 MHz, DMSO-*d6*) – Extended view

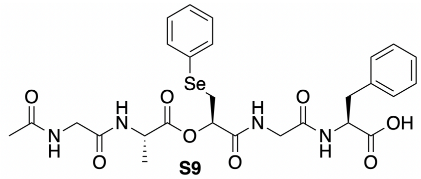

S9

Chemical Formula: $C_{27}H_{32}N_4O_8Se$

Exact Mass: 620.14

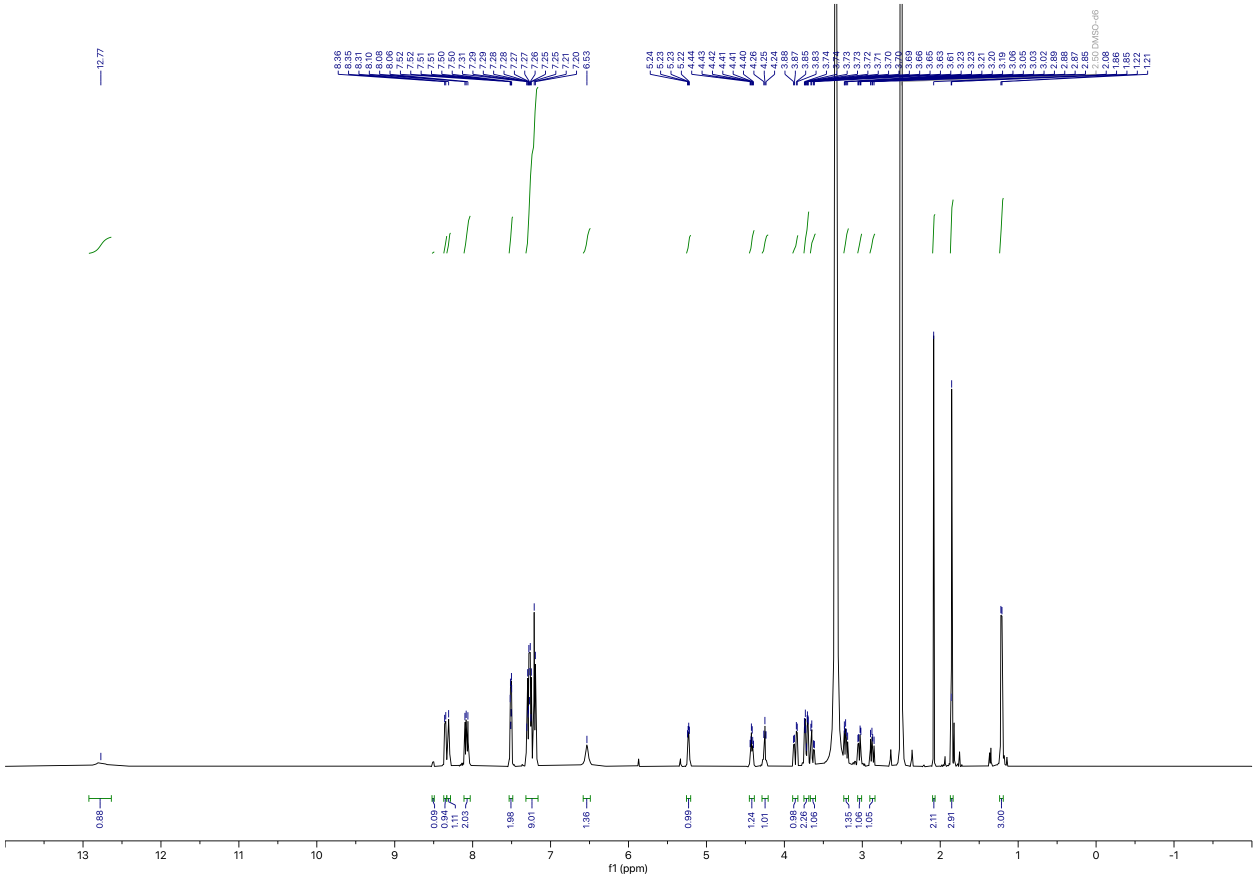

$^{13}C\{^{1}H\}$ NMR (125 MHz, DMSO-*d6*) – Standard view

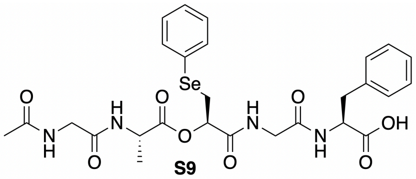

S9

Chemical Formula: $C_{27}H_{32}N_4O_8Se$

Exact Mass: 620.14

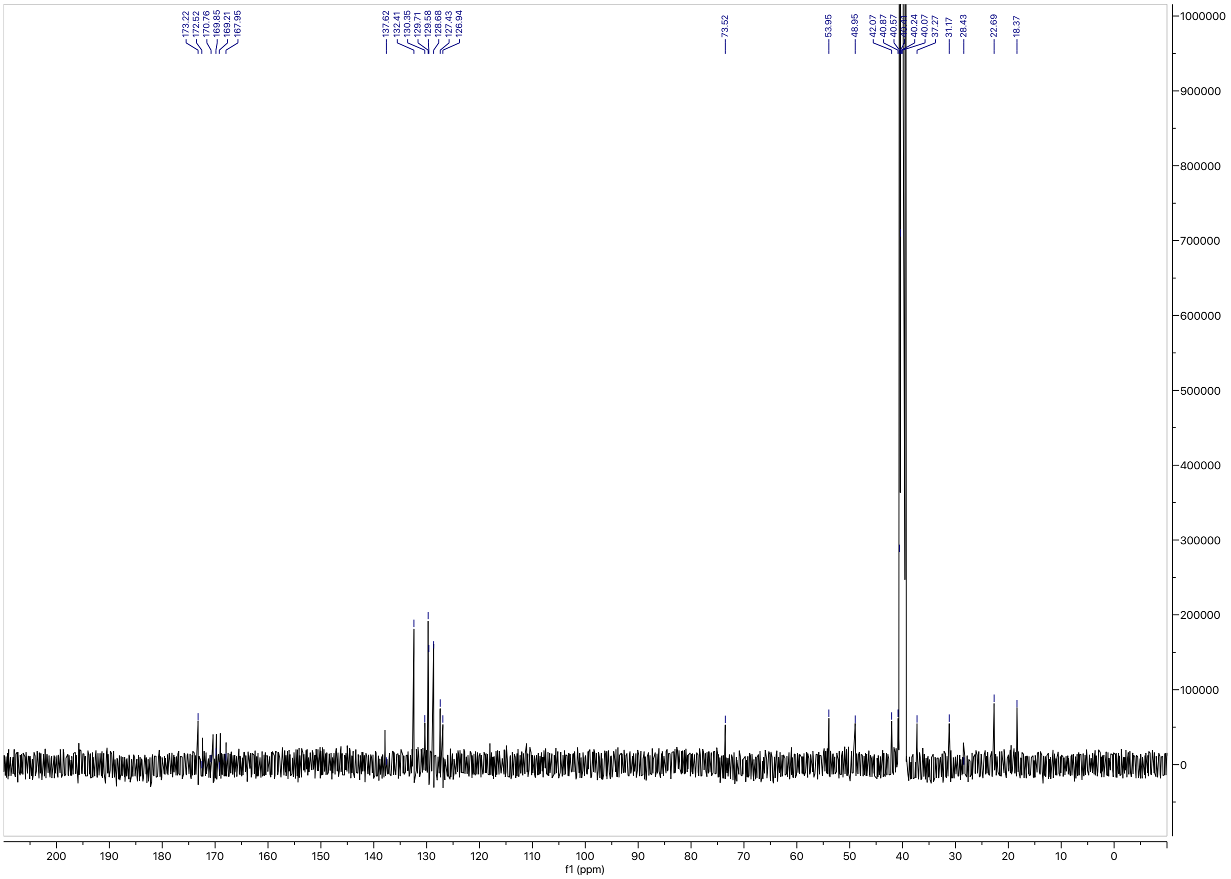

^{1}H NMR (500 MHz, DMSO-*d6*) – Extended view

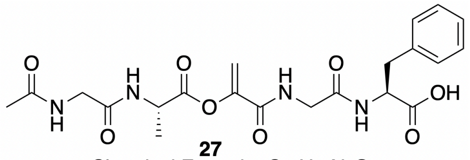

27
Chemical Formula: $C_{21}H_{26}N_4O_8$
Exact Mass: 462.18

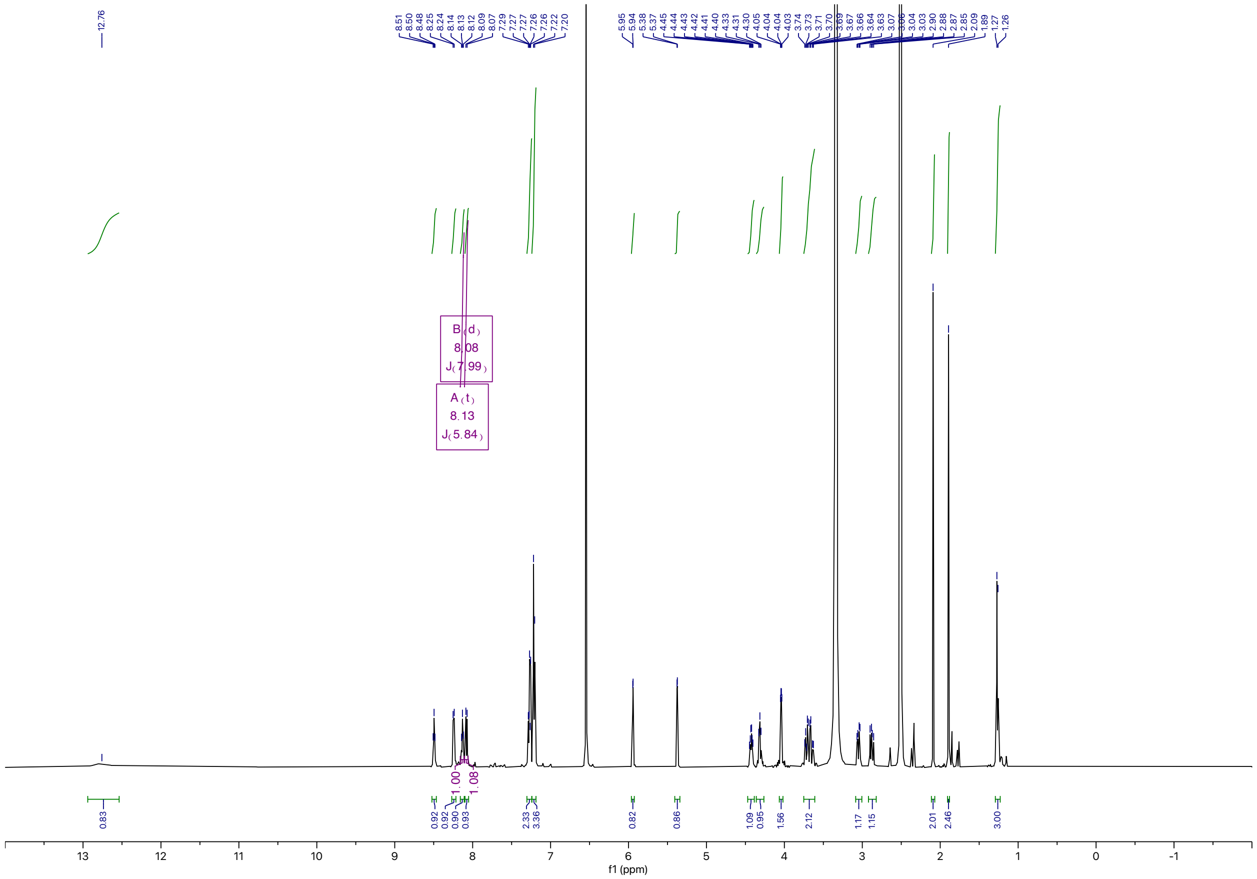

$^{13}C\{^{1}H\}$ NMR (125 MHz, DMSO-*d6*) – Standard view

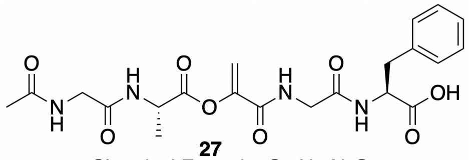

27
Chemical Formula: $C_{21}H_{26}N_4O_8$
Exact Mass: 462.18

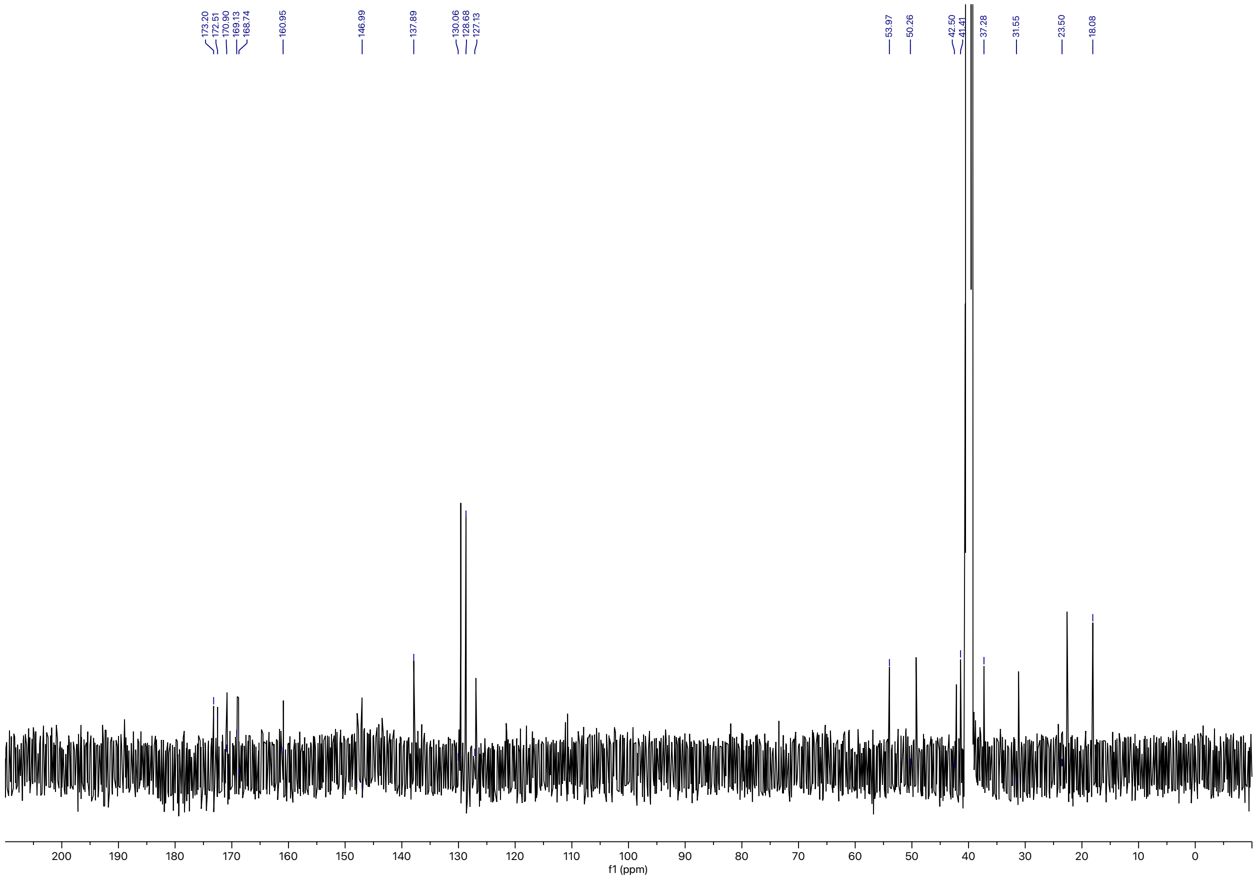

^{1}H NMR (500 MHz, DMSO-*d6*) – Extended view

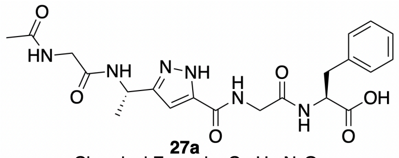

27a

Chemical Formula: $C_{21}H_{26}N_6O_6$

Exact Mass: 458.19

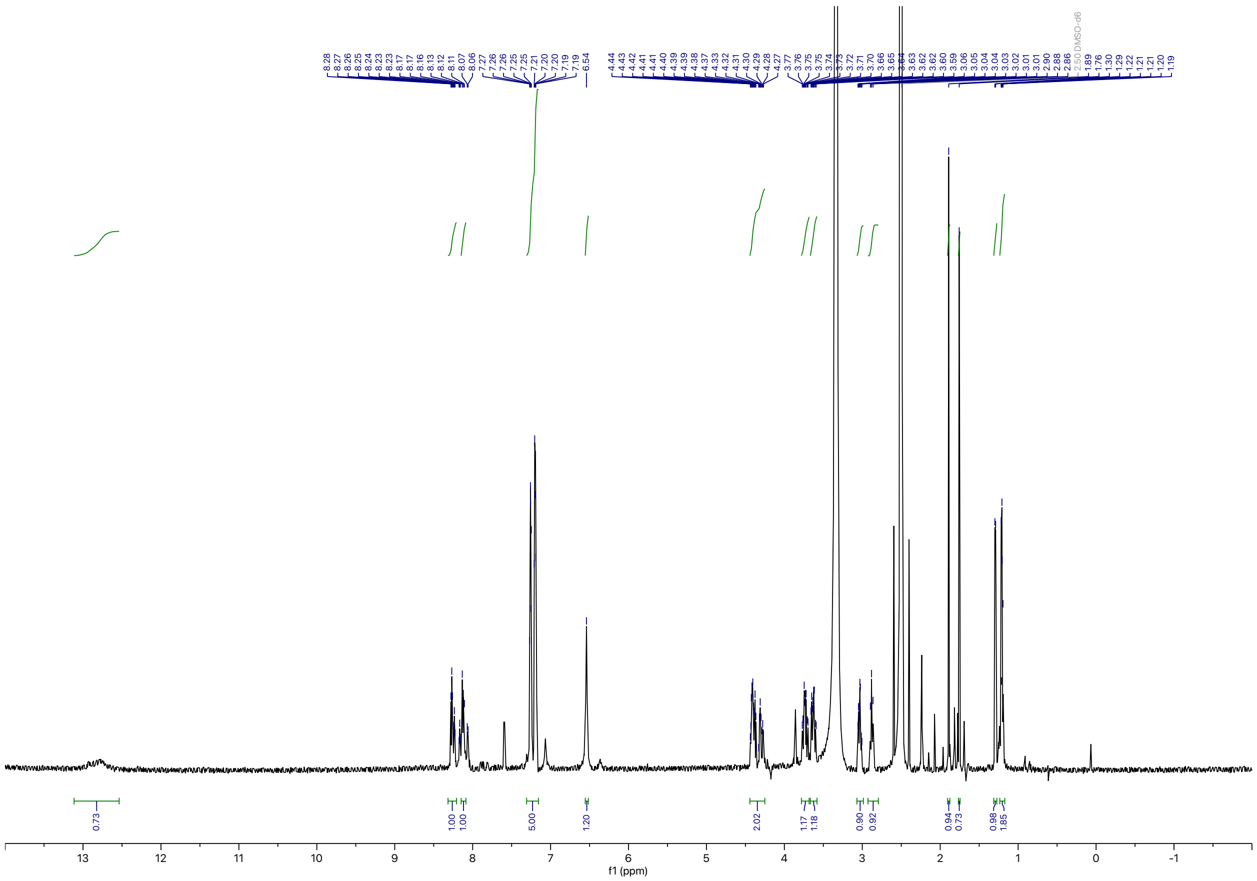

Multiplicity-edited HSQC NMR (125 MHz, chloroform-*d*) – Standard view

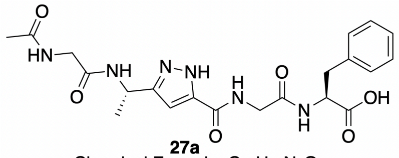

27a
Chemical Formula: $C_{21}H_{26}N_6O_6$
Exact Mass: 458.19

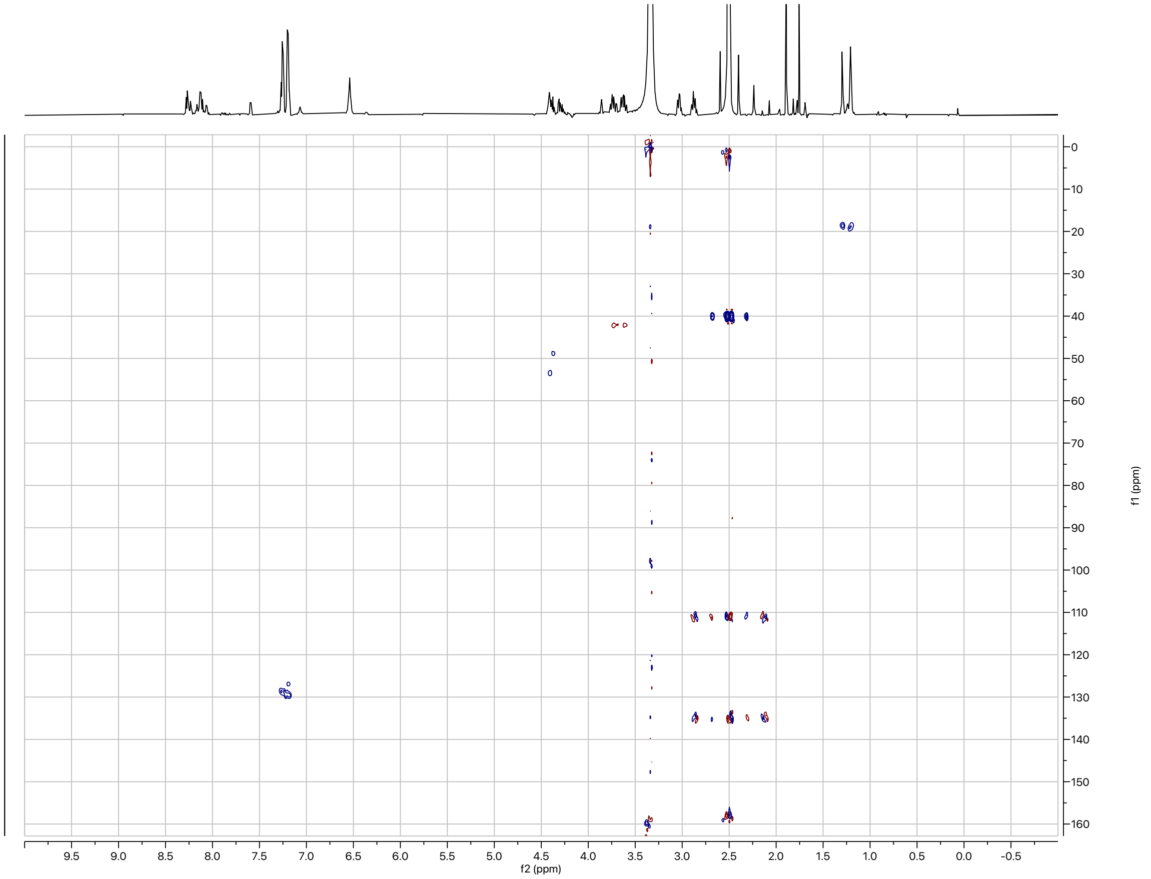

COSY NMR (125 MHz, chloroform-*d*) – Standard view

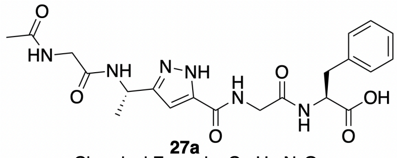

27a
Chemical Formula: $C_{21}H_{26}N_6O_6$
Exact Mass: 458.19

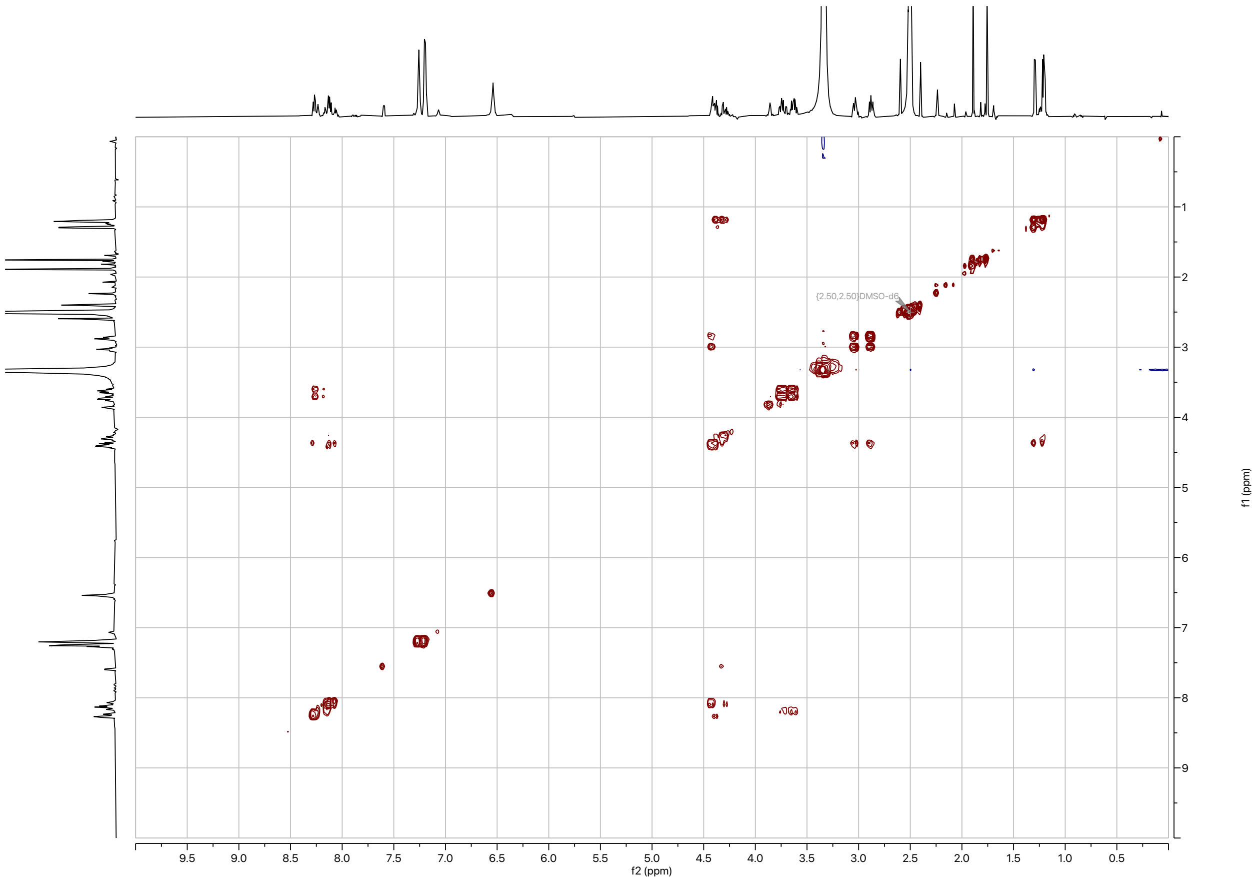

^{1}H NMR (500 MHz, DMSO-*d6*) – Extended view

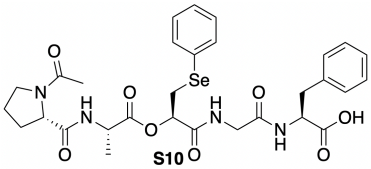

S10

Chemical Formula: $C_{30}H_{36}N_4O_8Se$
Exact Mass: 660.17

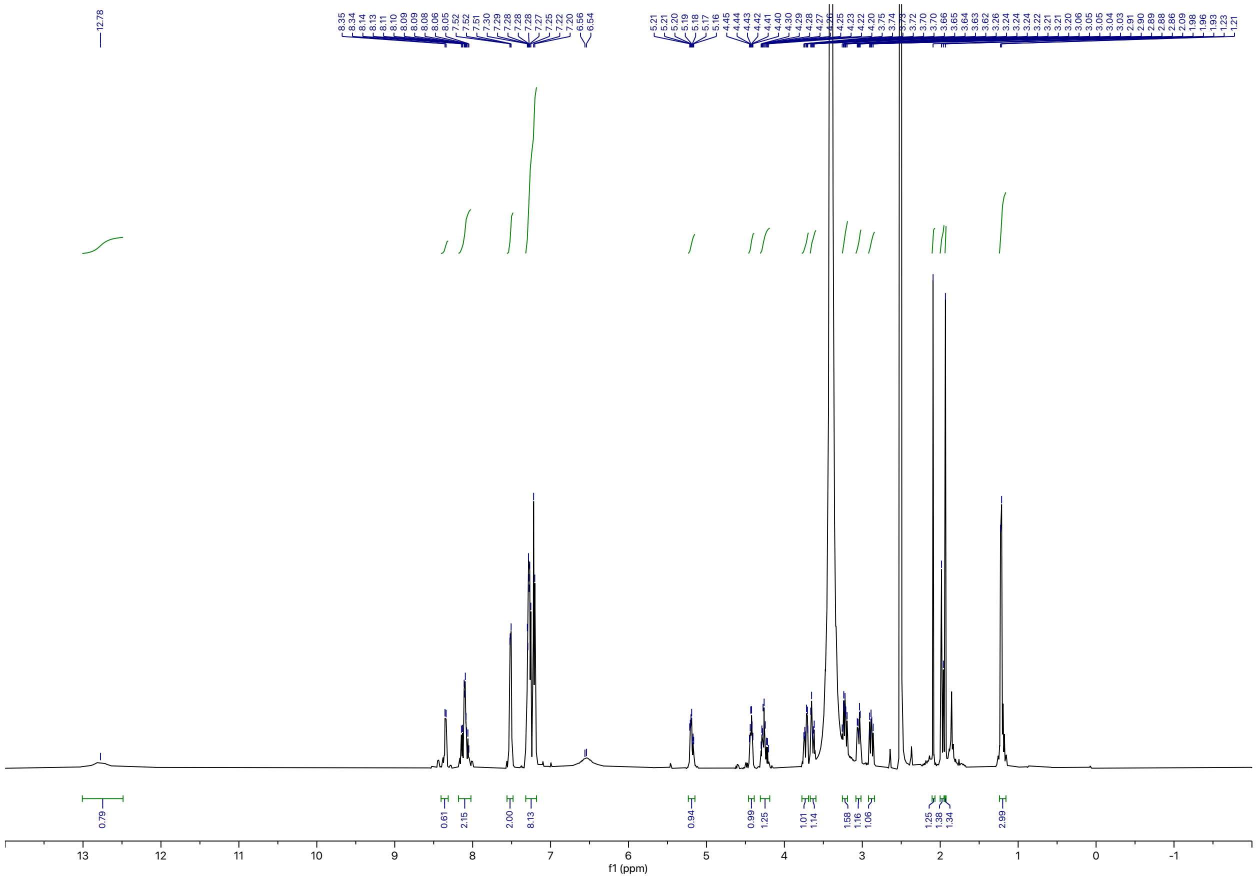

$^{13}C\{^{1}H\}$ NMR (125 MHz, DMSO-*d6*) – Standard view

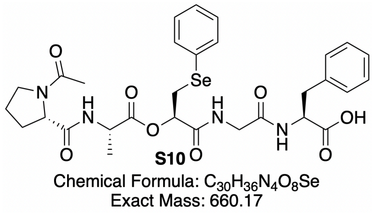

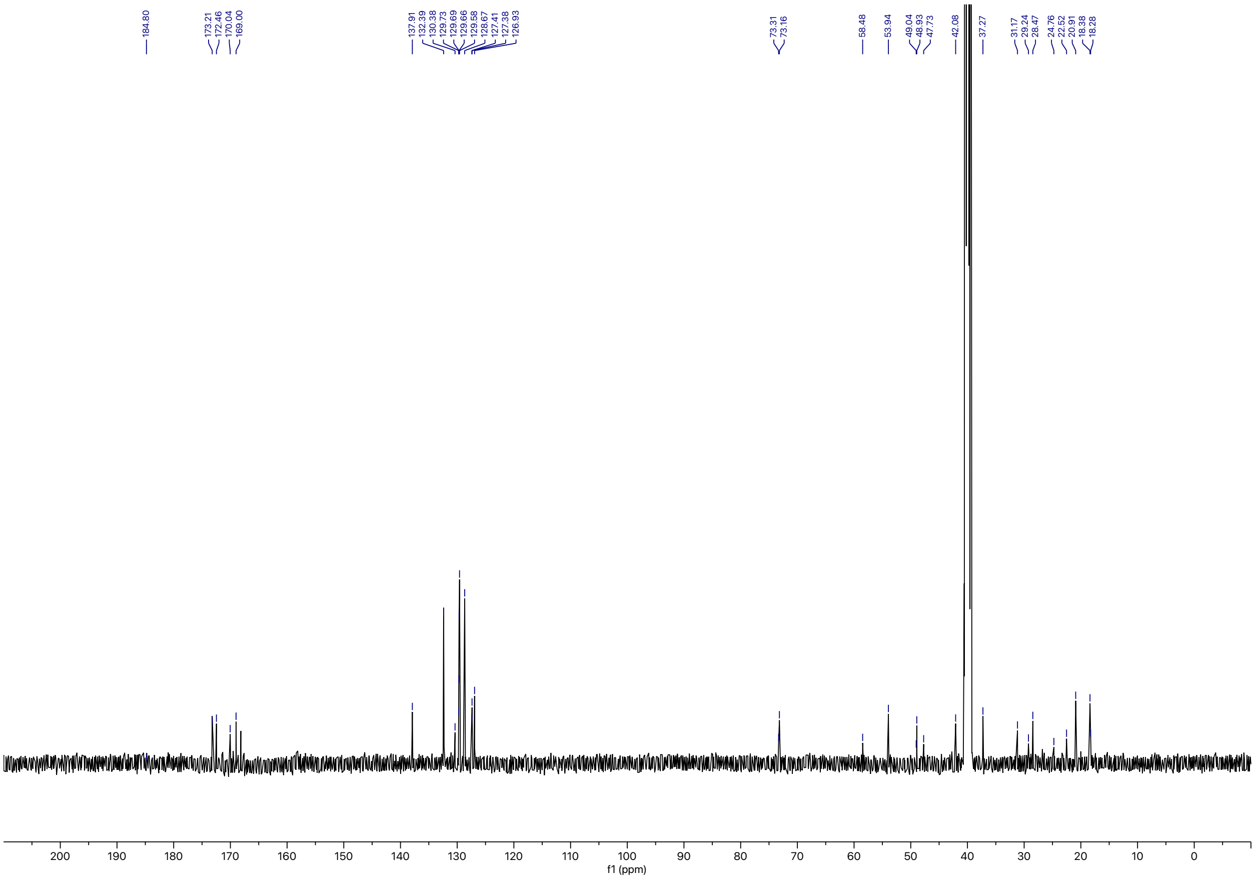

^{1}H NMR (500 MHz, DMSO-*d6*) – Extended view

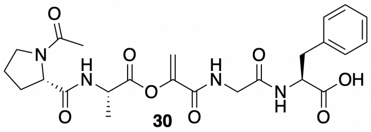

30

Chemical Formula: $C_{24}H_{30}N_4O_8$
Exact Mass: 502.21

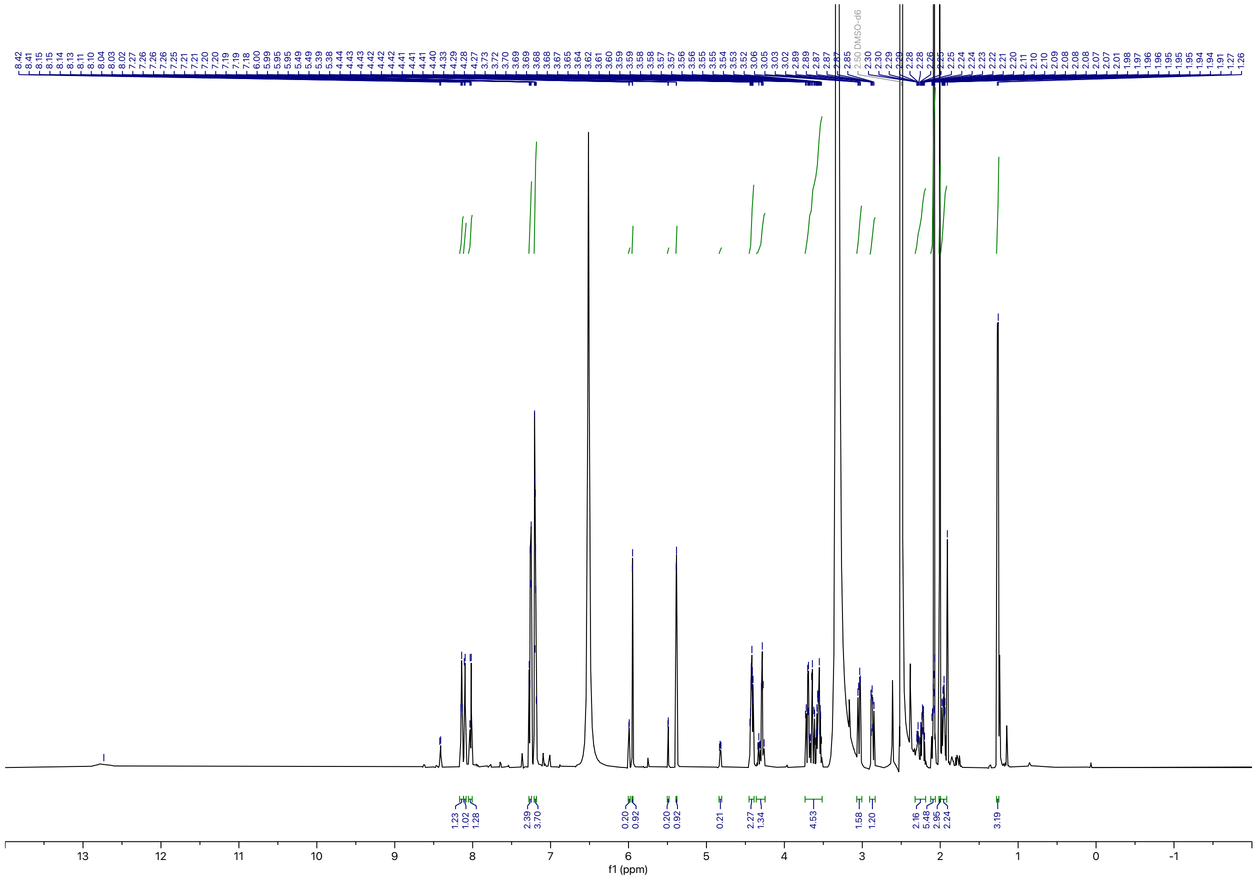

$^{13}C\{^{1}H\}$ NMR (125 MHz, DMSO-*d6*) – Standard view

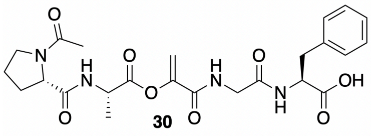

Chemical Formula: $C_{24}H_{30}N_4O_8$
Exact Mass: 502.21

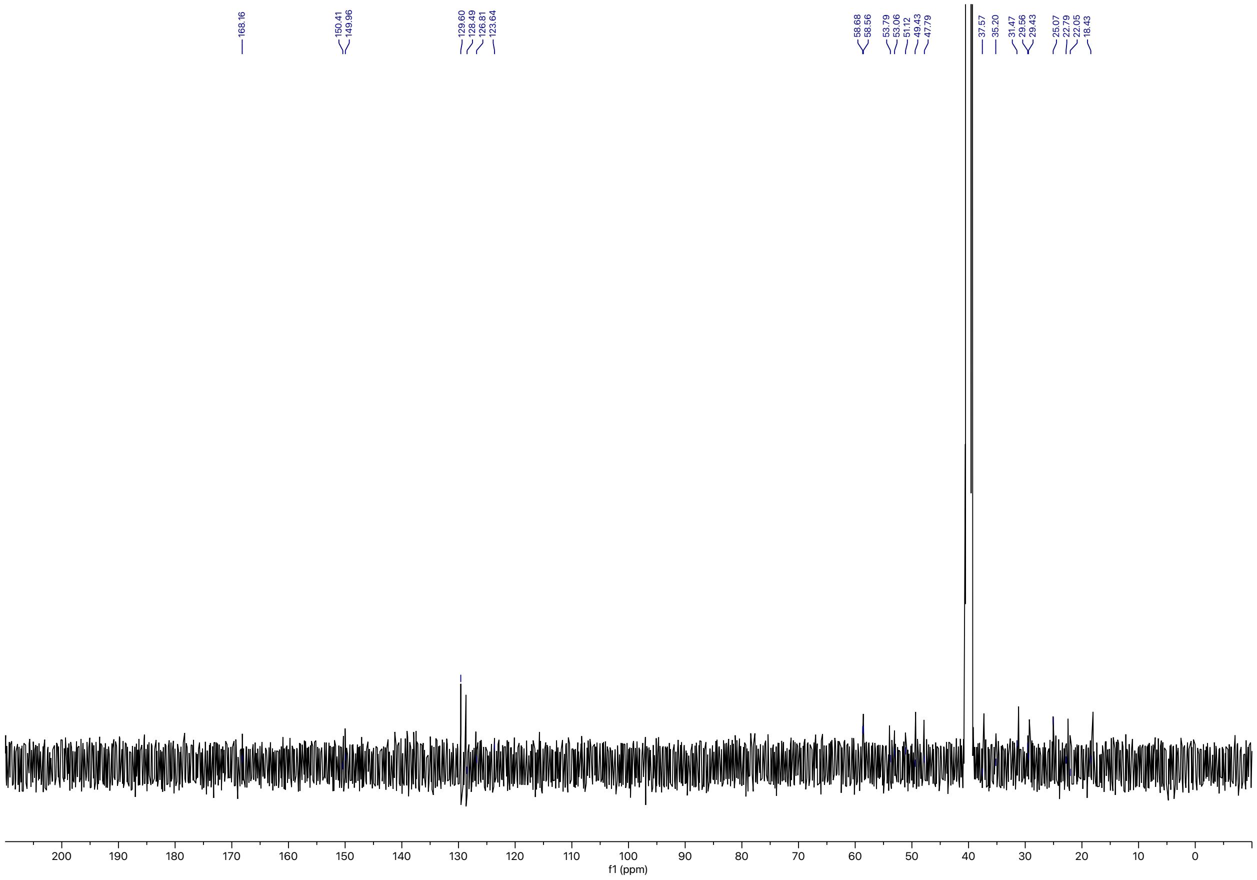

Multiplicity-edited HSQC NMR (125 MHz, chloroform-*d*) – Standard view

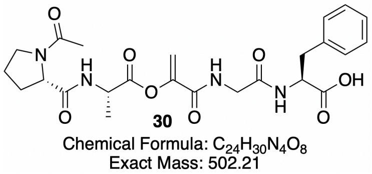

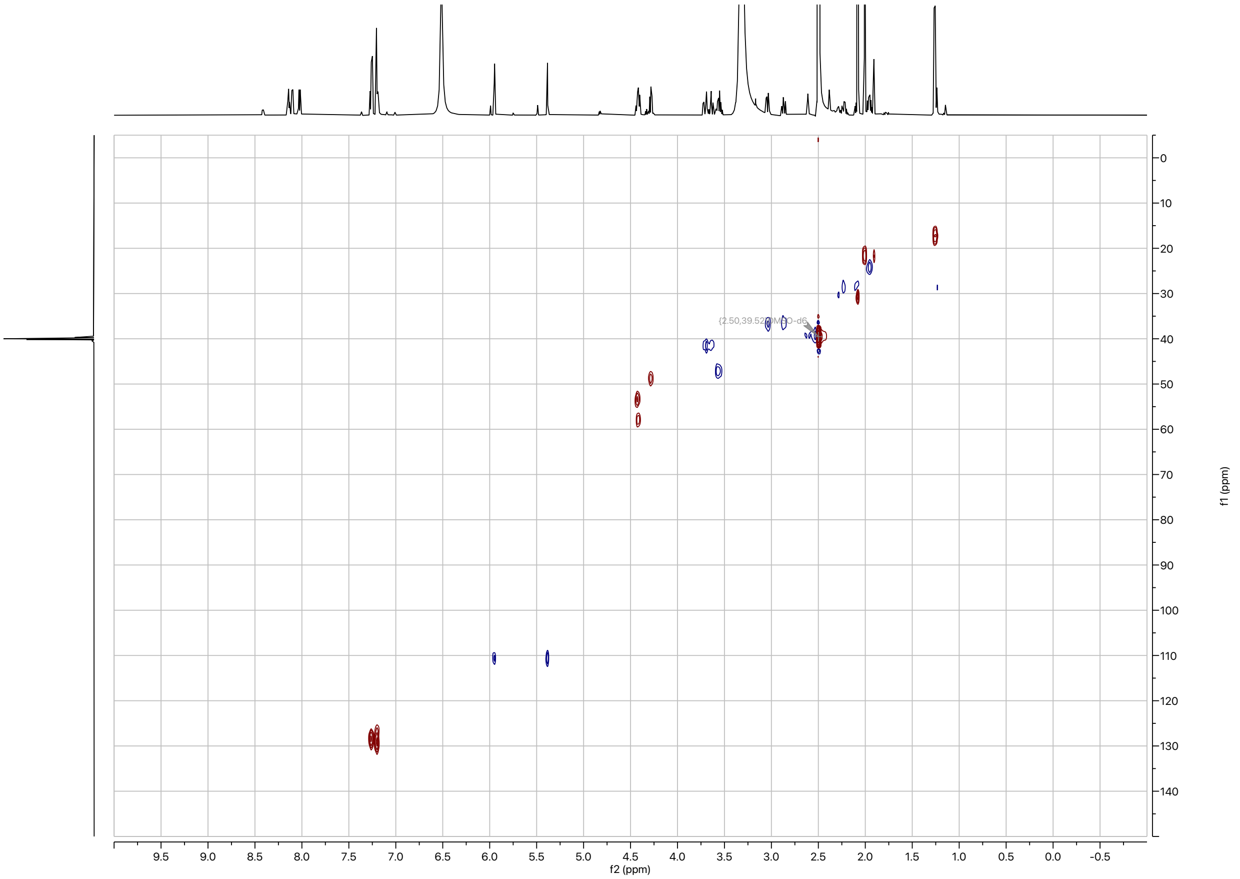

^{1}H NMR (500 MHz, DMSO-*d6*) – Extended view

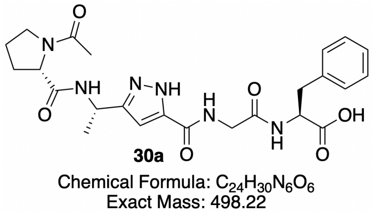

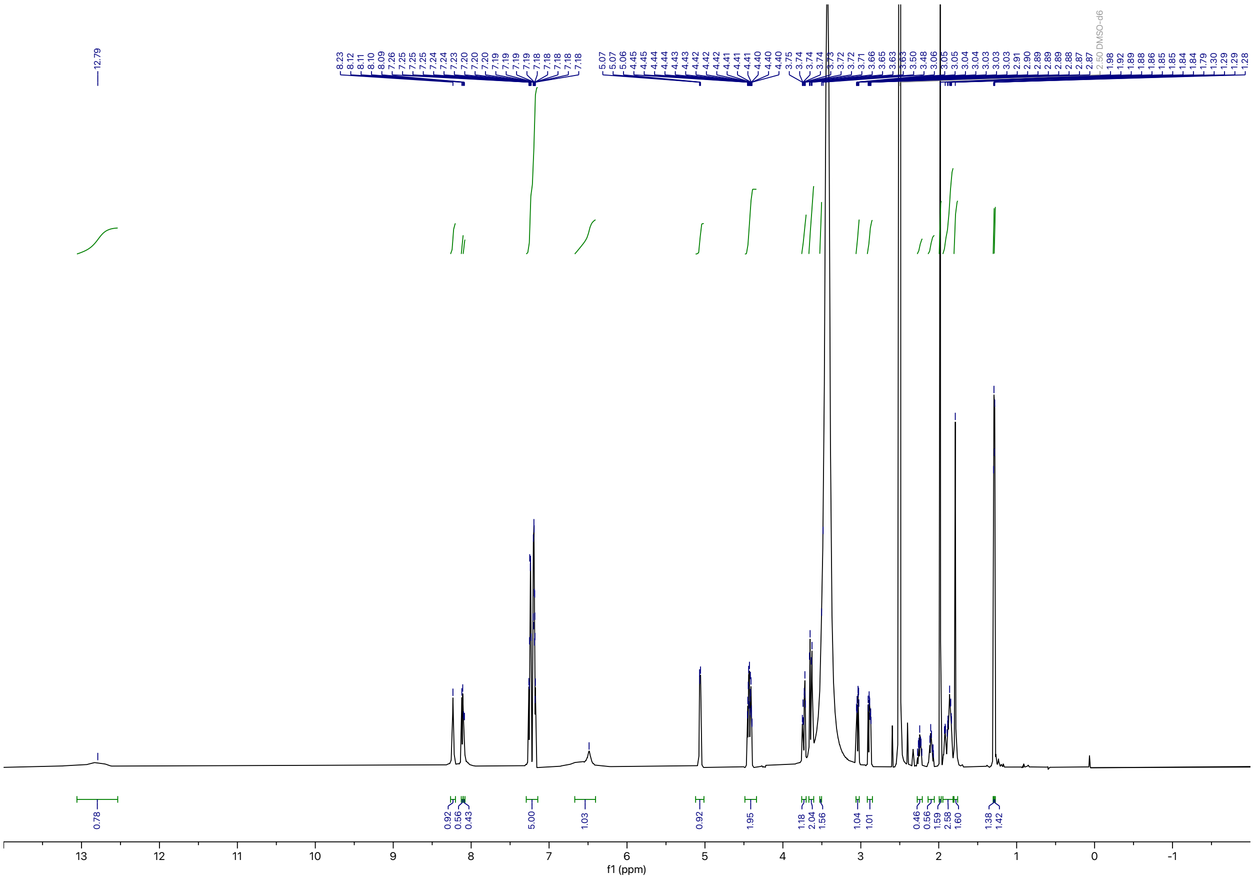

$^{13}C\{^1H\}$ NMR (125 MHz, DMSO-*d6*) – Standard view

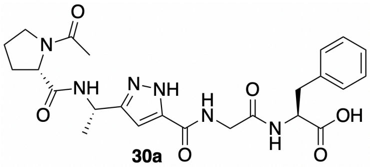

30a

Chemical Formula: $C_{24}H_{30}N_6O_6$

Exact Mass: 498.22

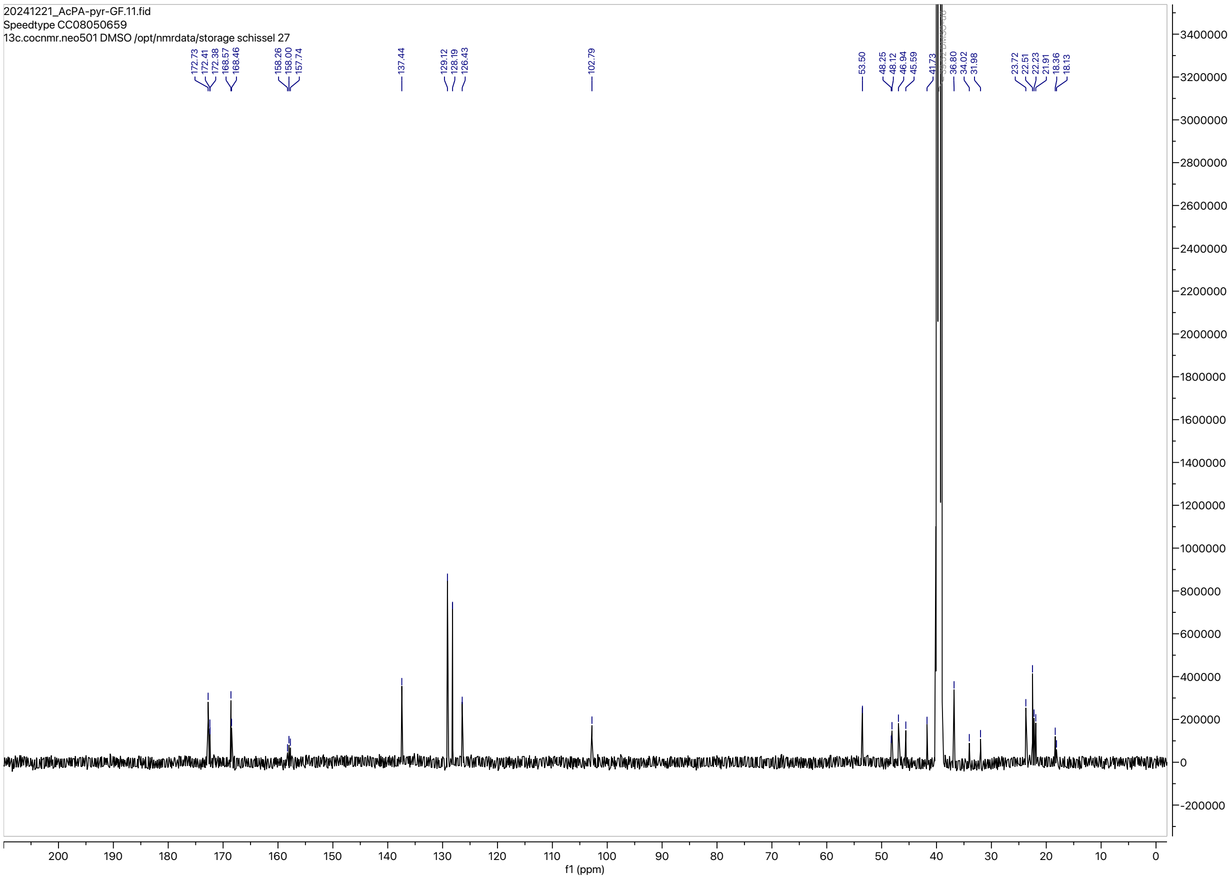

Multiplicity-edited HSQC NMR (125 MHz, chloroform-*d*) – Standard view

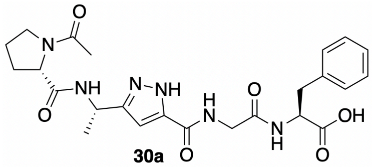

30a

Chemical Formula: $C_{24}H_{30}N_6O_6$
Exact Mass: 498.22

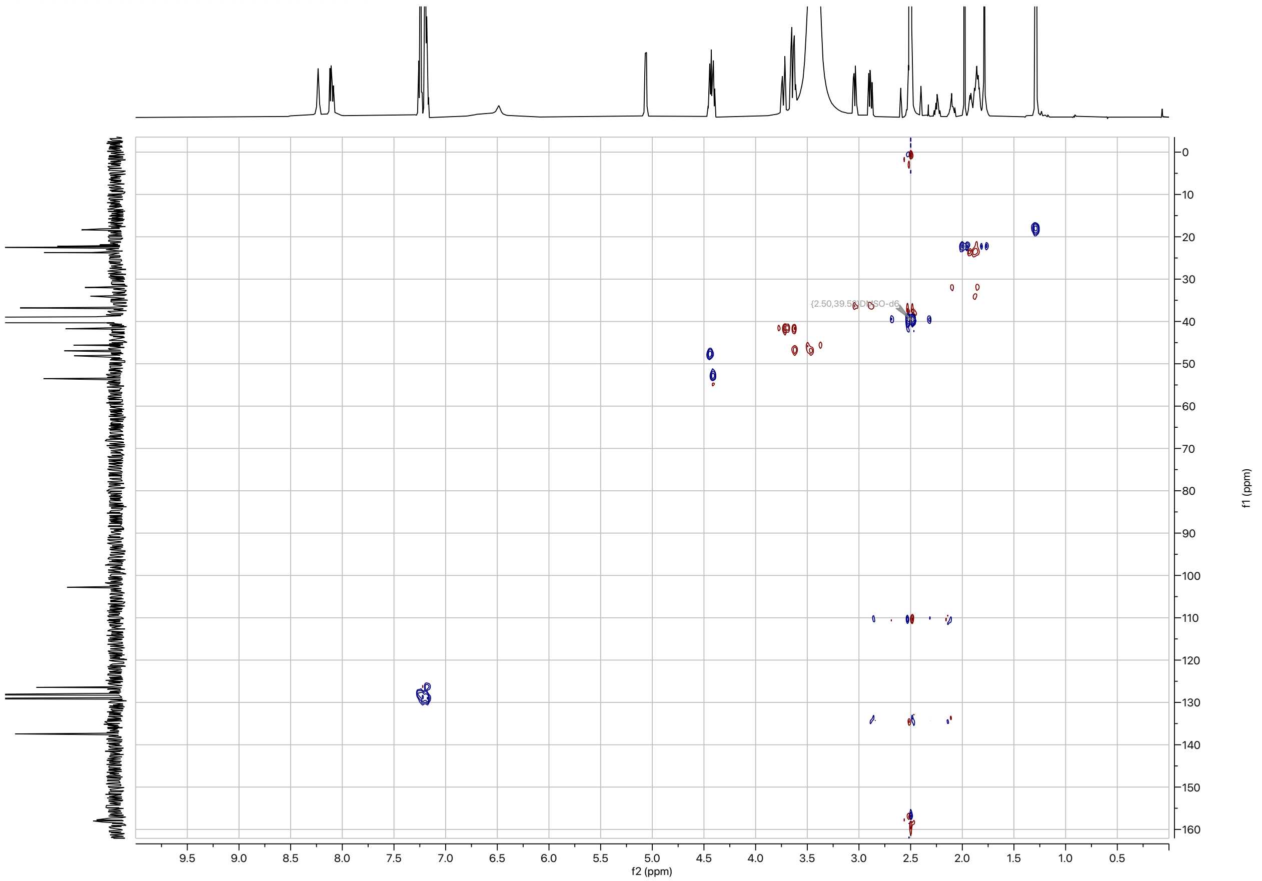

COSY NMR (125 MHz, chloroform-*d*) – Standard view

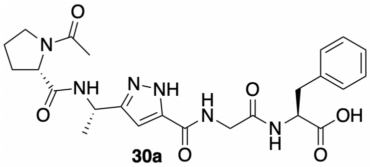

Chemical Formula: $C_{24}H_{30}N_6O_6$
Exact Mass: 498.22

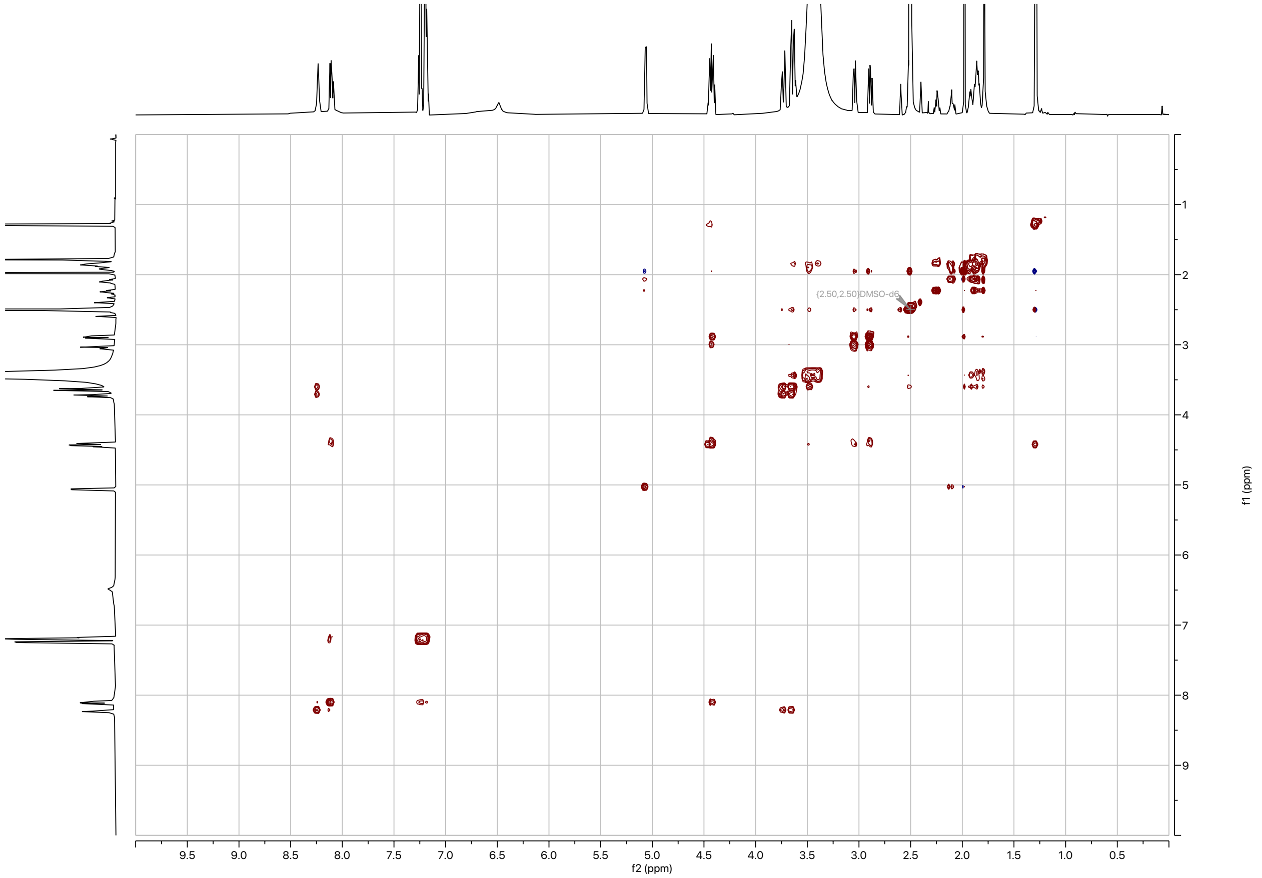

Supplementary Materials Section 4: LC chromatograms & mass spectra

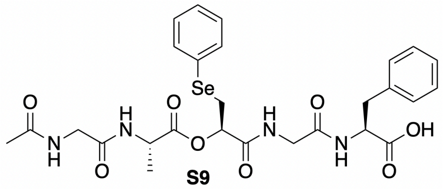

S9

Chemical Formula: $C_{27}H_{32}N_4O_8Se$

Exact Mass: 620.14

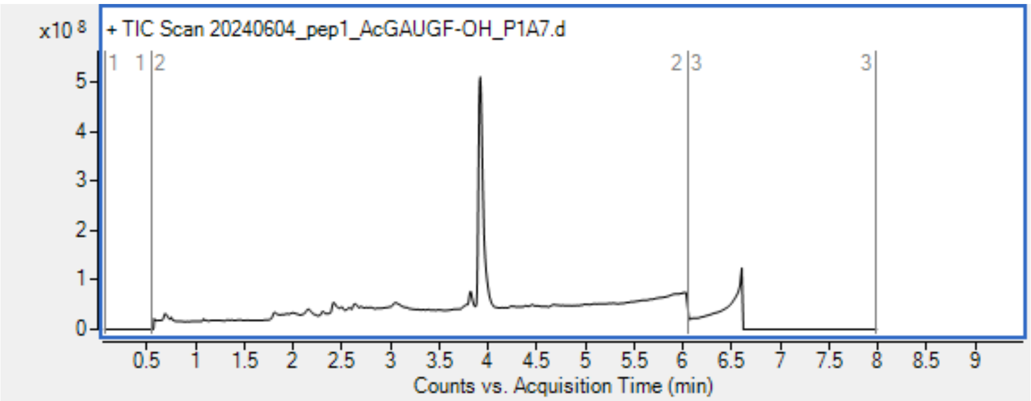

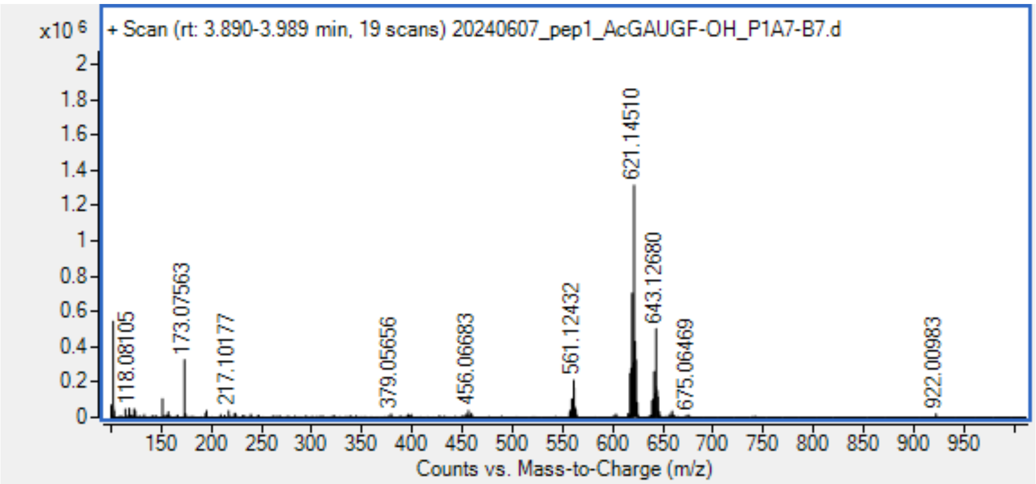

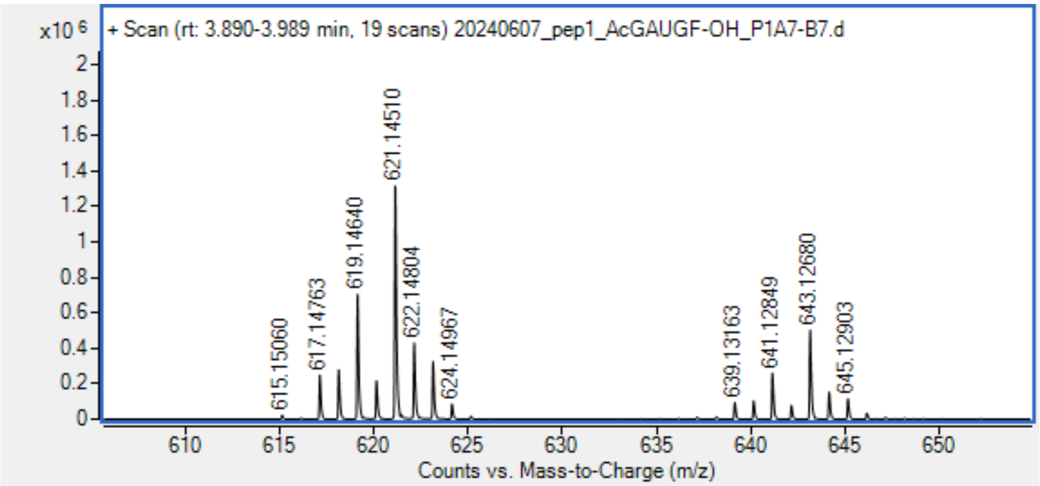

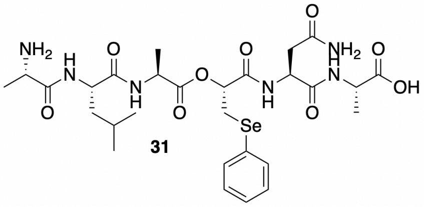

31

Chemical Formula: $C_{28}H_{42}N_6O_9Se$
Exact Mass: 686.22

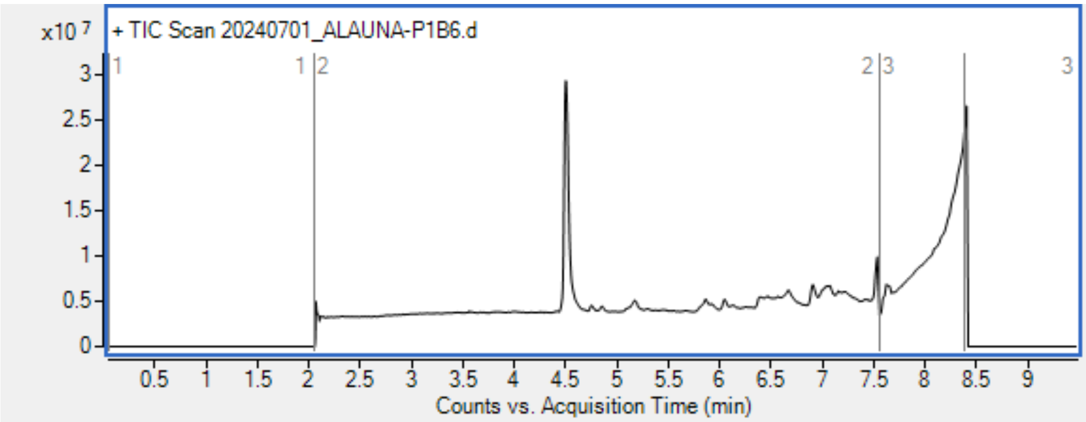

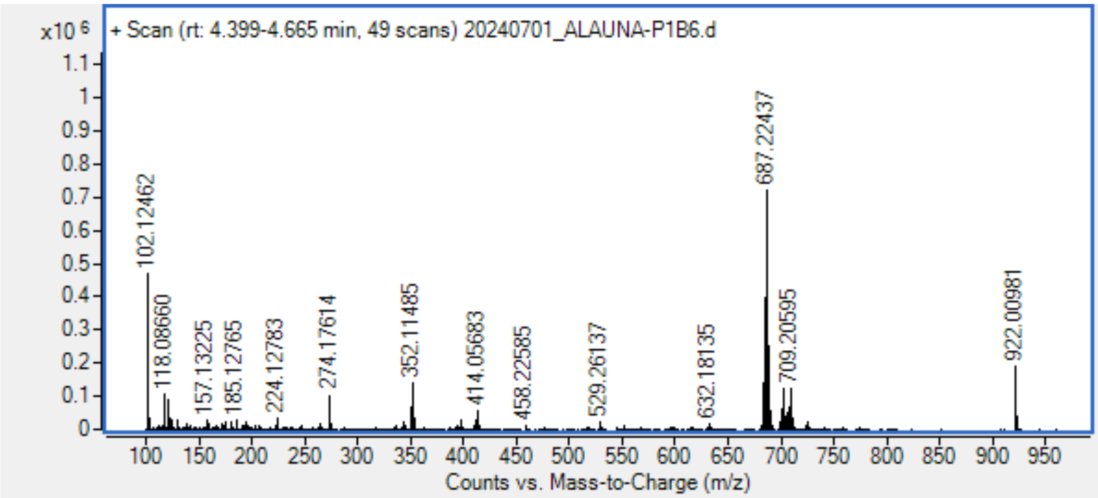

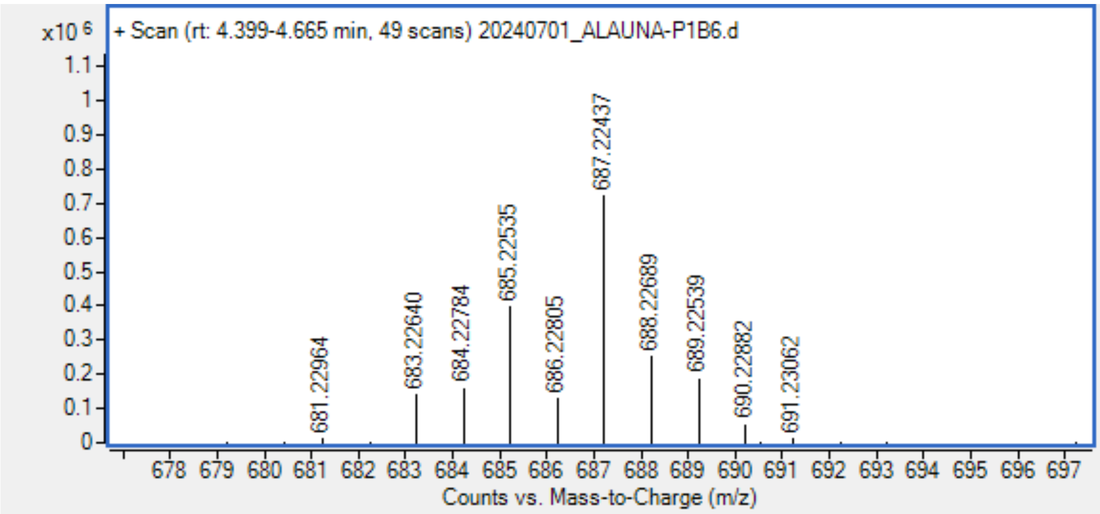

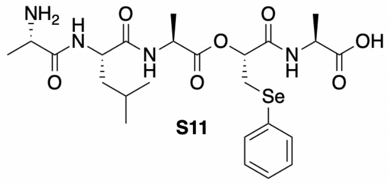

S11

Chemical Formula: $C_{24}H_{36}N_4O_7Se$
Exact Mass: 572.17

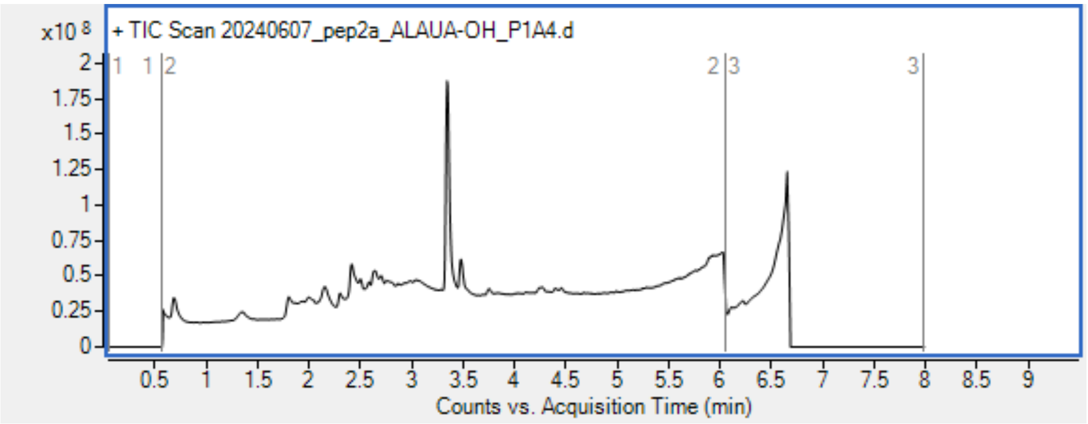

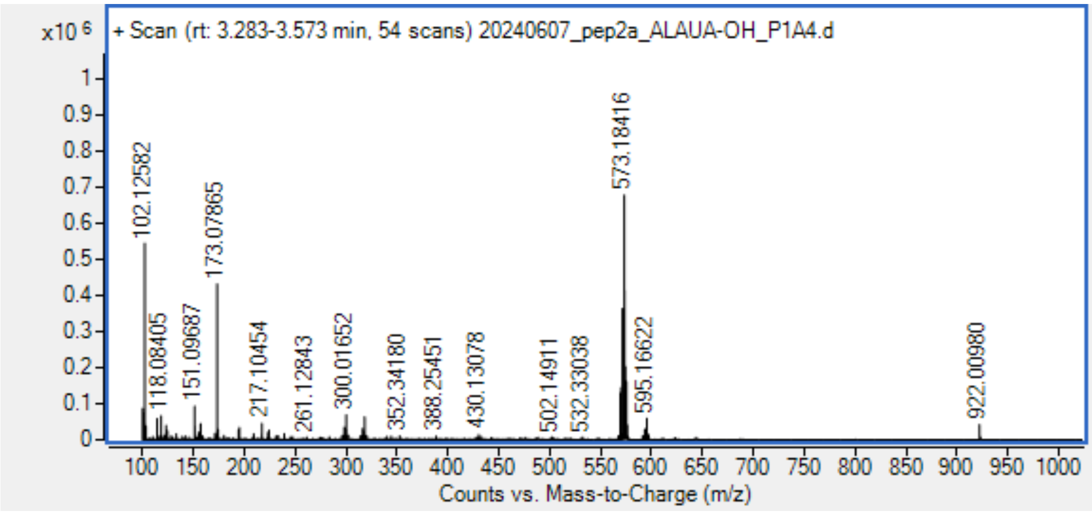

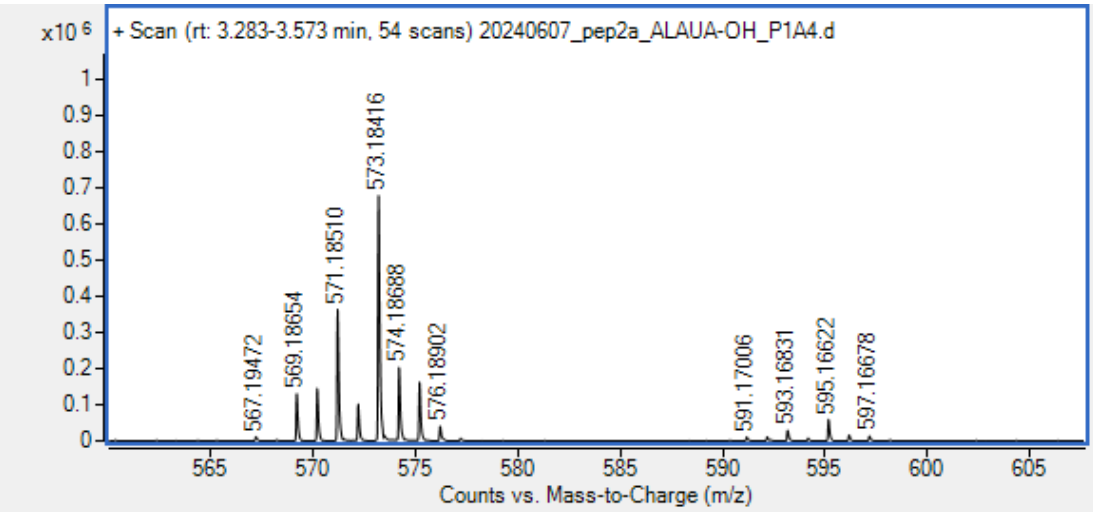

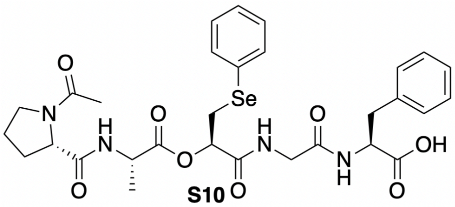

S10

Chemical Formula: $C_{30}H_{36}N_4O_8Se$
Exact Mass: 660.17

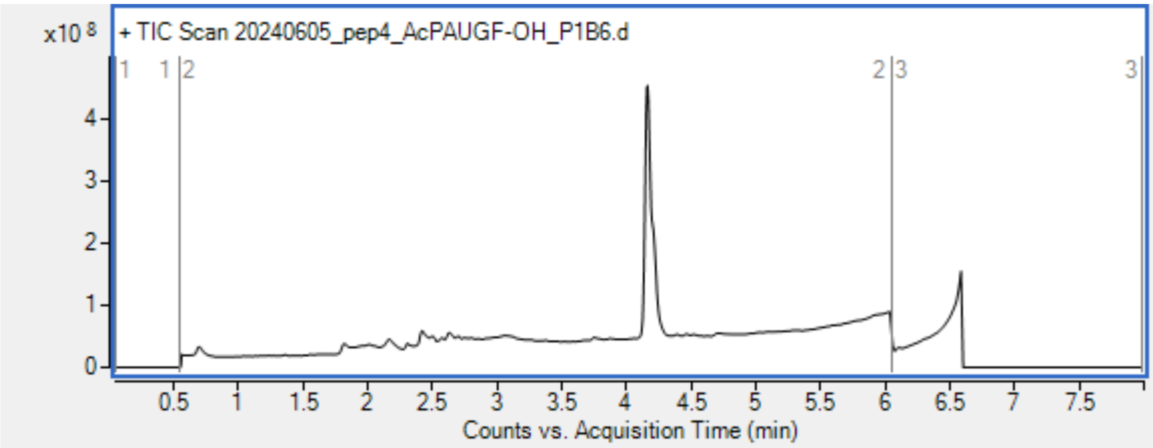

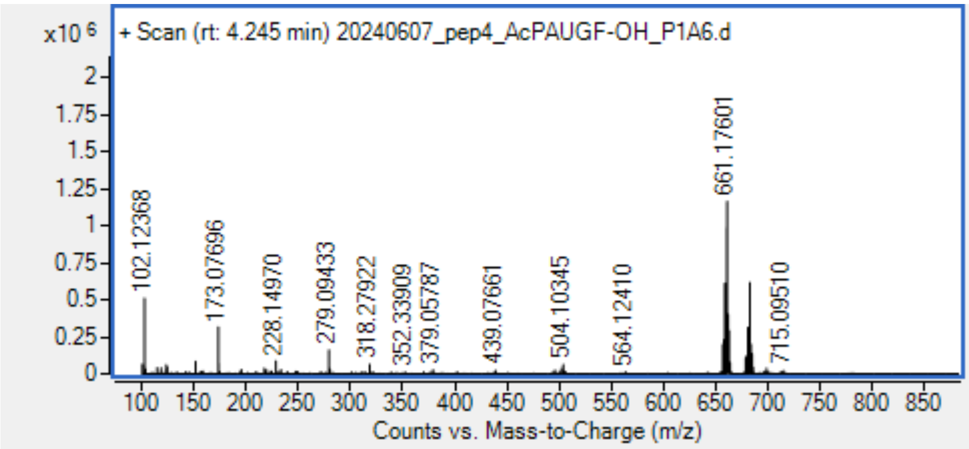

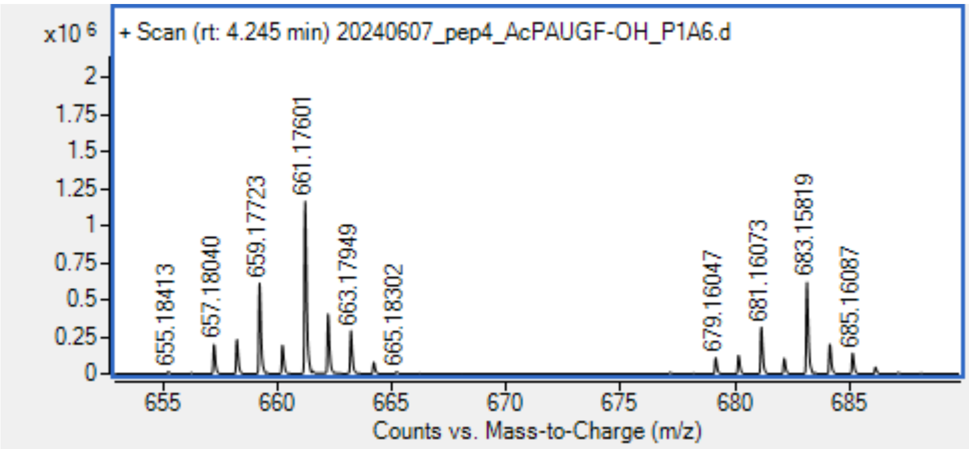

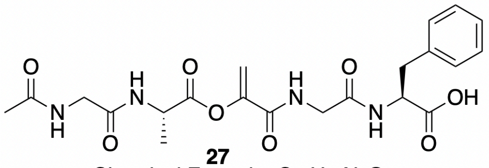

27

Chemical Formula: $C_{21}H_{26}N_4O_8$

Exact Mass: 462.18

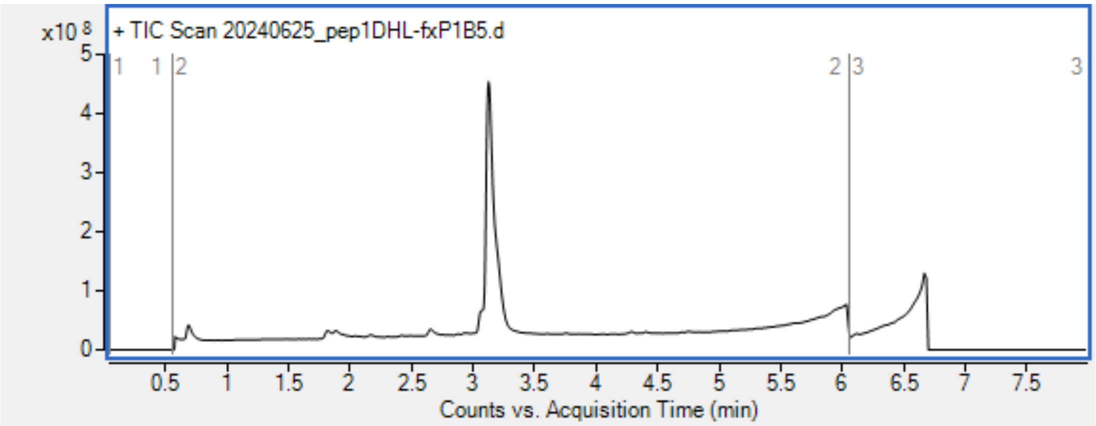

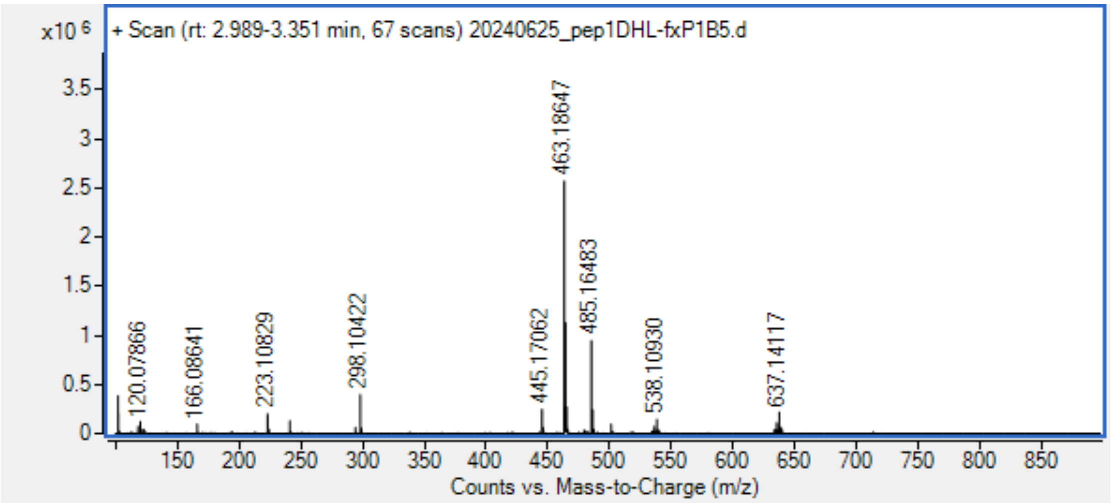

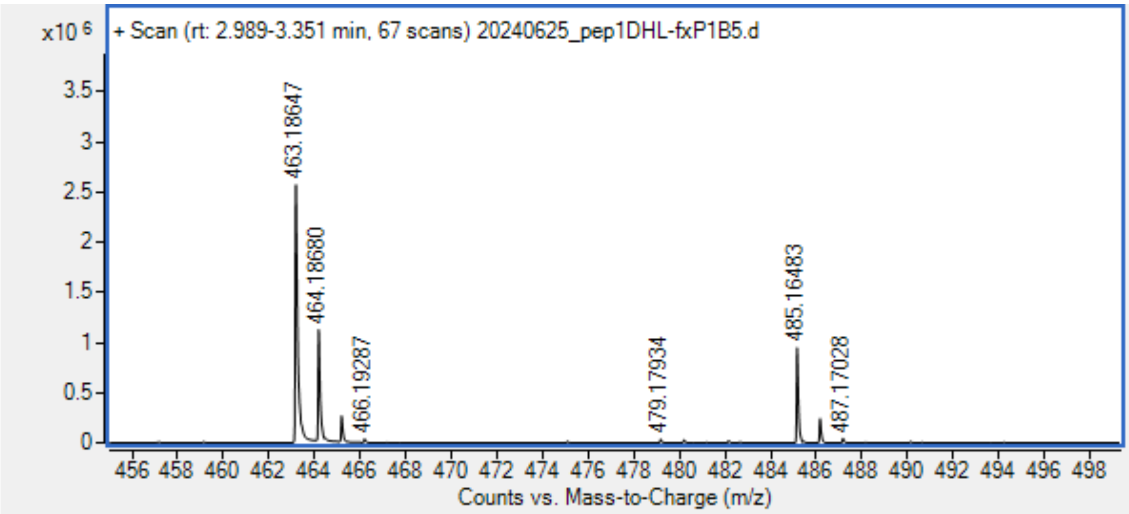

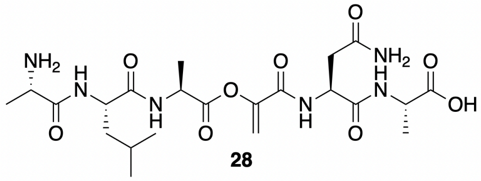

28

Chemical Formula: $C_{22}H_{36}N_6O_9$
Exact Mass: 528.25

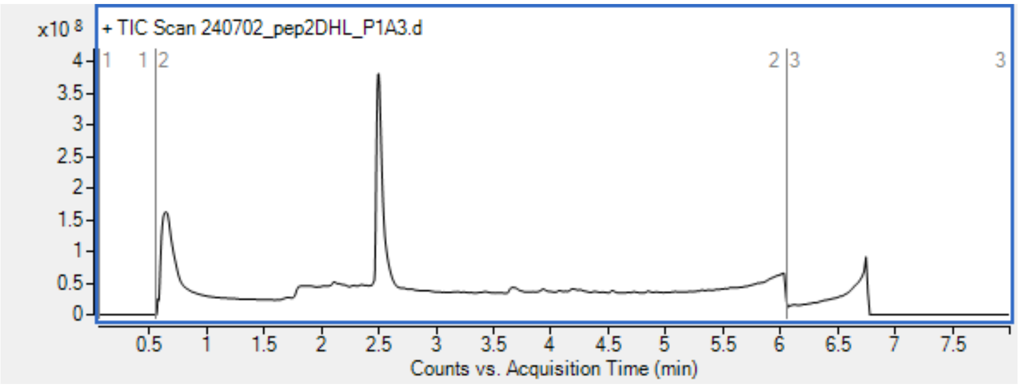

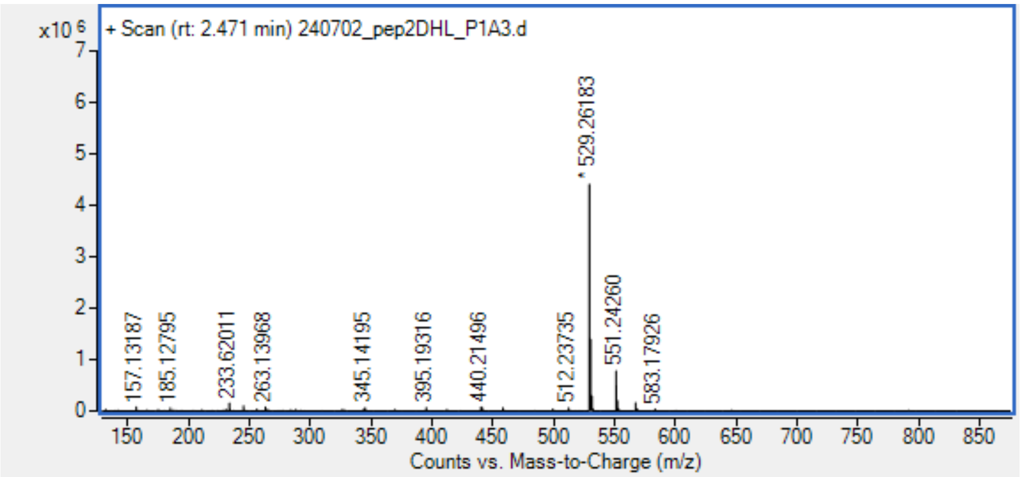

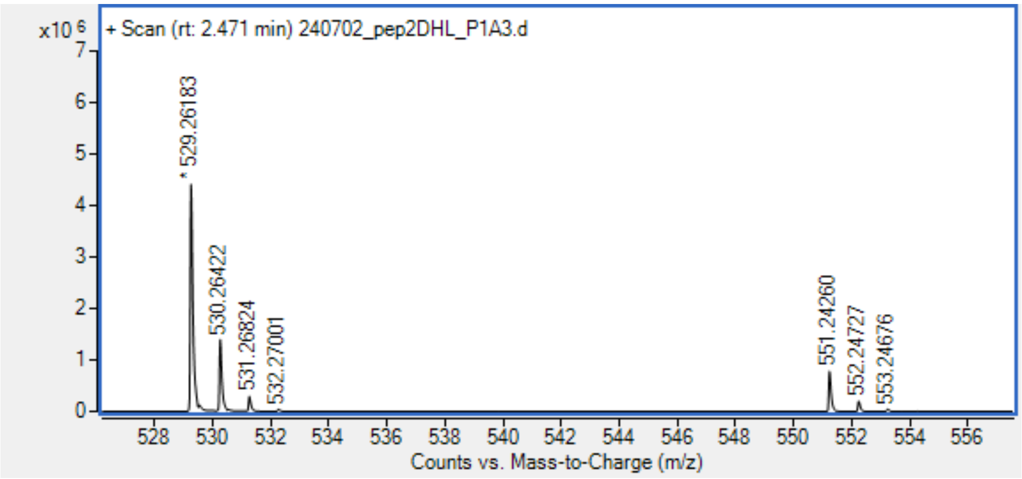

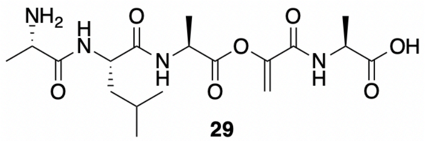

29

Chemical Formula: $C_{18}H_{30}N_4O_7$
Exact Mass: 414.21

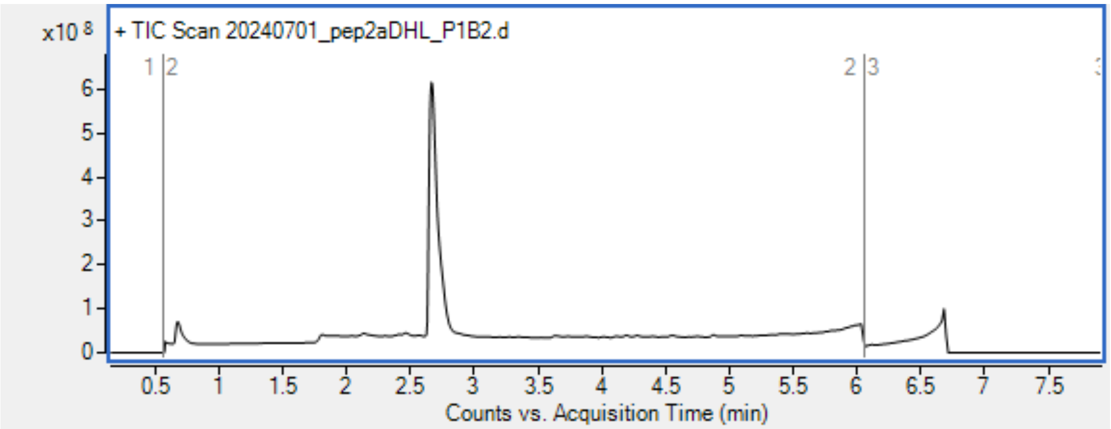

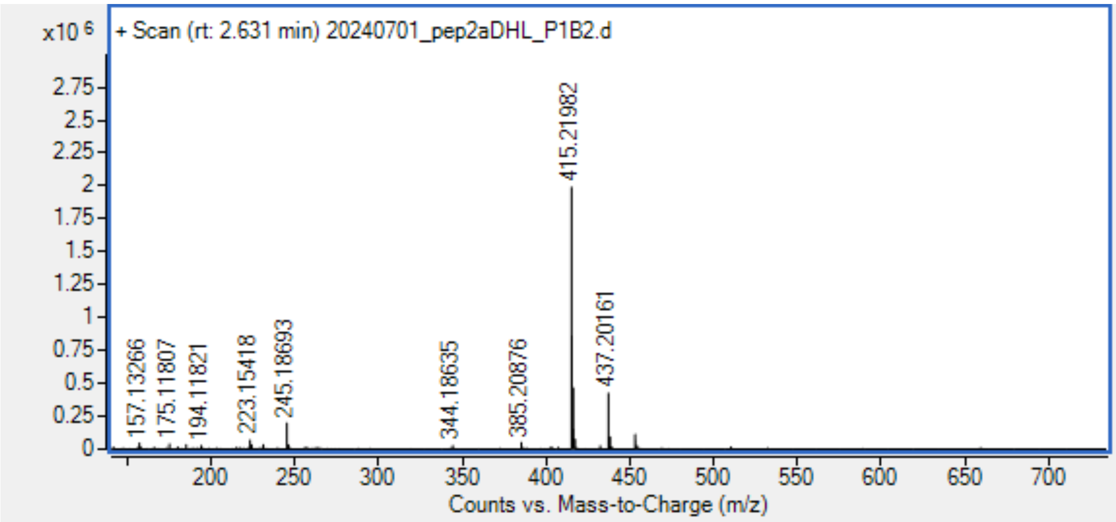

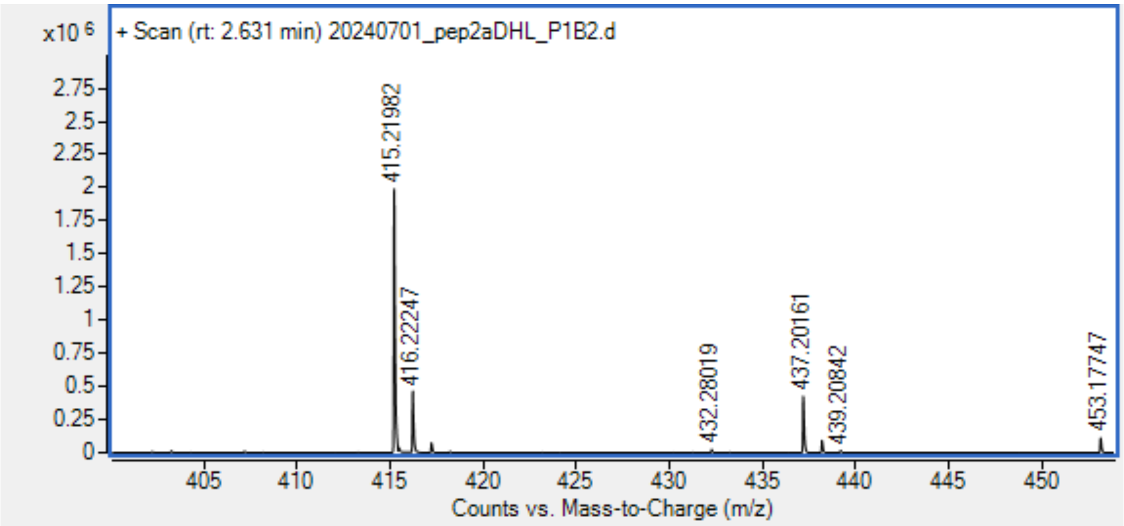

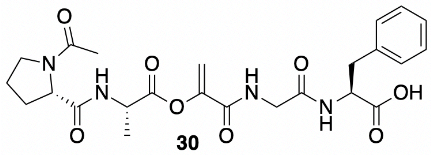

30

Chemical Formula: $C_{24}H_{30}N_4O_8$
Exact Mass: 502.21

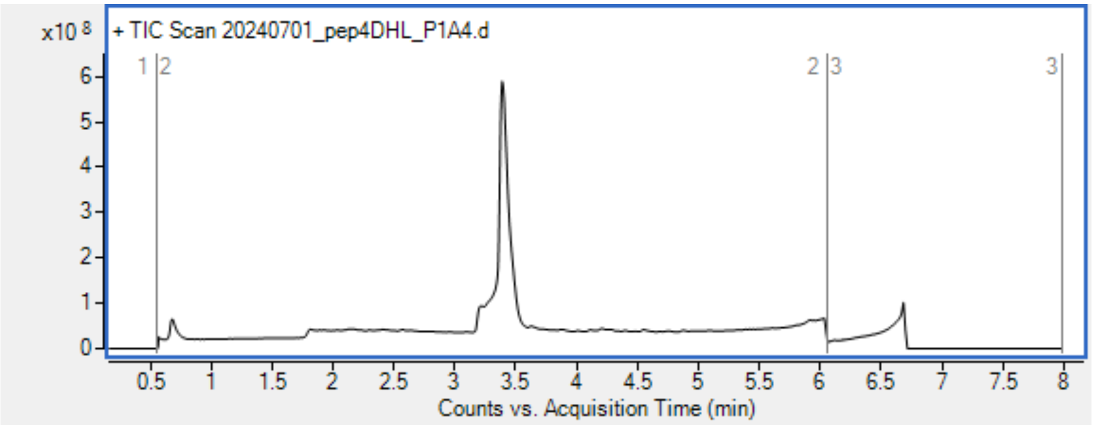

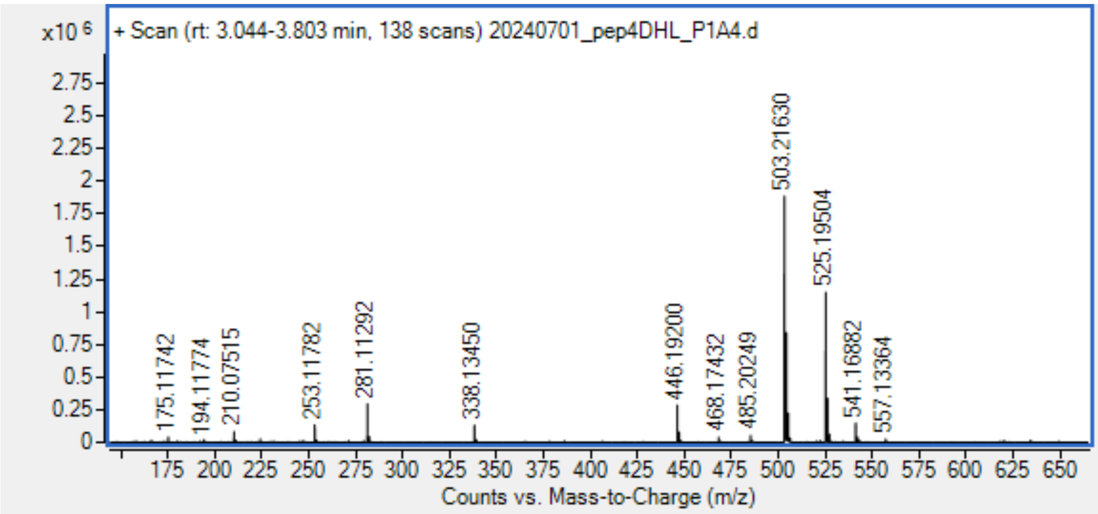

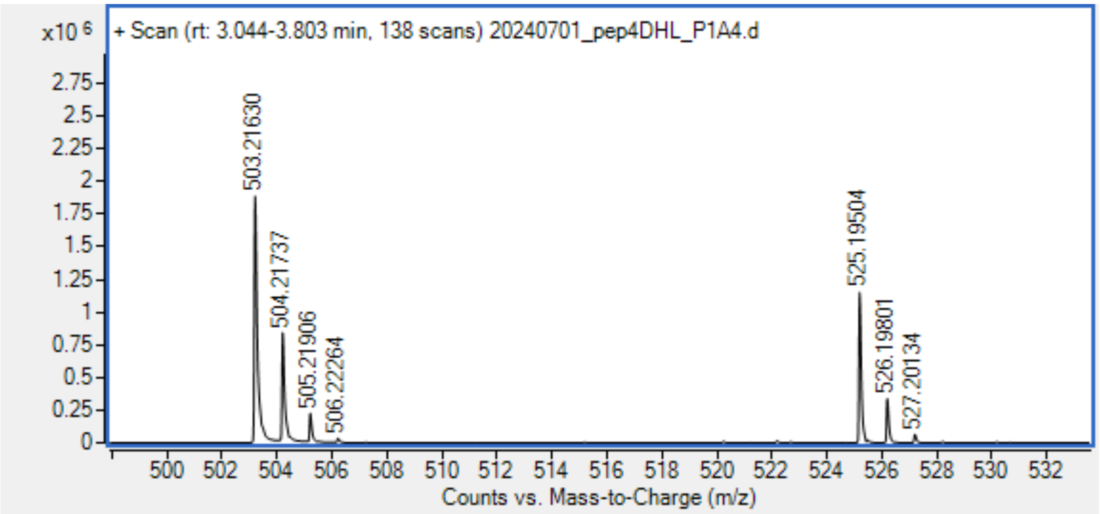

Supplementary Materials Section 5: DFT Coordinates

		wB97M-V/def2-TZVPPD/CPCM	B3LYP-D4/6-31G** CPCM			wB97M-V/def2-TZVPPD/CPCM		
Compound	Isomer	SCFE (hartrees)	ZPE (kcal/mol)	ZPE+H(int) (kcal/mol)	Entropy (cal/(mol•K))	H° (hartrees)	S° (cal/(mol*k))	G° (hartrees)
4	4_1	-514.50881	94.35	101.98	106.60	-514.34630	106.60	-514.39695
	4_2	-514.50953	94.13	101.28	100.84	-514.34813	100.84	-514.39604
	4_3	-514.50658	93.90	101.15	102.99	-514.34538	102.99	-514.39431
	Minimum:	-514.50953				-514.34813	100.84	-514.39604
	E(rel):	0.0				0.0	0.0	0.0
5	5_1	-514.48411	93.39	101.14	106.56	-514.32294	106.56	-514.37357
	5_2	-514.48287	93.70	101.27	103.57	-514.32150	103.57	-514.37071
	5_3	-514.48204	93.45	101.13	105.09	-514.32088	105.09	-514.37081
	5_4	-514.48088	93.47	100.70	103.53	-514.32040	103.53	-514.36959
	5_5	-514.48071	93.50	101.14	104.47	-514.31953	104.47	-514.36917
	Minimum:	-514.48411				-514.32294	106.56	-514.37357
	E(rel):	15.4				15.3	5.7	13.7
6	6_1	-514.51216	95.10	101.99	96.42	-514.34963	96.42	-514.39544
	6_2	-514.51084	94.93	101.88	97.13	-514.34848	97.13	-514.39463
	Minimum:	-514.51216				-514.34963	96.42	-514.39544
	E(rel):	-1.6				-0.9	-4.4	0.4

**7**	**7_1**	**-514.50330**	**94.73**	**101.94**	**100.04**	**-514.34085**	**100.04**	**-514.38838**
	**7_2**	-514.50131	94.47	101.86	102.24	-514.33899	102.24	-514.38756
	**7_3**	-514.49909	94.40	101.77	101.46	-514.33691	101.46	-514.38512
	**7_4**	-514.49924	94.22	101.66	102.53	-514.33724	102.53	-514.38596
	**7_5**	-514.49672	94.12	101.57	101.45	-514.33487	101.45	-514.38307
	**7_6**	-514.49668	94.24	101.70	102.52	-514.33462	102.52	-514.38333
	**7_7**	-514.49604	94.13	101.69	104.03	-514.33398	104.03	-514.38341
	**7_8**	-514.49507	94.45	101.76	101.33	-514.33291	101.33	-514.38105
	**7_9**	-514.49413	94.19	101.63	101.56	-514.33217	101.56	-514.38043
	**Minimum:**	**-514.50330**				**-514.34085**	**100.04**	**-514.38838**
	**E(rel):**	**3.8**				**4.4**	**-0.8**	**4.7**
**8**	**8_1**	**-514.51890**	**93.69**	**101.41**	**104.53**	**-514.35730**	**104.53**	**-514.40696**
	**8_2**	-514.51445	93.87	101.55	105.46	-514.35262	105.46	-514.40273
	**Minimum:**	**-514.51890**				**-514.35730**	**104.53**	**-514.40696**
	**E(rel):**	**-5.7**				**-5.6**	**3.7**	**-6.6**
**9**	**9_1**	**-514.52077**	**95.20**	**102.15**	**98.86**	**-514.35798**	**98.86**	**-514.40495**
	**9_2**	-514.52064	95.38	102.24	97.17	-514.35771	97.17	-514.40387
	**9_3**	-514.52016	95.06	102.07	98.46	-514.35750	98.46	-514.40428
	**Minimum:**	**-514.52077**				**-514.35798**	**98.86**	**-514.40495**
	**E(rel):**	**-6.8**				**-6.0**	**-2.0**	**-5.4**
**10**	**10_1**	**-514.49732**	**93.78**	**101.44**	**106.95**	**-514.33567**	**106.95**	**-514.38648**
	**10_2**	-514.49442	93.56	100.76	102.75	-514.33385	102.75	-514.38266

	**10_3**	-514.49344	93.79	100.93	105.03	-514.33259	105.03	-514.38249
	**10_4**	-514.49415	93.33	100.06	98.56	-514.33469	98.56	-514.38152
	**10_5**	-514.48781	93.86	101.35	104.92	-514.32630	104.92	-514.37615
	**Minimum:**	**-514.49732**				**-514.33567**	**106.95**	**-514.38648**
	**E(rel):**	**7.4**				**7.6**	**6.1**	**5.8**
**11**	**11_1**	**-514.51814**	**94.37**	**102.00**	**104.90**	**-514.35560**	**104.90**	**-514.40544**
	**11_2**	-514.51471	94.30	101.92	104.70	-514.35230	104.70	-514.40205
	**11_3**	-514.51504	94.03	101.83	108.34	-514.35278	108.34	-514.40425
	**11_4**	-514.51378	94.17	101.37	102.63	-514.35224	102.63	-514.40101
	**11_5**	-514.50962	94.43	102.03	105.34	-514.34702	105.34	-514.39706
	**Minimum:**	**-514.51814**				**-514.35560**	**104.90**	**-514.40544**
	**E(rel):**	**-5.2**				**-4.5**	**4.1**	**-5.7**
**12**	**12_1**	**-514.52219**	**94.34**	**101.33**	**100.48**	**-514.36072**	**100.48**	**-514.40846**
	**12_2**	-514.51521	94.45	102.05	108.57	-514.35259	108.57	-514.40418
	**Minimum:**	**-514.52219**				**-514.36072**	**100.48**	**-514.40846**
	**E(rel):**	**-7.7**				**-7.6**	**-0.4**	**-7.5**
